# An international effort towards developing standards for best practices in analysis, interpretation and reporting of clinical genome sequencing results in the CLARITY Challenge

**DOI:** 10.1186/gb-2014-15-3-r53

**Published:** 2014-03-25

**Authors:** Catherine A Brownstein, Alan H Beggs, Nils Homer, Barry Merriman, Timothy W Yu, Katherine C Flannery, Elizabeth T DeChene, Meghan C Towne, Sarah K Savage, Emily N Price, Ingrid A Holm, Lovelace J Luquette, Elaine Lyon, Joseph Majzoub, Peter Neupert, David McCallie Jr, Peter Szolovits, Huntington F Willard, Nancy J Mendelsohn, Renee Temme, Richard S Finkel, Sabrina W Yum, Livija Medne, Shamil R Sunyaev, Ivan Adzhubey, Christopher A Cassa, Paul IW de Bakker, Hatice Duzkale, Piotr Dworzyński, William Fairbrother, Laurent Francioli, Birgit H Funke, Monica A Giovanni, Robert E Handsaker, Kasper Lage, Matthew S Lebo, Monkol Lek, Ignaty Leshchiner, Daniel G MacArthur, Heather M McLaughlin, Michael F Murray, Tune H Pers, Paz P Polak, Soumya Raychaudhuri, Heidi L Rehm, Rachel Soemedi, Nathan O Stitziel, Sara Vestecka, Jochen Supper, Claudia Gugenmus, Bernward Klocke, Alexander Hahn, Max Schubach, Mortiz Menzel, Saskia Biskup, Peter Freisinger, Mario Deng, Martin Braun, Sven Perner, Richard JH Smith, Janeen L Andorf, Jian Huang, Kelli Ryckman, Val C Sheffield, Edwin M Stone, Thomas Bair, E Ann Black-Ziegelbein, Terry A Braun, Benjamin Darbro, Adam P DeLuca, Diana L Kolbe, Todd E Scheetz, Aiden E Shearer, Rama Sompallae, Kai Wang, Alexander G Bassuk, Erik Edens, Katherine Mathews, Steven A Moore, Oleg A Shchelochkov, Pamela Trapane, Aaron Bossler, Colleen A Campbell, Jonathan W Heusel, Anne Kwitek, Tara Maga, Karin Panzer, Thomas Wassink, Douglas Van Daele, Hela Azaiez, Kevin Booth, Nic Meyer, Michael M Segal, Marc S Williams, Gerard Tromp, Peter White, Donald Corsmeier, Sara Fitzgerald-Butt, Gail Herman, Devon Lamb-Thrush, Kim L McBride, David Newsom, Christopher R Pierson, Alexander T Rakowsky, Aleš Maver, Luca Lovrečić, Anja Palandačić, Borut Peterlin, Ali Torkamani, Anna Wedell, Mikael Huss, Andrey Alexeyenko, Jessica M Lindvall, Måns Magnusson, Daniel Nilsson, Henrik Stranneheim, Fulya Taylan, Christian Gilissen, Alexander Hoischen, Bregje van Bon, Helger Yntema, Marcel Nelen, Weidong Zhang, Jason Sager, Lu Zhang, Kathryn Blair, Deniz Kural, Michael Cariaso, Greg G Lennon, Asif Javed, Saloni Agrawal, Pauline C Ng, Komal S Sandhu, Shuba Krishna, Vamsi Veeramachaneni, Ofer Isakov, Eran Halperin, Eitan Friedman, Noam Shomron, Gustavo Glusman, Jared C Roach, Juan Caballero, Hannah C Cox, Denise Mauldin, Seth A Ament, Lee Rowen, Daniel R Richards, F Anthony San Lucas, Manuel L Gonzalez-Garay, C Thomas Caskey, Yu Bai, Ying Huang, Fang Fang, Yan Zhang, Zhengyuan Wang, Jorge Barrera, Juan M Garcia-Lobo, Domingo González-Lamuño, Javier Llorca, Maria C Rodriguez, Ignacio Varela, Martin G Reese, Francisco M De La Vega, Edward Kiruluta, Michele Cargill, Reece K Hart, Jon M Sorenson, Gholson J Lyon, David A Stevenson, Bruce E Bray, Barry M Moore, Karen Eilbeck, Mark Yandell, Hongyu Zhao, Lin Hou, Xiaowei Chen, Xiting Yan, Mengjie Chen, Cong Li, Can Yang, Murat Gunel, Peining Li, Yong Kong, Austin C Alexander, Zayed I Albertyn, Kym M Boycott, Dennis E Bulman, Paul MK Gordon, A Micheil Innes, Bartha M Knoppers, Jacek Majewski, Christian R Marshall, Jillian S Parboosingh, Sarah L Sawyer, Mark E Samuels, Jeremy Schwartzentruber, Isaac S Kohane, David M Margulies

**Affiliations:** 1 Division of Genetics and Genomics, The Research Connection and The Manton Center for Orphan Disease Research, Boston Children’s Hospital, Harvard Medical School, Boston, MA, USA; 2 Claritas Genomics, Boston, MA, USA; 3 Life Technologies, Carlsbad, CA, USA; 4 Center for Biomedical Informatics, Harvard Medical School, Boston, MA, USA; 5 University of Utah School of Medicine, ARUP Laboratories, Salt Lake City, UT, USA; 6 Division of Endocrinology, Boston Children's Hospital, Harvard Medical School, Boston, MA, USA; 7 Microsoft Health Solutions Group, Microsoft Corporation, Seattle, WA, USA; 8 Medical Informatics, Cerner Corporation, Kansas City, MO, USA; 9 Department of Electrical Engineering and Computer Science, Massachusetts Institute of Technology, Cambridge, MA, USA; 10 Duke Institute for Genome Sciences & Policy, Duke University, Durham, NC, USA; 11 Department of Medical Genetics, Children’s Hospitals and Clinics of Minnesota, Minneapolis, MN, USA; 12 Division of Neurology, Nemours Children's Hospital, Orlando, FL, USA; 13 Division of Neurology, Children’s Hospital of Philadelphia, and Department of Neurology, Perelmen School of Medicine, University of Pennsylvania, Philadelphia, PA, USA; 14 Division of Neurology, Children’s Hospital of Philadelphia, Philadelphia, PA, USA; 15 Division of Genetics, Brigham and Woman’s Hospital, Harvard Medical School, Boston, MA, USA; 16 Department of Medical Genetics, University Medical Center Utrecht, Utrecht, The Netherlands; 17 Laboratory for Molecular Medicine, Partners Healthcare Center for Personalized Genetic Medicine, Brigham and Woman’s Hospital, Harvard Medical School, Boston, MA, USA; 18 Center for Biological Sequence Analysis, Department of Systems Biology, Technical University of Denmark, Lyngby, Denmark; 19 Center for Computational Molecular Biology, Brown University, Providence, RI, USA; 20 Department of Genetics, Harvard Medical School, Boston, MA, USA; 21 Pediatric Surgical Research Laboratories, Massachusetts General Hospital, Harvard Medical School, Boston, MA, USA; 22 Analytic and Translational Genetics Unit, Massachusetts General Hospital, Harvard Medical School, Boston, MA, USA; 23 Division of Endocrinology and Center for Basic and Translational Obesity Research, Children’s Hospital Boston, Boston, MA, USA; 24 Genomatix Software GmbH, Bayerstr 85a, Munich, Germany; 25 CeGaT GmbH, Paul-Ehrlich-Str. 17, Tübingen, Germany; 26 Children's Hospital Reutlingen, Reutlingen, Germany; 27 Department of Prostate Cancer Research, Institute of Pathology, University Hospital of Bonn, Bonn, Germany; 28 Iowa Institute of Human Genetics, Carver College of Medicine, The University of Iowa, Iowa City, IA, USA; 29 Iowa Institute of Human Genetics, College of Liberal Arts and Sciences, The University of Iowa, Iowa City, IA, USA; 30 Iowa Institute of Human Genetics, Department of Epidemiology, College of Public Health, The University of Iowa, Iowa City, IA, USA; 31 Howard Hughes Medical Institute, University of Iowa, Iowa City, IA, USA; 32 Iowa Institute of Human Genetics, College of Engineering, The University of Iowa, Iowa City, IA, USA; 33 Iowa Institute of Human Genetics, Department of Biostatistics, College of Public Health, The University of Iowa, Iowa City, IA, USA; 34 SimulConsult, Chestnut Hill, MA, USA; 35 Geisinger Health System, Danville, PA, USA; 36 Nationwide Children’s Hospital, Department of Pediatrics, The Ohio State University, Columbus, Ohio, USA; 37 Clinical Institute of Medical Genetics, Department of Obstetrics and Gynecology, University Medical Centre Ljubljana, Ljubljana, Slovenia; 38 The Scripps Translational Science Institute, The Scripps Research Institute, La Jolla, CA, USA; 39 Department of Molecular Medicine and Surgery, Science for Life Laboratory, Karolinska Institutet, Stockholm, Sweden; 40 Centre for Inherited Metabolic Disorders, Karolinska University Hospital, Stockholm, Sweden; 41 Center for Molecular Medicine, Karolinska Institutet, Stockholm, Sweden; 42 Science for Life Laboratory, Department of Biochemistry and Biophysics, Stockholm University, Stockholm, Sweden; 43 School of Biotechnology, Science for Life Laboratory, Royal Institute of Technology, Stockholm, Sweden; 44 Department of Biosciences and Nutrition, Karolinska Institutet, Huddinge, Sweden; 45 Department of Clinical Genetics, Karolinska University Hospital, Stockholm, Sweden; 46 Department of Human Genetics, Radboud University Nijmegen Medical Centre, Nijmegen, The Netherlands; 47 Sanofi, Cambridge, MA, USA; 48 Seven Bridges Genomics Inc, Cambridge, MA, USA; 49 River Road Bio, Potomac, MD, USA; 50 Computational and Mathematical Biology Genome Institute of Singapore, A*STAR, Singapore, Singapore; 51 Strand Life Sciences, Bangalore, India; 52 Tel Aviv University, Tel Aviv, Israel; 53 Institute for Systems Biology, Seattle, WA, USA; 54 Ingenuity Systems, Redwood City, California, USA; 55 University of Texas Graduate School of Biomedical Sciences, Department of Epidemiology, University of Texas M. D. Anderson Cancer Center, Houston, TX, USA; 56 Division of Next Generation Sequencing, Center for Molecular Imaging, The Brown Foundation Institute of Molecular Medicine, UTHealth, Houston, TX, USA; 57 Department of Molecular and Human Genetics, Baylor College of Medicine, Houston, TX, USA; 58 Regeneron Pharmaceuticals Inc, Tarrytown, NY, USA; 59 Stem Cell Biology and Regenerative Medicine, School of Medicine, Stanford University, Palo Alto, CA, USA; 60 National Heart Lung and Blood Institute, National Institutes of Health, Bethesda, MD, USA; 61 Instituto de Biomedicina y Biotecnología de Cantabria-IBBTEC, Santander, Cantabria, Spain; 62 Consejo Superior de Investigaciones Científicasl (CSIC), Santander, Cantabria, Spain; 63 Universidad de Cantabria, Santander, Cantabria, Spain; 64 SODERCAN, Santander, Cantabria, Spain; 65 Departamento de Biología Molecular, Universidad de Cantabria, Santander, Cantabria, Spain; 66 División de Pediatría. Departamento de Ciencias Médicas y Quirúrgicas, Universidad de Cantabria Santander, Cantabria, Spain; 67 Hospital Universitario Marqués de Valdecilla -IFIMAV (Instituto de Formación e Investigación Marqués de Valdecilla), Santander, Cantabria, Spain; 68 CIBER Epidemiología y Salud Pública (CIBERESP), Santander, Cantabria, Spain; 69 Instituto de Formación e Investigación Marqués de Valdecilla (IFIMAV), Santander, Cantabria, Spain; 70 Omicia, Inc, Emeryville, CA, USA; 71 Real Time Genomics, Inc, San Bruno, CA, USA; 72 InVitae (formerly Locus Development), 458 Brannan Street, San Francisco, CA 94107, USA; 73 Stanley Institute for Cognitive Genomics, Cold Spring Harbor Laboratory, New York, NY, USA; 74 Utah Foundation for Biomedical Research, Salt Lake City, UT, USA; 75 Department of Pediatrics, University of Utah School of Medicine, Salt Lake City, Utah, USA; 76 Division of Cardiology and Department of Biomedical Informatics, University of Utah School of Medicine, Salt Lake City, Utah, USA; 77 Department of Human Genetics, Eccles Institute of Human Genetics, University of Utah School of Medicine, Salt Lake City, Utah, USA; 78 Biomedical Informatics, University of Utah School of Medicine, Salt Lake City, Utah, USA; 79 Department of Biostatistics, Yale School of Public Health, New Haven, CT, USA; 80 Program of Computational Biology and Bioinformatics, Yale University, New Haven, CT, USA; 81 Biostatistics Resource, Keck Laboratory, Yale University, New Haven, CT, USA; 82 Departments of Neurosurgery and Neurobiology, Yale University School of Medicine, New Haven, CT, USA; 83 Department of Genetics, Yale University School of Medicine, New Haven, CT, USA; 84 Department of Molecular Biophysics and Biochemistry, WM Keck Foundation Biotechnology Resource Laboratory, Yale University, New Haven, CT, USA; 85 Pearlgen, Inc, Durham, NC, USA; 86 Novocraft Technologies Sdn Bhd, Selangor, Malaysia; 87 Children’s Hospital of Eastern Ontario Research Institute, University of Ottawa, Ottawa, ON, Canada; 88 Alberta Children’s Hospital Research Institute (ACHRI), University of Calgary, Calgary, AB, Canada; 89 Department of Medical Genetics and Alberta Children’s Hospital Research Institute (ACHRI), University of Calgary, Calgary, AB, Canada; 90 Centre of Genomics and Policy, McGill University, Montreal, QC, Canada; 91 McGill University and Genome Quebec Innovation Centre, Montreal, QC, Canada; 92 McLaughlin Centre, University of Toronto, The Centre for Applied Genomics, The Hospital for Sick Children, Toronto, ON, Canada; 93 Department of Medicine, Centre de Recherche du CHU Ste-Justine, University of Montreal, Montreal, QC, Canada; 94 Current Address: Zoological Department, Naturhistorisches Museum, Vienna, Austria

## Abstract

**Background:**

There is tremendous potential for genome sequencing to improve clinical diagnosis and care once it becomes routinely accessible, but this will require formalizing research methods into clinical best practices in the areas of sequence data generation, analysis, interpretation and reporting. The CLARITY Challenge was designed to spur convergence in methods for diagnosing genetic disease starting from clinical case history and genome sequencing data. DNA samples were obtained from three families with heritable genetic disorders and genomic sequence data were donated by sequencing platform vendors. The challenge was to analyze and interpret these data with the goals of identifying disease-causing variants and reporting the findings in a clinically useful format. Participating contestant groups were solicited broadly, and an independent panel of judges evaluated their performance.

**Results:**

A total of 30 international groups were engaged. The entries reveal a general convergence of practices on most elements of the analysis and interpretation process. However, even given this commonality of approach, only two groups identified the consensus candidate variants in all disease cases, demonstrating a need for consistent fine-tuning of the generally accepted methods. There was greater diversity of the final clinical report content and in the patient consenting process, demonstrating that these areas require additional exploration and standardization.

**Conclusions:**

The CLARITY Challenge provides a comprehensive assessment of current practices for using genome sequencing to diagnose and report genetic diseases. There is remarkable convergence in bioinformatic techniques, but medical interpretation and reporting are areas that require further development by many groups.

## Background

The transition of genomics from research into clinical practice has begun, predicated on rapidly improving technology, data analysis methods, and more recently and importantly, standardization [1,2]. Methods and tools for genomic diagnostics have quickly evolved to encompass all of the processes from consenting, through data generation and analysis, to interpretation, prioritization, and revisable reporting [3]. Nonetheless, there is not currently a widely accepted set of published standards to enable the consistent and widespread use of genomics in the practice of medicine.

There have been a growing number of publicized successes in the application of genomic sequencing and interpretations for children with rare diseases of unknown etiology and patients with refractory cancers [4-11]. This has led to a growing expectation that clinical whole exome sequencing (WES) or whole genome sequencing (WGS) services will soon be standard practice for a much larger population of patients. Unlike other data-intensive diagnostic modalities, such as magnetic resonance imaging (MRI), there are no standards for the use of computational tools to analyze the outputs of different next-generation sequencing (NGS) technologies for patient care [12]. There is a large methodological armamentarium for assembling genomic reads into a sequence, detecting variation, interpreting the clinical significance of specific sequence variants, and compiling a clinically usable report. Yet just how these methods are used in context, and in what combination, all critically impact the quality of genomically informed diagnoses. For example, many studies have utilized WES datasets essentially as large gene panels, interrogating data for only a small set of candidate genes determined based on clinical presentations [13], while others have utilized the entire datasets to identify and qualify mutations anywhere in the genome [9].

The present study was initially conceived at the *2010 Clinical Bioinformatics Summit* hosted in Boston by Harvard University, the Children’s Hospital Informatics Program, and Harvard Medical School Center for Biomedical Informatics. The conference was attended by a wide range of stakeholders who discussed what it would take to attain a consistent and safe standard for clinical-grade genome-wide data interpretation. One of the consensus outcomes of this conference was the catalytic effect that a full clinical-grade genomic diagnostic challenge contest would have upon the emergence of both *de facto* and formal standards for genome-scale diagnostics.

This contest – dubbed the CLARITY Challenge (Children’s Leadership Award for the Reliable Interpretation and Appropriate Transmission of Your Genomic Information) – was hosted by the Manton Center for Orphan Disease Research at Boston Children’s Hospital and the Center for Biomedical Informatics at Harvard Medical School [14]. Prizes totaling USD 25,000 were made available to the team or teams that could best analyze, interpret and report, in a clinically meaningful format, the results of parallel WES and WGS. The inspiration for CLARITY arose from the marked success of contests as a technique to focus a community on a particularly interesting and high-impact problem (e.g., various X Prizes). Successful competitions have accelerated progress in protein folding, including the MATLAB Protein Folding Contest [15] and the International Protein Folding Competition (CASP) [16], gene identification, such as EGASP [17], and *in silico* tools for predicting variant pathogenicity such as the CAGI experiment [18]. Contests have been used to evoke ‘co-opetition’ – a collaboration centered on competition – in the hopes of crystallizing best practices and, thereby, accelerating the field. Comparative analysis is not new to this field either, as projects such as the 1000 Genomes Project [19] have provided the opportunity to compare technological and analytic methods across platforms and pipelines; its Exon Pilot project compared technologies from 454 Life Sciences, a Roche company (Branford, CT, USA), Applied Biosystems (Carlsbad, CA, USA), and Illumina Inc (San Diego, CA, USA), comparing capture biases, coverage fluctuations, indel alignment issues, population biases, and sequencing errors [20]. More recently, a prominent paper compared the accuracy and sensitivity of results obtained using an Illumina Hiseq 2000 instrument and Complete Genomics’ WGS service [21]. But there has not been a competition that has focused on the entire front-to-back process of applying NGS to patient care in a manner suitable for large-scale clinical adoption.

Admittedly, there are limitations to this method. To keep the scope of the competition manageable, it was focused largely on assessing the processes of variant annotation and subsequent medical interpretation and reporting, and no attempt was made to represent a range of clinical conditions and genetic models, or deal with the challenges of assessing clinical similarities amongst different presentations. Thus, the contest did not fully assess the real world challenges of finding causal mutations, but instead focused on comparative methods by which variants are called and assessed bioinformatically. Also outside the scope of the CLARITY Challenge are issues related to the importance of direct experimental evaluation of the functional consequence of mutation, which is a key part of the interpretation of novel variants and where improvement is also needed.

We present here a survey of the various methods used in the Challenge and summarize the opinions and attitudes of the contestants after the fact regarding the practice of clinical-grade genome-scale diagnostics for clinical practice.

## Results and discussion

Three families were identified by the Manton Center for Orphan Disease Research to serve as test cases for the CLARITY challenge on the basis of having a child with clinical manifestations and/or pedigree structure suggestive of a likely genetic disease (Table 1). The clinical study reported here was performed under the auspices of the Boston Children’s Hospital Institutional Review Board (IRB) under Protocol IRB-P00000167. The organizing team worked closely with the IRB to define a protocol that protected the families’ interests, as well as the patients’ rights and prerogatives, yet allowed them to share their de-identified medical histories and DNA sequences with teams of qualified competitors around the world.

**Table 1:** Clinical findings in challenge families

**Family**	**Diagnosis**	**Clinical history**
1	Centronuclear myopathy and bilateral sensorineural hearing loss	• 10-year-old male diagnosed with centronuclear myopathy at 13 months based on clinical exam and muscle biopsy findings
		• Uses a G-tube for supplemental feeding
		• Uses nighttime ventilation support
		• Able to walk limited distances (up to four city blocks), to run and to climb stairs with use of a railing
		• Bilateral mild low to mid-frequency hearing loss
		• No contributory family history
2	Right-sided structural heart defects and conduction defects	• Multiple family members with a variety of right-sided cardiac defects ranging in severity
		• Proband is a 5-year-old female with history of a right ventricle mass that resolved spontaneously, persistent right bundle branch block (RBBB) and slightly dilated ascending aorta
		• Mother has the same condition, not requiring intervention
		• Maternal uncle has a pacemaker for Type II AV block and a history of pulmonary stenosis
		• Maternal aunt died in neonatal period due to cardiac defects
		• Maternal first cousin died in neonatal period due to a complex congenital cardiac defects involving hypertrophied right ventricle, tricuspid valve atresia, and second degree heart block
3	Nemaline myopathy	• 7-year-old male diagnosed with nemaline myopathy at 7 months based on muscle biopsy findings and clinical exam
		• Bilateral club feet, requiring casting
		• Myopathic facies, decreased muscle bulk, diffuse hypotonia (axial > appendicular), decreased range of motion and mild finger contractures noted at 4.5 months
		• G-tube placed at 23 months for supplemental feeding
		• No ventilation support is needed
		• Can sit unsupported, but uses a walker to aid in ambulation

DNA samples and medical records from 12 individuals in total were collected under informed consent. Probands and their parents (i.e., trios) were enrolled from Families 1 and 3, and two affected first cousins and their parents were enrolled for Family 2. WES for all 12 participants was performed and donated by Life Technologies (Carlsbad, CA, USA), using standard protocols for the LIFE Library Builder, and sequenced with Exact Call Chemistry on SOLiD 5500xl machines. Both raw reads (XSQ format) and aligned reads (BAM format, generated with LifeScope [22]) were provided.

WGS for ten individuals (excluding an affected male cousin of the Family 2 proband and the cousin’s unaffected mother, for whom sufficient DNA was not available) were donated by Complete Genomics Incorporated (Mountain View, CA, USA) utilizing their standard proprietary protocols and generated using their Standard Pipeline v. 2.0. Variant call files along with aligned reads in Complete’s proprietary format, ‘masterVarBeta’, were provided.

Comprehensive clinical summaries providing clinical and diagnostic data for the presenting complaints and significant secondary findings were prepared by Manton Center staff from the primary medical records and made available on a secure server to the contestants, together with the genomic data described above.

Contestants were solicited from around the world via professional contacts, word of mouth, and an external website [14]. Forty teams applied to participate in the Challenge, 32 of the most experienced multidisciplinary groups were invited to compete, and 30 accepted the offer. Participants – working either independently or as teams – were tasked with working toward an analysis, interpretation, and report suitable for use in a clinical setting.

At the conclusion of the Challenge, 23 teams successfully submitted entries that included descriptive reports of their bioinformatic analytical strategies with rationale, examples of data output and tables of variants, and clinical diagnostic reports for each family. Some groups also provided examples of their patient education materials, informed consent forms, preference setting documents, plans for revisable reporting, and protocols for dealing with incidental findings. Reasons given by four of the seven non-completing teams for dropping out were: technical and management issues, personnel changes within the team, inability to finish on time, or difficulty re-aligning the WES datasets (*N* = 1 each). The other three teams gave no reason.

The 23 completed entries represented a diverse group of approaches and treatments, with some groups focusing almost entirely on bioinformatic issues, others on clinical and ethical considerations. The most compelling entries including a detailed description of the bioinformatic pipelines coupled with clear, concise, and understandable clinical reports. Among the 23 entries, multiple genes were listed as possibly causative for all families (25 for Family 1, 42 for Family 2 and 29 for Family 3). Nevertheless, a consensus was achieved regarding probable pathogenic variants in two of the families. In Family 1, mutations of the titin gene, *TTN* [Online Mendelian Inheritance in Man (OMIM) 188840/603689], recently reported to cause a form of centronuclear myopathy [23], were identified as possibly or likely pathogenic by 8/23 groups, and 6/23 groups reported *GJB2* (OMIM 121011/220290) variants as the likely cause of the hearing loss in the proband. Similarly, 13/23 groups identified and reported a variant in *TRPM4* (OMIM 606936/604559) [24] as likely responsible for the cardiac conduction defects in Family 2. Although no convincing pathogenic variants were identified for Family 3, there were two plausible candidates requiring further study, *OBSCN* and *TTN*, mentioned by six groups each (Table 2).

**Table 2:** Genetic variants

**Family**	**Phenotype**	**Gene**	**Genetic mutation**^**a**^	**Protein change**^**a**^	**Predicted effect**	**Interpretive status**
1	Centronuclear myopathy	*TTN*	c.[35635G > C] + [39893-1G > A]	p.[V11879L] + [spl]	Splice/splice	Likely pathogenic (research result)
	Hearing loss	*GJB2*	c.[101 T > C] + [35delG]	p.[M34T] + [G12Vfs^a^2]	Deleterious missense/frameshift	Clinically confirmed
2	Cardiac conduction defects	*TRPM4*	c.503 T > A	p.V168E	Deleterious missense	Likely pathogenic
3	Nemaline myopathy	*OBSCN*	c.[2245G > T] + [3322 T > A]	p.[G749C] + [Y1108N]	Missense	Uncertain
		*TTN*	c.[84130A > T] + [14492G > A]	p.[K28044X] + [C4831Y]	Missense/nonsense	Uncertain

^a^Reference sequences as follows: *TTN* – NM_001256850.1 and NC_000002.11, *GJB2* – NM_004004.5 and NC_000013.10, *TRPM4* – NM_017636.3 and NC_000019.9, *OBSCN* – NM_001098623.1 and NC_000001.10.

Following the independent review and discussion by the panel of judges, one ‘winner’, the multi-institution team led by Brigham and Woman’s Hospital, Division of Genetics, *et al*. (Boston) was selected, largely on the basis of having a solid pipeline that correctly identified most of the genes judged to be likely pathogenic, as well as for having clear and concise clinical reports that were judged to be best at conveying the complex genetic information in a clinically meaningful and understandable format. Two runners-up were also cited. The first was a combined team from Genomatix (Munich, Germany), CeGaT (Tübingen, Germany) and the University Hospital of Bonn (Bonn, Germany), which had a robust pipeline that correctly identified every relevant gene in clear clinical reports. The second was a team from the Iowa Institute of Human Genetics at the University of Iowa, which had an outstanding array of patient education materials, procedures for patient preference setting and dealing with incidental findings, and policies for transfer of results of uncertain significance to an appropriate research setting if so desired by the patients. The content of the three winning entries is available as Additional files 1, 2 and 3. Five additional teams were cited for ‘honorable mention’ for having pipelines that identified one or more of the likely ‘correct’ genes and for providing clear clinical reporting (Table 3). These eight teams recognized by the judges are defined as ‘finalists’ in the text and for purposes of statistical analysis.

**Table 3:** Challenge participants

**Contest result**	**Contestant**
Winner	The Brigham and Women's Hospital, Multi-Institutional Consortium (Boston, MA, USA)
Runners-up	Genomatix (Munich, Germany), CeGaT (Tübingen, Germany), Institute of Pathology, University Hospital of Bonn (Bonn, Germany)
	Iowa Institute of Human Genetics, University of Iowa (Iowa City, IA, USA)
Finalists	Clinical institute of Medical Genetics, University Medical Centre Ljubljana (Ljubljana, Slovenia)
	Scripps Translational Science Institute (San Diego, CA, USA)
	Science For Life Laboratory (SciLifeLab), Karolinska Institute (Stockholm, Sweden)
	SimulConsult/Geisinger (Chestnut Hill, MA, USA and Danville, PA, USA)
	The Research Institute at Nationwide Children's Hospital (Columbus, OH, USA)
Completed the contest	Tel Aviv University (Tel Aviv, Israel)
	Genome Institute of Singapore, A*STAR (Singapore)
	National Institutes of Health, Regeneron Pharmaceuticals and Stanford University (Bethesda, MD, USA; Tarrytown, NY, USA; Palo Alto, CA, USA)
	Yale School of Public Health, Division of Biostatistics (New Haven, CT, USA)
	River Road Bio/SNPedia (Potomac, MD, USA)
	Pearlgen (Durham, NC, USA)
	Institute for Systems Biology (Seattle, WA, USA)
	Strand Life Sciences (Bangalore, India)
	Sanofi (Cambridge, MA, USA)
	Universidad de Cantabria (Santander, Spain)
	Radboud University Nijmegen Medical Center (Nijmegen, Netherlands)
	Seven Bridges Genomics (Cambridge, MA, USA)
	Omicia Inc/University of Utah (supported by LocusDev Inc (now InVitae)) (Emeryville, CA, USA)
	The University of Texas Health Science Center at Houston, The Brown Foundation Institute of Molecular Medicine (Houston, TX, USA)
	FORGE Canada Consortium (Ottawa, ON, Canada)
Did not complete the contest	BGI (Shenzhen, China)
	British Columbia Cancer Agency (Vancouver, BC, Canada)
	Genedata AG (Basel, Switzerland)
	HudsonAlpha Institute for Biotechnology (Huntsville, AL, USA)
	IRCCS Casa Sollievo della Sofferenza (San Giovanni Rotondo, Foggia, Italy)
	NextBio (Santa Clara, CA, USA)
	The Medical College of Wisconsin (Milwaukee, WI, USA)

### Criterion 1 (pipeline): what methods did each team use to analyze and interpret the genome sequences?

#### Bioinformatic analysis

The particulars of the bioinformatic pipelines, variant annotation and report generation approaches employed by the contestants are summarized in Table 4.

**Table 4:** Pipeline elements and characteristics of successful CLARITY entries

	**Consensus (if any)**	**Finalists**	**Other tools used (% overall)**
**Mapping**			
Read alignment	Used supplied alignments (52%)	Used supplied alignments (63%)	Recomputed alignment data (48%)
Variant detection	GATK and/or SAMtools (75%)	GATK and/or SAMtools (75%)	DNAnexus (5%), FreeBayes (5%), CGI variant table (5%), Avadis NGS (5%), LifeScope (5%)
**Quality control metrics**			
Annotation	Annovar (52%)	Annovar (63%)	Online Mendelian Inheritance in Man (19%), Uniprot (5%), in-house software (5%), SeattleSeq (5%), Variant Tools (10%), KggSeq (5%), SNPedia (5%), ClinVar (5%), PharmGKB (5%), Ingenuity (10%), SG-ADVISER (5%), Human Gene Mutation Database (10%), Genome Trax (5%), dbNSFP (5%), VEP, in-house MapSNPs tool (5%), snpEFF (5%), Genomatix GeneGrid and CeGaT annotation pipeline (5%)
Clinical extraction	Sift and/or Polyphen (90%)	Sift and/or Polyphen (100%)	MutationTaster (10%), LRT Omega, GERP, PhyloP, and FreeBayes (5%)
**Validation**			
Report generation	Filter by relevance to phenotype (71%). Consult with clinician in relevant area (63%). Clinical summary geared towards: non-geneticist clinician (47%), clinical geneticist (29%).	Filter by relevance to phenotype (100%). Consult with clinician in relevant area (100%). Clinical summary geared towards: non-geneticist clinician (38%), clinical geneticist (38%).	

##### *Alignment*

The majority of contestants chose to use the supplied alignments of the data. This is not surprising since the read data from Complete Genomics and SOLiD require special handling due to the nature of sequencing, split reads in the former, and potential for color-space reads in the latter. However, three teams were unable to read the data formats provided and did not submit complete entries.

Alignments were recomputed for the Complete Genomics data by 5 out of 21 teams, with only one team reporting use of the aligner DNAnexus (Palo Alto, CA, USA), while 8 out of 21 teams recomputed alignments for the SOLiD data. For the SOLiD data, five teams recomputed alignments with software aware of color-space, and two teams indicated that they compared their color-space results against a base-space aligner. Reported aligners used for SOLiD data included the LifeScope aligner, BFAST [25], BWA [26-28], Novocraft’s novoalignCS (Selangor, Malaysia) and the Genomatix aligner (Munich, Germany), with some teams utilizing multiple tools for comparison. One team performed error correction prior to alignment for the SOLiD data using LifeScope’s SAET (SOLiD Accuracy Enhancement Tool, Carlsbad, CA, USA).

Prior to variant calling, many teams removed read duplicates using Picard [29] or SAMtools [30], while some teams omitted this step due to the danger of removing non-duplicate reads from single-end data. Using WGS and WES data together gave an additional way to account for PCR duplication. Limited quality control (QC) was performed prior to variant calling, with a single team using BEDTools [31] to analyze coverage QC metrics, and one other team reporting custom mapping QC filters.

##### *Variant calling*

O’Rawe *et al*. suggested that the choice of pipeline might be a significant source of variability in the outcome of NGS analyses [32]. Of the teams, 40% used both the Gene Analysis Toolkit (GATK) [33,34] and SAMtools [30] for variant calling, with the majority using at least one or the other. This indicates that while there is not complete consensus, using GATK, SAMtools or both resulted in acceptable results for the challenge. While GATK and SAMtools are the most popular variant callers used today and reported in this survey, their relative performance has been shown to vary with the sequencing depth [35,36], and direct comparison of variant calls resulting from a parallel analysis of the same raw data by different variant-calling pipelines has revealed remarkably low concordance [32], leading to words of caution in interpreting individual genomes for genomic medicine.

SAMtools was used by some teams to jointly call SNPs and indels while recalibrating quality scores, while other teams used GATK to call SNPs and indels separately. Teams using GATK typically followed the Broad Institute’s best practice guidelines, performing indel realignment prior to indel calling, base quality score recalibration prior to SNP calling, and variant-calling score recalibration after variant calling. Some teams ignored GATK’s base quality score recalibration, mentioning that at the time GATK did not support SOLiD error profiles. LifeScope software containing DiBayes was also used on SOLiD data to call SNPs, and with local realignment to call small indels. In some cases, multiple variant-calling methods were used and compared, with all but one using GATK, SAMtools or some combination thereof. Other tools used with one mention each include: the DNAnexus variant caller, FreeBayes [37] and Avadis NGS (v1.3.1). A number of teams utilized the WGS results from Complete Genomics to look for potentially pathogenic *de novo* copy number variants, but none were found.

A significant source of variation among the different entries was the number of *de novo* mutations reported. Less than five *de novo* mutations per exome, and only about 75 *de novo* mutations per genome, are expected for each trio [38,39], yet some groups reported much higher numbers, recognizing that many of these changes fell within areas with low or poor coverage. Groups that used a family-aware zygosity calling approach, such as the GATK module ‘Phase by Transmission’, developed much more refined lists of only a few potential *de novo* variants per proband, demonstrating the importance of this approach. However, several teams reported problems using the SOLiD data for this analysis as the BAM format provided by SOLiD was different from that expected by GATK, limiting the analysis to Complete Genomics data in those cases.

Variant filtering or recalibration after initial variant calls was performed by 16 out of 20 teams. Six teams used GATK variant quality score recalibration, with other teams reporting use of custom tools. Some teams used BEDTools for coverage QC metrics, but there was no consensus on tools to report sequencing and analysis QC metrics for post-alignment and variant calling.

Teams were asked if they employed any reference datasets in calling variants or comparing datasets to known variants (e.g., batched variant calls, known variant lists, etc.). The most common reference data reported included variants from the 1000 Genomes Project, dbSNP [40], HapMap Project [41], NHLBI Grand Opportunity Exome Sequencing Project (Bethesda, MD, USA), and the GATK Resource Bundle (distributed with GATK). Other reference datasets mentioned were the Mills Indel Gold Standard [42], NCBI ClinVar (Bethesda, MD, USA) as well as public sequencing data produced from the technologies used in this challenge.

##### *Coverage analysis*

One limitation of exome and genome sequencing is that the low/no coverage regions can lead to false positive or false negative results (sometimes 7% to 10% of the exons of the genes of interest have insufficient sequence reads to make a variant call [43]). Only 42% of teams quantified and reported on regions with insufficient coverage or data quality, though 50% of the finalists and two of the top three teams did.

##### *Variant validation*

Many clinical diagnostic protocols still require independent confirmation of NGS results, often by Sanger-based resequencing studies, to validate clinically relevant findings. Although this was not possible in the context of a competition where the contestants did not have access to DNA from the participants, 11 groups took advantage of the independently derived WES and WGS datasets to cross-check and validate their findings. In every instance except two, the teams reported concordance between the variant calls for the *TTN*, *GJB2,* and *TRPM4* mutations that were considered likely pathogenic. The exceptions were both related to calls that were considered false positives in the SOLiD data due to poor quality or coverage at the *GJB2* and *TRPM4* loci, respectively. The *GJB2* findings had previously been clinically confirmed and the contest organizers subsequently arranged for independent research and clinical testing, which confirmed the *TTN* and *TRPM4* variants as well.

#### Medical interpretation of variant lists

The most frequent methods used to annotate variants reported were Annovar [44] (52%), in-house developed software (17%), and Ingenuity (Redwood City, CA, USA) (12%). Other tools reported were Variant Tools [45], KggSeq [46], SG-ADVISER (Scripps Genome Annotation and Distributed Variant Interpretation Server, La Jolla, CA, USA), Genome Trax (Wolfenbüttel, Germany), VAAST (Variant Annotation and Search Tool) [47], Omicia Opal [48], MapSNPs [49], in-house pipelines, and combinations thereof. There were a large variety of annotation sources (see Table 4), including but not limited to: OMIM [50], Uniprot [51], SeattleSeq [52], SNPedia [53], NCBI ClinVar, PharmGKB [54], Human Gene Mutation Database [55], dbNSFP [56], and in-house annotations. More importantly, most teams (14/20, 70%) performed their own curation of annotations, for example, by performing a medical literature review or by checking for errors in externally accessed databases. Thus, a manual review of annotations was deemed necessary by most contestants. Many teams considered the family pedigree structure as an important input for evaluating variants, as this allowed identification of potential *de novo* mutations, filtering for dominant inheritance in Family 2, ensuring Mendelian segregation and carrier status in parents for recessive mutations, etc. The function was largely performed manually, but use of automated tools such as the GATK module ‘Phase by Transmission’ was considered by some groups although the underlying structure of the SOLiD data led to problems with the analysis.

Reasons given for why teams did not report each of the likely pathogenic variants in Families 1 and 2 varied by gene and by team, but in many instances, were due to decisions made during the medical interpretation phase of analysis. Of the 15 teams that did not report the *TTN* variants for whom survey data were available, the variant calls generated by three failed to identify them. Twelve groups reported that their variant callers identified the two variants, but in six of these, automatic filters eliminated the gene from further consideration because the frequency of potentially pathogenic variants in this enormous gene was considered too high to be credible as a likely disease gene. Of the six instances where the automated pipelines reported the variants as potentially pathogenic, five were subsequently manually eliminated from further consideration because medical consultants lacked the clinical expertise or did not believe the published association with cardio- or skeletal-myopathy because of the high frequency of missense changes in the normal population. Notably, in none of the exclusions based on the high degree of heterogeneity of the gene was a distinction made between predicted truncating mutations, which are much rarer, versus more common missense changes. In one instance, a simple programming error prevented *TTN* from rising to the top of the candidate gene list in an automated expert system, and subsequent correction of this mistake resulted in a correct call of likely pathogenicity for the *TTN* variants in Family 1.

Seventeen teams reported not flagging the *GJB2* mutations as likely causative for hearing loss in the proband of Family 1. Remarkably, the variant callers employed by ten teams failed to identify these changes despite the fact that seven of these teams used either GATK and/or SAMtools. Among the remaining seven teams, two ignored the findings because they were considered irrelevant to the ‘primary phenotype’ of skeletal myopathy and two reported a lack of clinical expertise necessary to recognize that hearing loss was a distinct phenotype. The remaining three teams reported that one of the previously published known pathogenic variants was automatically filtered out due to its high minor allele frequency in normal populations.

The *TRPM4* variant in Family 2 was clinically reported by 13 of the 23 teams. Only two teams cited failure of their variant callers to identify this mutation, but five more reported that the variant was discarded due to poor quality data (low depth and noisy location with multiple non-reference alleles at that location in the SOLiD data) in one of more of the individuals, which led to inconsistent calls among the different affected family members. Two groups failed to recognize the likely pathogenicity of this variant; one reported it as a variant of unknown significance while the last one’s computational genetic predictive scoring simply failed to weight this gene highly enough to pass the cutoff given their entered phenotypic parameters. The remaining group identified the *TRPM4* variant, but strongly favored another variant in the *NOS3* gene as a better explanation for the structural heart defects.

##### *Pathogenicity prediction of missense variants*

The most common tools to tackle the problem of determining the effect of amino acid substitutions on protein function for missense mutations were SIFT [57] and Polyphen [49]. While 80% of teams used both SIFT and Polyphen to predict pathogenicity, there was no significant difference in the success of the teams using both SIFT and Polyphen and those who used one or the other or some other tool entirely. Other tools listed by teams were PhyloP [58], likelihood ratio test scores (LRT) [59], MutationTaster [60], GERP [61], and in-house developed tools. Also of note: 45% of teams attempted to assess the statistical confidence of assignment of pathogenicity (63% of finalists). Methods named included custom in-house methods (*N* = 3), considering gene size (*N* = 2), utilizing known predictions of pathogenicity (*N* = 3) and allele frequencies (*N* = 2), assessing commonly mutated segments (*N* = 2), and using true positive and neutral datasets within a Bayesian framework (*N* = 1).

Use of splice prediction tools is particularly important, as approximately 14% to 15% of all hereditary disease alleles are annotated as splicing mutations [55]. Groups that utilized a suite of splice prediction tools, such as the maximum entropy model MAXENT [62], ExonScan [63] or positional distribution analysis [64,65], were more likely to have identified potentially pathogenic mutations, particularly in the *TTN* gene in Family 1.

It was well recognized by all groups that allele frequency is an important consideration in assessing pathogenicity (though specific cutoffs were not mentioned). All groups also agreed that conservation of amino acid sequence across species is useful for interpretation of missense variants. Half of the teams (63% of finalists) took advantage of the whole genomic sequences to analyze non-coding variants, but none of the teams reported potential pathogenic changes in deep intronic or intergenic regions, even for Family 3, likely largely due to the undefined and uncertain status of such variants. Of teams that reported methods for predicting pathogenicity of non-coding variants, the most frequently used methods were splicing prediction algorithms (85%) and transcription factor binding site prediction (46%), with 23% also considering changes in known promoter/enhancer elements, and one team each assessing evolutionary conservation, DNase hypersensitivity sites and microRNA-binding sites.

##### *Medical interpretation and correlation of pathogenic variants with the clinical presentations*

Almost all entrants performed a clinical correlation at the level of a single general diagnosis such as ‘myopathy’, ‘centronuclear myopathy’ or ‘nemaline myopathy’ with a list of predetermined candidate genes. From a clinical perspective, this reduces clinical diagnostic decision support to a list or panel and counts on that subset being complete for maximum sensitivity. However, in the case of Family 1, for example, the likely pathogenic gene was not generally recognized as causative for centronuclear myopathy at the time of the contest. In contrast, one entrant used clinically driven diagnostic decision support [66] in which the clinical analysis was carried out based on a description of the patient’s various pertinent positive and pertinent negative findings, including their age of onset. This was then paired to the genome analysis in a way that used a novel pertinence calculation to find the one or more genes among those with described phenotypes that best explains the set of pertinent positive and negative findings [66]. As they become refined and validated, such automated approaches will become a critical aid in the future for reducing the analysis times to a manageable level necessary to support the higher throughputs required in a clinical diagnostic setting. Indeed, the reported range of person-hours per case required for medical interpretation of each case was 1 to 50 hours, with the automated approach requiring less than 4 hours on average to complete.

#### Attitudes and remarks

Three teams were unable to read the data formats provided and did not submit complete applications. This likely reflects the unique nature and format of SOLiD and Complete Genomics data and suggests that greater adoption of standard formats (FASTQ, SAM/BAM and VCF) for bioinformatics tools is required.

We observed that finalists were significantly more likely to express a preference for generating their own sequencing data instead of having it generated by an external sequencing provider (75% versus 27%, *P* = 0.041). The main reason expressed for in-house data generation was control over the sequencing process to ensure production and assessment of high quality data. Other reasons expressed included cost, turnaround time, and ability for reanalysis. This preference may also reflect a tendency for the most experienced groups to have a legacy capacity to generate sequence data, and thus a bias towards using their own capacity. However, it also raises the reasonable possibility that integrated control of the process from sequence generation through variant calling is important for producing the highest quality variant calls.

Overall, the teams when asked for reasons for their preference in their preferred sequencing technology mentioned accuracy and standardized software tools, highlighting the need for standard methods and tools for primary bioinformatics analysis. Furthermore, the majority of teams (13/18) felt that NGS should be combined with classical techniques (e.g. Sanger sequencing and PCR methods) for confirmatory testing in clinical situations. However, a few recognized that with increasing depth of coverage and accuracy of alignment, NGS, particularly of less complex libraries such as gene panels and possibly exomes, had potential to be utilized as a stand-alone test once QC studies demonstrate sufficient concordance with traditional methods.

Interestingly, all four of the finalists that did not report low-coverage or uncallable regions reported that they were going to begin doing so, whereas one of the non-finalists mentioned that they were going to add coverage quality to their reports. Regions in which sequencing technology or reference-genome-specific difficulties exist are important considerations for accurate variant detection. Moreover, it is critical to provide locations in which variant calling is not possible due to lapses in coverage.

Teams had different opinions on the level of coverage they felt was necessary for accurate variant calling from NGS of whole genomes. The finalists reported that they felt a higher level of coverage was necessary (59× average) than the rest of the teams (38× average). Similarly, the finalists differed on the coverage required for whole exomes (74× versus 49×) or gene panels (121× versus 69×).

A large majority of the teams used SIFT and Polyphen to predict the pathogenicity of a variant, which is a sound strategy given the programs do not always agree in protein predictions, and in both, specificity is reported to be high but sensitivity low [67].

When asked about their process used to validate pathogenicity predictions, 58% of teams reported that they did not use any validation method, or did not have any datasets to compare estimates against. The finalists were more likely to have had in-house datasets to work against, which may be due to differences in analytical resources that could be devoted to this problem. Overall, this process was reported as manual for the majority of the teams.

The diversity of approaches to preparing the contest entries made direct comparisons of methods difficult, so the post-contest survey was designed to elicit a more homogeneous dataset. Nevertheless, several contestants neglected to respond to some of the questions, and the responses to others was variable, indicating some confusion on the part of respondents regarding the intent of the query.

### Criterion 2: were the methods used efficient, scalable and replicable?

There are still some manual elements to many pipelines that inhibit scalability. For an average case, teams reported that the interpretation process ranges from 1 to 50 hours (mean 15 ± 16 hours). For the CLARITY challenge, the time spent was much greater: each case took from 1 to 200 hours (mean 63 ± 59 hours). The average CPU time required for the analyses was difficult to estimate as contestants utilized different approaches, and not every entry was normalized for the number of parallel processors, but contestants reported utilizing 306 ± 965 CPU hours per case (range 6 to 8,700 hours). Reported costs to run the pipeline also varied considerably ranging from USD 100 to USD 16,000 (average USD 3,754 ± 4,589), but some contestants were unable to calculate salary costs leading to some lower estimates. Although costs have fallen dramatically, and computational resources are becoming increasingly available, the requirement for manual curation and interpretation of variant lists remains a considerable barrier to scalability, which could inhibit widespread use of NGS exome and genome diagnostics in the clinic if well-validated and substantially automated annotation tools do not emerge.

### Criterion 3: was the interpretive report produced from genomic sequencing understandable and clinically useful?

#### Consent and return of results

When asked about their approach to consenting and return of results in the survey, teams’ responses varied considerably. The question was irrelevant for a number of contestants (9/21) whose activities were restricted to research or contract sequencing without direct patient contact. Finalists were more likely to ask patients undergoing WES/WGS to sign a specific consent form or provide specific explanatory materials for the methodology (*P* = 0.057). Finalists were much more likely to detail how they were going to handle incidental (i.e., unanticipated) results (*P* = 0.002). However, only 35% of teams reported that their consent materials include an option for patients to express their preferences around the return of incidental results. Most teams (76%) reported that they did not provide examples of consent and/or explanatory materials for patients with their CLARITY submissions, and since patient interaction was not allowed for the challenge, a number of contestants simply considered the issue moot. However, upon reflection, many teams agreed that including consent and explanatory materials would have strengthened their entries.

Overall, it is notable that most teams’ submissions did not include specific consent and explanatory materials, did not detail a predetermined approach for handling incidental results, and did not describe any options for patient preferences. In some cases, survey responses indicated that such materials and plans are used in practice but were not included in the CLARITY Challenge submission because it was not clear that such content was in the scope of the challenge. In other cases, teams reported that they have not developed these materials and plans or they do not routinely focus on this aspect of the process. These findings highlight the fact that these components, though they are essential for the patient-facing implementation of clinical sequencing, are not consistently prioritized or highlighted by many groups involved in the clinical use of NGS.

#### Reporting methods

**Figure 1:**
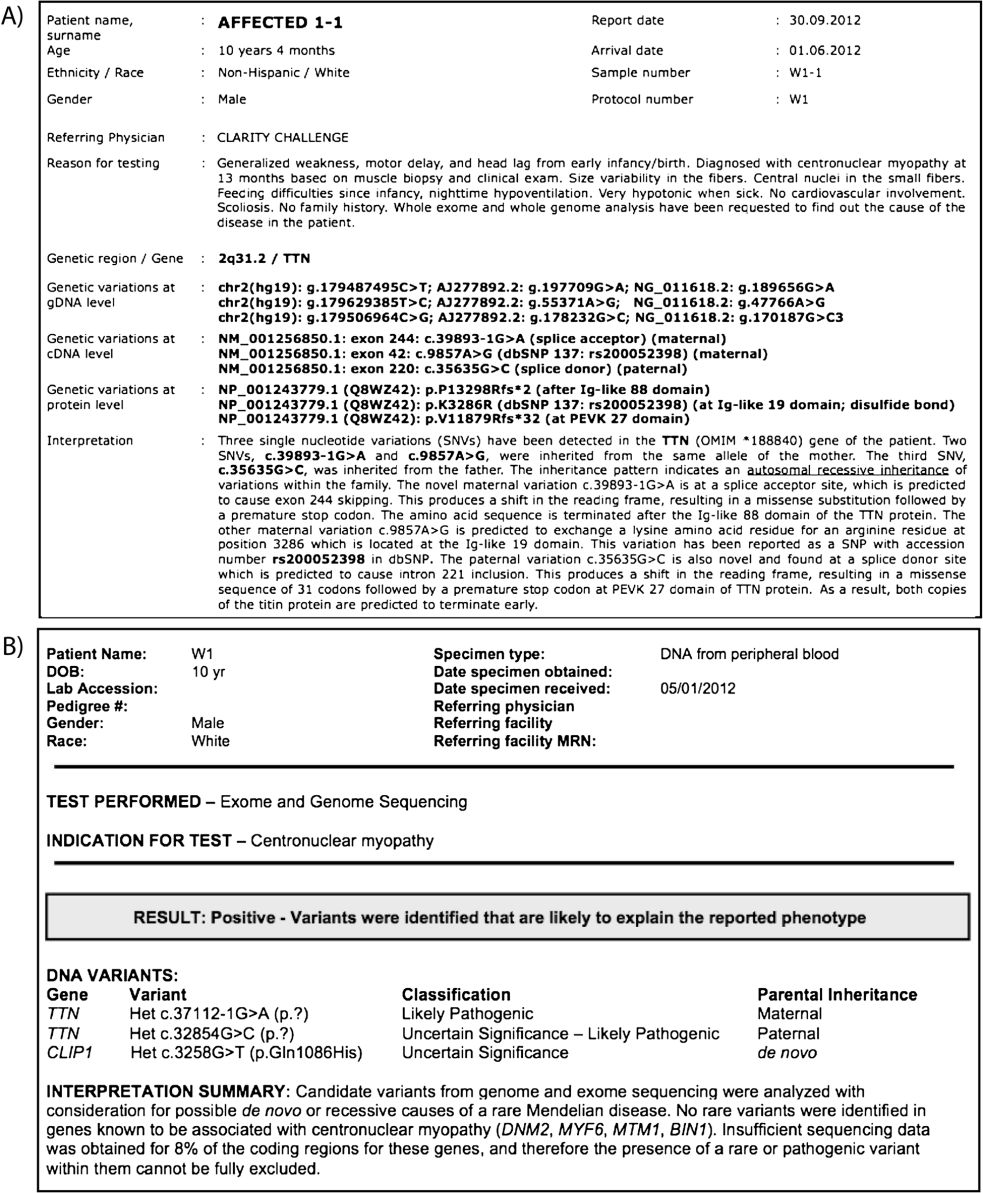
**Representative clinical report from two of the finalist teams (A and B).** Desirable elements include subject demographics, indication for testing, use of HUGO-approved gene symbols, specification of the relevant variants at the genomic DNA, cDNA and protein levels including reference sequences and dbSNP identifiers, description of zygosity, estimation of insufficient coverage for candidate genes, and succinct clinical interpretation and interpretative summary. Note the use of different reference sequences, and the lack of specification in **(B)** makes direct correlation between reports difficult.

Reporting methods were not uniform amongst teams. Reporting the accession number for cDNA reference sequences was significantly more frequent in finalists than in non-finalists (87% versus 22%, *P* = 0.009). However, teams did converge on some items: reporting zygosity was standard, with 88% of responding teams doing so. Reporting the genome build was also specified by 72%. That said, the genome build reporting was problematic even among the winning teams; two of the finalists submitted elegant reports, clearly stating the variants found, summarizing the location, the classification and the parental inheritance, with a short interpretation (Figure 1). However, the accession numbers reported were different: a different build was used in each report and not specified, so it would take considerable effort to discern whether the two reports were truly referring to the same variants.

#### Clinical reports

Finalists were more likely to present a clinical summary report with their entry, with the trend approaching significance (100% versus 69%, *P* = 0.089). Perhaps in response to recently published guidelines [68], there was striking concordance in interpretation and reporting philosophy, with all finalist and most non-finalist teams gearing their reports towards a clinical geneticist, genetic counselor or non-geneticist clinician. Almost all teams agreed that a non-geneticist clinician should be the target audience of clinical summary reports (75% of finalists and 89% of non-finalists). Finalists were more likely to feel that their clinical summary report could be used in clinical care (100% versus 67%, *P* = 0.08), though there was overall agreement that it was important that NGS studies produce a clinical summary report that can be implemented in the clinic (95% ranked this as ‘important’ or ‘extremely important’). Most of the teams (80%) filtered their variant list by relevance to phenotype, with more successful teams more likely to do so (*P* = 0.074). All teams but one finalist (95%) agreed that filtering the variant list by relevance to phenotype is an appropriate method for communicating information to clinicians.

It is still not commonplace to consult with an expert physician during report preparation, but doing so clearly correlated with success. Only 61% of teams routinely consult with a medical doctor in a relevant disease area. Finalists were significantly more likely to involve clinicians on a regular basis (100% versus 36%, *P* = 0.001). Perhaps related, in their reports prior to the survey, all but one of the finalists considered the hearing loss to be a separate phenotype from the myopathy in Family 1, while only 36% of the less successful teams did (*P* = 0.059). Of those who considered the separate phenotype, 75% of finalists and 63% of non-finalists considered its genetic basis.

## Conclusions

### Overall convergence and agreement across the finalists

Overall concordance among the teams in the development of variant lists was remarkable given the dozens of available measurement and analytical components of NGS pipelines and the hundreds of thousands of variants harbored by the genomes of the families. Despite the many paths that could be taken, the finalists utilized much the same philosophy and tools in processing the data and generating variant calls, and there were often minimal differences between finalist and non-finalist teams in the large lists of potentially pathogenic variants. A caveat of our study design was the choice of sequencing technologies, as Illumina platforms now account for a greater proportion of clinical studies than either SOLiD or Complete Genomics-based studies. Eight groups analyzed only the SOLiD WES data and four restricted their analysis to the Complete Genomics WGS data, often because of real or perceived difficulties with converting the extensible sequence format from the SOLiD runs into generic FASTQ files that would run on BWA, or unfamiliarity with the proprietary Complete Genomics data formats. However, as many aspects of the analytical pipelines, including variant calling and annotation, pathogenicity prediction, medical interpretation and reporting methods, are platform independent, most results discussed here should be generally applicable even as sequencing technology continues to evolve.

A number of teams preferred to recompute alignments, even though vendor alignment data was supplied, showing a preference for control over the analysis process and methods, and to ensure high quality results. Furthermore, a subset of teams for the same reasons expressed a preference for generating sequencing data in-house with higher coverage.

The selection of bioinformatic tools used by the teams did not appear to differ greatly. Tools for variant calling centered on GATK and/or SAMtools. Of the teams, 80% performed variant filtering or recalibration after initial calls were made. It is difficult to evaluate the need for recomputing alignment, performing indel realignment, variant filtering, or recalibration, given the small number of samples in this exercise. Fewer teams reported regions with insufficient coverage or data quality, only 42% overall. Without this information, it is impossible to evaluate the sensitivity of any NGS-based testing, making this an area requiring further development throughout the field.

Use of reference datasets (1000 Genomes, dbSNP, HapMap, NHLBI Go ESP and OMNI), and annotation databases (OMIM, Uniprot, SeattleSeq, SNPedia, ClinVar, PharmGKB, Human Gene Mutation Database, dbNSFP and in-house annotations) revealed considerable consensus and uniformity across entries. This shows the preference for a wide variety of rich data sources to maximize power to understand how to prioritize and contextualize variants in the presence of known information. Annovar was the most common annotation tool, with Ingenuity also used frequently. SIFT and Polyphen were overwhelmingly used to predict pathogenicity of missense changes.

Supplementary analyses that were more likely to be employed by successful teams included consideration of allele frequency, conservation of amino acid sequence across species (for coding variants), use of splicing prediction algorithms, and assessment of transcription factor binding sites (non-coding variants). Finalists were more likely to have in-house datasets to validate pathogenicity estimates. The use of in-house datasets to serve as validation sets for estimates of pathogenicity shows the need for a large, publicly available database for this purpose.

Methods and results diverged more widely in the medical interpretation of the variant lists and correlation of variants with the clinical presentations and the medical literature. Nearly half of the teams rated their process to determine pathogenicity as ‘manual’, while the mean time per case was over 10 hours, underscoring the need for standardized automated processes. Some teams have made progress towards automating this process – e.g., Genomatix’s automated literature search tool; LitInspector [69] was noted by judges and other teams alike as being best in class. Some teams mentioned a desire to utilize such methods in their own pipelines. SimulConsult was able to determine most variants with minimal manual effort and less hours per case than average, providing a tremendous potential advantage in high throughput clinical environments. The ability to automate the genome–phenome correlations is a key capability that can make the difference between an analysis that can become part of clinical care and an analysis that is only practical in a research setting of gene discovery.

### Patient choice

Questions of patient preference and the responsibilities of laboratories to return incidental findings are a controversial and rapidly evolving area [70]. The team from Iowa highlighted the importance of patient preferences in defining the style of their reports. This represents an open challenge to the medical community to decide whether future reports should take into account patient preferences or defer to a more paternalistic model of clinically indicated disclosure. In terms of clinical reports and return of results, finalists were more likely to have consent or explanatory materials, and have a plan for incidental result return. Regardless, upon being surveyed, there was general agreement amongst all teams that clinical reports should be geared towards a clinical geneticist, genetic counselor, or non-geneticist clinician.

### Variability of detection power

The fact that only two teams identified all the likely causative mutations, despite using generally similar approaches, demonstrates the need for consistency and rigor in approaches to variant interpretation. There is room for tuning the tradeoffs in sensitivity, specificity and number of etiologic hypotheses being tested that would benefit many teams performing NGS interpretation. Currently, there is little consensus on the thresholds used by various teams to determine pathogenicity of potential disease-causing variants. In some cases contestants explicitly excluded variants as potentially causative due to the belief that they were likely sequencing or variant-calling false positives or benign variants that, although occurring naturally, are not disease causing or not solely disease causing. Several groups, for example, noted that in Family 3 the proband carries multiple variants in the *OBSCN* gene, and that any diagnosis based upon variants in this gene must therefore be viewed cautiously.

The titin gene, *TTN,* presented a similar dilemma as multiple potentially pathogenic variants were detected in both Families 1 and 3. Nevertheless, successful teams recognized the probable causative nature of the *TTN* variants in Family 1 based on the fact that one was a published pathogenic change previously reported to cause dilated cardiomyopathy [71] and the second mutation was predicted to alter splicing. The winning team also cited a conference abstract, then available on the web [72] and now published [23], describing a parallel study of a cohort of patients with centronuclear myopathy with validated mutations in the *TTN* gene. Thus, the ability to correlate genomic results with emerging literature, almost in real time, provided the determining factor between making the correct call or not, and highlights the potential power of retrospectively revising reports as new research results become available: i.e., the concept of ‘revisibility’.

The two *GJB2* gene variants identified as causative for sensorineural hearing loss for the proband in Family 1 had been clinically confirmed prior to the contest, but were not disclosed to the participants, and therefore served as a validated disease-causing variant set. Six groups identified and reported these mutations as likely responsible for the sensorineural hearing loss. The way teams dealt with the reported hearing loss in Family 1 is illustrative of variation in their understanding of the clinical phenotypes, as well as their views on reporting incidental findings. Two groups considered that the defect was likely part of the myopathic phenotype, while seven others considered the *GJB2* mutations to be incidental, and hence did not look for or report them, because, even though the audiometry results were detailed in the clinical records, the hearing deficit was not listed as part of the primary diagnosis.

### Pre-test differential diagnosis is needed

Fourteen of 19 teams reported having a medical geneticist on board and another included a physician partner, but four teams among the non-finalists did not have a medical expert. The fact that many teams did not appreciate the significance of *GJB2* mutations for Patient 1 suggests that additional detailed input from medical experts reviewing the clinical data would have been beneficial, highlighting the need to have a clinician with genetics expertise involved in preparing a carefully considered pre-test differential diagnosis.

### Emergence of standard of care

Implied by the convergent methods across the leading contestants is that there is a *de facto* consensus of experts for interpretation of NGS. This represents a signal opportunity to codify and make this consensus explicit to ensure the greater safety and accelerated commoditization of NGS. Aspects that still need attention and further development before becoming part of the standard of care include robust family-aware zygosity calling, coverage estimation and reporting, splice site prediction and analysis, and automation of genome–phenome interpretation.

While there has been rapid progress in the development and characterization of each of the individual components of the analysis, interpretation, and reporting pipeline, there is not yet a set of best practices that can be applied to the entire ‘end-to-end’ process of genomic measurement and interpretation. Genomic medicine will require such consensus and standardization to achieve widespread, routine, and reliable clinical use. While, eventually, organizations such as the American College of Medical Genetics and the College of American Pathologists will promulgate standards to be used in the management and accreditation of laboratories, it was the intention of the CLARITY challenge to help identify the emerging forerunners of such standards, and accelerate their development. The general feedback among contestants has been very positive and the stimulus for these groups and the entire industry to generate more and better tools and reports for molecular diagnosis has truly been achieved, also clearly documented by the number of participants.

In summary, the contest highlighted: a) the relative uniformity of methods employed for alignment, variant calling, and pathogenicity prediction; b) the need to continue developing publicly available reference genome databases; c) the need for more attention to coverage analysis and estimation of false negative rates for candidate genes; d) the need for greater attention to the development of clear, concise clinical reports, with common elements such as use of reference accession numbers and genome builds, consistent criteria for definition of pathogenicity (or degree of uncertainty); e) the value of input from medical experts who could correlate the reported phenotypic elements with the expanding literature on genes and gene function; and f) the importance of clinical genetics expertise in identifying candidate families for testing. Given the labor-intensive nature of variant analysis and clinical report generation, attention to automated genome–phenome analysis based on methods for literature mining and curation, as well as variant assessment, is a pressing need that will improve reproducibility and scalability of genomic-level analyses in the future.

## Materials and methods

### Subject recruitment and informed consent

Probands with rare medical conditions of apparent, but unknown, genetic etiology were identified through the Manton Center for Orphan Disease Research and their families were approached about participation in the contest. Every subject who provided clinical information and DNA specimens for analysis first provided informed consent through Protocol IRB-P00000167 under the supervision of the Boston Children’s Hospital IRB. Under the terms of this protocol, the distribution of the complete genome and exome sequences was restricted to contest organizers and qualified contestants, who all signed legal agreements to protect the privacy of the participants and pledges to return or destroy the sequences at the conclusion of the contest. Because of the risk of detection of incidental findings not related to the specific medical conditions identified in the clinical descriptions, and the fact that some participants might be publicly identified through publicity related to the Challenge, the IRB precluded any possibility of public dissemination of the raw genomic sequences. All clinical and molecular datasets were de-identified prior to distribution to the contestants, and any identifiers included in the contest entries and additional files are pseudonyms or codes with no relationships to the participants’ actual protected health information as defined by the HIPAA Privacy Rule of the US Department of Health and Human Services [73].

### Contest judging

Contest entries were evaluated by an independent group of six judges not affiliated with the contest organizers (ISK, AHB and DMM). Judges represented a diverse array of disciplines, including computer science and bioinformatics (PN, DM Jr and PS), medical/human genetics (J Majzoub and HFW), and clinical diagnostics (EL). Judges were asked to evaluate all aspects of the entries, but to pay particular attention to their areas of expertise. Final selection of winners was achieved by consensus among the six independent judges and was largely based on evaluation of three main criteria:

1. What methods did each team use to analyze and interpret the genome sequences?

2. Were the methods used efficient, scalable and replicable?

3. Was the interpretive report produced from genomic sequencing understandable and clinically useful?

Although identification of the ‘correct’ likely causative mutations for each family was considered, this was not an overriding factor, especially in light of the fact that the mutations for each family were not previously known and in some cases the results remain uncertain and fall into the realm of ongoing research. As it was, multiple genes were listed as possibly causative for all families (25 for Family 1, 42 for Family 2 and 29 for Family 3).

### Post-contest data collection and analysis

After the finalists and winners were declared, all teams were sent a packet including a structured survey of contestants’ methods and practices and copies of the winning three teams’ entries. The purpose of the survey was to provide uniformity in data for summarization and allow for self-assessment of each team’s entries relative to the winning entries. Of 23 groups that submitted contest entries, 21 (91%) returned the survey. A follow-up survey in response to reviewers’ suggestions resulted in a 100% response rate for the 23 contestants. The complete set of survey questions and aggregate responses are provided as Additional file 4. Statistical analyses were performed using the computing environment R [74] and all reported *P* values are from unpaired *t*-tests.

## Abbreviations

CLARITY: Children’s Leadership Award for the Reliable Interpretation and Appropriate Transmission of Your Genomic Information; GATK: Gene Analysis Toolkit; IRB: Institutional Review Board; NGS: next generation sequencing; OMIM: Online Mendelian Inheritance in Man; PCR: polymerase chain reaction; QC: quality control; SNP: single nucleotide polymorphism; WES: whole exome sequencing; WGS: whole genome sequencing.

## Competing interests

BM is an employee of, and holds an equity stake in, Life Technologies, Inc. EL is an employee of the University of Utah, with an assignment as medical director at ARUP Laboratories and receives consulting fees from Complete Genomics. DM Jr is an employee of, and holds an equity stake in, Cerner Corp. HD, BHF, MSL, IL, HMM and HLR are employees of the Laboratory for Molecular Medicine of the Partners Healthcare Center for Personalized Genetic Medicine. HLR sits on advisory boards for BioBase, Clinical Future, Complete Genomics, GenomeQuest, Ingenuity, Knome and Omicia. BHF holds an equity stake in InVitae Corporation. C Gugenmus, A Hahn and BK are or were employees of Genomatix GmbH. MS, J Supper and M Menzel are employees of CeGaT GmbH. SB is an employee of, and holds an equity stake in, CeGaT GmbH. PF is an employee of the Children’s Hospital Reutlingen and is on the scientific advisory board of CeGaT GmbH. SP is a member of the scientific advisory board of CeGaT GmbH, Tübingen, Germany. MMS is an employee of and holds an equity stake in SimulConsult. AT holds an equity stake in Cypher Genomics, Inc. WZ was an employee of Sanofi SA. LZ, K Blair and DK are employees of, and hold equity stakes in, Seven Bridges Genomics, Inc. M Cariaso and GGL are principals of River Road Bio. PCN holds an equity stake in Illumina, Inc. DRR is an employee of Ingenuity Systems. YB and YH are employees of, and hold equity stakes in, Regeneron Pharmaceuticals, Inc. MGR and EK are employees of, and hold an equity stake in, Omicia. M Cargil, RKH and JMS are employees of, and hold equity stakes in, InVitae Corporation. ZIA is an employee of, and holds an equity stake in, Novocraft Technologies Sdn Bhd. The remaining authors have not declared any competing interests.

## Authors’ contributions

ISK, AHB and DMM conceived of, organized and ran the Challenge. CAB conducted and analyzed the post-challenge survey. NH, BM, TWY and LJL analyzed bioinformatic approaches. KCF coordinated the Challenge and collected data. ETD, MCT and AHB ascertained and recruited the subject families. CAB, MCT, SKS and ENP analyzed contest entries. IAH analyzed aspects of the entries regarding reporting of consent and return of results. EL, J Majzoub, PN, DM Jr, PS and HFW judged the Challenge and reported on the entries. NJM, RT, RSF, SWY and LM referred the families and provided and analyzed clinical data. SRS, IA, CACassa, PIWdB, HD, WF, LF, BHF, MAG, REH, KL, MSL, ML, IL, DGM, HMM, MFM, PPP, SR, HLR, R Soemedi, NOS, SV, J Supper, C Gugenmus, BK, AHahn, MS, M Menzel, SB, PF, MD, MB, SP, RJHS, JLA, JH, KR, VCS, EMS, TB, EAB-Z, TAB, BD, APD, DLK, TES, AES, R Sompallae, KW, AGB, EE, KM, SAM, OAS, PT, AB, CACampbell, JWH, AK, TM, KP, TW, DVD, HA, K Booth, NM, MMS, MSW, GT, PW, DC, SFB, GH, DLT, KLM, D Newsom, CRP, ATR, AM, LL, AP, BP, AT, AW, MH, AA, JML, M Magnusson, D Nilsson, HS, FT, C Gilissen, AHoischen, BVB, HY, MN, WZ, J Sager, LZ, K Blair, DK, MCariaso, GGL, AJ, SA, PCN, KSS, SK, VV, OI, EH, EF, NS, GG, JCR, JC, HCC, DM Jr, SAA, LR, DRR, FASL, MLGG, CTC, YB, YH, FF, YZ, ZW, JB, JMGL, DGL, JL, MCR, IV, MGR, FMDLV, EK, MCargill, RKH, JMS, GJL, DAS, BEB, BMM, KE, MY, HZ, LH, XC, XY, MChen, CL, CY, MG, PL, YK, ACA, ZIA, KMB, DEB, PMKG, AMI, BMK, J Majewski, CRM, JSP, SLS, MES and J Schwartzentruber were contestants who participated in the Challenge, reported on their standard practices, and contributed comments and material for the manuscript. CAB and AHB drafted the manuscript. NH, SKS, IAH, EL, J Majzoub, PN, DM Jr, PS, HFW, NJM, ISK and DMM contributed to drafting the manuscript. All authors read and approved the final manuscript.

## Supplementary Material

Additional file 1 The complete entry from the Brigham and Woman’s Team containing seven PDF files, six PNG image files, and one XLS table. Click here for file

Additional file 2 The entry from the Genomatix/CeGaT/University Hospital of Bonn team containing five PDF files and six XLS tables. Click here for file

Additional file 3 The entry from the University of Iowa. Click here for file

Additional file 4 **Individual and aggregated results from questions in the structured surveys of contestants’ practices.** Responses are broken down into separate sheets according to category as follows: PART A: Consenting and explanatory materials for whole exome/genome sequencing technology. PART B: About your summary clinical report. PART C: Interpretive reports. PART D: Revisible reporting. PART E: Variant identification. PART F: Data analysis. PART G: Validation of analytical tools. PART H: Methods predicting variant pathogenicity. PART I: From variants to phenotype. PART J: Overall impressions and team composition. PART K: Follow-up questions, costs and sensitivity. Click here for file
