# Supplementary material for: An international effort towards developing standards for best practices in analysis, interpretation and reporting of clinical genome sequencing results in the CLARITY Challenge
Source: Genome Biol. 2014 Mar 25;15(3):R53. doi: 10.1186/gb-2014-15-3-r53 (PMC4073084; doi:10.1186/gb-2014-15-3-r53)

SUMMARY OF THE SCIENTIFIC REPORT

General Approach

Our analysis pipeline consisted of an initial upstream analysis, focused on sequence assembly and annotation, a higher level downstream analysis, focused on prioritizing variants, and an interpretative analysis by our clinical genetics subteam (**Fig. 1**).

Review of the three Clarity clinical cases suggested potential inheritance modes for each case. From this starting point, for each case we identified single nucleotide variants (SNVs) and copy number variants (CNVs). We called SNVs using a pipeline that we originally developed to interpret short sequence reads from HiSeq (Illumina) data, that we generalized to accommodate reads from generic sequencing platforms. Following extensive quality control (see **Supplement**), we defined a consensus call set, based on both exome sequencing data (SOLiD platform) and whole genome sequencing data (CGI platform), for downstream analysis. We called CNVs solely on the CGI whole genome sequencing data. In addition, we estimated the probability to miss causal variants due to undercalling. Analysis of raw BAM files did not reveal potential causal variants in sufficiently covered regions of known genes.

We applied principal components analysis (PCA) to assess individual ancestry, in order to assess the background allele frequencies in an appropriate ancestry-matched population. We observed that all patients were of European descent.

We assigned functional annotations to SNVs and CNVs using both VEP software and MapSNPs (in-house annotation tool), accounting for the possibility that multiple gene transcripts might result in different functional annotations. We identified confidently called *de novo* mutations and rare SNVs (<0.1% frequency in ESP) that were consistent with the proposed inheritance mode for each pedigree. All CNVs were computationally genotyped in the 1000 Genomes dataset to exclude common CNVs from the downstream analysis.

The upstream analysis was highly efficient and identified only a small number of candidate variants. These candidates were further prioritized using the following criteria listed in the order of importance. **First**, we assessed the potential phenotype of variants based on known phenotypes of candidate genes in humans and model organisms. Our confidence in these candidates was reduced if we observed that known phenotypes were inconsistent with patient phenotypes. **Second**, we assessed the potential molecular function and cellular localization of candidate genes based on the literature, mRNA expression data, and data on protein-protein interactions. **Third**, we evaluated the potential for candidate variants to have a substantial effect on molecular function based on computational analysis and manual inspection of amino acid sequence and comparative genomics data. We commenced minigene assays to evaluate variants with potential effect on splicing; these assays are ongoing. **Fourth**, we evaluated the potential for predicted candidate variants to represent technical artifact with a context-dependent model for *de novo* mutations and estimates rates of compound heterozygote and homozygote individuals for specific genes in the general population. **Collectively, these analyses resulted in a <u>single</u> candidate for W3 (*OBSCN*), a single candidate for the conduction defect in W2 (*TRPM4*), two ranked candidates for W1 (*TTN* and *CLIP1*) and a short ranked list of potential candidates for other cardiac phenotypes in W2.**

These results were communicated and discussed with the clinical genetics subteam and a two part clinical report was generated consisting of a laboratory interpretation and clinical guidance. For the CLARITY challenge report, we did not include standard general genome reports of incidental findings usually provided by the genetics diagnostics lab because this information was not requested by the CLARITY challenge. While many steps of the pipeline are automated, certain steps require manual evaluation and inter-disciplinary discussion. An important feature of our analytical pipeline is that many elements of the pipeline are already actively used by members of our team in the analysis of clinical genomics cases. In sum, the CLARITY cases represent important analytical challenges, not only by themselves, but also due to the specific sequencing technologies employed and the unavailability of patients and patient DNA for further clinical evaluation and Sanger confirmation.

Overview of the results

W1 pedigree

The absence of family history is consistent with either *de novo* occurrence of either a dominant variant or with recessive inheritance; moreover, centronuclear myopathy has been described with both dominant and recessive inheritance patterns. Therefore, both modes of inheritance were considered. We report variants in two genes as possible candidates ranked in the following order.

The proband is likely compound heterozygous for two rare splice site variants in Titin (*TTN*) (assuming a recessive mode of inheritance). Titin has a highly relevant molecular function and its potential role in centronuclear myopathy is clearly supported by expression data and protein-protein interaction data. However, we also recognize that spurious findings are possible given that *TTN* is the longest gene in the genome; compound heterozygous missense changes are particularly frequent. However, we estimate the chance to detect a *TTN* compound heterozygote for nonsense or splice variants, as observed here, is about $8x10^{-5}$ in the general population.

The proband also likely carries a *de novo* missense change in *CLIP1* (assuming a dominant mode). The *CLIP1* gene product has a relevant molecular function and is highly and specifically expressed in muscle. We are also confident in our prediction that the missense change is damaging.

Additional variants were identified in the upstream analysis, but were not supported by the downstream analysis, including: (i) a *de novo* missense change in *FLYWCH1*; (ii) a *de novo* synonymous change in *CA3* without clear support for an impact on splicing; (iii) a homozyous rare missense variant in *TRIM50*; and (iv) compound heterozygous missense variants in *MYO5B*.

W2 pedigree

The clinical assessment of the pedigree suggested that the conduction phenotype is likely to be shared by all affected pedigree members. The resolved ventricular mass in the proband was thought to potentially represent a separate phenotype specific to the first trio. The structural heart defects seen in the second trio were thought to more likely represent an independent phenotype not relevant to the proband.

For the conduction defect, we identified a single strong candidate. A very rare missense change in *TRPM4* segregates with the conduction defect in the W2 pedigree. *TRPM4* encodes a cation channel known to be involved in conduction defects. The inheritance is dominant with a suggested gain-of-function mechanism. One reported pedigree involves variable expressivity (AVB vs. RBBB) similar to the variation observed in W2. In addition, a number of known missense changes involved in conduction defects are located in the same N-terminal domain as the newly identified missense variant. Comparative genomic analysis suggests a possible gain-of-function mechanism and parallels the observation for another known mutation in *TRPM4* (see Long Report).

The upstream analysis also identified a rare CNV harboring a microRNA gene in some individuals; however, since we lacked complete genome data for one affected individual, we could not assess segregation definitively. In addition, the downstream analysis provided no support for the causal role of this CNV.

The resolved ventricular mass phenotype in the proband is a clinical scenario seen in tuberous sclerosis complex; however, we also recognize that it could be non-genetic (e.g., thrombus). We did not find any potential candidates in the established candidate genes *TSC1* and *TSC2*. Considering an alternative genetic etiology, we identified one rare homozygous variant in *PRKG1* involved in a cardiac phenotype in mouse. We also identified a *de novo* missense change in *FXBO4,* although additional support for this variant is relatively weak. The upstream analysis also identified a *de novo* intronic change in *XPO4*. The *XPO4* mutation was found to have a low predicted impact on splicing in the downstream analysis. The possibility of an inherited dominant variant in mTOR pathway was effectively excluded by an inability to identify candidate variants.

Thus, for W2 there are five potential candidates to explain the structural defect in the second trio (not involving the proband) ranked in the following order.

A nonsense variant in *KCNH8* not observed in ESP. *KCNH8* is involved in the human ventricular muscle proliferative response to stress as well as aorta smooth muscle proliferation and migration.

A missense variant in *TNC*. TNC is involved in local vascular remodeling by virtue of increasing migratory capacity of cardiac microvascular endothelial cells. The missense variant has been confidently predicted to be damaging. Six alleles carrying this variant are observed out of 6800 alleles in the ESP cohort.

A missense change in *SMYD1*. *SMYD1* is highly expressed in heart and loss-of-function of *SMYD1* results in right ventricular hypoplasia in mice. The missense variant has been confidently predicted to be damaging. Six such alleles out of 6800 alleles are observed in the ESP cohort.

A very rare missense variant in *NEBL*. *NEBL* is a gene with relevant biological function and high expression in heart.

A stop loss variant in *IL1Rl1*. An association between this gene and heart failure has been established in different heart failure cohorts.

W3 pedigree
We considered both dominant and recessive modes of inheritance since both modes are described for nemaline myopathy in the literature. We report a single gene as a possible candidate. **The proband is likely compound heterozygous for rare missense variants in *OBSCN*.** *OBSCN* has a relevant biological function and its potential role in nemaline myopathy is supported by strong and specific expression in muscle. We predict that missense mutations are highly damaging. Given the length of the gene, we estimated that there is relatively a high frequency of individuals with a missense compound heterozygote in the general population (~0.9%).

Additional variants identified in the upstream analysis but not supported by the downstream analysis include: (i) missense variants in *USP6* (with relatively low variant call quality); (ii) a *de novo* missense variant in *ARHGDID*; and (iii) a *de novo* synonymous variant in *DCAF17*.

This submission
In addition to this short summary, we include a long scientific report, three clinical reports, supplementary images, supplementary tables, and supplementary VCF files.

Figure 1. Major steps of the analysis pipeline.

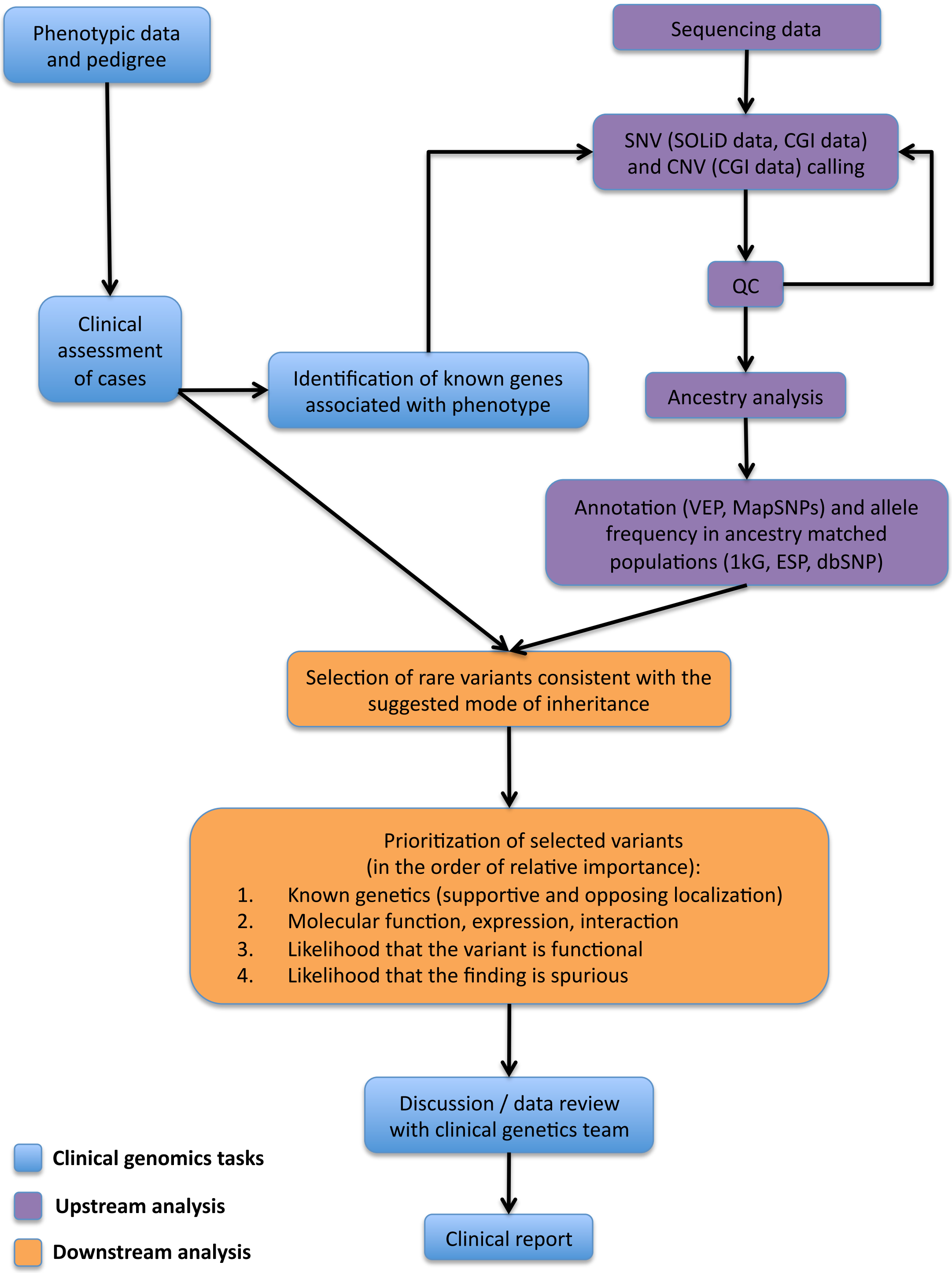

Supplement: Additional file 1 — The complete entry from the Brigham and Woman’s Team containing seven PDF files, six PNG image files, and one XLS table. [file gb-2014-15-3-r53-S1.zip › Additional_file_1/BWH Short Summary FINAL.pdf]
