# Supplementary material for: An international effort towards developing standards for best practices in analysis, interpretation and reporting of clinical genome sequencing results in the CLARITY Challenge
Source: Genome Biol. 2014 Mar 25;15(3):R53. doi: 10.1186/gb-2014-15-3-r53 (PMC4073084; doi:10.1186/gb-2014-15-3-r53)
Supplement: Additional file 1 — The complete entry from the Brigham and Woman’s Team containing seven PDF files, six PNG image files, and one XLS table. [file gb-2014-15-3-r53-S1.zip › Additional_file_1/CLARITY Challenge Cvr Ltr.pdf]

BRIGHAM AND WOMEN'S HOSPITAL

HARVARD MEDICAL SCHOOL

HMS New Research Building
77 Avenue Louis Pasteur
Boston, Massachusetts 02115
Tel. 617-525-4735
Fax 617-525-4751
E-mail ssunyaev@rics.bwh.harvard.edu

Shamil R. Sunyaev, Ph.D.
Associate Professor of Medicine (Genetics)
Division of Genetics
Department of Medicine
Brigham and Women's Hospital
Harvard Medical School

September 29, 2012

CLARITY Challenge
Boston Children's Hospital
300 Longwood Avenue
Boston, MA 02115

RE: BWH-Affiliated Team
CLARITY Challenge Materials

Dear Judges:

I am delighted to submit materials on behalf of the BWH-Affiliated Team for the 2012 CLARITY Challenge sponsored by Boston Children's Hospital. My many talented colleagues and I derived considerable satisfaction from the challenges posed by this initiative, and believe that we have arrived at satisfactory solutions to CLARITY cases W1, W2 and W3 and a highly efficient analytical pipeline for mutation discovery from clinical genome sequence data.

Our submission includes three clinical reports, a short scientific report accompanied by the full scientific report. We also include one supplementary figure and one supplementary table, IGV images corresponding to the most promising candidate variants and VCF files for the three pedigrees.

While we believe that cases W1 and W3 most likely have compelling single gene solutions, case W2 is more complex, with a single compelling candidate for the cardiac conduction system phenotype, but the possibility of additional contributory genes (and even possibly non-genetic causes) to the structural cardiac defect phenotype. Also, it goes without saying that a definitive assignment of causality will require yet further analyses, including confirmatory Sanger sequencing and functional experiments. For *e.g.*, in W1, where we assign compound heterozygous splice mutations in *TITIN* as the most likely etiology, the appropriate site directed mutants are currently being subjected to *in vitro* splicing analyses.

In addition to our best efforts to solve these interesting cases, we present a robust sequence analysis pipeline that integrates both automated computational analyses with incisive input from a clinical genetics group. The pipeline is sequence platform independent, and already incorporates several features that are already in routine use for clinical genome sequence analyses at multiple sites by our Team members.

I hope you will enjoy evaluating our CLARITY Challenge materials, and look forward further discussions with your panel and with the other teams.

On behalf of our CLARITY Challenge Team,

Shamil R. Sunyaev, Ph.D.
