# Supplementary material for: An international effort towards developing standards for best practices in analysis, interpretation and reporting of clinical genome sequencing results in the CLARITY Challenge
Source: Genome Biol. 2014 Mar 25;15(3):R53. doi: 10.1186/gb-2014-15-3-r53 (PMC4073084; doi:10.1186/gb-2014-15-3-r53)

Scientific Report Table of Contents
CLARITY Challenge Submission

Team leader: Shamil Sunyaev, Ph.D.

1. Clinical Assessment of Cases
2. Upstream Analysis of Sequencing Data
    a. SNV and Short Indel Calling
    b. Variant Calling Quality Control (QC)
    c. Analysis of Variants in Known Genes
    d. Ancestry Analysis
    e. Variant Annotation
    f. Allele Frequency Annotation
    g. Annotation of Copy Number Variants (CNVs)
3. Downstream Analysis of Sequencing Data
    a. Literature Analysis of Known Genetics and Molecular Function
    b. Analysis of Gene Expression
    c. Analysis of Protein-Protein Interactions Data
    d. Prediction of Functional Significance of Missense Variants
    e. Comparative Genomics Analysis of *TRPM4*
    f. Prediction of the Effect on Splicing
    g. Analysis of microRNA gene *MIR4268*
    h. Random Expectation for the Number of de novo Variants in Each Gene
    i. Random Expectation of Compound Heterozygotes in Each Gene
    j. Summary of Case 1 (W1)
    k. Summary of Case 2 (W2)
    l. Summary of Case 3 (W3)
4. Preparing Clinical Reports

Clinical Assessment of Cases

CASE 1: CENTRONUCLEAR MYOPATHY (CNM) IN 10-YEAR-OLD BOY

Clinical Record Review:

Affected (1-1) is a 10 year old Caucasian boy with myopathy presenting in first year of life. Specific diagnosis of CNM at 13 months of age on basis of muscle biopsy. Noted to have neurocognitive intact, normal CK, normal EMG, and normal cardiac echocardiogram. Noted to have nocturnal hypoventilation, mild sensorineural hearing loss, scoliosis and poor weight gain (3%ile).

Mother of proband (1-2) is a 42 year old Caucasian woman with no history or suggestion of skeletal myopathy reported. She reportedly has thyroid disease and premature ovarian failure. She has uncharacterized "arrhythmia" with no additional data, specifically no report of cardiomyopathy.

Father of proband (1-3) is a 45 year old Caucasian man with no history or suggestion of skeletal myopathy reported. He reportedly has myopia and optic drusen, and he has hemochromotosis reported in maternal uncles; we are not aware of any associations between these conditions and CNM.

Family History the isolated presentation of CNM myopathy in a boy in the first year of life appears to have occurred without suggestion of either overt or unrecognized/subclinical disease in first, second or third degree relatives. It should be kept in mind that CNM has a x-linked recessive caused by MTM1 mutations, which can be non-penetrant or clinically mild in heterozygous females. Variants for the MTM1 gene in mother and child should be considered and if found the mother should be clinically evaluated.

Clinical Genomics Diagnostic Impression:
The clinical history suggests that CNM has occurred in the only child of an unaffected couple. The known inheritance patterns for this condition include autosomal dominant, x-linked recessive, and autosomal recessive. Mutations in the four known associated genes in the table below do not explain all cases and additional genetic associations should be sought if careful review of these candidate genes fails to identify a causative mutation.

Table 1-1 – CNM known gene associations

Disease	Gene Symbol	Gene Name	Mendelian	Anticipate
CNM1	DNM2	dynamin-2	AD	de novo 1-1
CNM1	MYF6	Myogenic factor-6	AD	de novo 1-1
CNMX	MTM1	Myotubularin	XLR	de novo 1-1, or inherited from 1-2
CNM2	BIN1	bridging integrator 1	AR	homozygous 1-1 or compound het 1-1

Bioinformatic Considerations

- In CNMX, the occurrence of at least one deep intronic mutation detected by cDNA and protein analysis has been described. Deletions and duplications are known mechanism in *MTM1*.

Recommendations for Initial Mutation Analysis

- Interrogate proband sample for mutations in *DNM2*, *MYF6*, *MTM1*, *BIN1*

CASE 2: <u>CARDIAC DISEASE</u>

<u>Clinical Record Review</u>:

<u>A. TRIO ONE</u>

<u>Affected Proband (2-1</u>) is a 4 year old Caucasian girl with a right ventricular mass (3/4 of ventricle) recognized at DOL1 and spontaneously resolved at 3 months. Aortic root upper limit of normal. Persistent RBBB recognized first at DOL1.

<u>Mother of proband (2-2)</u> is a 35 year old Caucasian woman with transient newborn murmur and incomplete RBBB recognized in newborn period and spontaneously resolved at age 12 years.

<u>Father of proband (2-3)</u> is a 40 year old Caucasian man with no findings reported in the medical record. "Normal" or "none" in all data categories.

<u>B. TRIO TWO</u>

<u>Affected 1st Cousin (2-4)</u> was a Caucasian boy who died at 12 days of life with high grade AV block requiring pacemaker. Complex congenital heart disease with dilated RA, hypoplastic TV, hypoplastic RV, membranous VSD, hypertrophic LV, bicusp AV, single coronary ostium, PDA.

<u>Aunt of proband (2-5)</u> is a 31 year old Caucasian woman with migraine HA and otherwise unremarkable medical history.

<u>Uncle of proband (2-6)</u> is a 32 year old Caucasian man with pacemaker for type II AV block (age 7). Pulmonary valve stenosis requiring balloon dilatation.

<u>C. COMBINED TRIOS</u>

<u>Family History</u>

Structural heart disease including the pulmonary valve has occurred in three individuals (2-4, 2-6 and deceased maternal aunt), but not the proband (2-1) this may be consistent with an AD risk in those individuals. The proband (2-1) shares only conduction defect with the other "affected" family members (2-2, 2-6, 2-4). The appearance and spontaneous resolution of an intracardiac mass in the newborn period does not appear to be related to the structural disease or the conduction defect.

<u>Clinical Genomics Diagnostic Impression</u>:

We suspect that the clinical ascertainment of these trios may have allowed for the connection of cases that have potentially eitiologically unrelated phenotypes. Although all the affected individuals have "heart disease" the three different aspects of heart disease – namely the conduction system, the anatomic structure, and the poorly defined "intraventricular mass" – are not necessarily related. There is the possibility of mis-attribution if there is an assumption that a single variant is causative for all aspects of these varied phenotypes. We note that the "ventricular mass" in the proband is either a benign tumor (raising the question of Tuberous Sclerosis complex - TSC) or a non genetic cause such as a "clot". The finding of a mass in a newborn where TSC is suspected should prompt a careful clinical evaluation of both parents for subclinical TSC, it is not clear from the available data that this has been done.

Table 2-1 – Potential gene associations

Disease	Gene Symbol	Gene Name	Mendelian	Comment
Tuberous Sclerosis	*TSC1*	Hamartin	AD	Trio one analysis
Tuberous Sclerosis	*TSC2*	Tuberin	AD	Trio one analysis
Tuberous Sclerosis pathway disease	multiple	--	Unknown	mTOR pathway genes trio one
Congenital structural and electrical heart defect	*NKX2-5*	NK2 Homeobox 5	AD	Shared 2-1, 2-2, 2-4, 2-6
Atrial conduction defects	*AKAP10*	A-kinase anchor protein	AD	Shared 2-1, 2-2, 2-4, 2-6

Genetic susceptibility to conduction defects	*ID2*<br>*RXRA*<br>*HF1B/SP4*<br>SCN5A<br>*NRG1*			Shared 2-1, 2-2, 2-4, 2-6

Bioinformatic Considerations

- Considering Trio 2 in isolation, interrogate for heterozygous variants shared by 2-4 and 2-6.
- Considering Trio 1 in isolation, all manners of inheritance are conceivable (including heterozygous mutation from 2-3 to 2-1) as it is unclear whether the phenotype of 2-2 and 2-1 are indicative of the same genetic alteration.

Recommendations for Mutation Analysis

- Pursue heterozygous genetic variant present in all affected individuals (2-1, 2-2, 2-4, 2-6) to seek explanation for cardiac conduction phenotype.
- Right ventricular mass could represent cardiac rhabdomyoma suggestive of Tuberous Sclerosis Complex; consider heterozygous variants in mTOR pathway in patient 2-1. All manner of inheritance should be considered: inherited from 2-2, 2-3, or *de novo* as phenotype can be subtle.
- Independently pursue heterozygous genetic variants shared by 2-6/2-4 as these could be distinct conditions.
- If no mTOR candidate variant is identified in 2-1, pursue heterozygous variants shared by 2-1/2-2.

CASE 3: NEMALINE MYOPATHY (NEM) IN A 6-YEAR-OLD BOY

Clinical Record Review:

Affected (3-1) is a 6 year old Caucasian boy with myopathy presenting in first year of life. He was specifically diagnosed with NEM at 7 months of age on basis of muscle biopsy. Noted to have neurocognitive intact, normal CK, and normal cardiac echocardiogram. Noted to have osteoporosis, bilateral club feet, and poor weight gain (5%ile).

Mother of proband (3-2) is a 39 year old Caucasian woman with no history of skeletal myopathy reported. She reportedly had a congenital issue with lower extremities and has scoliosis.

Father of proband (3-3) is a 43 year old Caucasian man with no history or suggestion of skeletal myopathy reported. He reportedly has plantar fascitis and "muscle cramping with exercise".

Family History the isolated presentation of NEM myopathy in a boy in the first year of life appears to have occurred without suggestion of either overt or unrecognized/subclinical disease in first, second or third degree relatives. The proband has a reportedly unaffected 16 month old sister.

Clinical Genomics Diagnostic Impression:
The clinical history suggests that NEM has occurred in the first of two children of an unaffected couple. The known inheritance patterns for this condition include autosomal dominant, and autosomal recessive. Mutations in the six known associated genes in the table below do not explain all cases and additional genetic associations should be sought if careful review of these candidate genes fails to identify a causative mutation.

Table 3-1 – NEM known gene associations

Disease	Gene Symbol	Gene Name	Mendelian	Anticipate
NEM1	*TPM3*	Tropomyosin-3	AD or AR	de novo 3-1 or homozygous 3-1 or compound het 3-1
NEM2	*NEB*	Nebulin	AR	homozygous 3-1 or compound het 3-1
NEM3	*ACTA1*	alpha-actin-1	AD	de novo 3-1
NEM4	*TPM2*	Tropomyosin-2	AD	de novo 3-1
NEM5	*TNNT1*	troponin T1	AR	homozygous 3-1 or compound het 3-1
NEM6	*KBTBD13*	Kelch repeat and BTB/POZ domains-containing protein 13	AD	de novo 3-1

Bioinformatic Considerations

- NEM2 is associated with an increased carrier frequency in some populations, e.g. Ashkenazi Jewish, therefore cannot use stringent MAF filter to rule out significant change
- Known genes may only account for 30% of clinically diagnosed NM; of those 25% are attributed to *ACTA1*

Recommendations for Mutation Analysis

- Interrogate proband sample for mutations in *TPM3, NEB, ACTA1, TPM2, TNNT1, KBTBD13*

Upstream Analysis of Sequencing Data

Upstream analysis included Single Nucleotide Variant (SNV) calling, calling of short insertions and deletions, Copy Number Variant (CNV) calling, QC procedures, basic functional annotation, ancestry analysis and annotation with allele frequencies in the general population.

Many members of our team are actively involved in sequencing studies including diagnostic studies in pedigrees, using sequence reads from Illumina Hi-seq. This team has a standard set of QC metrics that we usually demand for the analysis of sequencing data (e.g. Kiezun, Garimella, Do, Stitziel, *et al*., Nature Genetics 2012). The team needed to adapt the different technology platforms used in the CLARITY challenge, and thus upstream analysis required several rounds of variants calling with successive QC until the team converged on a single set of annotated variants.

SNV and Short Indel Calling

We reformatted SOLID Whole Exome Sequence (WES) alignment files (BAMs) to reorder reads in accordance with the UCSC-style h19 human genome reference and to make the data compliant with the GATK Unified Genotyper engine. We restructured Complete Genomics Inc. (CGI) Whole Genome Sequence (WGS) data into BAM format using standard cgatools modules and then cleaned to be ready for Genome Analysis Tool Kit (GATK) variant calling. For CGI WGS, we used only evidence-BAMs due to CGI proprietary local *de novo* reassembly technology for evidence files that should produce better call results.

We used parts of Medical Genomic Sequencing Pipeline (medGS) that is being developed by our group for the analysis of Illumina sequencing data in downstream analysis (Figure 1).

Figure 1 Screenshot of Medical Sequencing Pipeline (medGS) software.

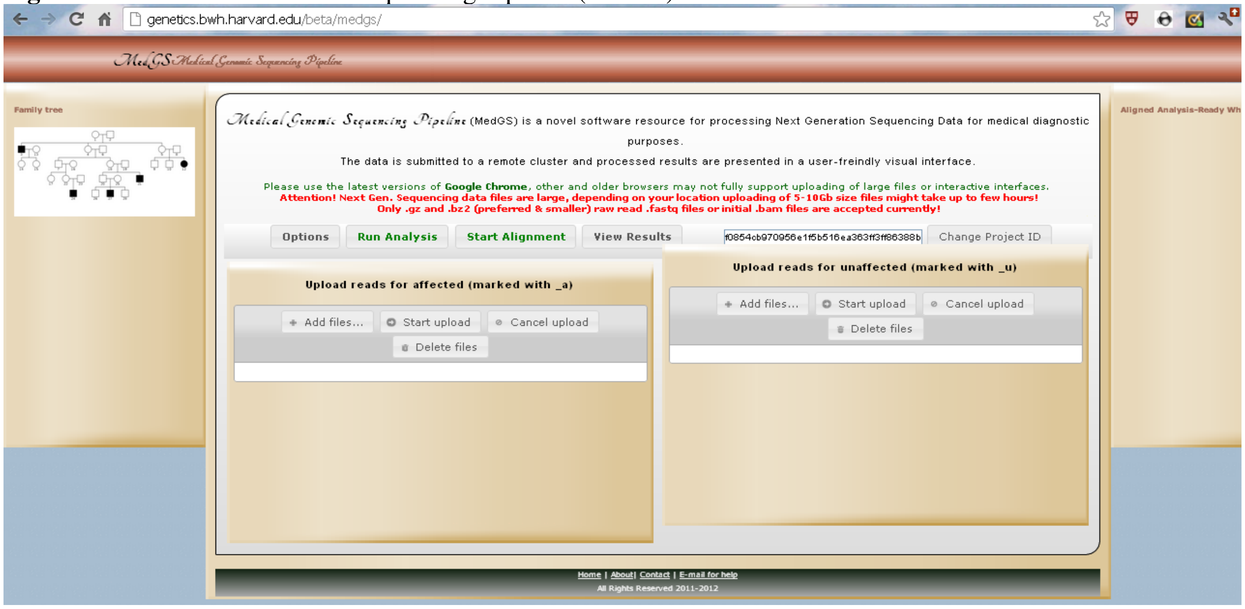

The medGS pipeline is being designed for fast parallel sequence processing and analysis, with interactive output of possible candidates and means for assessment and verification. The pipeline primarily relies on Burrows-Wheeler Aligner (BWA) and GATK Unified Genotyper.

Target region intervals were downloaded for the capture technology used in producing SOLiD WES. The calling by GATK was limited to these regions +/-10bp only to remove any of target reads.

Our original plan was to leverage pedigree information in the process of SNV calling. We planned to use a newly developed trio-aware caller entitled "Phase by Transmission". Phase by Transmission is a GATK module that computes the joint posterior probability of each possible genotype combination at a bi-allelic site in a parents-child trio. This method has shown excellent results in Illumina data. However, variant calling QC suggested that the results on the SOLiD data were quite poor. The description of the method and the discussion of the applicability to data generated on the SOLiD platform are described in **Appendix I**.

QC analysis described in the subsequent section raised concerns regarding call qualities for CGI WGS only and SOLiD WES only datasets. The problems were attributed in part to the presence of duplicated reads and to technology differences. Removal of duplicated reads in WES data led to considerable loss of non-duplicated coverage due to single-end read nature of the data. Therefore, the team has made a decision to use a coherent set of SNPs/variants detected in both WGS and WES datasets.

QC analysis of consistent SNV calls identified that allelic balance distribution for SOLiD WES calls was substantially shifted towards the reference allele unlike the perfectly centered and symmetric distribution for CGI WGS calls (see discussion and Figure 3 in the QC section). We assumed that this is most likely due to a bias in the targeted selection technology towards selecting reference vs. non-reference fragments. We developed specific genotyping criteria different from GATK default options to accommodate this finding.

A consensus WGS/WES genotype was called by the pipeline for each sample. Due to complexity and variability of the data, the genotype spectra were widened to allow for a human readable genotype interpretation. We used the following "genotype" notations:

HOMO_A - homozygous alternative allele
HOMO_R - homozygous reference allele
HOMO_Ap - homozygous alternative allele but with <5% admixture reads

HOMO_Rp - homozygous reference allele but with <5% admixture reads
HOMO_plcA - probably alternative homozygous or heterozygous with 15% admixture reads (low quality) - most likely false SNP
HOMO_plcR - probably referencehomozygous or heterozygous with 15% admixture reads (low quality) - most likely false SNP
HET_Un - heterozygous but with low coverage
Unc - non-covered in this sample
HET_nowgs - heterozygous but no WGS confirmation (might be a SOLID error or WGS not done like in several samples)
HET - relatively good heterozygous call

This discrete sequencing technology specific genotype interpretation allows to rapidly screen for best SNP/variation candidates according to the desired inheritance pattern. The general upstream data processing scheme (including annotation steps described below) is depicted in **Figure 2**.

Figure 2 The general upstream data processing pipeline for SOLiD WES and CGI WGS data.

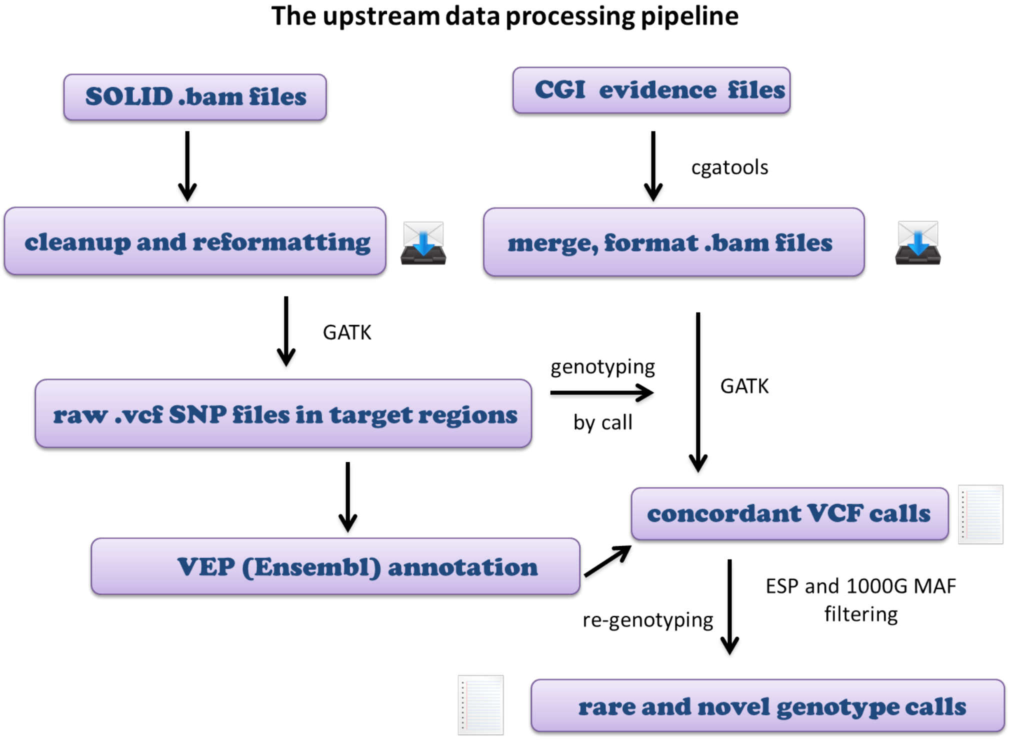

Variant Calling Quality Control (QC)

We checked if the resulting list of SNVs had statistical properties that were similar to previous studies and as expected from population genetics. Statistics derived on population genetics principles are independent from sequencing technologies, and can help highlight technical problems with the data. These statistics include the transition to transversion (Ti/Tv) ratio, fraction of mutations in CpG contexts, ratio heterozygote to homozygote calls (HETs to HOMs), and the ratio of non-synonymous to synonymous calls.

To investigate the statistics we used three SNV lists. The first list is denoted by unfiltered SNP, this list composed of all variants that were called by GATK. Second group is of SNP that passed the quality control of GATK (denoted by Filter-PASS). The third group is variants that are found both in SOLiD and the CGI whole genome, these SNPs are considered the most reliable calls since they were detected by two independent platforms. Comparison of these lists motivated our final choice of the variant call set.

The ratio of HETs (0/1 in VCF files) to HOMS (1/1 in VCF files) is 1.5X in the full SOLiD data, which is slightly lower than 1.6 observed in ESP samples, This ratio of about 1.5 is observed in the two filtered variant i.e Filter-PASS and Filer-CGI.

Transition transversion (Ti/Tv) ratio is as expected. The ratio between transition mutations and transversion mutations is 2.9 for unfiltered SNPs and rise to 3.1 for the Filter-PASS variants list as observed by Keizun, Garimella, Do, Stitziel *et al*. (Nature Genetics 2012) and as expected by evolutionary models.

Table 1 SNV counts per SOLiD-sequenced sample

	Filter	Counts	HETs	HOMs	HETS/HOMS
Total	Unfiltered	30000	20000	10000	1.5
	Filter-PASS	22000	13400	8600	1.5
	Filter-CGI	26300	15800	10500	1.5
Functional					
Silent	Unfiltered	11515	7500	4015	1.87
	Filter-PASS	8649	5215	3434	1.5
	Filter-CGI	9954	5997	3957	1.5
Missense	Unfiltered	12000	8273	3785	2.18
	Filtered-PASS	7724	4674	3049	1.53
	Filter-CGI	9340	5687	3690	1.51
Nonsense	Unfiltered	160	134	24	5.6
	Filter-PASS	70	52	17	3
	Filter-CG	87	65	22	3

Underrepresentation of non-reference allele proportion in SOLiD HETs calls. In SOLiD data, the average proportion of non-reference alleles among the total number of reads for a specific variant is 0.43 while in CGI the average is about 0.49. The histogram below demonstrates that the distribution of alternative-alleles-proportion in CGI's HETs is centered close to 0.5 i.e. in a typical HET call about half of the reads carry the variant in the reference allele and the half is of reads that contain non-reference variant. In HETs called by SOLiD there is a significant underrepresentation of reads with non-reference alleles.

Figure 3 Histogram of Alternative-allele-proportion of heterozygote site reads in sample 1 of case W1 in CGI and SOLiD.

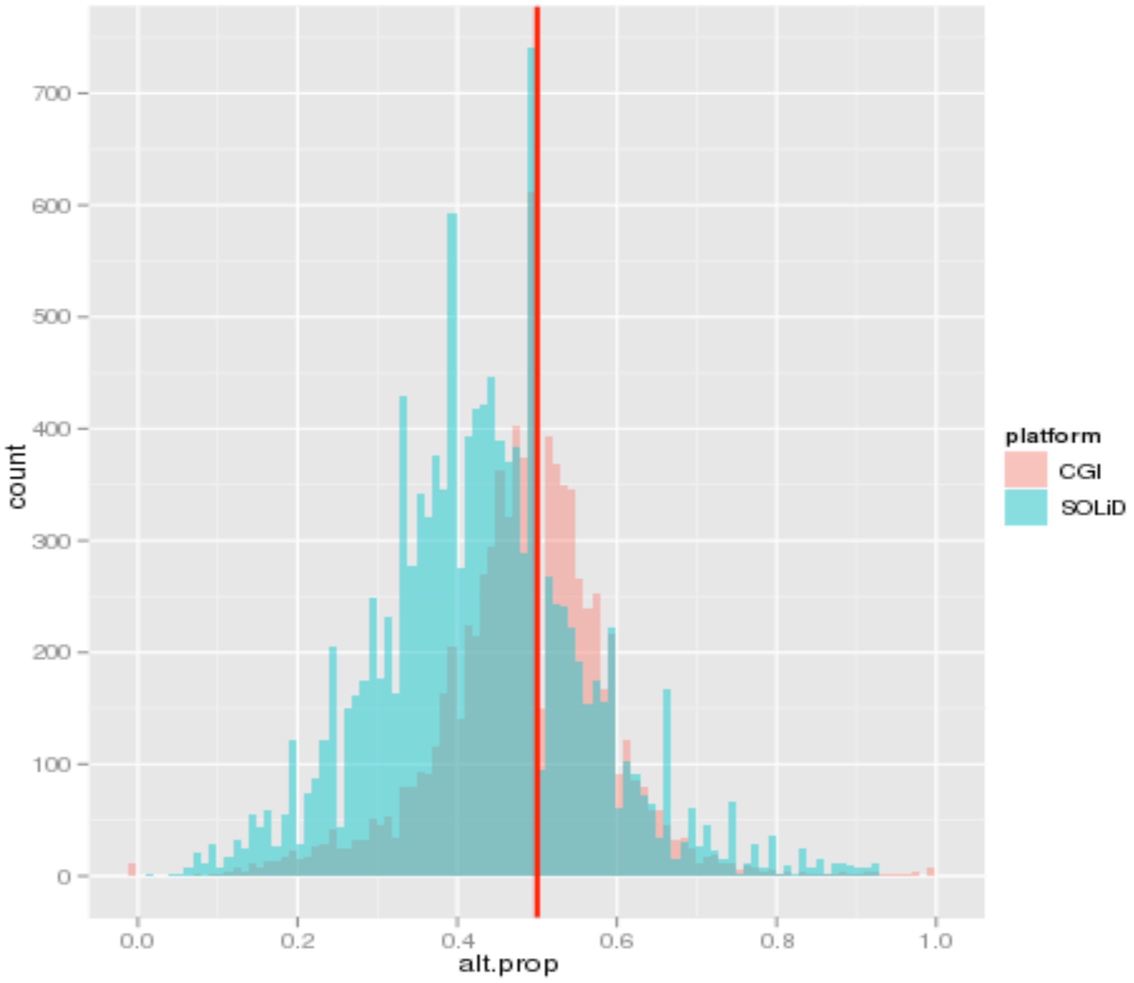

The above quality control procedures were instrumental for the pipeline modifications described in the SNV calling section above.

Analysis of Variants in Known Genes

Next, we specifically focused on genes that are involved in diseases of the CLARITY challenge patients. For cases W1 and W3, representing myopathies, we examined 63 genes published by the World Muscle Society as being involved in muscular dystrophies, congenital muscular dystrophies, congenital myopathies, distal myopathies and other myopathies. We also considered separately the three genes known to be associated with centronuclear myopathy and the eight genes previously linked to nemaline myopathy (Kaplan, Neuromuscul Disord 2011) , the diagnoses for families W1 and W3, respectively. Analysis of these genes is necessary to exclude known causes of these diseases.

Table 2 Genes known to be involved in centronuclear myopathy and nemaline myopathy.

Muscle Disease	Inheritence	Gene
Centronuclear myopathy	AD	DNM2
Centronuclear myopathy	AR	BIN1
Centronuclear myopathy	AR	RYR1

Muscle Disease	Inheritence	Gene
Nemaline myopathy	AD	TPM3
Nemaline myopathy	AR	NEB
Nemaline myopathy	AD	ACTA1
Nemaline myopathy	AD	TPM2
Nemaline myopathy with Escobar syndrome	AR	TPM2
Nemaline myopathy	AR	TNNT1
Nemaline myopathy	AD	KBTBD13
Nemaline myopathy	AR	CFL2

Key: AD = Autosomal Dominant, AR = Autosomal Recessive

Call-ability of known disease genes

We used a GATK pipeline to call variants in the SOLiD sequencing data. Although coverage is important, a more important metric is whether all bases within the 63 known muscular dystrophy genes could be called. The *CallableLoci* option in GATK allows this question to be addressed. The three reasons why variants could not be called were no coverage, low coverage and poor mapping. The un-callable bases also included mutation sites contained in the Human Gene Mutation Database (HGMD). In total there was 3526 single nucleotide mutation sites within HGMD.

Table 3 Call-ability data on the CLARITY challenge dataset

W1	Un-callable (%)	HGMD Un-callable (%)
Sample1	8.232	8.366
Sample2	9.350	8.735
Sample3	7.417	7.232

W3	Un-callable (%)	HGMD Un-callable (%)
Sample10	6.761	6.410
Sample11	6.869	7.317
Sample12	6.386	5.786

For comparison we show comparable data on exome sequencing performed at the Broad Institute using Agilent exome capture and Illumina sequencing. This dataset has a much lower proportion of un-callable bases.

Table 4 Call-ability data on a comparison dataset

Sample	Un-callable (%)	HGMD Un-callable (%)
1A	3.225	1.503
2A	3.262	1.333
3A	3.381	1.361
4A	3.609	2.411

The un-callable percentage highlights the challenges of exome capture and sequence analysis of muscle genes, which have large coding regions and repetitive regions. It also emphasizes that the raw sequence data provided for this challenge were of a substantially lower quality than the data used for most current clinical sequencing projects, increasing the probability that the true causal mutations were missed in these families. Figure 4 presents the overall coverage statistics over known disease genes in two trios (W1 and W3).

Figure 4 Coverage data over known disease genes in W1 and W3.

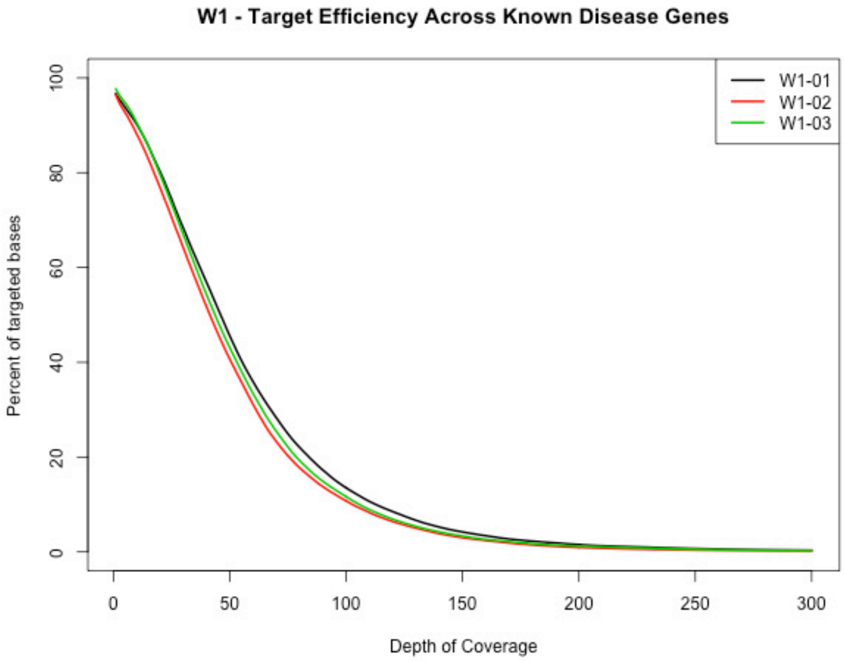

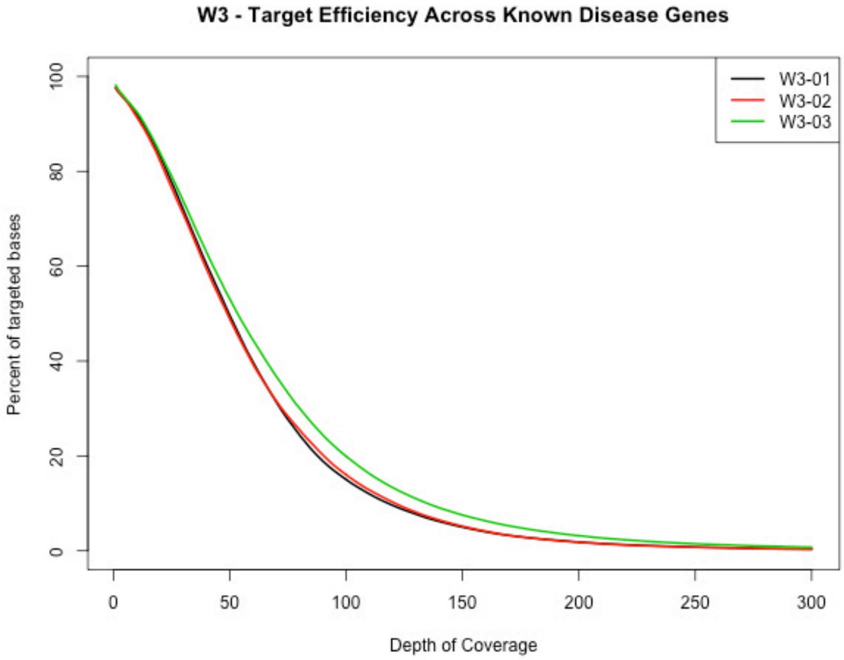

Similar analysis was performed for genes *TSC1* and *TSC2*, which may cause aspects of phenotype of the proband in pedigree W2 (2-1).

Ancestry Analysis

The parents in this study identified themselves as individuals of Western European ancestry. We wanted to confirm these self-assessments because of the following two main reasons: First, this type of analysis is another type of quality control. Second, in any study of rare variants one should use the right populations to determine allele frequency of SNVs in ancestry-matched populations.

The population stratification of our samples was done using the script smart_pca.pl in the EIGENSOFT package. We used variants from Omni2.5 genomes from the 1K (low coverage) genome project combined with variants from the 9 genomes that were sequenced by both SOLiD and CGI. The SNP were restricted to SOLiD target regions i.e. exome.

We concluded that the self reported ancestry was accurate. A scatter plot of the first and second principle component (PC1 and PC2) shows that all the 9 genomes in the 3 cases are clustered with Europeans populations. We further decided to re-run the PCA analysis using only Europeans genomes. In this case PC1 separated Finnish, Great Britain, CEU and southern European populations. The PC2 separated CLARITY challenge cases from the other European genomes presumably due to technology differences. Scatter plot of PC1 vs. PC3 shows that the CLARITY cases cluster well with West European populations (GBR and CEU).

Figure 5 Ancestry PCA plot of HapMap populations and the CLARITY challenge cases

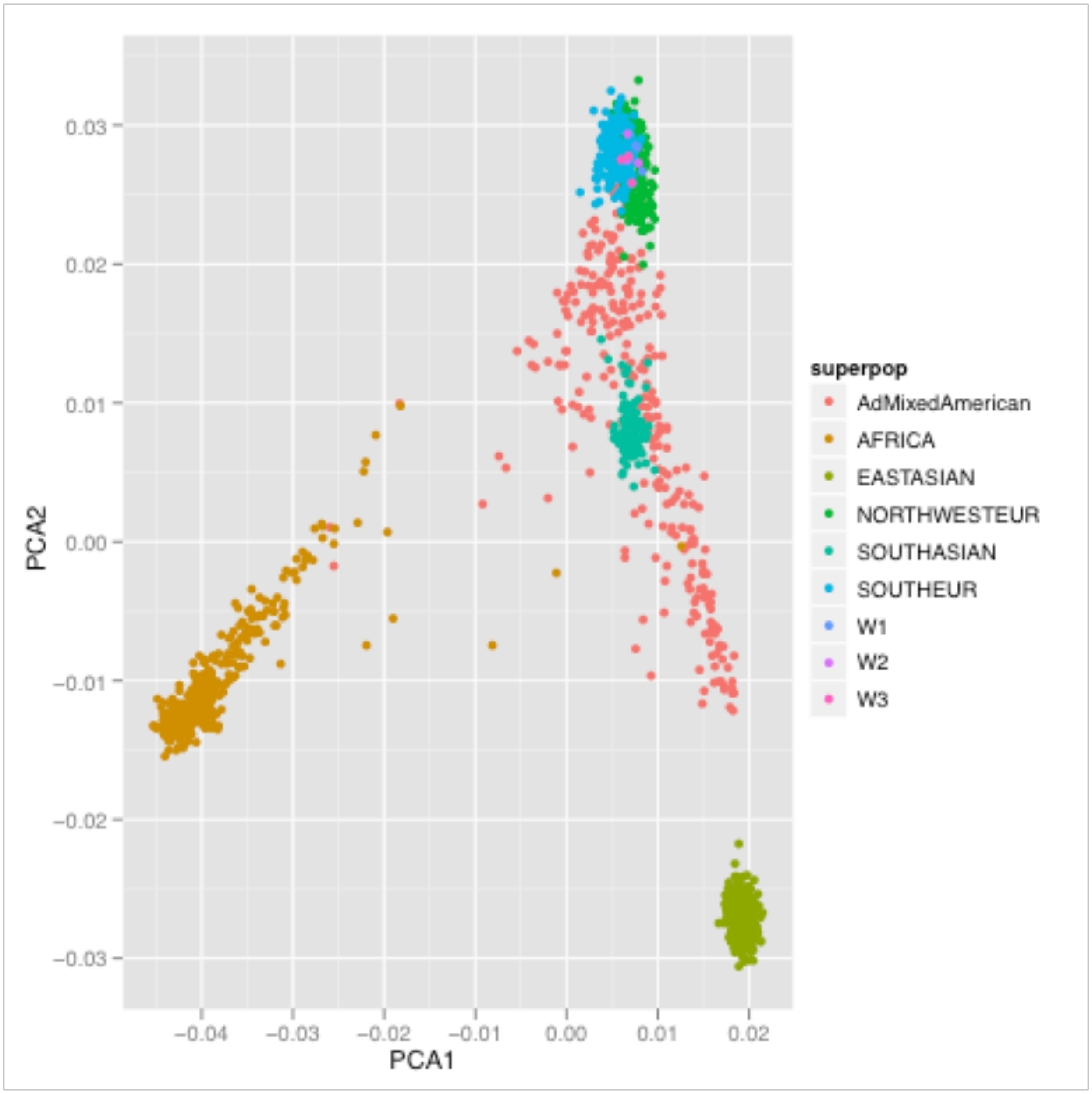

Figure 6 Ancestry PCA plot of European populations and the CLARITY challenge cases

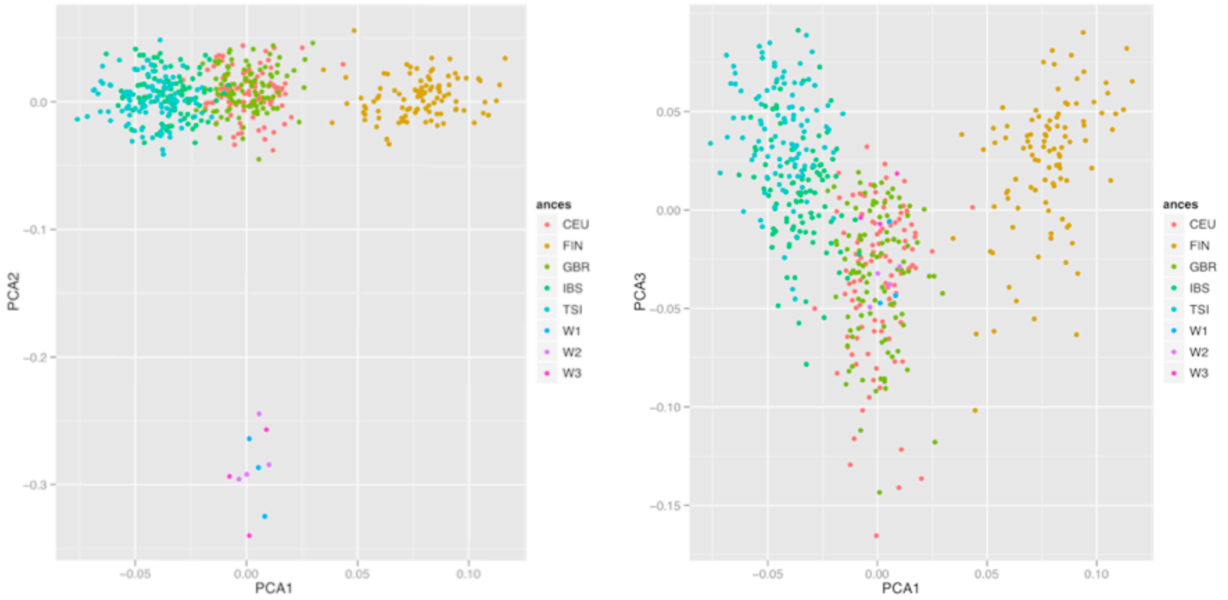

Variant Annotation

Produced SNV and indel calls were annotated with Variant Effect Predictor (by Ensembl). These calls were further re-genotyped in WGS evidence-BAM files and a concordant set of variants in VCF (Variant Call Format) files was produced. Being aware of annotation complexities in presence of multiple transcripts, we separately annotated variants using MapSNPs software. MapSNPs is an integrated annotation tool in the MapSNPs/PolyPhen-2 mutation effect analysis pipeline available via the PolyPhen-2 Web server and also as a part of the standalone PolyPhen-2 software. MapSNPs annotations for candidate variants were obtained using UCSC Genome Browser known Canonical set of gene models (GRCh37/hg19) After assigning functional classes to variants, various sequence context and positional-specific features describing mutations were extracted and non-synonymous variants located in the canonical transcripts were mapped to human protein sequences from the UniProtKB database (Release 2012_08). We also compared annotations for canonical transcripts with the corresponding alternative splice forms from the full knowGene set of UCSC gene models and found no major differences.

The resulting calls were further re-genotyped in WGS evidence-BAM files and a concordant set of variants in VCF (Variant Call Format) files was produced. These files are included with this submission.

The VEP annotation includes computational predictions of the functional effect of missense variants by PolyPhen-2 and SIFT software. We separately annotated variants with the latest version of the PolyPhen-2 software that we continuing to maintain and develop.

Allele Frequency Annotation

ESP6800 (NHLBI Exome Sequencing Project) and 1000 Genomes project datasets were used to filter the VCF files for rare (with MAF<0.1%) or novel variants.

The genotypes were used to produce *de novo*, rare compound-heterozygous, rare homozygous and dominant (if applicable) variation candidates. Compound-heterozygous SNPs were produced based on SOLiD target annotation and were checked for inheritance from different parents.

Candidate SNPs were visually verified in IGV (Integrative Genomics Viewer by Broad Institute MIT) for possible misalignments, repetitive regions or erroneous calls.

The resulting list of rare variants or de novo mutations that are compatible with the suggested segregation is given in Supplementary Material.

Annotation of Copy Number Variants (CNVs)

All CNVs from Complete Genomics data were included in this analysis. For each CNV region, population data were collected using Genome STRiP (Handsaker et al., Nature Genetics 2011). Genome STRiP assessed copy number in the selected regions in 946 individuals from multiple populations from 1000 Genomes Phase 1. All variants with a frequency of > 0.05 were excluded. Variants with frequency < 0.05 were checked for segregation if they overlapped any part of a gene. Any variants present in more than one family were filtered. Segregation analysis was also performed for any variants not present in the Database of Genomic Variants (which includes CNVs that are common in healthy individuals).

Finding
In family W2, a CNV was identified at chr2:220770000-220876000 that includes a microRNA gene MIR4268. No individuals in the 1000 Genomes Project have a deletion in this region. This CNV segregates in the available data: cases 2-1, 2-2, and 2-6 have the deletion and are affected, and case 2-3 does not have the deletion and is not affected. Confirmation through loss of heterozygosity analysis was conducted (by SNP inspection). This analysis confirms the expected inheritance pattern for a CNV deletion; W2-3 is heterozygous at many SNP loci throughout the deletion and the calls were homozygous minor for those SNPs in 2-1.

Insufficient data for complete analysis
The CGI whole genome sequencing data only includes individuals 2-1, 2-2, 2-3, and 2-6, so a complete segregation analysis including 2-4 and 2-5 is not possible. We were unable to use the WES data from SOLiD for confirmation because there was no targeted sequencing for this region. Untargeted SOLiD reads in this region were inconsistent with the CGI data and did not appear to be reliable.

Figure 7 Population analysis for CNV in family W2. Genome STRiP assessed copy number in the selected regions in 946 individuals from multiple populations from 1000 Genomes Phase 1. No individuals in the 1000 Genomes Project have a deletion in this region.

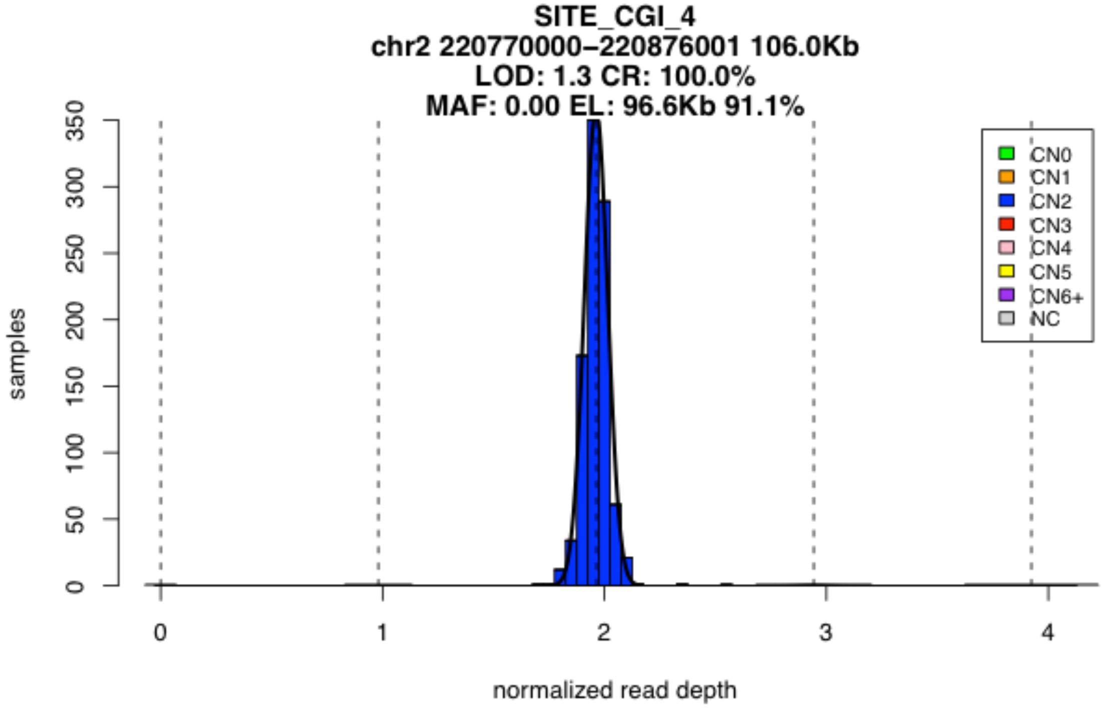

Figure 8 Identified CNV region chr2:220770000-220876000, viewed in IGV. The first major track includes untargeted SOLiD calls, the second track contains no targeted SOLiD reads, and the third track contains Complete Genomics data for individuals 2-1, 2-2, 2-3, and 2-6, respectively. Heterozygous calls are in royal blue, and homozygous minor calls are in cyan. The region (which includes gene MIR4268) in the third track shows that individuals 2-1 and 2-3 have matched calls (except that 2-3 is heterozygous at these SNP loci) and individuals 2-2 and 2-4 both have matched homozygous minor SNP calls in the region of the deletion.

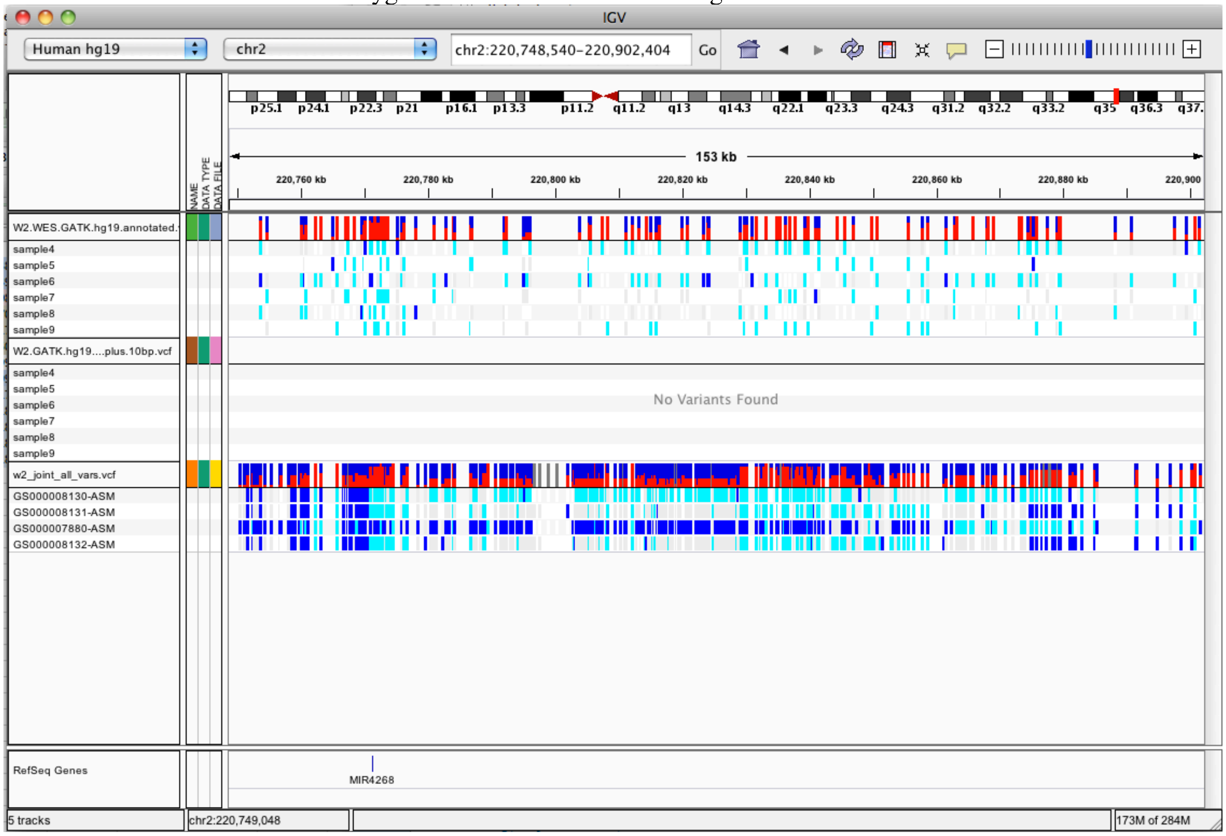

Downstream Analysis of Sequencing Data

Upstream analysis generated a manageable number of candidates. The goal of downstream analysis was to select candidates most likely involved in phenotypes in all three cases selected for the CLARITY challenge.

First, we assessed the potential phenotype of variants based on known phenotypes of candidate genes in humans and model organisms. Our confidence in variants was reduced, if we observed that known phenotypes are inconsistent with patient phenotypes. Second, we assessed the potential molecular function and cellular localization of candidate genes based on the literature, mRNA expression data, and data on protein-protein interactions. Third, we evaluated the potential for candidate variants to have a substantial effect on molecular function based on computational and manual analysis of amino acid sequence and comparative genomics data. We commenced minigene assays to evaluate variants with potential effect on splicing; these assays are ongoing. Fourth, we evaluated the potential for predicted candidate variants to represent technical artifact with a context-dependent model for de novo mutations and estimates rates of compound heterozygote and homozygote individuals for specific genes in the general population.

The following genes were selected for extensive downstream analysis. In W1, we analyzed genes with *de novo* mutations *CLIP1*, *CA3* and *FLYWCH1*, one gene harboring a rare homozygote variant *TRIM50*, *TTN* where the proband is compound heterozygous for two splice variants and *USP6* where the proband is compound heterozygous for missense variants (although the quality of variant calls was relatively low). In W2 our approach is against the Occam razor as we hypothesized that all affected individuals in the pedigree share a conduction defect but other phenotypes are specific to two trios in the pedigree. For the potential role in the conduction defect we analyzed *TRPM4* with a rare missense variant segregating in all affected individuals and microRNA gene *MIR4268*. For the

potential role in resolved mass in right ventricle and in dilated aorta we analyzed *PRKG1* and *LRRC37A* with homozygous variants, *FXBO4* and *XPO4* with a de novo mutations. We also evaluated genes involved in mTOR pathway for the presence of heterozygous transmitted variants. We analyzed several heterozygous transmitted variants as candidates for the structural defect (*KCNH8, TNC, IL1RL1, SMYD1, NEBL*).

Literature Analysis of Known Genetics and Molecular Function

We originally considered conducting literature analysis with the help of automated system finding most likely gene-phenotype associations (e.g. developed by Michael Bridno lab at the University of Toronto). However, our upstream analysis generated a relatively small list of candidates. We concluded that manual literature analysis in this case is both faster and more accurate. It is impossible to list all literature evidence (positive and negative) in this report. We include supportive evidence into Summaries of individual cases at the end of this document.

Analysis of Gene Expression

The GTEx data provides a rich gene expression resource and currently contains RNA-Seq data from over ten human tissues for each donor. Reads Per Kilobase of exon model per Million mapped reads (RPKM) was calculated for each gene, which serves as a normalized expression metric. The tissue expression profile of candidate genes may be used to rank potential candidate genes based on the tissue it predominately affects. In W1, *TTN*, *CLIP1* and *CA3* showed strong expression in muscle.

Figure 9 Tissue specific expression data on known centronuclear myopathy genes and W1 candidate genes

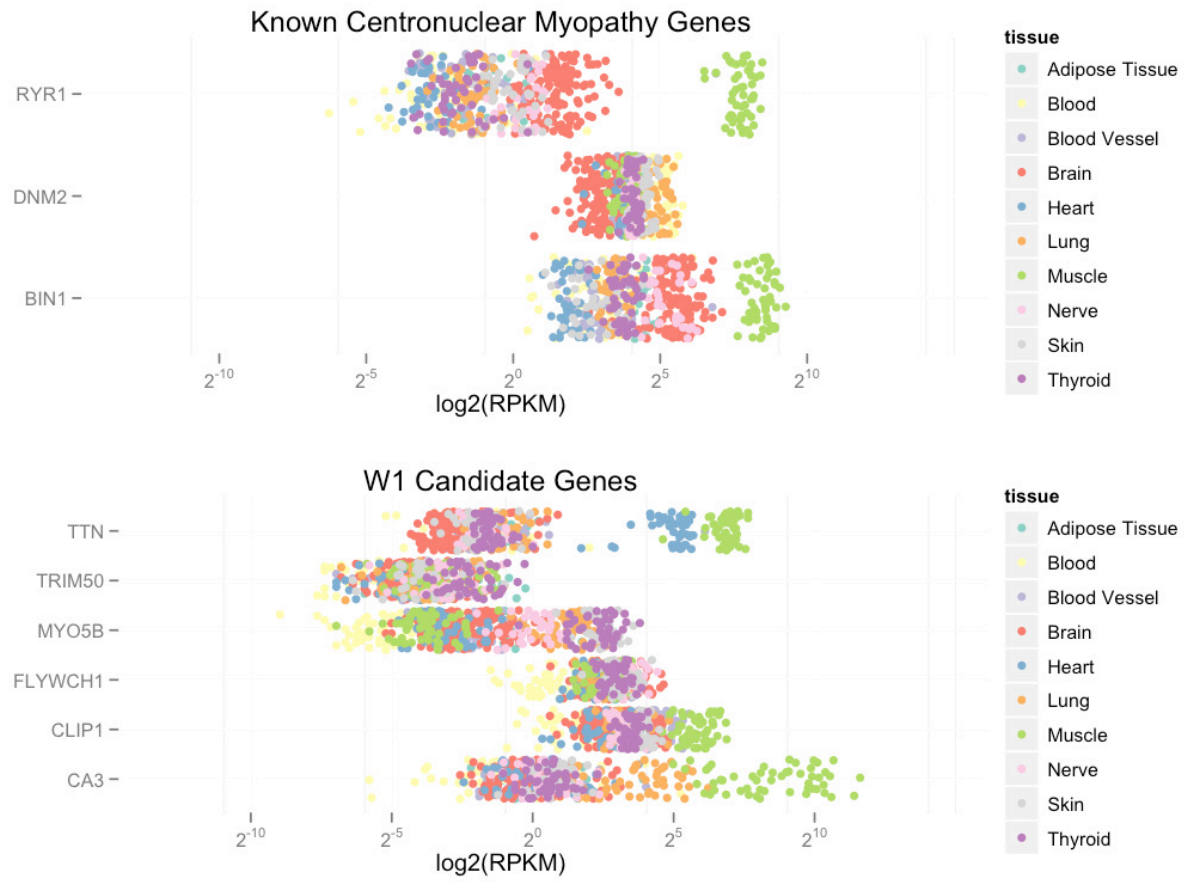

In W2, *NEBL* and *SMYD1* showed strong expression in heart. TRPM4 the most muscle-specific expression patterns. *TRPM4* and *XPO4* are expressed in heart. We note than *TNC* is known to be expressed during development but is not expressed in adult heart.

Figure 10 Tissue specific expression data on W2 candidate genes

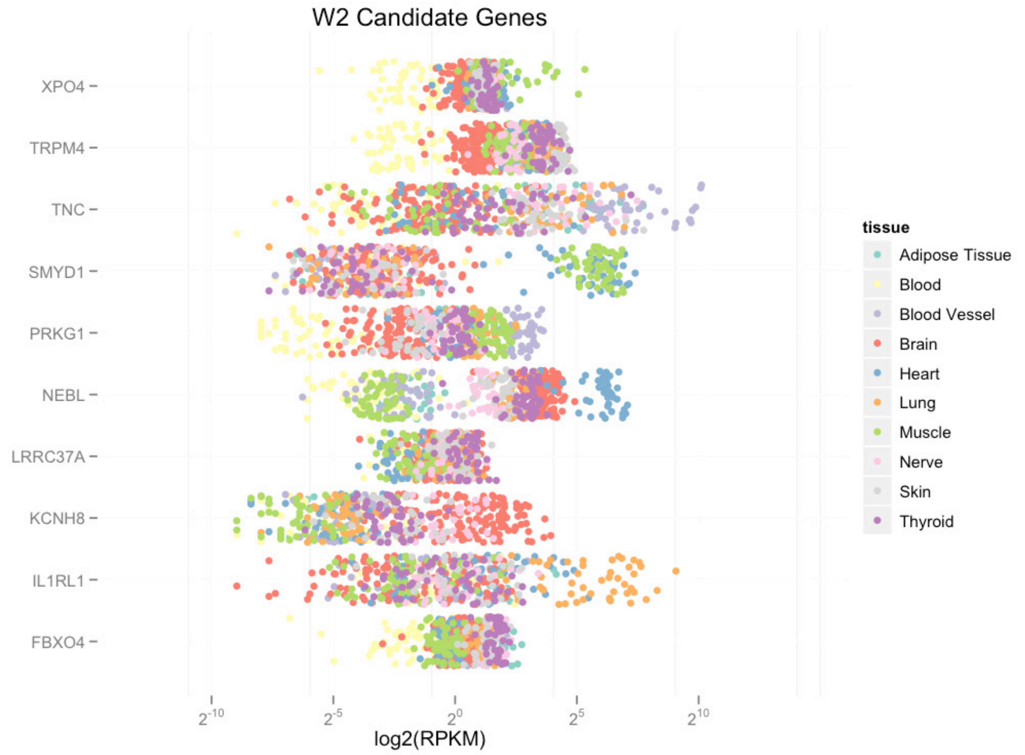

In W3, *USP6* and *OBSCN* showed the most muscle-specific expression patterns.

Figure 11 Tissue specific expression data on known nemaline myopathy genes and W3 candidate genes

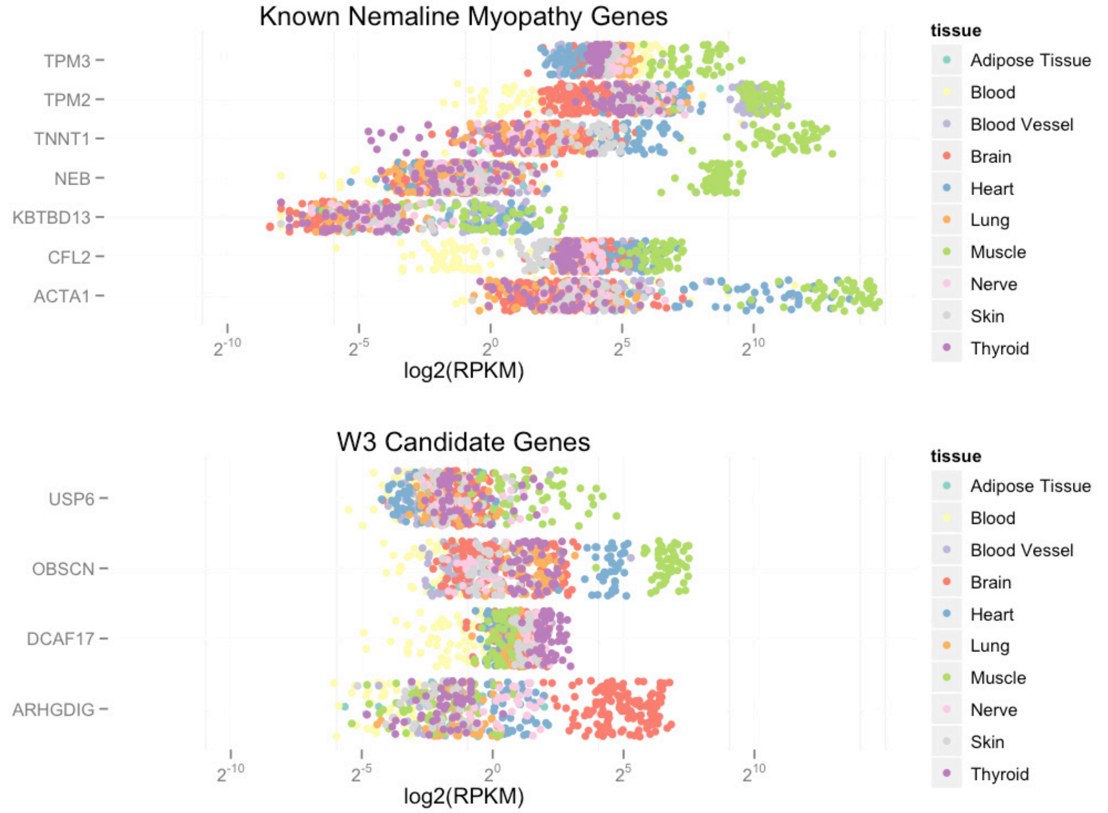

Analysis of Protein-Protein Interaction Data

In the analysis of protein-protein interaction date for this submission we relied on work by Kasper Lage, Tune Pers and Piotr Dworzyński.

The Interactome Based Affiliation Scoring (IBAS) tool uses a protein interaction database, InWeb to assess interaction patterns between proteins encoded by a list of known disease genes. This allows for new candidate disease genes to be predicted and scored based on their direct and indirect interaction with the known genes for a specific phenotype. A separate analysis was performed using the known centronuclear myopathy and nemaline myopathy genes to rank the candidate genes based on *p*-values. None of the candidate genes provided compelling evidence for inclusion within the protein interaction networks of the known genes associated with these two disorders, but there was nominal support for *TTN* in W1.

Table 5 Ranks and p-values of protein-protein interaction analysis of W1 Candidate Genes.

**Genes**	**Rank**	**p-value**
TTN	169	0.029
CLIP1	1111	0.978
FLYWCH1	2501	0.998
CA3	9357	0.998
MYO5B	9698	0.998
TRIM50	NA	NA

Table 6 Ranks and p-values of protein-protein interaction analysis of W3 Candidate Genes.

**Genes**	**Rank**	**p-value**
USP6	5006	0.998
OBSCN	7656	0.998
DCAF17	NA	NA
ARHGDIG	NA	NA

Prediction of Functional Significance of Missense Variants

We manually reviewed evidence underlying PolyPhen-2 predictions for all candidate missense changes. In W1, the manual review was in agreement with prediction of the *de novo* change in *CLIP1* as probably damaging, *de novo* mutation in FLYWCH1 as probably damaging, rare variant in *TRIM50* as benign, and rare variants in *MYO5B* as probably damaging. In W2 rare variants in *PRKG1* and *NEBL* were predicted possibly damaging, variants in *TNC* and *SMYD1* were predicted probably damaging. At the stage of manual review, the variant in *TRPM4* (predict benign) triggered in-depth analysis. The reasons for the this analysis and results are described below. In W3, de novo change in *DCAF17* was predicted benign, both *OBSCN* variants were predicted as probably damaging and variants in *USP6* were predicted benign, all in agreement with the manual review.

Comparative Genomics Analysis of TRPM4

The TRPM4 variant Val168Glu segregates with the conduction phenotype in pedigree W2. Glu in this position is observed in a number of paralogous sequences and in some vertebrate species. We earlier estimated that approximately 8% of human disease missense mutations are present in corresponding positions in vertebrate proteins as wild type amino acids. This can be attributed to compensated pathogenic deviations (Kondrashov et al., PNAS 2002). Alternatively, if amino acids corresponding to human mutations originate at duplication events, these changes can reflect functional residues. Given that the analysis of multiple sequence alignment revealed several potential duplication events and that all known mutations involved in conduction defects in *TRPM4* are gain-of-function changes, we decided to investigate this possibility.

Phylogenetic tree of TRPM4 homologs is shown in Supplementary Figure 1. It is seen that Glu corresponds to the ancestral variant predating a duplication leading to TRPM4. Val is, however, conserved in one-to-one orthologs. Most importantly, one known gain of function disease mutation in TRPM4 (Gln131His) shows a highly similar

pattern with His observed in remote vertebrate species prior to the duplication leading to TRPM4. Several well-known bioinformatics algorithms predict specificity determining residues based on coordinated changes between paralogous groups accompanied by conservation within orthologous groups. Thus, we concluded that in spite of Glu present in paralogous sequences, phylogenetic analysis of Val168Glu is consistent with this change resulting in a gain of function.

Prediction of the Effect on Splicing

In addition to protein coding changes, the causal mutation could alter the processing of a gene. The signals required for processing presents a large mutational target and are often a causal mechanism in human disease. Most hereditary disease alleles that affect processing affect pre-mRNA splicing. Approximately 14-15% of all hereditary disease alleles are annotated as splicing mutations (Stenson et al., 2003). Historically, most splicing mutations have been annotated on the basis of their overlap with canonical splice sites. Because of this narrow definition of splicing signals and the subsequent discovery of enhancer and silencer elements, it is thought that splicing mutations have been dramatically undercounted. Recent analysis predict about a third of all hereditary diseases to have a splicing component to them(Lim et al.; Sterne-Weiler et al.). For the current task of estimating the likelihood of causality for mutations identified within the families, we evaluated four mutations.

Two of these mutations fell within splice site regions of the titin (*TTN*) gene (-20 to +3 for the 3'ss; -3 to +6 for the 5'ss). These mutations were evaluated with maximum entropy models for splice sites which utilizes a Markov model to capture dependencies within different regions of the splice signal (Yeo and Burge, 2004). The first mutation is almost certain to disrupt splicing as it falls within the invariant AG of the three prime splice site (3'ss). The mutation calls in the 37th exon 30 of NM-133378 transcript where the AG of the 3'ss is mutated to AA, which reduces the MAXENT score 9.27 to 0.57. Exon 37 is likely recognized by exon definition as the exon is short (<250 nt) and the flanking introns are long (> 700 nt) (Robberson et al., 1990). For exons that are recognized under an exon definition model, the most common consequence of splicing is exon skipping. As Titin is an extremely large gene there is an ascertainment bias that may increase the likelihood of variants that false positives. However it is likely that this mutation is deleterious. The skipping of exon 37 (98 nt long) would create an out of frame transcript that starts in the 5' half of the transcript. This aberrant mRNA would likely be the target of nonsense mediated decay (NMD) or, if translate, a significantly disrupted protein.

The second mutation in the titin gene falls in the donor site of an upstream exon (exon 13). This mutation is an exonic G to C mutation at position +1 in a donor site with weak agreement with the consensus (GCGgtatat). This mutaiton reduces the MAXENT score from 3.9 to -10.3. Negative scores indicate the sequences with higher probability of occurring in the background than in the set of splice sites. The skipping event will not create a frameshift as exon 13 is a multiple of three but will result in the loss of 29 amino acids from the protein. As exon 13 is included in all full length transcript models for the titin is likely that this variant is disruptive to gene function.

The remaining mutations fell outside the splice signals. One was a *de novo* intronic variant 8 nt downstream of a donor site in the XPO4 gene. The second mutation was a synonymous variant in the CA3 gene. The strategy for evaluating these was to use ExonScan – a modified gene finding program that uses information thought to be available to the splicing machinery and discarding information like reading frame and conservation that is predictive but unlikely to be a physical basis of recognition (Wang et al., 2004). This *in silico* spliceosome spliced the wildtype and mutant versions of both sequences identically. In addition, positional distribution analysis was used to identify variants that disrupted splicing (Lim and Fairbrother; Lim et al.). As splicing signals are highly position dependant, variations that create new motifs that have a different genomic distribution than the existing motifs often reflect a disruption of a splicing signal. The threshold for a positive call (78 percentile) was not reached for either of these variations (XPO4 -66 percentile, CA3 – 64 percentile) (Lim and Fairbrother). In conclusion, the mutations in the TTN gene are the most compelling candidates for causal mutations in W1.

Analysis of microRNA gene MIR4268

Three target prediction tools were used to identify potential gene targets for the identified microRNA gene, MIR4268. Any target gene that appeared in two or more databases with a target prediction score of greater than 50%

was reviewed, in addition to all other candidate genes in the main review. Gene *IL1R1* with a rare variant in the proband (individual 2-1) is a potential target (but only appeared in one database with a moderate score) but the gene does not appear to match the observed phenotypes in family W2.

The potential gene targets were reviewed (Table 7) but no potential targets were obviously associated with the phenotypes in family W2. Gene MIR4268 has been confirmed through experimental analysis to be involved in neural cell precursors (Goff *et al*., PLoS One 2009), however there are no other known associations with electrophysiology or other cardiac phenotypes.

Table 7 A list of potential gene targets for microRNA gene MIR4268.

Gene	Gene Description
**DUSP6**	dual specificity phosphatase 6
**HOXB6**	homeobox B6
**SGSM2**	small G protein signaling modulator 2
**STAG1**	stromal antigen 1
**AGTR1**	angiotensin II receptor, type 1
**AP4E1**	adaptor-related protein complex 4, epsilon 1 subunit
**BCL11A**	B-cell CLL/lymphoma 11A (zinc finger protein)
**C17orf103**	chromosome 17 open reading frame 103
**CAMK2G**	calcium/calmodulin-dependent protein kinase II gamma
**CIAO1**	cytosolic iron-sulfur protein assembly 1
**COL6A6**	collagen, type VI, alpha 6
**CSGALNACT1**	chondroitin sulfate N-acetylgalactosaminyltransferase 1
**CSMD3**	CUB and Sushi multiple domains 3
**CXorf21**	chromosome X open reading frame 21
**DLX3**	distal-less homeobox 3
**DMP1**	dentin matrix acidic phosphoprotein 1
**DSC1**	desmocollin 1
**EFTUD2**	elongation factor Tu GTP binding domain containing 2
**ERP29**	endoplasmic reticulum protein 29
**ESRP2**	epithelial splicing regulatory protein 2
**FAM160B2**	family with sequence similarity 160, member B2
**FUT8**	fucosyltransferase 8 (alpha (1,6) fucosyltransferase)
**GIT2**	G protein-coupled receptor kinase interacting ArfGAP 2
**GTF2E2**	general transcription factor IIE, polypeptide 2, beta 34kDa
**KIF1C**	kinesin family member 1C
**MECOM**	MDS1 and EVI1 complex locus
**MPZL1**	myelin protein zero-like 1
**PHF3**	PHD finger protein 3
**PRRX1**	paired related homeobox 1
**RAD54B**	RAD54 homolog B (S. cerevisiae)
**RBM27**	RNA binding motif protein 27
**RHOBTB2**	Rho-related BTB domain containing 2
**RHOV**	ras homolog gene family, member V
**SC4MOL**	sterol-C4-methyl oxidase-like
**SCAF8**	SR-related CTD-associated factor 8
**SESN3**	sestrin 3
**SLC43A3**	solute carrier family 43, member 3
**SLC46A1**	solute carrier family 46 (folate transporter), member 1
**SNX1**	sorting nexin 1

**TAF12**	TAF12 RNA polymerase II, TATA box binding protein (TBP)-associated factor, 20kDa
**TMCO7**	transmembrane and coiled-coil domains 7
**UBE2K**	ubiquitin-conjugating enzyme E2K
**USP22**	ubiquitin specific peptidase 22

Random Expectation for the Number of de novo Variants in Each Gene

It is critically important to prioritize genes harboring *de novo* mutations in light of the expectation that *de novo* mutations in these genes would be observed in the general population. Genes with high mutation rate may result in spurious false positive findings.

Mutation rate in humans is highly context dependent with the largest increase in local mutation rate due to hypermutable CpG contexts. In addition, mutation rate varies along the genome. Finally, long genes would be expected to mutate more frequently than short genes.

We estimated the expected rate for each gene in the human genome based on tri-nucleotide context and the local mutation rate matrix in 100kb windows. The context dependent rates were derived using comparative genomics data (human-chimpanzee-baboon comparison). For each gene we estimated the probability that a *de novo* mutation will happen in this gene in a single generation. This model was used in a recent exome sequencing study (Neale *et al*., Nature 2012) and was validated using *de novo* coding mutations observed in pedigree sequencing.

Figure 12 Relative expected mutational rate per gene vs ranking. (A) In blue, genes with de-novo mutation in CLARITY and in red the rest of genes. (B) Quartile representation of 6 different genes with de-novo mutations in coding sequence.

(A) (B)

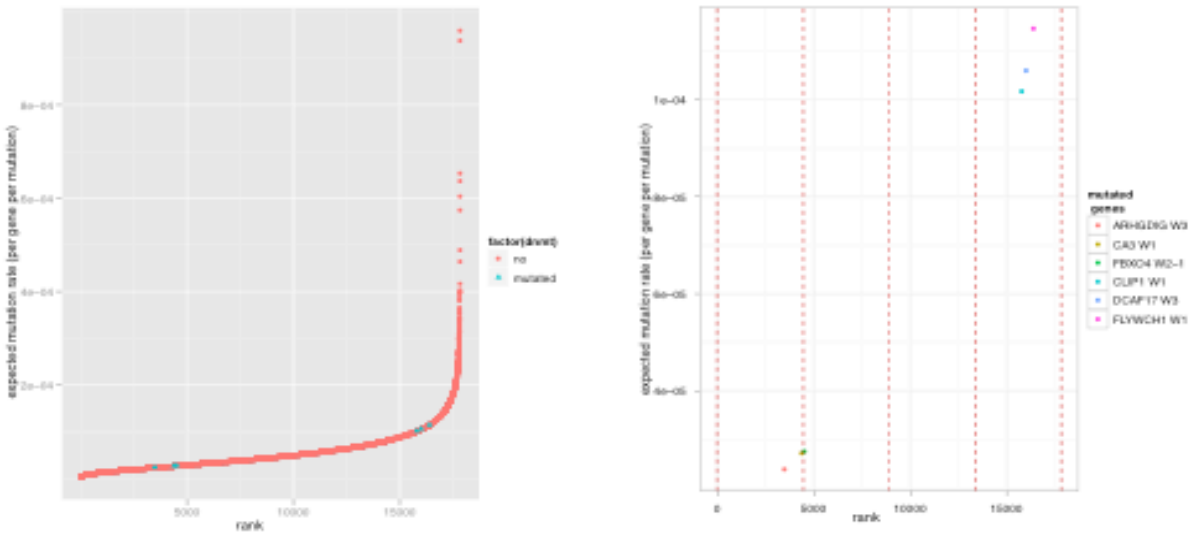

Random Expectation of Compound Heterozygotes in Each Gene

Similarly, it is critical to estimate the chance of false discovery, in the analysis of compound heterozygotes or homozygote variants. We sought to identify the expected numbers of variants we might identify in each gene, including compound heterozygotes, rare homozygous minor variants, and compound heterozygotes that are splicing related. For each class of variants described above, we used data from Europeans in the Exome Sequencing Project, to calculate the expected number of each variant type in each gene. We restricted our analysis to very rare variants, with MAF < 0.1%, in each gene (as we did in the variant candidate search).

For compound heterozygotes, we calculated the sum of all products of pairs of minor allele frequencies (q_i*q_j) for all variants within each gene that were missense, nonsense, or splicing related, and we include the top 10 genes from this analysis with the expected number of compound heterozygotes in each gene (Table 8). The expected number of compound heterozygotes in TTN is 0.086 in Europeans, ranked 1 of all genes, and the expected number in OBSCN is 0.009 in Europeans, ranked 3.

Table 8 Expected number of compound heterozygotes in each gene based on a simulation using European data from the Exome Sequencing Project. This analysis included all missense, nonsense and splicing related variants with MAF < 0.1% in each gene.

Gene	Expected Comp. Hets. in Europeans	Rank
**TTN**	0.08642718	1
**MUC16**	0.021574909	2
**OBSCN**	0.009034693	3
**AHNAK2**	0.008961462	4
**FLG**	0.005955055	5
**MUC5B**	0.004303622	6
**SYNE1**	0.004232804	7
**PLEC**	0.00379456	8
**NEB**	0.003625365	9
**DNAH17**	0.003329175	10

We conducted the same analysis restricted to only splicing related variants, restricted to only nonsense variants, and restricted to all nonsense and splicing variants. When only considering splice-related variants that are MAF < 0.1%, the probability that these will form a pair-wise compound heterozygote is 3.26E-05 for TTN, and 1.74E-05 for OBSCN in Europeans (Table 9).

Table 9 Expected number of compound heterozygotes in each gene based on a simulation using European data from the Exome Sequencing Project. This analysis included only splicing related variants with MAF < 0.1% in each gene.

Gene	Expected Comp. Hets. in Europeans	Rank
**TTN**	3.26E-05	1
**OBSCN**	1.74E-05	2
**PLB1**	1.38E-05	3
**CUBN**	7.77E-06	4
**SSPO**	7.77E-06	5
**NEB**	7.77E-06	6
**MYH4**	6.54E-06	7
**COL22A1**	6.54E-06	8
**ZAN**	6.37E-06	9
**SYNE1**	5.39E-06	10

For rare homozygous minor variants, we calculated the Hardy-Weinberg homozygous minor probability (q^2) from the ESP minor allele frequency and summed all rare homozygous minor variant frequencies from each gene to find the expected number of homozygous minor variants in each gene (Table 10).

Table 10 Expected number of homozygotes in each gene based on a simulation using European data from the Exome Sequencing Project. This analysis included only splicing related variants with MAF < 0.1% in each gene.

Gene	Expected Homozygous Minor Vars in Europeans	Rank
**TTN**	1.71E-04	1
**MUC16**	1.00E-04	2
**OBSCN**	6.76E-05	3
**AHNAK2**	6.54E-05	4
**SSPO**	5.64E-05	5
**DNAH17**	5.15E-05	6
**MUC5B**	4.67E-05	7
**SYNE1**	4.15E-05	8
**PLEC**	4.12E-05	9
**RNF213**	3.90E-05	10

Summary of Case 1 (W1)

The absence of family history could suggest either de novo occurrence of a dominant variant or recessive inheritance and centronuclear myopathy has been described by both dominant and recessive inheritance patterns. Therefore, both modes of inheritance were considered. We report variants in two genes as possible candidates ranked in the following order.

The proband is likely compound heterozygous for two rare predicted splice site variants in Titin (TTN) (assuming a recessive mode of inheritance). Titin plays a key role in muscle assembly, force transmission at the Z line, and maintenance of resting tension in the I band region (Itoh-Satoh et al., 2002). Titin has been previously suggested as a candidate in multiple myopathies. The potential role of this candidate gene is supported by expression data and protein-protein interaction data. We do realize that TTN is an extremely long gene and spurious findings in this gene by chance are possible; in particular compound heterozygous missense changes are particularly frequent. However, we estimate the chance to detect a TTN compound heterozygote for nonsense or splice variants, as observed here, at $8x10^{-5}$ in the general population.

The proband likely carries a de novo missense change in *CLIP1*. The *CLIP1* (*CLIP170* isoform) is strongly expressed in muscle (Griparic & Keller, BBA 1998) and is involved in microtubule dynamics (Mishima et al., PNAS 2007).

Our analysis confirmed strong expression in muscle. The missense change is predicted to be damaging by computational analysis.

Additional variants identified in the upstream analysis, but were not supported by the downstream analysis, include: de novo missense change in FLYWCH1; de novo synonymous change in CA3 without clear support for an impact on splicing; a homozygous rare missense variant in TRIM50; and compound heterozygous missense variants in MYO5B.

Summary of Case 2 (W2)

The clinical assessment of the pedigree suggested that the conduction phenotype is likely to be shared by all affected pedigree members. The resolved ventricular mass in the proband was thought to more likely be a separate phenotype specific to the first trio. The structural heart defects seen in the second trio were thought to more likely be an independent phenotype not relevant to the proband.

For the conduction defect, we identified a single strong candidate. A very rare missense change in *TRPM4* segregates with the conduction defect in the pedigree. *TRPM4* encodes a transient receptor potential cation channel. Missense variants in the TRPM4 gene have been previously reported in over 10 families with dominant cardiac conductance defects (Kruse *et al.*, 2009, Liu *et al.* 2010, Stallmeyer *et al.* 2012). The Val168Glu variant affects a

residue in the N-terminal domain of the *TRPM4* channel, a domain commonly mutated in patients with *TRPM4*-related conduction defects (Stallmeyer *et al.*, 2012). Missense variants in the *TRPM4* gene have been shown to result in impaired endocytosis and elevated *TRPM4* channel density at the cell surface (Kruse 2009, Liu 2010). Study by Stallmeyer (2012) identified patients with both RBBB and AV block similarly to pedigree W2. One pedigree in Liu *et al.*, (2010) includes individuals with phenotypic features characteristic to both trios in W2. Although variant Val168Glu is predicted to be benign using computational methods, manual review triggered a comprehensive comparative genomics analysis as described above. The phylogenetic analysis is consistent with the possibility that Val168Glu is a gain-of-function variant (and one other disease mutation shows a similar phylogenetic pattern).

The resolved ventricular mass phenotype in the proband is a clinical scenario seen in tuberous sclerosis complex, however we also recognize that it could be non-genetic (e.g thrombus); we did not find any potential candidates in the established candidate genes TSC1 and TSC2. Considering an alternative genetic etiology, we identified one rare homozygous variant in *PRKG1*. This Tyr474Phe variant in *PRKG1* has been seen in 0.09% of European American chromosomes in an ethnically-matched broad population (ESP sample). The variant was computationally predicted as possibly damaging. *PRKG1* is not strongly expressed in adult heart. In addition, selective postnatal ablation of *PRKG1* (*cGK1*) in vascular smooth muscle cells in mice inhibits atherogenesis, including reduced rate of aortic cell proliferation (Wolfsgruber 2003). Therefore, considering a possible gain of function conferred by this variant could render aortic cells unable to proliferate properly and lead to slightly dilated aorta during development (2-1). Also, due to being a negative regulator of cardiac myocyte hypertrophy (Fiedler 2002) this variant may also explain the resolution of hypertrophic mass in right ventricle (if it were a muscle originated or angioma nature) (2-1).

We also identified a de novo missense change in FXBO4, though additional support for this variant is relatively weak. The upstream analysis also identified a *de novo* intronic change in XPO4. The XPO4 mutation was found to have a low predicted impact on splicing in the downstream analysis. We also considered the possibility of an inherited dominant variant in mTOR pathway but did not identify suitable candidates.

There are four potential candidates to explain the structural defect in the second trio (not involving the proband) ranked in the following order.

A nonsense variant in *KCNH8* (*ELK1*) not observed in ESP. This nonsense variant leads to a premature termination codon at position 959, which is predicted to lead to a truncated or absent protein. *KCNH8* has been reported to be involved in ventricular muscle proliferation in response to cellular stresses induced by phenylephrine (Babu 2000) or ischemia (Hwang 2001). Also, it mediates antiproliferative effects of estrogens in rat aorta smooth muscle cells (Hwang 2001). KCNH8 has also been shown to mediate TNF-alpha induced human aortic smooth muscle cell proliferation and migration (Kang 2012). Collectively, these in vivo and in vitro studies emphasize the essential role of *KCNH8* in human ventricular muscle proliferative response to stress as well as aorta smooth muscle proliferation and migration. The lack of a functional KCNH8 protein in embryonic life may therefore lead to the defects such as hypoplastic right heart (2-4) and coarctation (2-6).

A missense change in SMYD1. SMYD1 is highly expressed in heart and loss-of-function of SMYD1 results in right ventricular hypoplasia in mice. The missense variant has been confidently predicted to be damaging. 6 out of 6800 alleles are observed in the ESP cohort.

A rare missense variant in *NEBL*. The Glu409Lys variant is absent in the ESP cohort. *NEBL* is highly expressed in heart. Missense variants in the nebulette gene have been identified in patients with dilated cardiomyopathy (DCM) and endocardial fibroelastosis which is characterized by proliferation of fibrous and elastic tissue. Overexpression of either of these four missense NEBL variants transgenic mice have induced development of DCM and severe heart failure. The mutations have shown to modulate distribution of nebulette in the sarcomere and the Z-disks (Purevjav 2010). In addition, *NEBL* has been shown to organize actin filaments into bundles and interact directly with the Z-disc protein alpha-actinin, therefore functioning as a scaffolding and actin filament organizing protein within striated muscle Z-discs in chick and mouse cardiomyocytes (Zieseniss 2008). These findings are consistent with a potential role of *NEBL* in heart development, therefore its possible contribution to hypoplastic right heart (2-4).

A missense variant in *TNC*. The Gly792Cys variant has not been reported in the literature. The variant has been seen in 0.07% of European American chromosomes in ESP. TNC is involved in local vascular remodeling by virtue of increasing migratory capacity of cardiac microvascular endothelial cells (Ballard 2006) as well as tissue remodeling by modulating the attachment of cardiomyocytes to connective tissue, enhancing migration and differentiation of myofibroblasts and inducing matrix metalloproteinases (Imanaka-Yoshida 2004). In addition, TNC is involved in neointimal proliferation in arterial wall (Golledge 2011). High tissue levels of TNC have also been reported within a range of other cardiovascular pathologies, including carotid atherosclerosis, pulmonary artery hypertension (Jones 1997), abdominal aortic aneurysm (Kimura 2011), renal access graft intimal hyperplasia, renal transplant vasculopathy, and varicose veins. In summary, *TNC* may play a role in pathologic conditions such as coarctation (2-6), pulmonary stenosis (2-6,and 2-4, with a potential anticipation) and hypoplastic right heart (2-4) observed in the probands.

A missense change in *SMYD1*. The Arg398Gln variant has been identified in 0.07% of European American chromosomes in ESP and 0.02% of African American chromosomes. This variant is predicted as probably damaging. *SMYD1* is strongly expressed in heart. Furthermore, although homozygous loss-of-function of *SMYD1* results in right ventricular hypoplasia in mice (Gottlieb 2002) and absence of heart beat in zebrafish despite normal heart development (Tan 2006 and Just 2011) there are no reports with respect to the deleterious impact of any missense variant in *SMYD1* in cardiac disease.

We also identified a potential stop loss variant in *IRL1L1*.

Summary of Case 3 (W3)

The proband is likely compound heterozygous for rare missense variants in *OBSCN* (Obscurin). *OBSCN* is a giant sarcomeric protein, and is involved in myofibrillogenesis (Young et al., J Cell Biol 2001). The potential role in nemaline myopathy is supported by strong and specific expression in muscle. Obscurin belongs to the same family as nebulin, one of the known genes involved in nemaline myopathy. We predict that missense mutations are highly damaging. Given the length of the gene, we estimated that there is a relatively high frequency of individuals with compound heterozygous missense variants in the general population (f=0.009, i.e. nearly 1% of the population are expected to harbor such a genotypic combination by chance).

Additional variants there were identified in the upstream analysis but were not supported by the downstream analysis include missense variants in *USP6* (with relatively low variant call quality); a *de novo* missense variant in *ARHGDID*; and a *de novo* synonymous variant in *DCAF17*.

Preparing Clinical Reports

Our approach to clinical reporting for genomic sequencing is to include any variants that have a strong or reasonable basis for being considered etiologic for the primary indication for testing. Those genes with variants identified by the described filters that were not strongly considered are mentioned briefly whereas those variants that remain as known or probable causes of the phenotype are described more thoroughly on the report. In these cases, we did not pursue the reporting of any secondary findings as it was not considered part of the requested challenge. Also, given the complexity or reporting genomic sequencing data, we feel that accompaniment of clinical guidance from a practicing physician familiar with genetics is critical. As such, each laboratory report is followed by a clinical guidance report.

Appendix I

Phase by Transmission

Phase by Transmission is a GATK module that computes the joint posterior probability of each possible genotype combination at a bi-allelic site in a parents-child trio. It uses the individual genotype likelihoods for each of the trio members and the per-generation mutation rate as input. Modern SNP callers such as the GATK Unified Genotyper can compute the individual genotype likelihoods.

In humans the mutation rate between generations is estimated to be in the order of 10^{-8} per base per generation. Based on the mutation rate μ, a prior can be derived for each possible genotype combination in a trio:

$$P_C = \begin{cases} 1 - 10\mu - 2\mu^2\text{, if the combination follows Mendel's laws} \\ \mu\text{, if the combination implies 1 mutation} \\ \mu^2\text{, if the combination implies 2 mutations} \end{cases}$$

Using the combination priors above, the joint posterior probability for each genotype combination is computed as follows:

$$P(D|G_M, G_F, G_C) = P_C \cdot P(D|G_M) \cdot P(D|G_F) \cdot P(D|G_C)$$

Phase by Transmission then assigns the most likely genotype combination to the trio. If the most likely combination implies a *de novo* mutation, it is reported as such. Moreover a confidence pred-scaled quality score is associated with each genotype combination.

Note regarding the SOLiD data

This method has shown excellent results in Illumina data for *de novo* mutation detection, both on deep coverage exome (60x) and medium coverage (12x) whole-genome with validation rates >85% and >62% respectively.

Using the SOLiD data provided for the CLARITY challenge however the results were quite poor: for family W1 24 *de novo* mutations were called, out of which only 1 was not situated in a poorly mapped region or coming from duplicated reads. This behavior most probably comes from the GATK Unified Genotyper genotype likelihood calculation that assumes that the base quality scores are properly calibrated and duplicate reads marked. Even after using tools for marking duplicate reads (Picard MarkDuplicates) and recalibration of the base quality score using GATK BQSR, the results did not improve. This probably is the result of an incompatibility between the BAM format expected by the GATK and provided by SOLiD for the CLARITY challenge but turned out to be a major hurdle for the use of some of our standard tools including GATK Phase by Transmission.

Figure 1 Calculation of posterior probabilities

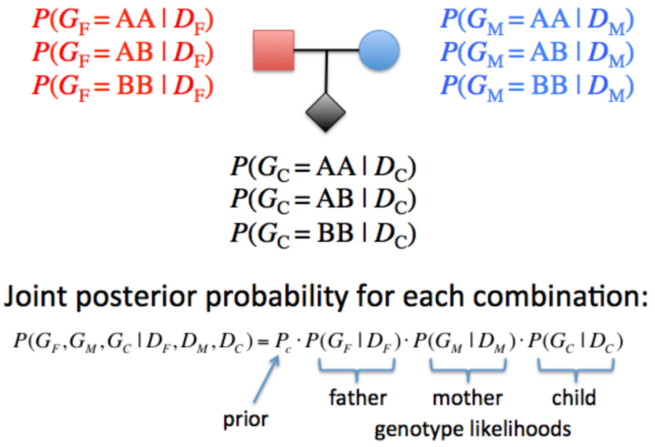

Supplement: Additional file 1 — The complete entry from the Brigham and Woman’s Team containing seven PDF files, six PNG image files, and one XLS table. [file gb-2014-15-3-r53-S1.zip › Additional_file_1/FINAL_Long_Science.pdf]
