# Supplementary material for: An international effort towards developing standards for best practices in analysis, interpretation and reporting of clinical genome sequencing results in the CLARITY Challenge
Source: Genome Biol. 2014 Mar 25;15(3):R53. doi: 10.1186/gb-2014-15-3-r53 (PMC4073084; doi:10.1186/gb-2014-15-3-r53)
Supplement: Additional file 1 — The complete entry from the Brigham and Woman’s Team containing seven PDF files, six PNG image files, and one XLS table. [file gb-2014-15-3-r53-S1.zip › Additional_file_1/W1_final_clinical.pdf]

PARTNERS® HEALTHCARE | CENTER FOR PERSONALIZED GENETIC MEDICINE

A teaching affiliate of: HARVARD MEDICAL SCHOOL

Laboratory for Molecular Medicine

65 Landsdowne St, Cambridge, MA 02139
Tel: 617-768-8500 Fax: 617-768-8513
pcpgm.partners.org/lmm

**Patient Name:**	W1	**Specimen type:**	DNA from peripheral blood
**DOB:**	10 yr	**Date specimen obtained:**	
**Lab Accession:**		**Date specimen received:**	05/01/2012
**Pedigree #:**		**Referring physician**	
**Gender:**	Male	**Referring facility**	
**Race:**	White	**Referring facility MRN:**	

TEST PERFORMED – Exome and Genome Sequencing

INDICATION FOR TEST – Centronuclear myopathy

RESULT: Positive - Variants were identified that are likely to explain the reported phenotype

DNA VARIANTS:

**Gene**	**Variant**	**Classification**	**Parental Inheritance**
TTN	Het c.37112-1G>A (p.?)	Likely Pathogenic	Maternal
TTN	Het c.32854G>C (p.?)	Uncertain Significance – Likely Pathogenic	Paternal
CLIP1	Het c.3258G>T (p.Gln1086His)	Uncertain Significance	*de novo*

INTERPRETATION SUMMARY: Candidate variants from genome and exome sequencing were analyzed with consideration for possible *de novo* or recessive causes of a rare Mendelian disease. No rare variants were identified in genes known to be associated with centronuclear myopathy (*DNM2*, *MYF6*, *MTM1*, *BIN1*). Insufficient sequencing data was obtained for 8% of the coding regions for these genes, and therefore the presence of a rare or pathogenic variant within them cannot be fully excluded.

Analysis of additional candidate variants revealed one gene, *TTN*, as the likely explanation for this individual's phenotype. Two variants, one highly likely and one likely to have a functional effect, were identified, one inherited from each parent. See detailed variant interpretations below. The *TTN* gene is strongly associated with dilated cardiomyopathy (Herman 2012), and has also been linked to skeletal muscle disorders, including tibial muscular dystrophy, hereditary myopathy with early respiratory failure, and early-onset myopathy with fatal cardiomyopathy with both recessive and dominant modes of inheritance described (Carmignacet al. 2007, Hackmanet al. 2002, Ohlssonet al.2012, Pfefferet al. 2012). In addition, there is recent evidence linking *TTN* to centronuclear myopathy, the indicated phenotype of this individual (Ceyhan et al. 2012). It should be noted that the *TTN* gene is extremely large and therefore, the chance of an individual carrying more than one rare variant is higher than for any other gene in the genome. However, the chance of observing the combination of variants present in this individual is low (estimated at $8x10^{-5}$), further supporting that they are likely responsible for this individual's disease.

In addition, a variant of unknown significance was identified in the *CLIP1* gene, a *de novo* missense variants not present in either of the parent. The *CLIP1* gene is specifically expressed in muscle, and may play a role in microtubule dynamics (Griparic and Keller 1998, Mishima et al., 2007). *De novo* occurrence and tissue specific expression support a causative or contributory role, though additional data is needed to fully establish the pathogenicity of this variant.

Several additional variants were considered but were felt to be unlikely causes of this individual's presentation. Two variants, a synonymous variant in *CA3* and a missense variant in *FLYWCH1*, were observed as *de novo* events but were considered unlikely causes based upon type of change and/or absence any other plausible disease or functional data that that could implicate the genes in the disease. Rare compound heterozygous variants in the *MYO5B* gene and a rare homozygous variant in the *TRIM50* gene were also considered but were excluded based upon computational predictions and a lack of a plausible biological or functional role in disease.

INDIVIDUAL VARIANT INTERPRETATIONS:

37112-1G>A in *TTN* – Likely Pathogenic
The 37112-1G>A variant in *TTN* has not been reported in the literature nor previously identified by our laboratory. This variant occurs in the invariant region (+/- 1,2) of the splice consensus sequence and is predicted to cause altered splicing, most likely leading to skipping of exon 37. Removal of this exon would lead to a frameshift and most likely cause nonsense-mediated decay, leading to an absent protein. If the exon is not skipped this variant would result in a missense change Val10952Leu. In addition, this variant has not been identified in large and broad European American and African American populations by the NHLBI Exome Sequencing Project (http://evs.gs.washington.edu/EVS). In summary, this data supports a pathogenic interpretation of this variant.

c.32854G>C in *TTN* - Uncertain Significance Likely Pathogenic
This variant in *TTN* has not been reported in the literature nor previously identified by our laboratory. This variant has not been identified in large and broad European American and African American populations by the NHLBI Exome Sequencing Project (http://evs.gs.washington.edu/EVS). This variant is located in the 5' splice region, in the last base of exon 13, and computational tools suggest a likely impact to splicing. If splicing were affected, exon skipping would most likely occur, resulting in the loss of 29 amino acids from the protein. As exon 13 is included in all full length transcript models for *TTN*, it is likely that the loss of these 29 amino acids would be disruptive to protein function. In summary, the available data suggests a more likely pathogenic role for this variant; however, additional information is needed to fully assess the clinical significance of this variant.

Gln1086His *in CLIP1* - Uncertain Significance
The Gln1086His variant in CLIP1 has not been reported in the literature nor previously identified by our laboratory. In addition, this variant has not been identified in large and broad ethnically-matched populations by NHLBI Exome Sequencing Project (http://evs.gs.washington.edu/EVS). Computational analyses provide some support for an impact to the protein. *CLIP1* (*CLIP170* isoform) is specifically expressed in muscle (Griparic and Keller 1998) and is involved in microtubule dynamics (Mishima et al., 2007). Furthermore, this variant arose *de novo* in this individual. However, in the absence of other data to implicate this variant in the patient's disease, the clinical significance of this variant remains unknown.

RECOMMENDATIONS:
Please note, a DNA sample was unavailable to confirm the technical results of this test. Therefore, we recommend an independent confirmation of all clinically relevant findings before medical action is considered.

Genetic counseling is recommended for this individual and their family. For assistance in locating nearby genetic counseling services please contact the laboratory at 123-456-7890.

A medical provider can request reanalysis of the exome data, and this is recommended on an annual basis. Data from this exome sequencing analysis can be reassessed for the presence of any variants that may be newly linked to established genes or to newly characterized genes and/or disorders identified since the date of this report that could be associated with the patient's phenotype, based on currently available scientific information. A charge may apply for reanalysis. Please contact the laboratory for more information at the time reanalysis is requested.

TEST METHOD: Raw sequencing data was provided for the exome (on the SOLiD platform) and genome (on the CGI platform) for the proband and parental samples. Bioinformatic analyses were performed to examine potential explanations for disease in the proband**.** These analyses included variant calling, variant annotation, ancestry analysis, analysis of allele frequencies in the ancestry-matched population, analysis of gene expression, analysis or protein-protein interactions data, computational predictions of the effect of missense mutations, computational predictions of the effect on splicing and estimation of gene-specific background probabilities of de novo mutations, rare homozygote and compound heterozygote variants.

LIMITATIONS: Variants have not been confirmed by an independent analysis and could represent technical artifacts. Some types of genetic abnormalities may not be detectable with the technologies performed by this exome analysis test. It is possible that the genomic region where a disease causing mutation exists in the proband was not captured using the current technologies and therefore was not detected. Additionally, it is possible that a particular genetic abnormality may not be recognized as the underlying cause of the genetic disorder due to incomplete scientific knowledge about the function of all genes in the human genome and the impact of variants in those genes. Only variants in genes associated with the medical condition, or thought to potentially be clinically relevant for the proband's medical condition, are reported here.

REFERENCES:
1. Herman DS, Lam L, Taylor MR, Wang L, Teekakirikul P, Christodoulou D, Conner L, DePalma SR, McDonough B, Sparks E, Teodorescu DL, Cirino AL, Banner NR, Pennell DJ, Graw S, Merlo M, Di Lenarda A, Sinagra G, Bos JM,

Ackerman MJ, Mitchell RN, Murry CE, Lakdawala NK, Ho CY, Barton PJ, Cook SA, Mestroni L, Seidman JG, Seidman CE. Truncations of titin causing dilated cardiomyopathy. N Engl J Med. 2012 Feb 16;366(7):619-28.

2. Carmignac V, Salih MA, Quijano-Roy S, Marchand S, Al Rayess MM, Mukhtar MM, Urtizberea JA, Labeit S, Guicheney P, Leturcq F, Gautel M, Fardeau M, Campbell KP, Richard I, Estournet B, Ferreiro A. C-terminal titin deletions cause a novel early-onset myopathy with fatal cardiomyopathy. Ann Neurol. 2007 Apr;61(4):340-51.

3. Hackman P, Vihola A, Haravuori H, Marchand S, Sarparanta J, De Seze J, Labeit S, Witt C, Peltonen L, Richard I, Udd B. Tibial muscular dystrophy is a titinopathy caused by mutations in TTN, the gene encoding the giant skeletal-muscle protein titin. Am J Hum Genet. 2002 Sep;71(3):492-500. Epub 2002 Jul 26.

4. Ohlsson M, Hedberg C, Brådvik B, Lindberg C, Tajsharghi H, Danielsson O, Melberg A, Udd B, Martinsson T, Oldfors A. Hereditary myopathy with early respiratory failure associated with a mutation in A-band titin. Brain. 2012 Jun;135(Pt 6):1682-94. Epub 2012 May 9

5.Pfeffer G, Elliott HR, Griffin H, Barresi R, Miller J, Marsh J, Evilä A, Vihola A, Hackman P, Straub V, Dick DJ, Horvath R, Santibanez-Koref M, Udd B, Chinnery PF. Titin mutation segregates with hereditary myopathy with early respiratory failure. Brain. 2012 Jun;135(Pt 6):1695-713. Epub 2012 May 9.

6. Agrawal PB, Schmitz K, DeChene ET, Ceyhan Ö, Mercier M, Viola M, Markianos K. Complete genetic analysis by whole exome sequencing of a cohort with centronuclear myopathy identifies titin gene mutations. Neuromuscular Disorders Volume 22, Issue 9 , Page 840, October 2012.

7. Griparic L, Keller TC. Identification and expression of two novel CLIP-170/Restin isoforms expressed predominantly in muscle. Biochim Biophys Acta. 1998 Oct 21;1405(1-3):35-46.

8. Mishima M, Maesaki R, Kasa M, Watanabe T, Fukata M, Kaibuchi K, Hakoshima T. Structural basis for tubulin recognition by cytoplasmic linker protein 170 and its autoinhibition. Proc Natl Acad Sci U S A. 2007 Jun 19;104(25):10346-51. Epub 2007 Jun 11.

Report draft provided by Matthew Lebo, PhD, FACMG on Sep 27th, 2012.
Report approved by Heidi L. Rehm, PhD, FACMG on Sep 27th, 2012.

BWH Resource Center
for Clinical Genomics
41 Avenue Louis Pasteur, Suite 309
Tel: 617-264-5833 Fax: 617-264-3018

**TEST PERFORMED**	Exome and Genome Sequencing
**INDICATION FOR TEST**	Centronuclear Myopathy (CNM) in 1-1

CLINICAL GUIDANCE *

A. **Diagnostic Certainty of CNM in Affected Proband** – This diagnosis was reported to have been made in this case on the basis of the defining histo-pathological finding and confirmed by a second pathology opinion; there is no reason identified that raises doubt about the diagnosis. It is noted that this histo-pathological diagnosis has a genetically heterogeneous etiology, and has a broad clinical spectrum.
CLINICAL GENOMICS ACTION TO CONSIDER: None

B. **Diagnostic Certainty of a Lack of CNM in First Degree Relatives** – Individuals with CNM typically have onset of muscle weakness in the first or second decade in life that is slowly progressive. In the reported clinical data there is no suggestion of skeletal myopathy in either parent. Although it is known that one form of the disease, namely CNMX (X-linked centronuclear myopathy) has been associated with mild clinical disease, *MTM1* is the only known gene associated with this X-linked disease and it was not found to have a mutation. Therefore no specific medical workup of parents for CNM is suggested for consideration at this time.
CLINICAL GENOMICS ACTION TO CONSIDER: None

C. **Follow-up Clinical Testing Based on Genomic Findings** – Some individuals with one *TTN* variant are known to be at increased risk for adult onset dilated cardiomyopathy. The parents (1-2, 1-3) are heterozygous for *TTN* variants and should be referred for evaluation for this condition. Additional at-risk relatives include the patient's aunts, uncles, and grandparents. Additional at-risk individuals should discuss this risk with the healthcare provider; genetic counseling is recommended.
CLINICAL GENOMICS ACTION TO CONSIDER: Clinical evaluation of parents for cardiomyopathy is indicated.

D. **Follow-up DNA Sequencing Based on Reported Family History** – the testing of the trio has achieved a probable diagnostic result. The pedigree provided does not suggest other individuals may be at risk for CNM, therefore no further CNM diagnostic testing is indicated. The pattern of mutations in *TTN* and *CLIP1* do not suggest that there would be utility to additional testing for evidence of segregation.
CLINICAL GENOMICS ACTION TO CONSIDER: None for parents. Consider evaluation of grandparents, aunts and uncles (on both sides) for *TTN* mutations that could confer risk of dilated cardiomyopathy.

E. **Additional follow-up to Improve Confidence in the Putative Genomic Diagnosis** – this test has not establised a causative relationship between either *TTN* or *CLIP1* and Centronuclear Myopathy. The association observed here will be strengthened by: [a] additional cases in unrelated patients, [b] in vitro studies.
CLINICAL GENOMICS BASED ACTION TO CONSIDER: In vitro splicing assays may supplement interpretation of *TTN* variants.

F. **Genomic Implications for Prognosis in Proband** – no known data

Clinical Guidance draft provided by: **Monica A. Giovanni, MS, CGC on Sep 28th, 2012.**

Clinical Guidance approved by: **Michael F. Murray, MD, FACMG on Sep 28th, 2012.**

** This guidance does not replace the clinical judgment of the patient's health care team. The guidance will be of limited value and may in fact be incorrect in cases where the data provided is incomplete or inaccurate. Periodic re-evaluation and updating of clinical guidance is recommended.*
