# Supplementary material for: An international effort towards developing standards for best practices in analysis, interpretation and reporting of clinical genome sequencing results in the CLARITY Challenge
Source: Genome Biol. 2014 Mar 25;15(3):R53. doi: 10.1186/gb-2014-15-3-r53 (PMC4073084; doi:10.1186/gb-2014-15-3-r53)
Supplement: Additional file 1 — The complete entry from the Brigham and Woman’s Team containing seven PDF files, six PNG image files, and one XLS table. [file gb-2014-15-3-r53-S1.zip › Additional_file_1/W2_final_clinical.pdf]

PARTNERS® HEALTHCARE | CENTER FOR PERSONALIZED GENETIC MEDICINE

A teaching affiliate of: HARVARD MEDICAL SCHOOL

Laboratory for Molecular Medicine

65 Landsdowne St, Cambridge, MA 02139
Tel: 617-768-8500 Fax: 617-768-8513
pcpgm.partners.org/lmm

**Patient Name:**	W2	**Specimen type:**	Sequencing data
**DOB:**	5 yr	**Date specimen obtained:**	
**Lab Accession:**		**Date specimen received:**	
**Pedigree #:**		**Referring physician**	CLARITY Challenge
**Gender:**	Female	**Referring facility**	
**Race:**	White	**Referring facility MRN:**	

TEST PERFORMED – Exome and Genome Sequencing

INDICATION FOR TEST- Right bundle branch block and resolved right ventricular mass

RESULT: A variant in a gene with an established role in cardiac conduction defects was identified

DNA VARIANTS:

Gene	Variant	Classification	Parental Inheritance
TRPM4	Het c.503T>A (p.Val168Glu)	Uncertain Significance – Likely Pathogenic	Maternal
PRKG1	Hom c.1421T>A (p.Tyr474Phe)	Uncertain Significance	Homozygous

INTERPRETATION SUMMARY: This individual is reported to have a clinical diagnosis and family history of RBBB, as well as a resolved right ventricular mass in the absence of a family history. Candidate variants from genome and exome sequencing were therefore analyzed with consideration for a dominant cardiac conductance disorder and related or independent causes of the other cardiac manifestations in this individual and her family members. Our analyses revealed a variant in the TRPM4 gene that segregates with disease in all four affected individuals examined and provides a plausible explanation for the cardiac conduction phenotype. *TRPM4* encodes a transient receptor potential cation channel. Missense variants in this gene have been previously described in several families with cardiac conduction blocks and functional studies indicate that these variants, particularly those in the N-terminal domain, lead to gain-of-function defects (Kruse 2009, Liu 2010, Stallmeyer 2012). The Val168Glu variant in *TRPM4* identified in this individual occurs in the N-terminal domain. Given the variant's consistent segregation with disease, its rarity in the general population, and its localization to the N-terminus, the Val168Glu variant represents a plausible explanation for the cardiac conduction disorder observed in this individual.

The resolved ventricular mass phenotype in this individual is a clinical scenario seen in tuberous sclerosis complex; however. there could also be a non-genetic origin (e.g thrombus). We did not find any potential candidates in the established candidate genes *TSC1* and *TSC2*. Insufficient sequencing data was obtained for 10% of the coding regions for these genes, and therefore the presence of a rare or pathogenic variant within them cannot be fully excluded. Considering an alternative genetic etiology, we identified one rare homozygous variant in *PRKG1*. *PRKG1* is not strongly expressed in the adult heart, but several functional studies have implicated this gene in aortic and cardiac functions. Selective postnatal ablation of *PRKG1* in vascular smooth muscle cells in mice inhibits atherogenesis, suggesting a gain of function variant could render aortic cells unable to proliferate properly and lead to slightly dilated aorta during development. Also, due to being a negative regulator of cardiac myocyte hypertrophy (Fiedler 2002) this variant may also explain the resolution of hypertrophic mass in right ventricle. However, at this time there is not enough data to implicate the *PRKG1* gene or the Tyr474Phe variant in this individual's resolved right ventricular mass.

Two variants, a missense variant in *FXBO4* and an intronic variant in *XPO4*, were observed as *de novo* variants but were considered unlikely causes of the mass or conduction defect based upon weak cardiac gene expression, splicing, and/or computational analyses. A rare Copy Number Variant (CNV) containing microRNA gene *MIR4268* segregates with the conduction defect in pedigree members with whole genome sequencing data available, though this CNV was considered an unlikely cause of the mass or conduction defect.

Members of the individual's extended family manifest a separate cardiac structural defect. We concluded that this defect is unrelated to the disease of the patient, and may have a separate genetic etiology. Four potential candidate genes were identified that may be involved in this structural defect.

INDIVIDUAL VARIANT INTERPRETATIONS:

Val168Glu in *TRPM4* – Likely Pathogenic

The Val168Glu variant in TRPM4 has not been previously identified by our laboratory or in the literature. This variant has not been identified in large and broad populations by the NHLBI Exome Sequencing Project (http://evs.gs.washington.edu/EVS/). Computational analyses suggest that the Val168Glu variant may not impact the protein, though this information is not predictive to rule out pathogenicity and does not rule out a potential gain of function mechanism. Missense variants in the TRPM4 gene have been previously reported in over 10 families with dominant cardiac conduction defects (Kruse 2009, Liu 2010, Stallmeyer 2012) and functional studies have shown certain missense variants result in impaired endocytosis and elevated TRPM4 channel density at the cell surface. TRPM4 encodes a transient receptor potential cation channel, and the Val168Glu variant affects a residue in the N-terminal domain of the TRPM4 channel, a domain commonly mutated in patients with TRPM4-related conduction defects (Stallmeyer 2012). In summary, the available data suggests that the Val168Glu variant is likely pathogenic, though additional studies are required to fully establish its clinical significance.

Tyr474Phe in *PRKG1*– Uncertain Significance

The Tyr474Phe variant in PRKG1 has not been reported in the literature nor previously identified by our laboratory, but has been detected in 0.09% (8/8600) of European American chromosomes in an ethnically-matched broad population by the NHLBI Exome Sequencing Project (http://evs.gs.washington.edu/EVS; rs149710600). Computational analyses suggest that the variant may impact the protein, though this information is not predictive enough to determine pathogenicity. In addition, several functional studies support a role of the *PRKG1* gene in both cardiac and aortic funtion. First, selective postnatal ablation of *PRKG1* (*cGK1*) in vascular smooth muscle cells in mice leads to a reduced rate of aortic cell proliferation and inhibits atherogenesis (Wolfsgruber 2003). Second, this gene was shown to act as a negative regulator of cardiac myocyte hypertrophy (Fiedler 2002), raising the possibility that this variant may explain the resolution of hypertrophic mass in right ventricle. However, in the absence of other data to implicate this variant in the patient's disease, its clinical significance remains unknown.

RECOMMENDATIONS:

Please note, a DNA sample was unavailable to confirm the technical results of this test. Therefore, we recommend an independent confirmation of all clinically relevant findings before medical action is considered.

Genetic counseling is recommended for this individual and their family. For assistance in locating nearby genetic counseling services please contact the laboratory at 123-456-7890.

A medical provider can request reanalysis of the exome data, and this is recommended on an annual basis. Data from this exome sequencing analysis can be reassessed for the presence of any variants that may be newly linked to established genes or to newly characterized genes and/or disorders identified since the date of this report that could be associated with the patient's phenotype, based on currently available scientific information. A charge may apply for reanalysis. Please contact the laboratory for more information at the time reanalysis is requested.

TEST METHOD: Raw sequencing data was provided for the exome (on the SOLiD platform) and genome (on the CGI platform) for the proband and parental samples. Bioinformatic analyses were performed to examine potential explanations for disease in the proband**.** These analyses included variant calling, variant annotation, ancestry analysis, analysis of allele frequencies in the ancestry-matched population, analysis of gene expression, analysis of predicted microRNA targets, computational predictions of the effect of missense mutations.

LIMITATIONS: Variants have not been confirmed by an independent analysis and could represent technical artifacts. Some types of genetic abnormalities may not be detectable with the technologies performed by this exome analysis test. It is possible that the genomic region where a disease causing mutation exists in the proband was not captured using the current technologies and therefore was not detected. Additionally, it is possible that a particular genetic abnormality may not be recognized as the underlying cause of the genetic disorder due to incomplete scientific knowledge about the function of all genes in the human genome and the impact of variants in those genes. Only variants in genes associated with the medical condition, or thought to potentially be clinically relevant for the proband's medical condition, are reported here.

REFERENCES:

1. Kruse M, Schulze-Bahr E, Corfield V, Beckmann A, Stallmeyer B, Kurtbay G, Ohmert I, Schulze-Bahr E, Brink P, Pongs O. J Clin Invest. 2009 Sep;119(9):2737-44

2. Liu H, El Zein L, Kruse M, Guinamard R, Beckmann A, Bozio A, Kurtbay G, Mégarbané A, Ohmert I, Blaysat G, Villain E, Pongs O, Bouvagnet P. Circ Cardiovasc Genet. 2010 Aug;3(4):374-85.

3. Stallmeyer B, Zumhagen S, Denjoy I, Duthoit G, Hébert JL, Ferrer X, Maugenre S, Schmitz W, Kirchhefer U, Schulze-Bahr E, Guicheney P, Schulze-Bahr E. Hum Mutat. 2012 Jan;33(1):109-17.

4. Wolfsgruber W, Feil S, Brummer S, Kuppinger O, Hofmann F, Feil R. A proatherogenic role for cGMP-dependent protein kinase in vascular smooth muscle cells. Proc Natl Acad Sci U S A. 2003 Nov 11;100(23):13519-24. Epub 2003 Nov 3.

5. Fiedler B, Lohmann SM, Smolenski A, Linnemuller S, Pieske B, Schroder F, Molkentin JD, Drexler H, Wollert KC. Inhibition of calcineurin-NFAT hypertrophy signaling by cGMP-dependent protein kinase type I in cardiac myocytes. Proc Natl Acad Sci U S A. 2002 Aug 20;99(17):11363-8. Epub 2002 Aug 12.

Report draft provided by Heather McLaughlin, PhD, FACMG on Sep 27th, 2012.
Report approved by Heidi L. Rehm, PhD, FACMG on Sep 27th, 2012.

BWH Resource Center
for Clinical Genomics
41 Avenue Louis Pasteur, Suite 309
Tel: 617-264-5833 Fax: 617-264-3018

TEST PERFORMED Exome and Genome Sequencing
INDICATION FOR TEST- Right bundle branch block (RBBB) and resolved right ventricular mass in 2-1

CLINICAL GUIDANCE *

A. **Diagnostic Certainty of RBBB and Right Ventricular Mass in Affected Proband** – The RBBB diagnosis is believed to be reliable; however it has a very large list of potential genetic and non genetic etiologies. The resolved mass could also have a genetic or non-genetic basis, and could perhaps even represent misreading of the imaging test or artifact. The clinical scenario is however consistent with neonatal cardiac rhabdomyoma which is strongly associated with tuberous sclerosis. There is no structural heart disease other than post natal onset of enlargement of the aorta recognized in the 5th year of life.
CLINICAL GENOMICS ACTION TO CONSIDER: None recommended at this time.

B. **Diagnostic Certainty of a Lack of Right Ventricular Mass or other relevant findings in First Degree Relatives** – The mother (2-2) shares the RBBB, however we cannot be sure that the mother or the father do not have subclinical findings consistent with TSC.
CLINICAL GENOMICS ACTION TO CONSIDER: Formal expert clinical evaluation of mother and father for both structural heart disease and signs of Tuberous Sclerosis (if not already undertaken).

C. **Follow-up Clinical Testing Based on Genomic Findings** – The surviving unaffected first degree relatives of 2-2 and 2-4 may have sub-clinical or undiagnosed cardiac disease.
CLINICAL GENOMICS ACTION TO CONSIDER: The parents and the 40 year old unaffected brother of 2-2 and 2-6 should be clinically evaluated for sub-clinical conduction abnormalities or structural heart disease.

D. **Follow-up DNA Sequencing Based on Reported Family History** – the testing of the two trios has achieved several probable diagnostic results. The pedigree provided suggests that additional testing of first degree relatives for identified mutations will potentially supplement the analysis.
CLINICAL GENOMICS ACTION TO CONSIDER: Targeted mutation testing in a sample from the proband's deceased maternal aunt (if obtainable), as well as the unaffected maternal uncle and the maternal grandparents will assist in determining the pathogenicity of the candidate genes.

E. **Additional follow-up to Improve Confidence in the Putative Genomic Diagnosis** – this test has not established a causative relationship between the variants identified and the cardiac diseases observed. The association observed here will be strengthened by: [a] additional cases in unrelated patients, [b] in vitro studies.
CLINICAL GENOMICS BASED ACTION TO CONSIDER: In vitro assays as possible.

F. **Genomic Implications for Prognosis in Proband** – no known data

Clinical Guidance draft provided by: **Monica A. Giovanni, MS, CGC on Sep 30th, 2012.**

Clinical Guidance approved by: **Michael F. Murray, MD, FACMG on Sep 30th, 2012.**

** This guidance does not replace the clinical judgment of the patient's health care team. The guidance will be of limited value and may in fact be incorrect in cases where the data provided is incomplete or inaccurate. Periodic re-evaluation and updating of clinical guidance is recommended.*
