# Supplementary material for: An international effort towards developing standards for best practices in analysis, interpretation and reporting of clinical genome sequencing results in the CLARITY Challenge
Source: Genome Biol. 2014 Mar 25;15(3):R53. doi: 10.1186/gb-2014-15-3-r53 (PMC4073084; doi:10.1186/gb-2014-15-3-r53)
Supplement: Additional file 1 — The complete entry from the Brigham and Woman’s Team containing seven PDF files, six PNG image files, and one XLS table. [file gb-2014-15-3-r53-S1.zip › Additional_file_1/W3_final_clinical.pdf]

Laboratory for Molecular Medicine
65 Landsdowne St, Cambridge, MA 02139
Tel: 617-768-8500 Fax: 617-768-8513
pcpgm.partners.org/lmm

**Patient Name:**	W3	**Specimen type:**	DNA from peripheral blood
**DOB:**	6 yr	**Date specimen obtained:**	
**Lab Accession:**		**Date specimen received:**	5/01/12
**Pedigree #:**		**Referring physician**	
**Gender:**	Male	**Referring facility**	
**Race:**	White	**Referring facility MRN:**	

TEST PERFORMED – Exome and Genome Sequencing

INDICATION FOR TEST – Nemaline myopathy

RESULT: Variants were identified in a gene with a plausible role in the reported phenotype

DNA VARIANTS:

**Gene**	**Variant**	**Classification**	**Parental Inheritance**
OBSCN	Het c.2245G>T (p.Gly749Cys)	Uncertain Significance	Paternal
OBSCN	Het c.3322T>A (p.Tyr1108Asn)	Uncertain Significance	Maternal

INTERPRETATION SUMMARY: This individual is reported to have nemaline myopathy in the absence of a family history. Candidate variants from genome and exome sequencing were therefore analyzed with consideration for possible *de novo* or recessive causes of disease. No rare variants were identified in genes known to be associated with nemaline myopathy (*ACTA1, CFL2, KBTBD13, NEB, TNNT1,TPM2,TPM3* Insufficient sequencing data was obtained for 7% of the coding regions for these genes, and therefore the presence of a rare or pathogenic variant within them cannot be fully excluded.

Analysis of additional candidate variants revealed one gene, *OBSCN*, with the highest plausible role in this individual's disease. Two missense variants of unknown significance, one inherited from each parent, were identified. *OBSCN* encodes the obscurin protein, which is involved in sarcomere assembly through interactions with other muscle proteins (Ackermann 2009, Bang 2001, Borisov 2008, Ford-Speelman 2009, Kontrogianni-Konstantopoulos 2004, Young 2001). This gene is also highly expressed in striated muscle (Russell 2002, Carlsson 2008). Both variants are located in a domain of the OBSCN protein that interacts with other proteins with an established role in myopathy (titin and myomesin; Fukuzawa 2008, Lange 2009, Young 2001). This supports that the combination of these 2 variants may be responsible for this individual's phenotype. Computational and population analysis as noted in the variant descriptions below are consistent with, though not conclusive for, a deleterious impact to the protein.

Note, *OBSCN* is a long gene that encodes a large protein. The chance of observing a compound heterozygote for rare missense variants in the general population is estimated to be 0.009. In summary, the available evidence supports that the combination of the two OBSCN variants is responsible for this individual's reported clinical diagnosis. However, additional information is needed to establish the clinical significance of these variants and their role in disease.

Several additional variants were considered but were felt to be unlikely causes of the phenotype. Two variants, a synonymous variant in *DCAF17* and a missense variant in *ARHGDIG*, were observed as *de novo* variants but were considered unlikely causes based upon absence of expression in muscle and absence any other plausible disease or functional data that that could implicate these genes in the disease. Rare compound heterozygous variants in the *USP6* gene were also considered but were excluded based upon computation analyses, limited technical confidence in the variant calls (allelic imbalance, highly repetitive region) and lack of a plausible biological or functional role in disease.

INDIVIDUAL VARIANT INTERPRETATIONS:

Gly749Cys in *OBSCN*- Uncertain Significance

This variant as not been reported nor previously identified by our laboratory. It has not been identified in large and broad European American and African American populations by the NHLBI Exome Sequencing Project (http://evs.gs.washington.edu/EVS). Computational analyses suggest that this variant may impact the protein, though this information is not predictive enough to determine pathogenicity. Additional information is needed to further classify the clinical significance of the Gly749Cys variant.

Tyr1108Asn in *OBSCN*- Uncertain Significance

This variant as not been reported nor previously identified by our laboratory. It has not been identified in large and broad European American and African American populations by the NHLBI Exome Sequencing Project (http://evs.gs.washington.edu/EVS). Computational analyses suggest that this variant may impact the protein, though this information is not predictive enough to determine pathogenicity. Additional information is needed to further classify the clinical significance of the Tyr1108Asn variant.

RECOMMENDATIONS:

Please note, a DNA sample was unavailable to confirm the technical results of this test. Therefore, we recommend an independent confirmation of all clinically relevant findings before medical action is considered.

Genetic counseling is recommended for this individual and their family. For assistance in locating nearby genetic counseling services please contact the laboratory at 123-456-7890.

A medical provider can request reanalysis of the exome data,and this is recommended on an annual basis. Data from this exome sequencing analysis can be reassessed for the presence of any variants that may be newly linked to established genes or to newly characterized genes and/or disorders identified since the date of this report that could be associated with the patient’s phenotype, based on currently available scientific information. A charge may apply for reanalysis. Please contact the laboratory for more information at the time reanalysis is requested.

TEST METHOD: Raw sequencing data was provided for the exome (on the SOLiD platform) and genome (on the CGI platform) for the proband and parental samples. Bioinformatic analyses were performed to examine potential explanations for disease in the proband**.** These analyses included variant calling, variant annotation, ancestry analysis, analysis of allele frequencies in the ancestry-matched population, analysis of gene expression, analysis or protein-protein interactions data, computational predictions of the effect of missense mutations, computational predictions of the effect on splicing and estimation of gene-specific background probabilities of de novo mutations, rare homozygote and compound heterozygote variants.

LIMITATIONS: Variants have not been confirmed by an independent analysis and could represent technical artifacts. Some types of genetic abnormalities may not be detectable with the technologies performed by this exome analysis test. It is possible that the genomic region where a disease causing mutation exists in the proband was not captured using the current technologies and therefore was not detected. Additionally, it is possible that a particular genetic abnormality may not be recognized as the underlying cause of the genetic disorder due to incomplete scientific knowledge about the function of all genes in the human genome and the impact of variants in those genes. Only variants in genes associated with the medical condition, or thought to potentially be clinically relevant for the proband’s medical condition, are reported here.

REFERENCES:

Ackermann MA, Hu LY, Bowman AL, Bloch RJ, Kontrogianni-Konstantopoulos A. Obscurin interacts with a novel isoform of MyBP-C slow at the periphery of the sarcomeric M-band and regulates thick filament assembly. Mol Biol Cell. 2009 Jun;20(12):2963-78. Epub 2009 Apr 29.

Bang ML, Centner T, Fornoff F, Geach AJ, Gotthardt M, McNabb M, Witt CC, Labeit D, Gregorio CC, Granzier H, Labeit S. The complete gene sequence of titin, expression of an unusual approximately 700-kDa titin isoform, and its interaction with obscurin identify a novel Z-line to I-band linking system. Circ Res. 2001 Nov 23;89(11):1065-72.

Borisov AB, Martynova MG, Russell MW. Early incorporation of obscurin into nascent sarcomeres: implication for myofibril assembly during cardiac myogenesis. Histochem Cell Biol. 2008 Apr;129(4):463-78. Epub 2008 Jan 25.

Ford-Speelman DL, Roche JA, Bowman AL, Bloch RJ. The rho-guanine nucleotide exchange factor domain of obscurin activates rhoA signaling in skeletal muscle. Mol Biol Cell. 2009 Sep;20(17):3905-17. Epub 2009 Jul 15.

Kontrogianni-Konstantopoulos A, Catino DH, Strong JC, Randall WR, Bloch RJ. Obscurin regulates the organization of myosin into A bands. Am J Physiol Cell Physiol. 2004 Jul;287(1):C209-17. Epub 2004 Mar 10.

Young P, Ehler E, Gautel M. Obscurin, a giant sarcomeric Rho guanine nucleotide exchange factor protein involved in sarcomere assembly. J Cell Biol. 2001 Jul 9;154(1):123-36.

Russell MW, Raeker MO, Korytkowski KA, Sonneman KJ. Identification, tissue expression and chromosomal localization of human Obscurin-MLCK, a member of the titin and Dbl families of myosin light chain kinases. Gene. 2002 Jan 9;282(1-2):237-46.

Carlsson L, Yu JG, Thornell LE. New aspects of obscurin in human striated muscles. Histochem Cell Biol. 2008 Jul;130(1):91-103. Epub 2008 Mar 19.

Fukuzawa A, Lange S, Holt M, Vihola A, Carmignac V, Ferreiro A, Udd B, Gautel M. Interactions with titin and myomesin target obscurin and obscurin-like 1 to the M-band: implications for hereditary myopathies. J Cell Sci. 2008 Jun 1;121(Pt 11):1841-51. Epub 2008 May 13.

Lange S, Ouyang K, Meyer G, Cui L, Cheng H, Lieber RL, Chen J. Obscurin determines the architecture of the longitudinal sarcoplasmic reticulum. J Cell Sci. 2009 Aug 1;122(Pt 15):2640-50. Epub 2009 Jul 7.

Young P, Ehler E, Gautel M. Obscurin, a giant sarcomeric Rho guanine nucleotide exchange factor protein involved in sarcomere assembly. J Cell Biol. 2001 Jul 9;154(1):123-36.

Report approved by Heidi L. Rehm, PhD, FACMG on Sep 27th, 2012.

BWH Resource Center
for Clinical Genomics
41 Avenue Louis Pasteur, Suite 309
Tel: 617-264-5833 Fax: 617-264-3018

**TEST PERFORMED**	Exome and Genome Sequencing
**INDICATION FOR TEST**	Nemaline Myopathy (NEM) in 3-1

CLINICAL GUIDANCE *

A. **Diagnostic Certainty of NEM in Affected Proband** – This diagnosis was reported to have been made in this case on the basis of the defining histo-pathological finding performed at 7 months of age; there is no reason identified that raises doubt about the diagnosis. It is noted that this histo-pathological diagnosis has a genetically heterogeneous etiology, and has a broad clinical spectrum.
CLINICAL GENOMICS ACTION TO CONSIDER: None

B. **Diagnostic Certainty of a Lack of NEM in First Degree Relatives** – Individuals with NEM typically have a variable onset of muscle weakness. If not already done formal clinical evaluation of first degree family members should be carried out to rule out unrecognized or subclinical disease.
CLINICAL GENOMICS ACTION TO CONSIDER: Formal expert clinical evaluation of mother, father, and 16 month old sister (if not already undertaken).

C. **Follow-up Clinical Testing Based on Genomic Findings** – Since NEM has both dominant and recessive forms, it is assumed that our candidate OBSCN is associated with recessive inheritance, however should either parent of 3-1 show sub-clinical or unrecognized "adult onset" disease, then further testing of both maternal and paternal relatives would be indicated.
CLINICAL GENOMICS ACTION TO CONSIDER: Further testing of relatives if mother or father is diagnosed with myopathy.

D. **Follow-up DNA Sequencing Based on Reported Family History** – the testing of the trio has achieved a probable diagnostic result. The pedigree provided suggests that the sister of the proband should undergo testing for OBSCN mutations.
CLINICAL GENOMICS ACTION TO CONSIDER: None for parents. Targeted testing of the proband's sister is indicated.

E. **Additional follow-up to Improve Confidence in the Putative Genomic Diagnosis** – this test has not established a causative relationship between OBSCN and Nemaline Myopathy. The association observed here will be strengthened by: [a] additional cases in unrelated patients, [b] in vitro studies.
CLINICAL GENOMICS BASED ACTION TO CONSIDER: In vitro assays as possible.

F. **Genomic Implications for Prognosis in Proband** – no known data

Clinical Guidance draft provided by: **Monica A. Giovanni, MS, CGC on Sep 30th, 2012.**

Clinical Guidance approved by: **Michael F. Murray, MD, FACMG on Sep 30th, 2012.**

** This guidance does not replace the clinical judgment of the patient's health care team. The guidance will be of limited value and may in fact be incorrect in cases where the data provided is incomplete or inaccurate. Periodic re-evaluation and updating of clinical guidance is recommended.*
