# Supplementary figures and images for: An international effort towards developing standards for best practices in analysis, interpretation and reporting of clinical genome sequencing results in the CLARITY Challenge
Source: Genome Biol. 2014 Mar 25;15(3):R53. doi: 10.1186/gb-2014-15-3-r53 (PMC4073084; doi:10.1186/gb-2014-15-3-r53)

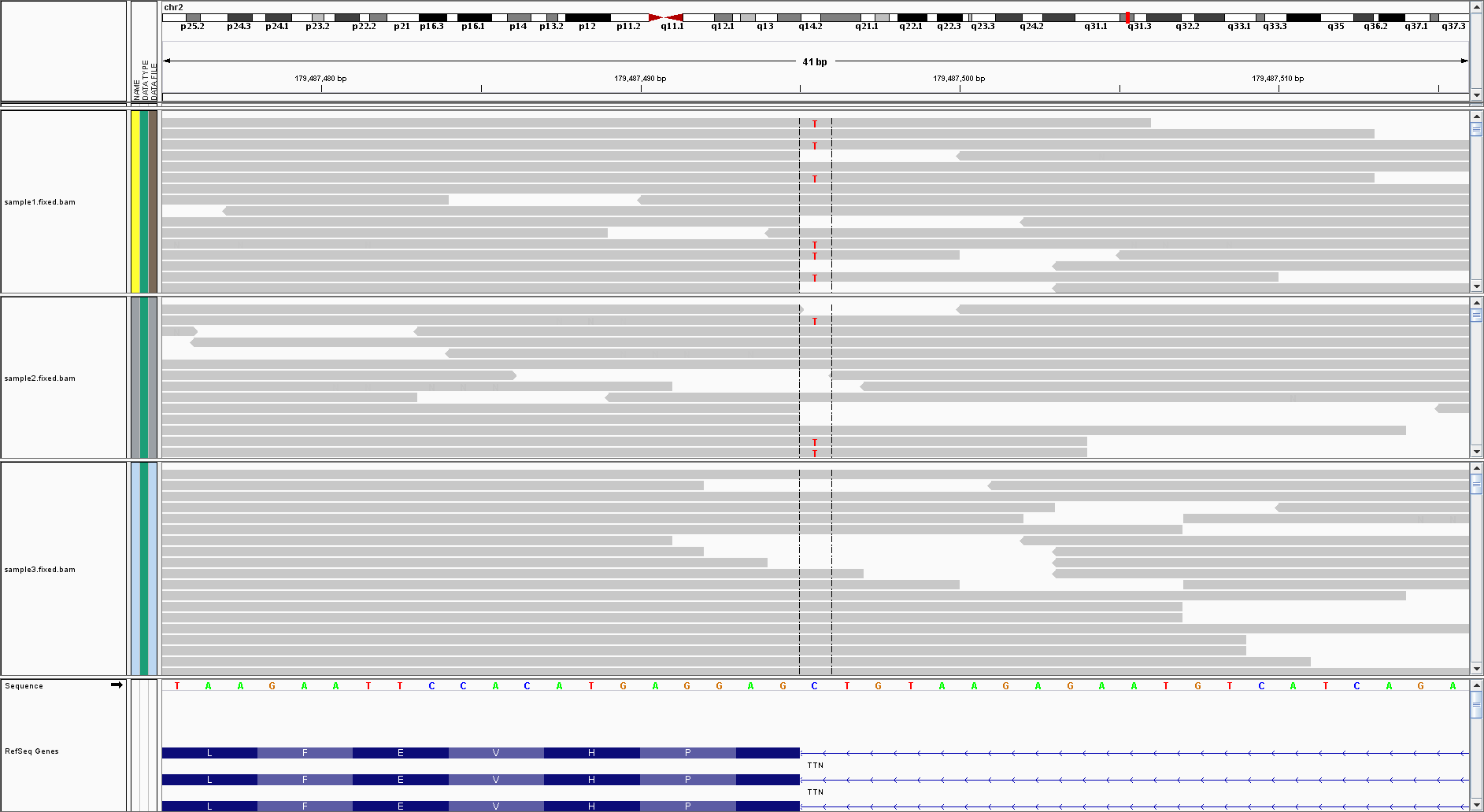

Supplement: Additional file 1 — The complete entry from the Brigham and Woman’s Team containing seven PDF files, six PNG image files, and one XLS table. [file gb-2014-15-3-r53-S1.zip › Additional_file_1/igv_TTN1.png]

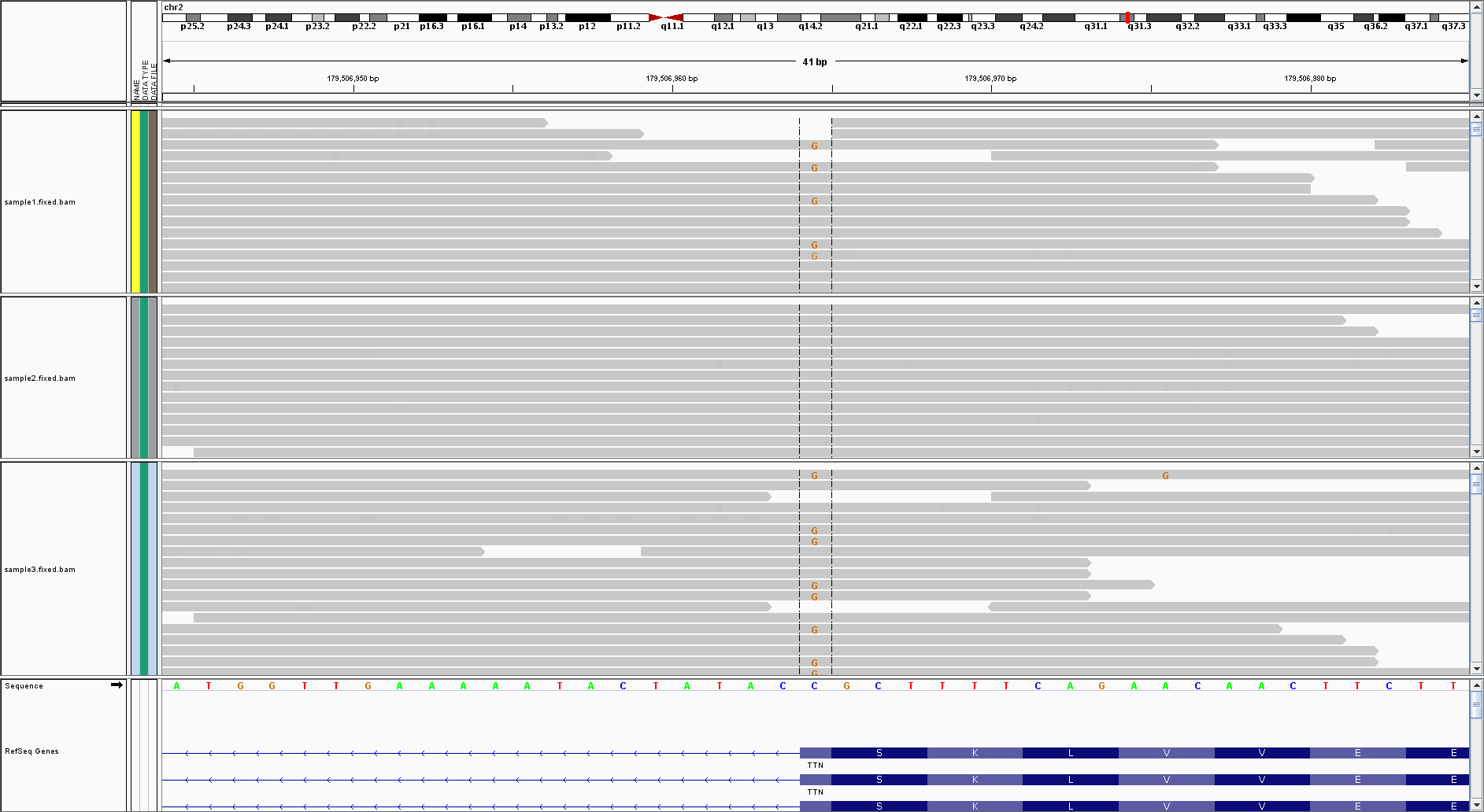

Supplement: Additional file 1 — The complete entry from the Brigham and Woman’s Team containing seven PDF files, six PNG image files, and one XLS table. [file gb-2014-15-3-r53-S1.zip › Additional_file_1/igv_TTN2.png]

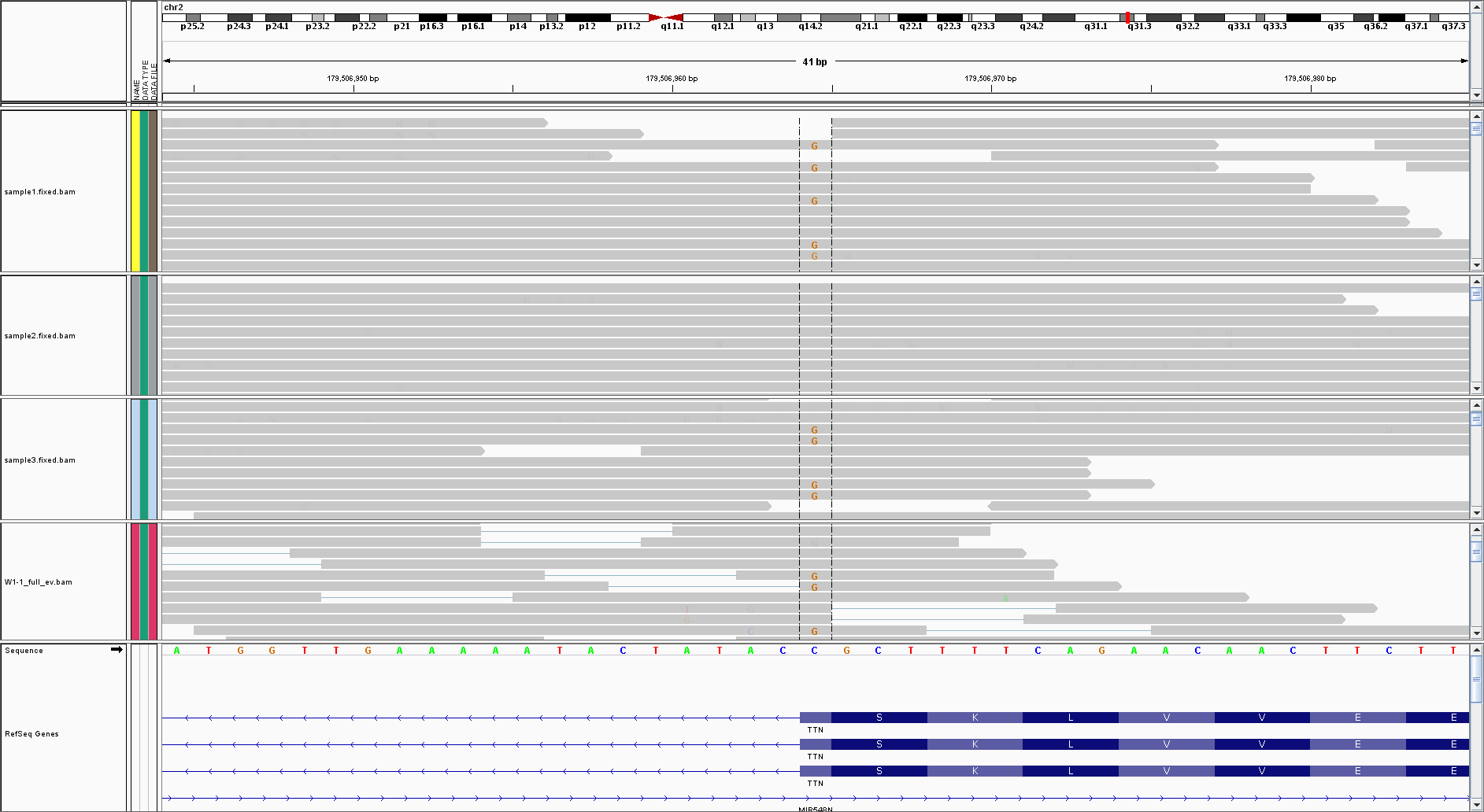

Supplement: Additional file 1 — The complete entry from the Brigham and Woman’s Team containing seven PDF files, six PNG image files, and one XLS table. [file gb-2014-15-3-r53-S1.zip › Additional_file_1/igv_TTN2_with_WGS.png]

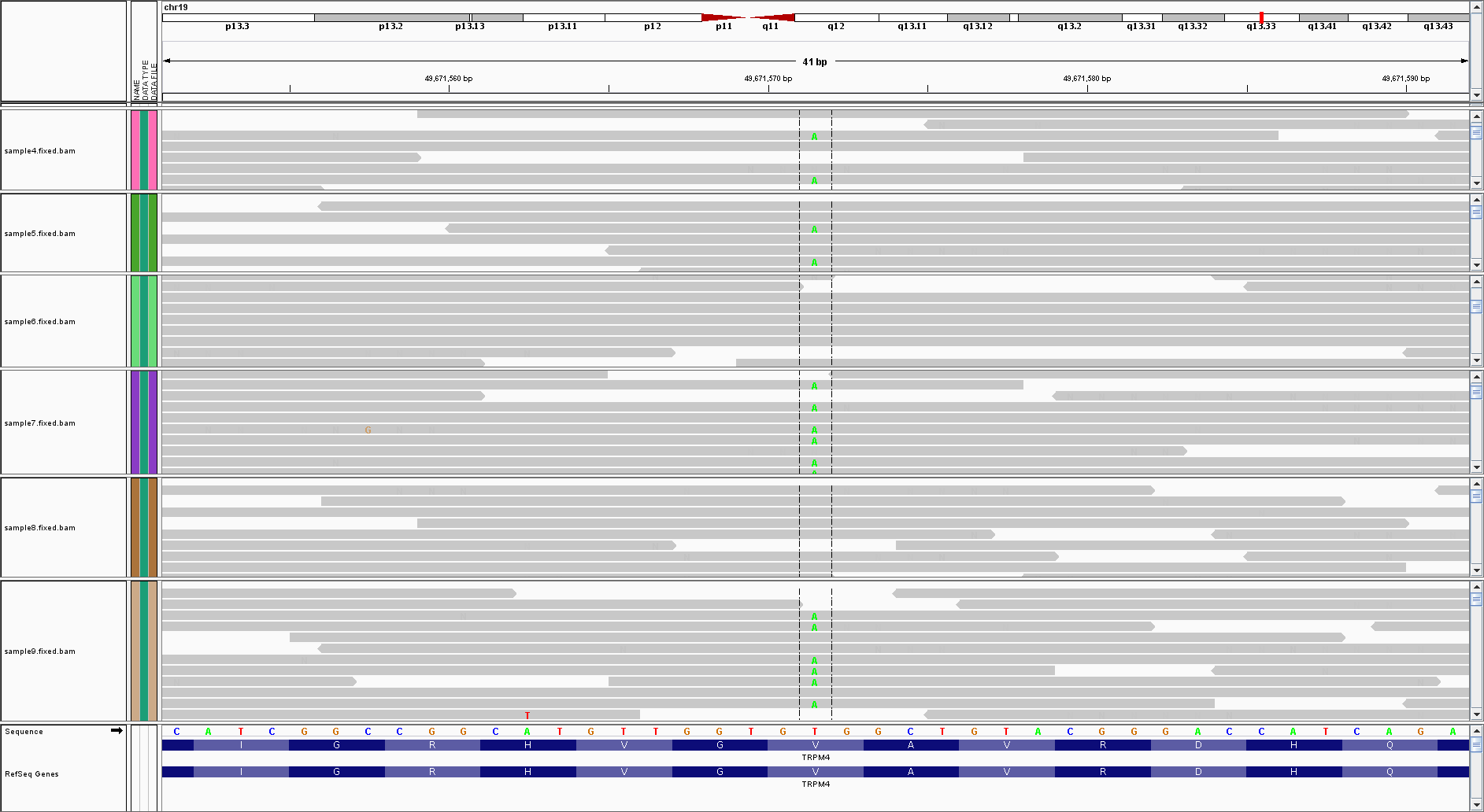

Supplement: Additional file 1 — The complete entry from the Brigham and Woman’s Team containing seven PDF files, six PNG image files, and one XLS table. [file gb-2014-15-3-r53-S1.zip › Additional_file_1/igv_W2_TRPM4.png]

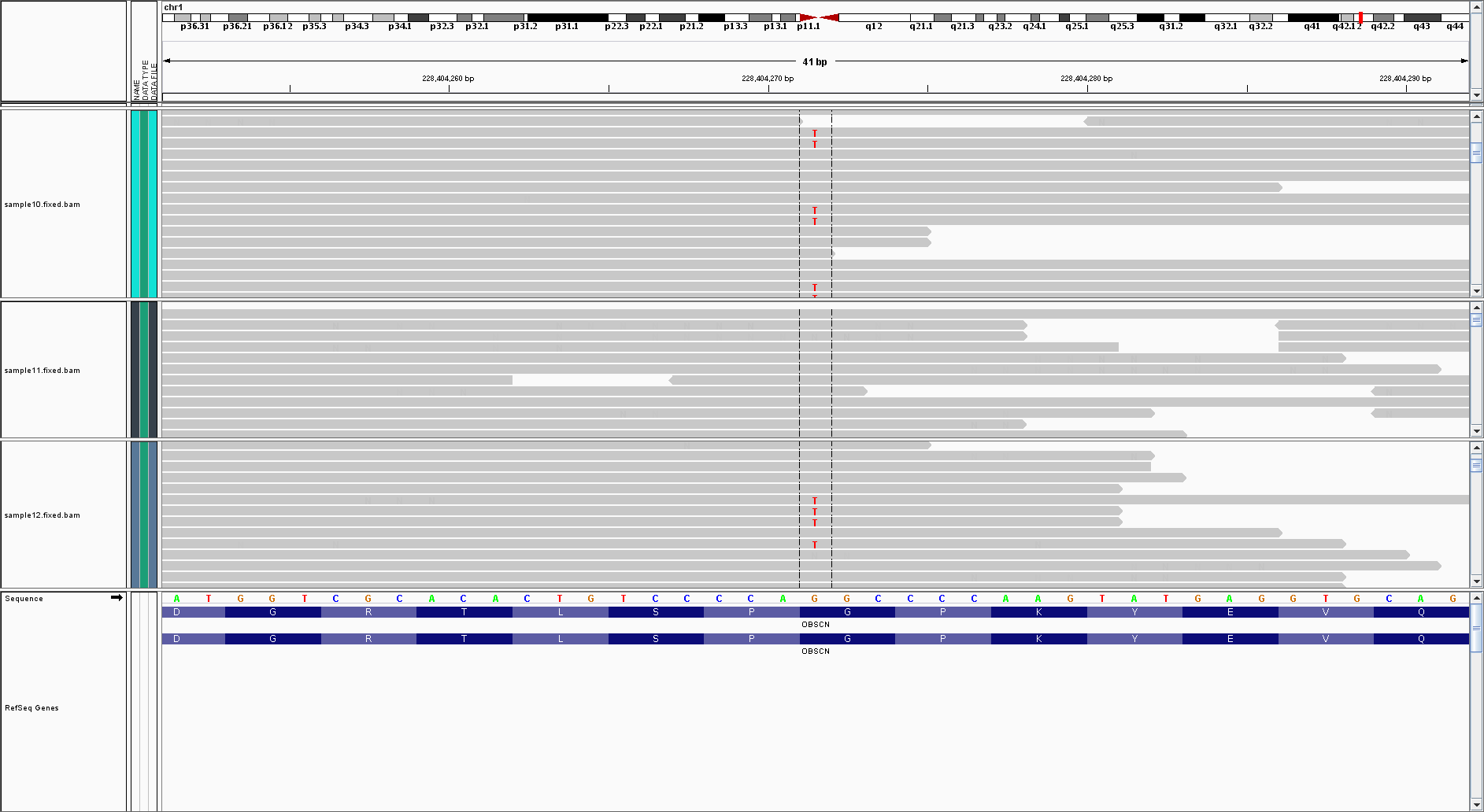

Supplement: Additional file 1 — The complete entry from the Brigham and Woman’s Team containing seven PDF files, six PNG image files, and one XLS table. [file gb-2014-15-3-r53-S1.zip › Additional_file_1/igv_W3_OBSCN1.png]

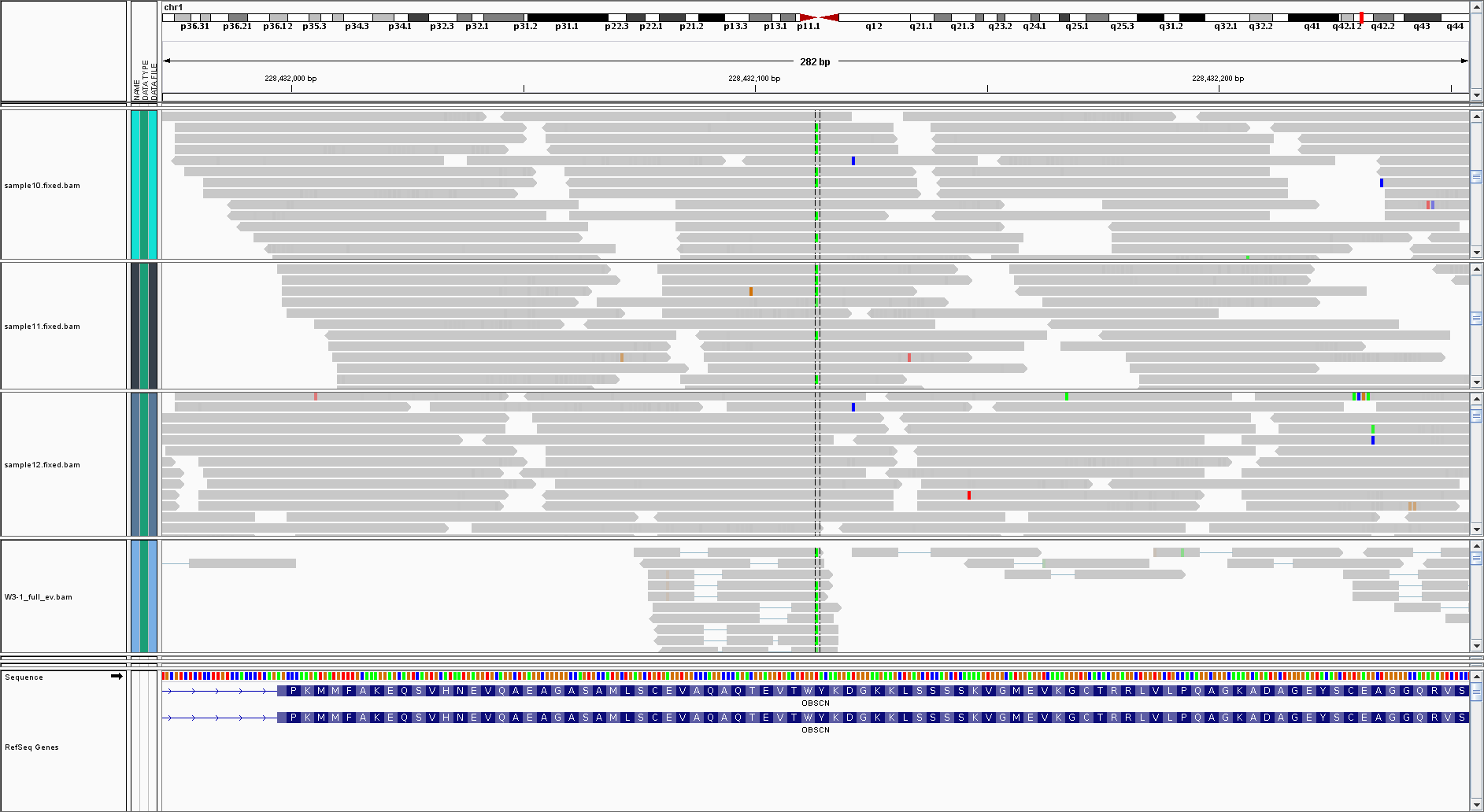

Supplement: Additional file 1 — The complete entry from the Brigham and Woman’s Team containing seven PDF files, six PNG image files, and one XLS table. [file gb-2014-15-3-r53-S1.zip › Additional_file_1/igv_W3_OBSCN2_with_WGS.png]
