# Supplementary material for: An international effort towards developing standards for best practices in analysis, interpretation and reporting of clinical genome sequencing results in the CLARITY Challenge
Source: Genome Biol. 2014 Mar 25;15(3):R53. doi: 10.1186/gb-2014-15-3-r53 (PMC4073084; doi:10.1186/gb-2014-15-3-r53)
Supplement: Additional file 2 — The entry from the Genomatix/CeGaT/University Hospital of Bonn team containing five PDF files and six XLS tables. [file gb-2014-15-3-r53-S2.zip › Additional_file_2/CLARITY_challenge.pdf]

CLARITY Challenge 2012

Children's Leadership Award for the Reliable Interpretation and appropriate Transmission of Your genomic information

Methods for analysis and interpretation

September 25, 2012

*Correspondence: Jochen Supper (supper@genomatix.de, Genomatix) &
Saskia Biskup (saskia.biskup@cegat.de, CeGaT)*

Summary

Goals and scope

The goal of this report is to provide an understanding of all analysis steps taken to analyze and interpret the CLARITY challenge data. For most analysis steps the quality of software and annotations has been widely discussed (Nielson *et al.* 2011). In this work, instead of reiterating such comparisons, we focus on the three cases presented here and discuss which approaches and annotations allowed us to find the presumed causative variants (single nucleotide variants (SNVs) and small insertions or deletions (indels)).

Analysis and interpretation

Data processing: mapping, variant calling & annotation

For the whole exome sequencing (WES) data we applied several mapping, variant calling and annotation workflows. Most of the presumed causative variants were detected in all workflows, however, in some cases variants were overlooked (see Section *Comparing different variant approaches*). Finally, the LifeScope and CompleteGenomics pipelines were both able to detect all causative variants.

Filtering and annotating variants

Our filtering strategy consists of two stages, first a variant based and second a gene annotation based filtering. The first stage includes a genotype and a population frequency filter. The genotype filter (filtering on inheritance patterns) removes all variant combinations that are not plausible according to the clinical report (e.g. autosomal dominant). The population frequency filter removes all frequent variants based on the Global Allele Frequency (GAF) score (more than 5%) from the 1000 Genomes Project and an internal allele frequency database by CeGaT. In the second stage all remaining variants are linked to expert-curated (e.g., OMIM and CeGaT panels) and literature data mining based disease associations (compiled by Genomatix LitInspector). With this strategy and MESH disease ontologies we were able to link all causative variants to the diagnoses. This setup provides a powerful search strategy and facilitates the generation of comprehensive and comprehensible reports.

Web-interface, infrastructure and integration

GeneGrid: interface & data hosting

To make all data available to medical staff, we installed a server and web-client based infrastructure, termed „geneGrid". All variants and annotations are stored in a database on which users can view and filter data. (see supplemented geneGrid webcast).

Annotation integration and Preliminary report

The final medical reports supplied with this submission have been written by medical experts. A preliminary report, however, can be automatically generated from the variants that pass the filters and appear to be causative. In addition, the infrastructure of geneGrid allows users to integrate custom annotations and in-house background populations.

Conclusions and recommendations

Pedigree analysis in combination with medical history, background populations and disease annotations provide a powerful information basis to find the needle in the haystack. This is owed to several favorable properties of our setup. First, pedigree analyses allow to rule out all variant combinations that do not align with the expected inheritance pattern. Second, when looking for rare diseases, background populations are a simple but powerful filter to remove polymorphisms. In addition, in-house population databases that have been generated with one platform allow the removal of false positive (FP) variants that occur due to technical and nucleotide biases or errors in the reference genome. Finally, we can filter variants based on disease annotations. In this work we show that with a combination of expert-curated and LitInspector based gene-disease annotations we were able to uncover presumably causative variants for the primary diagnosis in all families and even the secondary diagnosis in families 1 and 2 (sensorineural hearing loss and possibly pulmonary stenosis).

As final result we provide straightforward and accessible variant calls and filtering steps. Each variant has an inheritance pattern, a GAF frequency and an associated disease that is linked to publications or OMIM identifiers. This allows for a direct translation of the filtering steps into a comprehensible report.

CeGaT Center for Genomics and Transcriptomics

department of prostate cancer research

genomatix

Team

For the CLARITY challenge we have combined the expertise of two clinical groups and two biotech companies. CeGaT provided knowledge on the sequencing and diagnostic panels, which is complemented by Saskia Biskup's (CEO) background as human geneticist. The Perner lab has successfully applied NGS in a clinical setting for biomarker identification and diagnostic translation. Genomatix contributed knowledge on annotation databases and a web-based framework for delivering software solutions to researchers and clinicians. Pediatrician Peter Freisinger, from the Children's Hospital Reutlingen, provided expert knowledge.

Pipeline overview

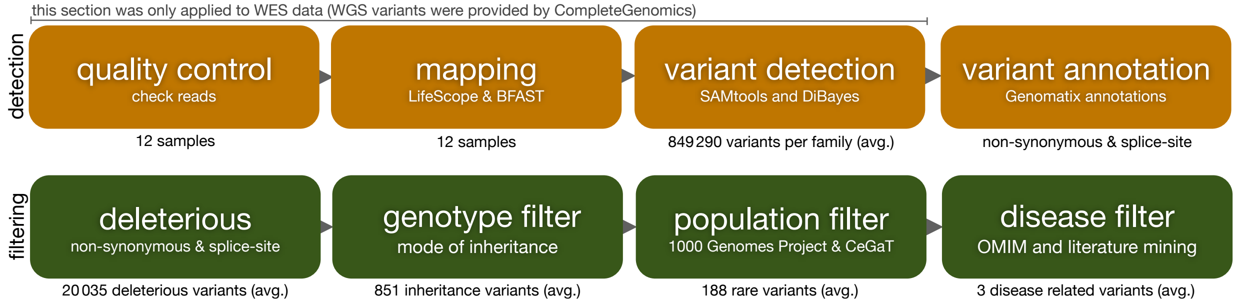

Schematic overview of the analysis pipeline

Table of Contents

Data, analysis and interpretation 1

Subjects and methods 1

Whole genome (WGS) and whole exome sequencing (WES) 1

NGS data processing 2

Alignment 2

Genotype calling 2

Variant annotation and analysis 3

Scoring variants 3

Screening for frequency of variants (1000 Genomes Project) 3

Screening for frequency of variants (in-house background) 3

Variant prioritization scores 4

Annotations 5

Gene-disease databases 5

Custom gene panels 5

Automated literature mining 6

Disease ontologies and search strategy 6

Trio analysis and filter settings 6

Results for families 1- 3 8

Variant statistics 8

Applying different disease filter settings 10
Family W1 (Centronuclear myopathy and hearing loss) 10
Family W2 (RBBB and pulmonary stenosis) 12
Family W3 (Nemaline myopathy) 15
Details on potential disease causing variants 16
Comparing different variant approaches 16
geneGrid: a novel tool for comparative variant analysis 19
Conclusions & recommendations 21
Supplements 22
References 23

Data, analysis and interpretation

Subjects and methods

The CLARITY challenge involves three families that show clinical manifestations and a pedigree that suggests a genetic basis for their disorders. For detailed information about the family selection process, sample preparation and medical record collection we refer to the Participation Agreement (Exhibit A). As starting point for the challenge we were provided with WGS and WES data from three families, along with medical records and pedigree history.

Whole genome (WGS) and whole exome sequencing (WES)

The genomes of all three families were sequenced (WGS and WES) and provide the basis for the molecular analysis of the Challenge (see Table 1).

sample id	WES	WGS	family	individual	phenotype
W1-01	sample1	W1-01	1	son	affected
W1-02	sample2	W1-02	1	mother	unaffected
W1-03	sample3	W1-03	1	father	unaffected
W2-01	sample4	W2-01	2	daughter	affected
W2-02	sample5	W2-02	2	mother	affected
W2-03	sample6	W2-03	2	father	unaffected
W2-04	sample7		2	cousin (male)	affected
W2-05	sample8		2	maternal aunt	unaffected
W2-06	sample9	W2-06	2	maternal uncle	affected
W3-01	sample10	W3-01	3	son	affected
W3-02	sample11	W3-02	3	mother	unaffected
W3-03	sample12	W3-03	3	father	unaffected

Table 1: Overview of the families and the WES and WGS sequencing.

Whole exome sequencing (WES)

12 DNA samples were received by the LIFE lab in LIFE/Carlsbad for initial processing.They were sheared and made into fragment libraries as per standard protocol for the LIFE Library Builder, then sequenced with the ECC chemistry on SOLiD 5500xl (further details were provided by Children's). From this dataset, Children's provided all raw data in XSQ-format and the mapping files (BAM-format) which were generated with LifeScope.

Whole genome sequencing (WGS)

Whole genome sequencing was performed by CompleteGenomics for 10 out of the 12 family members (see Table 1). Children's provided all sequencing, mapping and variant calling data generated by CompleteGenomics.

NGS data processing

Alignment

The SOLiD reads were aligned to the genomic reference (hg19). Two aligners were applied, LifeScope aligner (version 2.5 - using SAET as read correction method) and BFAST (Homer *et al.* 2009) .

The WGS read alignments to the genomic reference (NCBI build 37.2) were provided directly by CompleteGenomics (software version 2.0.2.22).

Quality control

The groups in this team (Genomatix, CeGaT and Uni Bonn) all have quality control (QC) measures in place to analyze their mapping and raw data. Generally it is highly recommended to do QC reports on raw and mapping data, to be sure the next steps are unbiased because of low quality. In the context of this challenge we chose not to provide a detailed discussion on QC of raw reads and mapping results. Such analyses depends on the sequencing platform, the specific setup and many useful tools are already available for this task (e.g., FastQC).

Genotype calling

For genotype calling DiBayes (Tang *et al.* 2008), GATK (DePristo *et al.* 2011) and SAMtools were applied (Li *et al.* 2009). These genotype calling tools are used on a regular basis by our team members, and have shown to provide good results. In case of the WGS data, we

were provided with variant calls by CompleteGenomics (software version 2.0.2.22), which we used directly for our pipeline.

Variant annotation and analysis

To annotate the variants we again employed multiple approaches. One was NGS-SNP (Grant *et al.* 2011) (based on Ensembl 64), the other was the Genomatix large scale SNP analysis (version 1.2). These SNP annotations are based on the ElDorado transcript database (version 08-2011), which integrates transcripts from RefSeq (release 46), Ensembl (release 61) and GenBank (download July 2011).

The WGS data was annotated by the CompleteGenomics software (version 2.0.2.22) using the dbSNP build 132 (Sherry *et al.* 2000) and the gene annotations from the NCBI build 37.2.

In addition, we scanned for transcription factor binding site (TFBS) changes that could potentially affect gene regulation. This analysis was performed using MatInspector (Quandt *et al.* 1996, Cartharius *et al.* 2005). TFBS predictions are based on Genomatix‘ TF database, MatBase (version 8.4).

Scoring variants

Screening for frequency of variants (1000 Genomes Project)

When searching for rare diseases, it is very helpful to compare the variants found in affected individuals against background populations. Today, several such populations exist that allow one to derive population frequency measures. These are, for instance, 1000 Genomes Project (Durbin *et al.* 2010), ESP (http://evs.gs.washington.edu/EVS/ - Exome Sequencing Project), HapMap (Frazer *et al.* 2007) and 69 genomes from CompleteGenomics (Drmanac *et al.* 2010). For each variant called within these populations, the Global Allele Frequencies (GAFs) are calculated. The population used in this work is from the 1000 Genomes Project with a filter setting of 0.05. Hence, the variants are observed in under 5% of the background population.

Screening for frequency of variants (in-house background)

It is generally known that specific errors can occur on the sequencing platforms and because of mapping and reference genome errors. To neutralize such errors we computed an allele frequency for every called position based on an internal database provided by

CeGaT. This database includes mapping information of 132 Exomes. All of them were generated with the SOLiD 5500xl instrument and reads were mapped with LifeScope. All detected variants that occur in 5% or more of the Exomes in the CeGaT database were removed.

Variant prioritization scores

Often, sequencing studies lead to a large number of potential variants that must be investigated manually. In such cases prioritization scores are helpful to first investigate variants that are more likely to cause a severe change in the protein structure or are located in strongly conserved DNA stretches.

In the setup of this challenge, family and detailed medical histories were provided. This allowed us to impose very powerful filtering techniques such as a genotype filter and a disease annotation filter. As we will show in the results section, the number of potential variants passing all filters is small and eliminates the need for prioritization scores.

The information used by prioritization filters, however, is still useful when collecting information about the genes and variants. Thus, we provide information about amino acid substitution scores and genomic conservation. For a different study design, for example, with populations, such scores would be used for prioritization. Furthermore, prioritization is needed in the investigation of TFBS changes due to their high false positive (FP) rate.

Predicting the effect of small variants on the protein

To determine how deleterious an amino acid substitution is, different scores have recently been published. The prediction of SIFT (Sorting Intolerant From Tolerant; Kumar *et al.* 2009) is based on the conservation of amino acids in sequence alignments derived from closely related proteins. PolyPhen2 (Adzhubei *et al.* 2010) predicts the impact of a amino acid change in a protein using physical and comparative considerations. MutationTaster evaluates disease-causing potential of sequence alterations, by employing a Bayes classifier to predict the disease potential of an alteration (Schwarz *et al.* 2010). NetGene2 uses a neural network to make predictions of splice sites in human (Hebsgaard *et al.* 1996). In addition to these scoring methods, we also provide the BLOSUM62 substitution score (Henikoff *et al.* 1992) for each exchanged amino acid pair.

Conservation based scores

Disease-causing variants can induce splice-site changes or changes in transcription factor (TF) binding. Such variants cannot be accessed through amino acid substitution scores. In these cases genomic conservation can be helpful to determine which variants are more likely to have a deleterious effect. Here, we used the conservations scores PhyloP (Cooper *et al.* 2005) and the functional regions reported in the 29 Mammals Project (Lindblad-Toh *et al.* 2011) as well as the NGS-SNP conservation filter (Grant *et al.* 2011).

Annotations

Gene-disease databases

Numerous databases exist that collect information about genetic phenotypes. The most widely used resources in this context are dbSNP (Single Nucleotide Polymorphism database), OMIM (Online Mendelian Inheritance in Man) (Hamosh *et al.* 2005) and HGMD (The Human Gene Mutation Database; Stenson *et al.* 2009). The database Issue of the NAR (Nucleic Acids Research) 2012 contains reports on many other online databases containing different types of genotype and phenotype resources (Galperin *et al*. 2012)

Our approach to integrate gene-disease relationships is based on three pillars: an *in-house* expert curated database, a public expert curated database and a data mining approach to extract gene-disease relations from literature (LitInspector; Frisch *et al.* 2009). The *in-house* based annotations allow the integration of knowledge on genes and diseases that have been collected internally (CeGaT panels). The public expert-curated database OMIM is used because it contains a large body of curated information that can be used to reference findings in a medical report. LitInspector is used to allow an exploratory mode, in which findings can be found that have been published but not yet included in the expert-curated databases.

Custom gene panels

CeGaT provides sequencing on a set of genes (panel) linked to a specific group of genetic disorders. These gene sets are continuously updated by experts of the disease. The gene list of a panel is divided into sub-panels that are more specific to a phenotype to offer a hierarchical search order. Filtering with genes on the expert generated panels covers all mutations in all genes associated with a genetic disorder.

Automated literature mining

LitInspector is a data mining literature search tool extracting genes, diseases, signal transduction pathways and their relations based on NCBI's PubMed database. A major challenge in gene and disease recognition is the resolution of homonyms and rejection of identical abbreviations used in a 'non-gene/disease' context. LitInspector uses automatically generated and manually refined filtering lists for this purpose. To provide a name-entity recognition for disease terms these are derived from MESH (Medical Subject Headings) (Rogers *et al.* 1963) and the NCI thesaurus NCIt. For relations between genes and diseases *p*-values are calculated. This allows searching for significantly enriched gene-disease relations or for all gene-disease relations that have been published. The default mode used here is a *p*-value of less than 0.05. A restricted online variant of LitInspector is available online: http://www.litinspector.org/

Disease ontologies and search strategy

When extracting a diagnosis from medical reports, one can attempt to directly match the diagnosis terms to gene-disease databases. In many cases this relationship between the medical term and the gene is known, but not directly identifiable through the specific term provided in the medical report. In the simplest case this might be due to synonyms used in the reports or databases. Another reason might be the level of detail. A gene might be linked to a more general diagnosis, where we are searching for a very specific one.

To give an example, patient W2-2 has the primary diagnosis *Right Bundle Branch Block (RBBB)*. In MESH this term is contained in the entity *Bundle-Branch Block*, which contains a total of 34 related terms (including *Bundle Branch Block, Right*). Both of these terms, however, are not linked to any gene in patient W2-2 with a found variant. When we look at the MESH hierarchy, we see that *Bundle-Branch Block* is contained in the more general group *heart block (*parent of Bundle-Branch Block*)*. The term *heart block* is linked to *TRPM4* through OMIM and is the gene we thus identified as putative causal variant for RBBB.

In this work we use the MESH ontology as basis for our search strategy when the primary diagnosis does not provide a direct match.

Trio analysis and filter settings

In families W1 and W3 both parents are unaffected but both children are affected. In this case our genotype search strategy includes all filters for a recessive disease, these are: compound heterozygous, x-linked, *de novo*, loss of heterozygosity and autosomal

recessive. For family W2 we assumed a dominant variation, thus we searched for a heterozygous variant for all affected family members and a reference call for all unaffected family members.

In addition to the genotype filter we removed all synonymous variants, thus only considering variants that altered the protein sequence or hit a canonical splice-site. Then we applied the background population filters. These filters removed all variants that occurred in at least than 5% of the 1000 Genomes Project or CeGaT background population DB (GAF score > 0.05).

Finally, we used gene-disease associations to filter the variant list and link the remaining variants to the diagnosed disease. To apply this filter, several term resolution aspects must be considered. First, the diagnosis itself can potentially be described by different synonyms. Thus, a primary diagnosis term from a patient record might not have a direct entry in a gene-disease database, but a synonym of this disease might. Furthermore, a disease database might not contain information about the specific diagnosis, but a broader description of the diagnosis might be linked to a causative gene.

In this work we applied an approach that considers both problems. In a first step we extracted all disease terms from the patient records with our literature mining tool (automatically from the PDF files containing the medical reports). These terms were collected from MESH (Medical Subject Headings). Then we specified the primary diagnosis and in addition listed all secondary diagnoses for which we assumed an independent genetic cause.

Given these medical terms (primary and secondary diagnoses) we employed an ontology based search strategy that operates on expert-curated and/or LitInspector based annotations. The obvious first step was to search directly for the diagnosis in expert-curated gene-disease databases. If this strategy directly linked a variant to a known disease association, the search could be terminated at this point. In some cases, however, the direct link between a certain gene and the specific diagnosis is unknown or has not been annotated in gene-disease databases yet. In this case the search was broadened to a more general medical term, that is, the parent term within the disease ontology (here MESH). This process was reiterated until a causative variant is found or a level in the disease ontology was reached that was too broad. In addition to the ontology guided search, we switched between different annotation bodies (CeGaT panels, expert-curated annotations (OMIM) and the LitInspector based gene-disease database). Our search

strategy first aimed at linking the disease through the expert-curated databases and if this failed, attempted to link the variants to the LitInspector based disease annotations.

Results for families 1 - 3

Variant statistics

For the SOLiD data we applied multiple mapping and variant calling strategies, and were provided with variants from the CompleteGenomics data. In this section we focus on the SAMtools variant calls as an example for one successful pipeline. The statistical results of other pipelines might differ significantly in terms of the raw numbers (e.g., WES vs. WGS), but in later sections we will show that the final results are identical in most cases.

Sample	SNVs	INDELs	non-synonymous	Ti/Tv	hom	het	ref
W1-1	215565	7467	11966	2.3	144825	78207	32464
W1-2	192344	6733	11722	2.3	129701	69376	31934
W1-3	203541	7051	12025	2.4	135987	74605	32913
W2-1	200564	6953	10329	2.3	116995	90522	77601
W2-2	143170	5424	9963	2.3	85373	63221	69614
W2-3	169479	6075	10003	2.3	99638	75916	77014
W2-4	146418	5599	9975	2.3	87710	64307	69624
W2-5	127835	4960	9768	2.3	76683	56112	67955
W2-6	151224	5513	10008	2.3	91774	64963	70570
W3-1	141914	5444	11945	2.4	89373	57985	27762
W3-2	135569	5031	11795	2.4	84796	55804	28431
W3-3	138549	4896	11932	2.4	89535	53910	27209

Table 2: Variant calling statistics. For each sample the number of called variants with SAMtools is listed. Non-synonymous variants are those that affect the protein sequence. The Ti/Tv column specified the transition/transversion ratio. The last three columns specify the genotype of the SNP. In case of ref, a variant called in an other family member was detected as reference in the sample.

In Table 2 the variant calls from SAMtools are summarized for each sample. These numbers include all called variants, even those with low quality. Local coverage biases in the flanking regions of the enrichments might lead to numerous non-overlapping variant calls between different samples. To avoid removal of any true positive (TP) calls at this stage, we did not apply any quality based filtering. After applying all filters, the latter is only considered when there are too many potential candidates for an expert to investigate manually.

Figure 1 gives an overview of the filtering steps and shows how many variants remain after each stage. The first filter removes all intergenic variants and has a filter factor of 1.7 (58 %). For the cases in this challenge we expected a strong effect on the protein structure and thus only considered non-synonymous variants and variants that lay in a canonical splice-site. The second filter removes all variants that do not directly alter the protein structure. This filter has a strong factor of 23.6, which means that on average only 1 of 23.6 variants remain after this filter. The next filter is the genotype (GT) filter. This filter depends on the medical history and the derived inheritance pattern. The GT filter also has a strong filter factor of 23.5. The next step requires a variant to have a GAF (1000 Genomes Project and CeGaT DB) score lower than 0.05. This filter removes 78% (filter factor 4.5) of the remaining variants. The last filtering step is the disease filter. The specific disease terms and annotations used for this filtering step are described for each family in the next section. The disease filter has the strongest filter factor of all (only 1 in 70 variants remain after the filtering), but it strongly depends on the disease and the specific disease term. Overall, including the GT filter, we could remove 99.99979 % of all called variants and 99.9943 % of all variants that fall on a known transcript.

To give us an indication of how many variants would have been obtained had the relatives not been sequenced, we filtered without utilizing the GT filter. This yields 101 (for W1), 44 (W2) and 90 (W3) candidate genes.

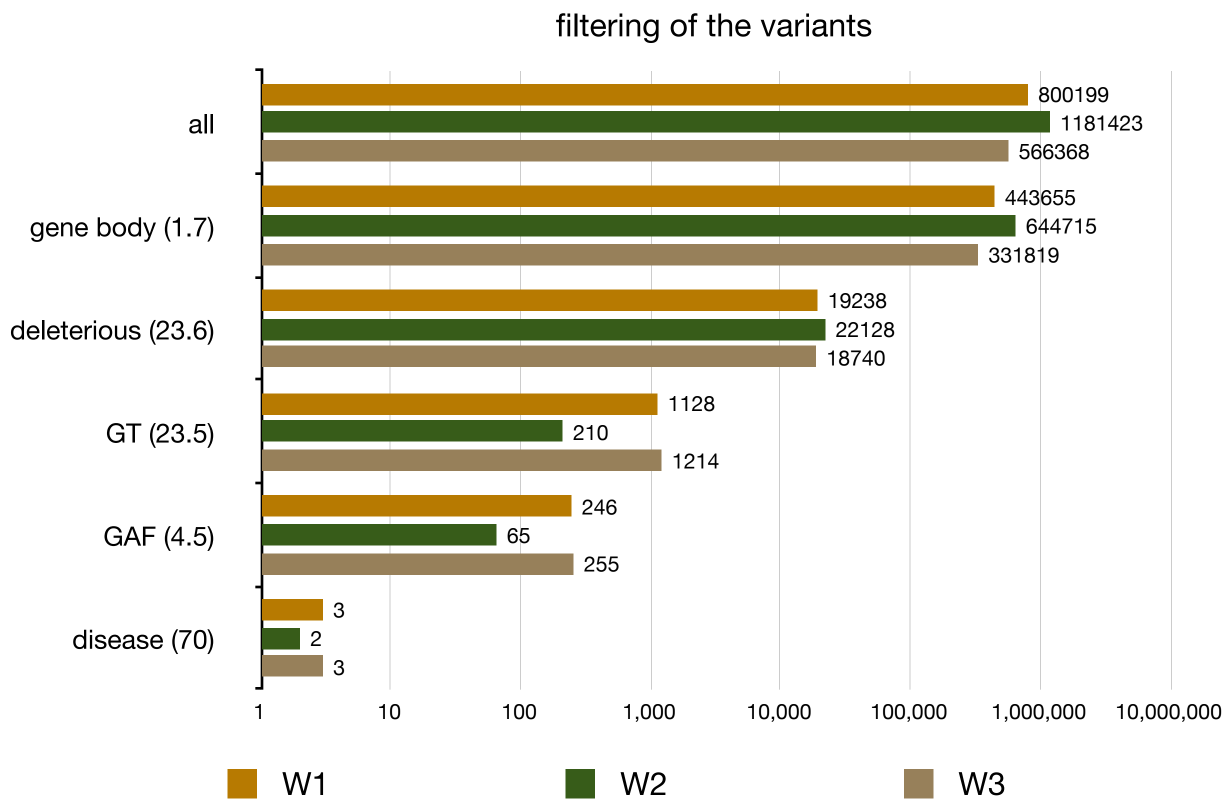

Figure 1: Filtering of variants for W1, W2 and W3 (log scale). The first bars (all) show the total of variants called in any of the family members. For each subsequent filtering step the number of remaining variants and the filter factor is given. The filters are: gene body (variants that overlap with a transcript), deleterious (variants that alter the protein sequence or hit a canonical splice-site), GT (genotype filter derived from the medical report), GAF (1000 Genomes Project background filter), disease (filter for the primary disease and MESH parents from the medical report).

Applying different disease filter settings

Family W1 (Centronuclear myopathy and hearing loss)

The primary diagnosis in family 1 is centronuclear myopathy with a secondary diagnosis of sensorineural hearing loss. The latter was derived from the health problems that were listed for the affected child (W1-1). Although several additional health problems are reported, it stands to reason that most of them are either secondary effects of the centronuclear

myopathy (e.g., reflux), or do not have a simple genetic background (e.g., seasonal allergies).

W1 - primary diag	DB	primary diagnosis	MESH (synonym)	MESH (1 step)	panel
terms		centronuclear myopathy	myopathy	muscular diseases	NMD
de novo (1 genes)	no disease associations found				
compound heterozygous (44 genes)	OMIM	none	**TTN**	none	**TTN**
	LitInspector	none	**TTN**,ALMS1	**TTN**	
homozygous (34 genes)	OMIM	none	none	none	none
	LitInspector	none	none	none	none
x-linked (1 gene)	no disease associations found				

Table 3: Disease associations for the primary diagnosis of family W1; in this case a compound heterozygous mutation is reported for TTN, which is found through OMIM, the NLP based annotations and the CeGaT NMD panel.

As shown in Table 3, we applied different disease annotation filters to the genes that match the genotype and population frequency criteria. For *de novo* and x-linked mutations one gene was found and for homozygous we found 34 genes, but in all cases no disease association to our medical terms was found. For the compound heterozygous case 44 genes were found, but with no association to the primary diagnosis. In OMIM, for instance, only three genes have so far been linked to *centronuclear myopathy* (DNM2, MYF6 and BIN1), but these genes are not contained in the filtered cases. According to MESH, centronuclear myopathy is a synonym for myopathy[1]. Searching with all synonyms, including the term *myopathy, we* linked TTN to the disease with both OMIM and the LitInspector based annotations. TTN is also present in the CeGaT panel for neuromuscular diseases (NMD). The LitInspector based diseases contain one additional links to ALMS1, which was not considered to be the cause for the primary diagnosis by our medical experts.

[1] http://www.nlm.nih.gov/cgi/mesh/2012/MB_cgi

W1 - hearing loss	DB	secondary diagnosis	MESH (1 step)	MESH (2 steps)	panel
terms		sensorineural hearing loss	hearing loss	hearing disorders	hearing
de novo (1 genes)	no disease associations found				
compound heterozygous (44 genes)	OMIM	none	none	none	**GJB2**
	LitInspector	**GJB2**	**GJB2**,RAI1,ALMS1	none	
autosomal recessive (34 genes)	OMIM	none	none	none	none
	LitInspector	none	none	none	
x-linked (1 gene)	no disease associations found				

Table 4: This table shows the disease associations for the secondary diagnosis of family W1 (sensorineural hearing loss). In this case a compound heterozygous mutation is reported for GJB2, which is found in the LitInspector based annotations using the primary diagnosis and the CeGaT NMD panel. This association is not found in OMIM.

The secondary diagnosis of sensorineural hearing loss can be directly linked to a compound heterozygous variant in the GJB2 gene through the LitInspector based annotations and the hereditary hearing loss panel from CeGaT (Table 4). In this case the term from the primary diagnosis links exclusively to GJB2. The details of the GJB2 variants are discussed in the medical report.

Family W2 (RBBB and pulmonary stenosis)

Family W2 is the most complex case in this challenge, with two family trios, where the trio parents are siblings (Figure 2). The primary diagnosis and reported health problems are different between the affected cases. W2-1 has the primary diagnosis cardiac defects with right bundle block branch (RBBB). RBBB is also the primary diagnosis of patient W2-2. The primary diagnosis of W2-4 and W2-6 includes pulmonary stenosis and Atrioventricular (AV) block. Thus, all affected patients can either be related to bundle-branch block or AV block. In the MESH tree, both *bundle-branch block* and *AV (Atrioventricular) block* are contained in *heart block*. Accordingly, when searching for variants shared by all affected patients we linked the genes to *bundle-branch block* or *AV block* as primary search term

and then broadened the search to *heart block*, which is the parent of both. For the additional diagnosis of pulmonary stenosis in patients W2-4 and W2-6 we conducted a separate search, because an additional factor might be causing the more severe health problems of W2-6 and W2-4 (deceased). In this case, we started the search with the primary diagnosis of pulmonary stenosis.

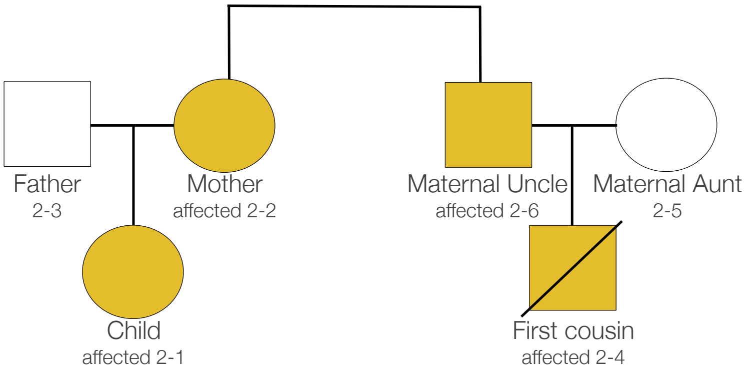

Figure 2: Family history of family 2.

W2 - RBBB	DB	diagnosis	MESH (1 step)	MESH (2 steps)	panel
terms		Bundle-Branch Block or AV Block	heart block	cardiac arrhythmias	HED
autosomal dominant (47 genes)	OMIM	none	**TRPM4**	none	**TRPM4**
	LitInspector	none	none	**TRPM4**,PHB	**TRPM4**

Table 5: This table shows the disease associations for the most common diagnosis of family W2 (RBBB - bundle-branch block and AV Block). In this case a heterozygous mutation is reported for TRPM4, which is found in the OMIM based annotations using the MESH parent of the primary diagnosis, in LitInspector using the second MESH parent and in the CeGaT HED panel.

The first search was for the causative variant for Bundle-Branch Block (RBBB) in all affected patients. After applying the phenotype and GAF filter 47 genes remained. Of these none could be linked to *Bundle-Branch Block*. This is not surprising as this term is not contained in OMIM or LitInspector. Broadening the search to *heart block* and *cardiac arrhythmias*, however, gave a hit for *TRPM4* in OMIM, *TRPM4* and PHB in LitInspector and *TRPM4* in an expert generated gene set of CeGaT for heart diseases (HED panel) (Table 5).

TRPM4 is discussed in detail in the medical report. *PHB* was was linked to cardiac arrhythmias. Although this mutation is in a conserved region and has a low BLOSUM62 score (-2; p.R43L). To the best of our knowledge, it has not been reported to play a role in heart block, and thus we did not consider it for the medical report.

W2 - stenosis	DB	diagnosis	MESH (1 + 2 steps)	tissue	panel
terms		pulmonary stenosis	Ventricular Outflow Obstruction or Heart Valve Diseases or Heart Diseases	*heart ventricles*	
autosomal dominant (188 genes)	OMIM	none	none	-	-
	LitInspector	none	PPARA, NOS3	**SMYD1**, NOS3, CHGB, CS	-

Table 6: This table shows the disease associations for the primary diagnosis of patients W2-4 and W2-6 (pulmonary stenosis). In this case no candidate gene was found when filtering for the diagnosis terms. When broadening the search to the LitInspector tissue filter (***heart ventricles***) we found a potential candidate gene (SMYD1).

The suspected genetic factor for the second search was not detected through our standard search strategy. To broaden the search to genes that lack extensive knowledge on their medical roles, we applied a tissue based filtering strategy. The tissues we selected for filtering were *heart ventricles.* The term *heart ventricles* led to the following findings ordered by GAF score: *SMYD1*, *NOS3*, *CHGB*, *CS*. Of these genes *CHGB* and *CS* have high GAF scores (0.03 and 0.04 respectively) and high BLOSUM62 scores (1 and 3 respectively). The variant in *NOS3* had a very low coverage. *SMYD1*, on the other hand, had sufficient coverage, is a novel SNV and is contained in a highly conserved region. In a recent publication Sirinupong *et al.* (2010) describe the role of *SMYD1* in early heart development:

> *"SMYD1 is a cardiac- and muscle specific histone methyltransferase that methylates histone H3 at lysine 4 and regulates gene transcription in early heart development." (Sirinupong et al, JBC, 2010)*

Although this SNV might play a role in early heart development, this finding is very speculative and will not be included into the medical report.

Family W3 (Nemaline myopathy)

The primary diagnosis for the child (W3-1) in family W3 is nemaline myopathy, whereas both parents are unaffected. In the MESH ontology *nemaline myopathy* is contained in the entity *centronuclear myopathy*, which in turn is connected to the parent term *muscular diseases*. As in case W1 the primary diagnosis could not be linked to any gene in which we found candidate variants, but we could link *TTN* to the term *myopathy* (synonym for *centronuclear myopathy*) . When searching for *muscular diseases* we could link *obscurin* (*OBSCN*) to this case (Table 7).

W3 - primary diag	DB	primary diagnosis	MESH (1 step)	MESH (2 steps)	panel
terms		nemaline myopathy	myopathy	muscular diseases	NMD
de novo mutations *(5 genes)*	*OMIM*	none	none	none	none
	LitInspector	none	none	none	
compound heterozygous (40 genes)	OMIM	none	TTN	TTN	TTN
	LitInspector	none	TTN	TTN/**OBSCN**	
autosomal recessive (29 genes)	OMIM	none	none	none	none
	LitInspector	none	none	none	
x-linked	*no genes found*				

Table 7: This table shows the disease associations for the primary diagnosis of patient W3-1 (nemaline myopathy). In this case a compound heterozygous mutation is reported for TTN and OBSCN. As in W1 TTN could be linked to this case through all disease annotations. OBSCN, however, could only be found using the LitInspector based annotations.

Obscurin would not have been found when only applying our expert-curated databases. The compound heterozygous mutations in obscurin might be the cause for the nemaline myopathy in W3-1 and the variants found in *TTN* might also have an effect. We identified the heterozygous mutation in exon 283 of the *TTN*-gene, which has to the best of our knowledge not been described in the literature. The mutation in exon 283 of the *TTN*-gene is very likely pathogenic, it leads to a premature stop codon. This severely truncates the

TTN protein and leads to a loss of the obscurin binding site. Further details are given in the medical report.

Details on potential disease causing variants

In the last section we showed how we linked disease annotations to potentially causative variants on a gene level. Details about the underlying variants can be found in Table 8. In total this table lists six genes and nine variants (where *TTN* occurs twice). Half of these genes contain compound heterozygous variants, in which one mutation is paternal and one maternal. Most (seven of nine) of the variants observed were missense, causing a single amino acid substitution. One surprising observation is that seven of nine variants have not been observed in the 1000 Genomes Project population and have not been reported in dbSNP. If this finding can be generalized it has strong implications on the way variants are linked to diseases. If one would have applied a direct linking of variants to diseases, almost all of the variants would have been overlooked.

Comparing different variant approaches

Within this challenge we applied different mapping, variant calling and variant annotation approaches to the provided datasets. We do not intend to provide an extensive comparative analysis of the applied methods, instead we focus on the reported variants (see Table 9). Overall, DiBayes and CompleteGenomics were the only approaches that detected all variants. SAMtools missed one variant in the compound heterozygous GJB2 mutation, whereas GATK missed multiple variants in this gene. When running SAMtools on the individual samples, however, all variants were detected.

This comparative analysis was helpful to ensure that we did not overlook variants, and to see that most variants are called accordingly. However, we refrain from drawing any general conclusions from these findings.

position	category	GAF	coverage (WES)	genotype
**TTN** W1 (1-1 affected) - cause for centronuclear myopathy (primary diagnosis)<br>Compound heterozygous				
chr2 179487495	canonical splice-site	never observed	38/28/32	1-1 (het) 1-2 (het)
chr2 179506964	missense, splice-site	never observed*	37/31/35	1-1 (het) 1-3 (het)
**GJB2** W1 (1-1 affected) - cause for sensorineural hearing loss (secondary diagnosis)<br>Compound heterozygous				
chr13 20763620	missense	1%	66/53/92	1-1 (het) 1-2 (het)
chr13 20763685	frameshift (deletion)	never observed*	NA	1-1 (het) 1-3 (het)
**TRPM4** W2 (2-1, 2-2, 2-4 and 2-6 affected) - cause for Cardiac defects (primary diagnosis)				
chr19 49671571	missense	never observed	9/15/27/28/32/35	2-1 2-2 2-4 2-6 (all het)
**OBSCN** W3 (3-1 affected) - cause for nemaline myopathy (primary diagnosis)<br>Compound heterozygous				
chr1 228432113	missense	never observed	126/102/166	3-1 (het) 3-2 (het)
chr1 228404271	missense	never observed	41/54/78	3-1 (het) 3-3 (het)
**TTN** W3 (3-1 affected) - cause for nemaline myopathy (primary diagnosis)				
chr2 179418785	nonsense	never observed	44/29/28	3-1 (het) 3-3 (het)

*Table 8: List of potential disease causing variants. The listed variants were included in the medical reports. We found three compound heterozygous cases, two cases of canonical-splicing disruption, one nonsense mutation and several missense mutations. The term “never observed” refers to the 1000 Genomes Project, here one of the detected variants was observed in the background populations. The coverages are listed ordered by the patient numbers (from WES). *Although these variants have never been observed in the 1000 Genomes Project, they have been reported in literature.*

Variants	DiBayes	SAMtools	GATK	CompleteGenomics
**TTN** W1 (1-1 affected) - cause for centronuclear myopathy (primary diagnosis)				
**Compound heterozygous**	found	found	found	found
chr2 179487495	found in W1-1,W1-2	found in W1-1,W1-2	found in W1-1,W1-2	found in W1-1,W1-2
chr2 179506964	found in W1-1,W1-3	found in W1-1,W1-3*	found in W1-1,W1-3	found in W1-1,W1-3
**GJB2** W1 (1-1 affected) - cause for sensorineural hearing loss (secondary diagnosis)				
**Compound heterozygous**	found	found*	not found	found
chr13 20763620	found in W1-1,W1-2	found in W1-1,W1-2	not found	found in W1-1,W1-2
chr13 20763685	found in W1-1,W1-3	found in W1-1,W1-3*	found in W1-1 (not 1-3)	found in W1-1,W1-3
**TRPM4** W2 (2-1, 2-2, 2-4 and 2-6 affected) - cause for Cardiac defects (primary diagnosis)				
chr19 49671571	found W2-1,2-2,2-4,2-6	found W2-1,2-2,2-4,2-6	found W2-1,2-2,2-4,2-6	found W2-1,2-2,2-4,2-6
**OBSCN** W3 (3-1 affected) - cause for nemaline myopathy (primary diagnosis)				
**Compound heterozygous**	found	found	found	found
chr1 228432113	found in 3-1,3-2	found in 3-1,3-2	found in W3-1,W3-2	found in W3-1,W3-2
chr1 228404271	found in 3-1,3-3	found in 3-1,3-3	found in W3-1,W3-3	found in W3-1,W3-3
**TTN** W3 (3-1 affected) - cause for nemaline myopathy (primary diagnosis)				
chr2 179418785	found in W3-1,W3-3	found in W3-1,W3-3	found in W3-1,W3-3	found in W3-1,W3-3

Table 9: List of the putative causal variants and which variant callers detected them. DiBayes and CompleteGenomics detected all variants (green). SAMtools missed variants in the gene GJB2 when run in the default trio mode. Applying SAMtools to the individual samples (with the -R option) provided all variant calls (yellow). GATK missed variants in the GJB2 genes (red), thus the compound heterozygous case was overlooked. For variant caller settings see Section 2.

geneGrid: a novel tool for comparative variant analysis

To analyze the dataset provided in the CLARITY challenge we employed a novel tool for comparative variant analysis, termed geneGrid. The infrastructure of geneGrid is a two-tier client/server system. On the server side a database (DB) stores all variants, pre-compiled annotations and user annotations. The client side is web-based and requires a URL and login. Data requests from the client are computed directly on the DB, ensuring reliable and fast filtering of the variant and annotation data. Through a versatile table based interface the user can fully customize the filter criteria or rely on the predefined filtering options (Figure 3). Currently, we are extending geneGrid to include a reporting option for the detected variants. For the CLARITY challenge this setup was only used in an restricted internal network.

The data model is designed to keep the variants separate from the annotations. This allows the reanalysis of cases whenever updates for pre-built annotations or novel user-annotations are available.

To show how geneGrid was applied in the CLARITY challenge, we supplemented a short video demonstrating how one can detect the variants in W2 and W3.

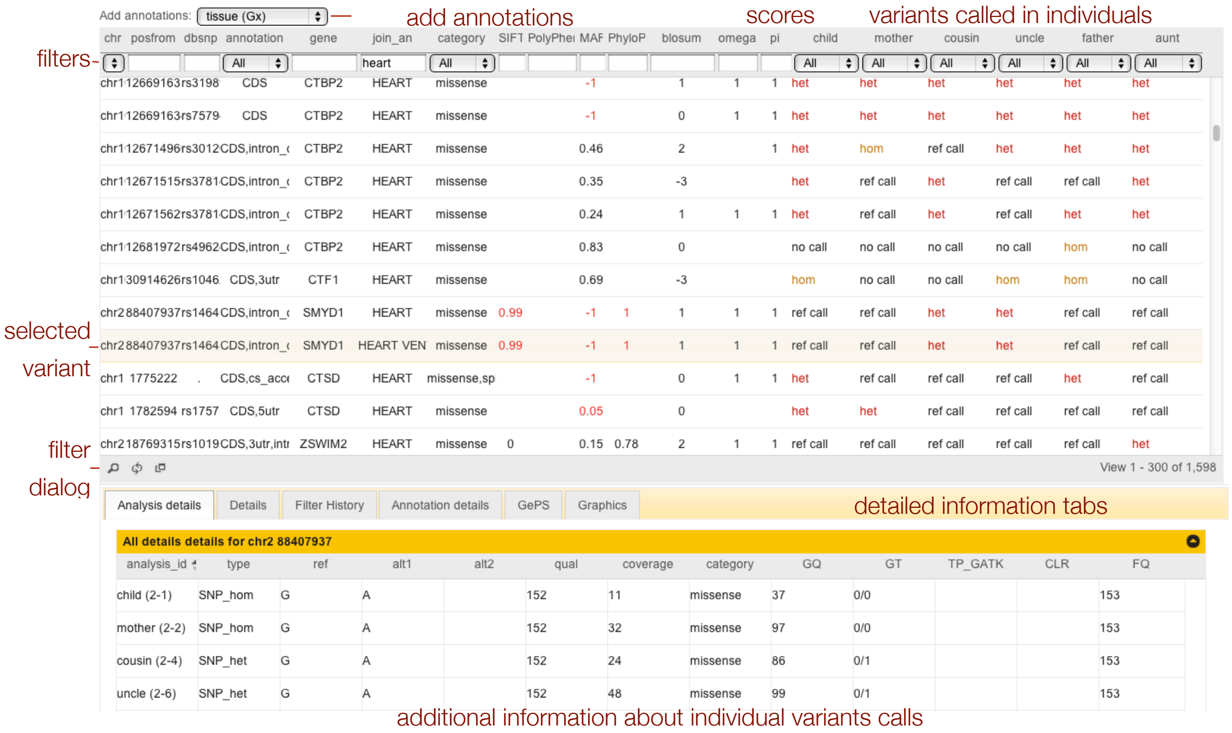

Figure 3: GeneGrid interface. The main table contains all variants called in family W2. It displays variant information like position, dbSNP, annotations, scores and the variant calls in the individuals. The user can filter for any combination of columns, either directly through the column field or in a specific filter dialog. In the right lower corner the total number of variants passing the current filter is displayed (tissue: heart). The annotation can be selected from the drop-down dialog at the top. The table at the bottom contains detailed information about individual variants (e.g., coverage). The information display in this area can be switched to other types of additional information (e.g., variant effects or annotation details)

Conclusions & recommendations

Years of clinical research have led to a wealth of information about disease causing variants. Thus, when an individual is diagnosed with a syndrome for which disease causing variants have been described, these can be investigated through targeted sequencing. In the case of centronuclear myopathy, for instance, variants in *MTM1* and *BIN1* have been described as disease causing SNVs. In the cases provided in the CLARITY challenge, however, the targeted approach did not uncover any causal variant.

If the causal variant is not found in a targeted approach, high-throughput sequencing of the exome (WES) or genome (WGS) provides a technology to detect variants on a genomic scale. Although this approach is likely to detect the causal variant, it will also detect thousands (WES) or millions (WGS) of additional variants (mostly silent). This shifts the problem from the detection of causal variants to the problem of finding the causal variants. Pedigree analysis in combination with medical history, background populations and disease annotations provide a powerful information basis to filter all silent mutations and find the disease causing variant(s). The use of these filters, however, depends on the study design that can be roughly divided into three groups: sequencing one affected individual, sequencing groups of affected and unaffected individuals and sequencing families.

Within the CLARITY challenge, the genomes of three families with suggested inherited diseases were sequenced. This study design has a very favorable property of allowing for a genotype filter and matching the findings against the clinical reports. Thus, we think that for previously unreported variant-disease associations the pedigree setup chosen in the CLARITY challenge is the most promising.

As sequencing platforms, CompleteGenomics and LIFE (SOLiD 5) were applied. For the LIFE data we applied several mapping and variant calling strategies, whereas the variant calls where directly provided from CompleteGenomics. All reported variants were detected through both platforms. We observed individual cases, in which a certain variant caller overlooked a variant, but based on the small sample size we cannot make any solid suggestions about the platform or variant detection.

The filtering based on the clinical report and background population databases (1000 Genomes Project and CeGaT in-house DB), allowed us to remove polymorphisms and

different types of errors (e.g., due to the reference genome, sequencing or mapping). To relate the remaining variants to diseases, public (OMIM) and in-house (CeGaT) expert-curated, as well as literature data mining based (LitInspector) annotations were applied. For the cases discussed here, we can show that expert-curated databases cover most variant-disease relations. However, in some novel cases the LitInspector annotations are needed (e.g., *OBSCN* in case W3). In addition to the annotation, the problem of linking diagnosis terms to annotation database entries exists. We resolved this problem through synonym resolution and the MESH disease ontology. This allowed us to search at different levels of detail and uncover gene-disease relations that have only been described in a more general context (e.g., *OBSCN* - myopathy).

All these filters and analysis steps have been integrated into a client-server based infrastructure, which allows medical staff controlled access the molecular results. In addition, filters can directly be applied to the data and (custom) annotations can be dynamically overlaid with the variants. Overall, this interface allows one to perform the variant analysis by filtering out large amounts of putative non-causal SNVs (99.9943 %). It thereby provides all variant and annotation based information to facilitate the writing of a medical report.

Supplements

I.Medical reports for all families
Medical_report_W<n>.pdf (W1, W2 & W3)

II.Webcast demonstration of geneGrid for all families
geneGrid_Screencast.mov

III.Molecular results
CLARITY_challenge_MolecularResults.pdf

IV.List of all variants that pass our filtering criteria
W<n>_filtered_variants_WES.xls & W<n>_filtered_variants_WGS.xls (W1, W2 & W3)

References

(Adzhubei *et al.* 2010) Adzhubei IA *et al.* (2010) A method and server for predicting damaging missense mutations. Nat Meth 7, 248-249.

(Cartharius *et al.* 2005) Cartharius K, Frech K, Grote K, Klocke B, Haltmeier M, Klingenhoff A, Frisch M, Bayerlein M, Werner T (2005) MatInspector and beyond: promoter analysis based on transcription factor binding sites. Bioinformatics 21: 2933-42.

(Cooper *et al.* 2005) Cooper GM *et al.* (2005) Distribution and intensity of constraint in mammalian genomic sequence. Genome Research 15, 901-913

(DePristo *et al.* 2011) DePristo MA *et al.* (2011) A framework for variation discovery and genotyping using next-generation DNA sequencing data. Nat Genet 43, 491-498.

(Drmanac *et al.* 2010) Drmanac R *et al.* (2010) Human genome sequencing using unchained base reads on self-assembling DNA nanoarrays. Science (New York, N.Y.) 327, 78-81.

(Durbin *et al.* 2010) Durbin RM *et al.* (2010) 1000 Genomes Project Consortium. A map of human genome variation from population-scale sequencing. Nature 467, 1061-1073.

(Frazer *et al.* 2007) Frazer K.A. *et al.* (2007) The International HapMap Consortium. A second generation human haplotype map of over 3.1 million SNPs. Nature 449, 851-861.

(Frisch *et al.* 2009) Frisch M, Klocke B, Haltmeier M, Frech K (2009) LitInspector: literature and signal transduction pathway mining in PubMed abstracts. Nucleic Acids Res. 1;37: W135-40.

(Galperin *et al.* 2012) Galperin MY & Fernández-Suárez XM (2012) The 2012 nucleic acids research database issue and the online molecular biology database collection. Nucleic Acids Research 40, D1-D8.

(Grant *et al.* 2011) Grant JR, Arantes AS, Liao X, Stothard, P (2011) In-depth annotation of SNPs arising from resequencing projects using NGS-SNP. Bioinformatics (Oxford, England) 27, 2300-2301.

(Hamosh *et al.* 2005) Hamosh A, Scott AF, Amberger JS, Bocchini CA, Mckusick, VA (2005) Online mendelian inheritance in man (OMIM), a knowledgebase of human genes and genetic disorders. Nucleic acids research 33.

(Hebsgaard *et al.* 1996) Hebsgaard SM *et al.* (1996) Splice site prediction in arabidopsis thaliana pre-mRNA by combining local and global sequence information. Nucleic acids research 24, 3439-3452.

(Henikoff *et al.* 1992) Henikoff S & Henikoff JG (1992) Amino acid substitution matrices from protein blocks. Proceedings of the National Academy of Sciences 89, 10915-10919.

(Homer *et al.* 2009) Homer N, Merriman B, Nelson SF (2009) BFAST: An alignment tool for large scale genome resequencing. PLoS ONE 4(11):e7767.

(Kumar *et al.* 2009) Kumar P, Henikoff S, Ng PC (2009) Predicting the effects of coding non-synonymous variants on protein function using the SIFT algorithm. Nature protocols4, 1073-1081.

(Li *et al.* 2009) Li H *et al.* (2009) The sequence Alignment/Map format and SAMtools. Bioinformatics 25, 2078-2079.

(Lindblad-Toh *et al.* 2011) Lindblad-Toh K *et al.* (2011) A high-resolution map of human evolutionary constraint using 29 mammals. Nature 478, 476-482.

(Nielson *et al.* 2011) Nielsen R, Paul JS, Albrechtsen A, Song YS (2011) Genotype and SNP calling from next-generation sequencing data. Nat Rev Genet 12, 443-451.

(Rogers et al. 1963) Rogers FB (1963) Medical subject headings. Bull Med Libr Assoc 51:114-116.

(Schwarz et al. 2010) Schwarz JM, Rodelsperger C, Schuelke M, Seelow D. (2010) Mutation-Taster evaluates disease-causing potential of sequence alterations. Nat Meth 7, 575-576.

(Sherry *et al.* 2000) Sherry ST *et al.* (2001) dbSNP: the NCBI database of genetic variation. Nucleic Acids Research 29, 308-311

(Sirinupong *et al.* 2010) Sirinupong N, Brunzelle J, Ye J, Pirzada A, Nico L, Yang Z (2010) Crystal structure of cardiac-specific histone methyltransferase SmyD1 reveals unusual active site architecture.J Biol Chem. 24;285(52):40635-44.

(Stenson *et al.* 2009) Stenson P D. *et al.* (2009) The human gene mutation database: 2008 update. Genome Med 2009, 1:13.

(Tang *et al. 2008*) Tang, S. et al. DiBayes: A SNP detection algorithm for Next-Generation di-base sequencing. URL: www3.appliedbiosystems.com/cms/groups/mcb_marketing/documents/generaldocuments/cms_057817.pdf.

(Quandt *et al.* 1995) Quandt K, Frech K, Karas H, Wingender E, Werner T (1995) MatInd and MatInspector: new fast and versatile tools for detection of consensus matches in nucleotide sequence data. Nucleic Acids Res. 23: 4878-84.
