# Supplementary material for: An international effort towards developing standards for best practices in analysis, interpretation and reporting of clinical genome sequencing results in the CLARITY Challenge
Source: Genome Biol. 2014 Mar 25;15(3):R53. doi: 10.1186/gb-2014-15-3-r53 (PMC4073084; doi:10.1186/gb-2014-15-3-r53)
Supplement: Additional file 2 — The entry from the Genomatix/CeGaT/University Hospital of Bonn team containing five PDF files and six XLS tables. [file gb-2014-15-3-r53-S2.zip › Additional_file_2/CLARITY_challenge_MolecularResults.pdf]

Molecular Results Summary - CLARITY Challenge

Listed below are all variants reported by our team and associated with the known medical conditions. For a detailed interpretation of these results please refer to the medical reports. All variants have been found in a heterozygous state.

family **W1**

gene	mutation	location	functional class	carriers	known variation
TTN	c.35635G>C; p.V11879L	chr2 179506964	missense & splice-site	W1-1, W1-3	Pathogenic (Herman *et al.* 2012)
TTN	c.39893-1G>A; p.?	chr2 179487495	splice-site	W1-1, W1-2	no
GJB2	c.101T>C; p.M34T	chr13 20763620	missense	W1-1, W1-2	rs35887622 known pathogenic
GJB2	c.35delG; p.G12Vfs*2	chr13 20763686	frameshift	W1-1, W1-3	rs80338939 known pathogenic

family **W2**

gene	mutation	location	functional class	carriers	known variation
TRPM4	c.503T>A; p.V168E	chr19 49671571	missense	W2-1, W2-2, W2-4, W2-6	no

family **W3**

gene	mutation	location	functional class	carriers	known variation
TTN	c.84130A>T; p.K28044X	chr2:179418785	nonsense	W3-1, W3-3	no
OBSCN	c.2245G>T; p.G749C	chr1:228404271	missense	W3-1, W3-3	no
OBSCN	c.3322T>A; p.Y1108N	chr1:228432113	missense	W3-1, W3-2	rs199696332
