# Supplementary material for: An international effort towards developing standards for best practices in analysis, interpretation and reporting of clinical genome sequencing results in the CLARITY Challenge
Source: Genome Biol. 2014 Mar 25;15(3):R53. doi: 10.1186/gb-2014-15-3-r53 (PMC4073084; doi:10.1186/gb-2014-15-3-r53)
Supplement: Additional file 2 — The entry from the Genomatix/CeGaT/University Hospital of Bonn team containing five PDF files and six XLS tables. [file gb-2014-15-3-r53-S2.zip › Additional_file_2/Medical_report_W1.pdf]

CeGaT & Praxis für Humangenetik, Paul-Ehrlich-Str. 17, 72076 Tübingen

Center for Genomics
and Transcriptomics

CeGaT GmbH &
Praxis für Humangenetik
Paul-Ehrlich-Str. 17
D-72076 Tübingen
www.cegat.de
Tel: +49 7071 5654400
saskia.biskup@cegat.de

The CLARITY Team

09/25/2012

Results of Next Generation Sequencing (NGS) diagnostics - CLARITY Challenge

**Patient**	**dob.**	**Pat. No.**	**Material**	***TTN* Mutation**
Family1, Affected 1-1	undisclosed	W1-1	sequencing data	**c.[35635G>C]+[39893-1G>A]; p.[V11879L]+[?]**
Family1, Mother 1-2	undisclosed	W1-2	sequencing data	**c.[39893-1G>A]+[=]; p.[?]+[=]**
Family1, Father 1-3	undisclosed	W1-3	sequencing data	**c.[35635G>C]+[=]; p.[V11879L]+[=]**

**Patient**	**dob.**	**Pat. No.**	**Material**	***GJB2* Mutation**
Family1, Affected 1-1	undisclosed	W1-1	sequencing data	**c.[101T>C]+[35delG]; p.[M34T]+[G12Vfs*2]**
Family1, Mother 1-2	undisclosed	W1-2	sequencing data	**c.[101T>C]+[=]; p.[M34T]+[=]**
Family1, Father 1-3	undisclosed	W1-3	sequencing data	**c.[35delG]+[=]; p.[G12Vfs*2] +[=]**

Dear CLARITY Team,

Thank you very much for giving us the opportunity to analyze the NGS data of the family mentioned above with respect to the clinical findings of centronuclear myopathy and sensorineural hearing loss in the index patient (W1-1). We received the data of the index patient and the parents (W1-1, W1-2 and W1-3) on 06/06/2012.

Methods: We applied different strategies (see detailed report) to search for de novo mutations in the dataset of the index patient (W1-1) as well as for genes containing a homozygous or compound heterozygous mutation. We used the datasets of the parents (W1-2 and W1-3) for filtering steps and for segregation analysis.

Results with respect to centronuclear myopathy: In your patient (W1-1) we detected the **compound heterozygous splice site mutations c.35635G>C; p.V11879L** and **c.39893-1G>A; p.? in exon 169 and intron 192 of the *TTN* gene** (Genbank: NM_001256850.1; NC_000002.11) using the whole exome dataset. In the patient's mother (W1-2) we detected the mutation **c.39893-1G>A; p.? in intron 192 of the *TTN* gene**, whereas the patient's father (W1-3) carries the mutation **c.35635G>C; p.V11879L in exon 169 of the *TTN* gene.** These results have been confirmed with the whole genome sequencing data of family members (W1-1, W1-2 and W1-3). As there is no DNA available to us, the mutations can not be validated by Sanger Sequencing. We strongly recommend to validate NGS data by conventional methods.

Interpretation of *TTN* gene variants: The centronuclear myopathy in your patient (W1-1) could be due to compound heterozygous mutations within the *TTN* gene. We identified the mutations c.35635G>C; p.V11879L and c.39893-1G>A; p.? in exon 169 and intron 192 of the *TTN* gene. The literature describes the mutation c.35635G>C; p.V11879L as cause of dilated cardiomyopathy (Herman *et. al.*, N Eng J Med 2012). The mutation c.35635G>C; p.V11879L changes the last position of exon 169. This leads to an alteration of a conserved amino acid and disrupts the donor splice site. The prediction programs “MutationTaster” and “NetGene2 Server” found the mutation to cause a loss of the splice site, probably leading to a dysfunctional protein. The mutation c.39893-1G>A; p.? has not yet been described in the literature. It changes the last position of intron 192, disrupting the acceptor splice site. The prediction programs “MutationTaster” and “NetGene2 Server” found the mutation to cause a loss of the splice site, probably leading to a dysfunctional protein.

Mutations in both *TTN* alleles have been described to cause early-onset myopathy with fatal cardiomyopathy (OMIM #611705). Muscle biopsies of these patients fit to the pathological evaluation of your patient's muscle biopsy. However, it is not clear whether the benign congenital heart murmur of your patient W1-1 could relate to the detected *TTN* mutations. It is important to mention that the mutations in your patient lie in a different domain of the TTN protein compared to the mutations mentioned in entry OMIM #611705. Thus, a prognosis for your patient can not be made with confidence based on OMIM #611705. It is of note, that the mutation c.35635G>C; p.V11879L has already been described in compound heterozygosity with another splice site mutation c.54704-1G>A; p.? (Herman *et. al.*, N Eng J Med 2012). The observed phenotype in the reported patient is severe dilated cardiomyopathy. However, the effects of splice site mutations are not easy to predict on protein level. Thus, it is not unlikely that different phenotypes are observed.

Heterozygous mutations of *TTN* are known to cause dilated cardiomyopathy (OMIM #604145). The mutation c.35635G>C; p.V11879L detected in your patient (W1-1) and his father (W1-3), has been described to cause dilated cardiomyopathy, albeit in compound heterozygosity in a severe case (Herman *et. al.*, N Eng J Med 2012). A paternal uncle and the paternal grandfather of the index patient (W1-1) are known to have atrial fibrillation. We suggest the testing of these individuals as well as other family members for the mutation c.35635G>C; p.V11879L and for signs of dilated cardiomyopathy.

The mother (W1-2) has heart arrhythmia, which might be due to the mutation c.39893-1G>A; p.?. We suggest an echocardiography examination of the heart for dilated cardiomyopathy.

Results with respect to sensorineural hearing loss: In your patient (W1-1) we detected the

compound heterozygous mutations c.101T>C; p.M34T and c.35delG; p.G12Vfs*2 in exon 1 of the *GJB2*-gene (CX26; GenBank NM_004004.5, NC_000013.10). In the patient's mother W1-2 we detected the mutation c.101T>C; p.M34T in exon 1 of the *GJB2*-gene, the patient's father W1-3 carries the mutation c.35delG; p.G12Vfs*2 in exon 1 of the *GJB2*-gene. These findings have been confirmed with the whole genome sequencing data of the family members (W1-1, W1-2 and W1-3). As there is no DNA available to us, the mutations have not been validated by Sanger Sequencing. We strongly recommend to validate these results also by conventional methods.

Interpretation of *GJB2* gene mutations: We are able to **support the clinical diagnosis of sensorineural hearing loss** due to mutations in the *GJB2* gene in your patient (W1-1). The mutations **c.101T>C; p.M34T and c.35delG; p.G12Vfs*2 in exon 1 of the *GJB2*-gene** are both pathogenic mutations that have been described in multiple publications. Patients that are compound heterozygous for c.101T>C; p.M34T and c.35delG; p.G12Vfs*2 in exon 1 of the *GJB2*-gene have mild to moderate hearing loss (Snoeckx *et. al*., Am J Hum Genet 2005). The mutation c.101T>C; p.M34T is a common pathogenic variant and listed in dbSNP (rs35887622) with a global minor allele frequency of 1%. It leads to the exchange of a highly conserved amino acid in the GJB2 protein. The mutation c.35delG; p.G12Vfs*2 is a 1 bp deletion that leads to a frameshift and a premature stop codon. It is also listed in dbSNP (rs80338939), but no minor allele frequency is given.

The mother (W1-) is a heterozygous carrier of the mutation c.101T>C; p.M34T. The father (W1-3) is a heterozygous carrier of the mutation c.35delG; p.G12Vfs*2. As both mutations have been reported with autosomal-recessive inheritance, neither the mother nor the father are expected to have hearing impairment due to these mutations.

Mutations in intronic, promoter and enhancer regions as well as deletions and duplications have not been investigated by our methods and can therefore not be excluded. Furthermore, we cannot rule out that additional mutations could have been found by conventional sequencing methods. Massively next generation high throughput sequencing is a very new and cost efficient screening method to test for known mutations in disease associated genes in parallel.

These results should be communicated by a human geneticist or by a genetic counselor. If you have any further questions please do not hesitate to contact us.

With kind regards,

Saskia Biskup, MD PhD — Consultant for Human Genetics

Prof. Peter Freisinger, MD — Pediatrician

Scientific use of these results requires permission by the investigators. The Center for Genomics and Transcriptomics Tübingen follows the quality guidelines for molecular genetic testing set up by the European Molecular Genetics Quality Network (EMQN).
