# Supplementary material for: An international effort towards developing standards for best practices in analysis, interpretation and reporting of clinical genome sequencing results in the CLARITY Challenge
Source: Genome Biol. 2014 Mar 25;15(3):R53. doi: 10.1186/gb-2014-15-3-r53 (PMC4073084; doi:10.1186/gb-2014-15-3-r53)
Supplement: Additional file 2 — The entry from the Genomatix/CeGaT/University Hospital of Bonn team containing five PDF files and six XLS tables. [file gb-2014-15-3-r53-S2.zip › Additional_file_2/Medical_report_W2.pdf]

CeGaT & Praxis für Humangenetik, Paul-Ehrlich-Str. 17, 72076 Tübingen

The CLARITY Team

Center for Genomics
and Transcriptomics

CeGaT GmbH &
Praxis für Humangenetik
Paul-Ehrlich-Str. 17
D-72076 Tübingen

www.cegat.de

Tel: +49 7071 5654400
saskia.biskup@cegat.de

09/25/2012

Results of Next Generation Sequencing (NGS) diagnostics - CLARITY Challenge

Primary Diagnosis: Cardiac Defects

**Patient**	**dob.**	**Pat. No.**	**Material**	***TRPM4***
Family2, Affected Child 2-1	undisclosed	W2-1	sequencing data	**c.503T>A; p.V168E (het.)**
Family2, Affected Mother 2-2	undisclosed	W2-2	sequencing data	**c.503T>A; p.V168E (het.)**
Family2, Father 2-3	undisclosed	W2-3	sequencing data	none
Family2, Affected Cousin 2-4	undisclosed	W2-4	sequencing data	**c.503T>A; p.V168E (het.)**
Family2, Aunt 2-5	undisclosed	W2-5	sequencing data	none
Family2, Affected Uncle 2-6	undisclosed	W2-6	sequencing data	**c.503T>A; p.V168E (het.)**

Dear CLARITY Team,

Thank you very much giving us the opportunity to analyze the exome and genome sequencing data of the family mentioned above with respect to the primary diagnosis of cardiac defects. We received the data on 06/06/2012 and analyzed the exome sequencing data for mutations shared by all patients (W2-1, W2-2, W2-4 and W2-6) that were not present in the unaffected family members (W2-3 and W2-5).

Results: All four patients (W2-1, W2-2, W2-4 and W2-6) share the **heterozygous mutation c.503T>A; p.V168E in exon 5 of the *TRPM4* gene** (Genbank: NM_017636; NC_000019.9). This mutation was not found in the datasets of the two unaffected family members (W2-3 and W2-5). These results have been confirmed with the genomic sequencing data of family members No. W2-1, W2-2, W2-3 and W2-6. As there is no DNA available to us, the mutations have not been validated by Sanger-Sequencing. Validation by Sanger-Sequencing is strongly recommended.

Interpretation: The cardiac defects observed in members of family W2 are probably due to the heterozygous mutation c.503T>A; p.V168E in exon 5 of the *TRPM4* gene. To our knowledge, this mutation has not yet been described in the literature. It changes a conserved amino acid in the cytoplasmic domain of TRPM4 protein. The prediction program "MutationTaster" predicts the mutation to be disease causing. Liu et. al. describe a mutation p.R164E, which is very close to the mutation found in family W2, as causing Autosomal Dominant Isolated Cardiac Conduction Disease (Liu et. al.; Circ Cardiovasc Genet 2010).

Heterozygous mutations in the *TRPM4* gene have been reported to cause progressive familial heart block, type IB (OMIM #604559). A high phenotypic variability has been described within affected families, mainly right bundle branch block (RBBB) and atrioventricular (AV) block, which has also been observed in family W2. Patients with pathogenic *TRPM4* mutations are at risk of sudden cardiac death.

All affected family members show cardiac arrhythmia (RBBB and AVB) which can be explained by the described mutation. Patient 2-4 and 2-6 also present severe structural abnormalities (2-4 pulmonary stenosis and hypoplastic right heart, 2-6 pulmonary stenosis and coarctation). Up to now no structural abnormalities are described in mutations of *TRMP4*. It remains unclear whether the structural cardiac abnormalities in 2-4 and 2-6 are due to an independent genetic defect or due to effects of the mutation c.503T>A; p.V168E in *TRPM4* during embryonic cardiac development

Mutations in intronic, promoter and enhancer regions as well as deletions and duplications have not been investigated by our methods and can therefore not be excluded. Furthermore we cannot rule out that additional mutations could have been found by conventional sequencing methods. Next generation high throughput sequencing is a new and cost efficient screening method to test for known mutations in many disease associated genes in parallel.

These results should be communicated by a human geneticist or by a genetic counselor. If you have any further questions please do not hesitate to contact us.

With kind regards,

Saskia Biskup, MD PhD — Prof. Peter Freisinger, MD

Consultant for Human Genetics — Pediatrician

Scientific use of these results requires permission by the investigators. The Center for Genomics and Transcriptomics Tübingen follows the quality guidelines for molecular genetic testing set up by the European Molecular Genetics Quality Network (EMQN).
