# Supplementary material for: An international effort towards developing standards for best practices in analysis, interpretation and reporting of clinical genome sequencing results in the CLARITY Challenge
Source: Genome Biol. 2014 Mar 25;15(3):R53. doi: 10.1186/gb-2014-15-3-r53 (PMC4073084; doi:10.1186/gb-2014-15-3-r53)
Supplement: Additional file 2 — The entry from the Genomatix/CeGaT/University Hospital of Bonn team containing five PDF files and six XLS tables. [file gb-2014-15-3-r53-S2.zip › Additional_file_2/Medical_report_W3.pdf]

CeGaT & Praxis für Humangenetik, Paul-Ehrlich-Str. 17, 72076 Tübingen

The CLARITY Team

Center for Genomics
and Transcriptomics

CeGaT GmbH &
Praxis für Humangenetik
Paul-Ehrlich-Str. 17
D-72076 Tübingen

www.cegat.de

Tel: +49 7071 5654400
saskia.biskup@cegat.de

09/25/2012

Results of Next Generation Sequencing diagnostics (NGS) - CLARITY Challenge

Primary Diagnosis: Nemaline Myopathy

Patient	dob.	Pat. No.	Material	Genetic variants
Family3, Affected 3-1	n.a.	W3-1	sequencing data	***TTN*: c.[84130A>T]+[=]; p.[K28044X]+[=]**<br><br>***OBSCN*: c.[2245G>T]+[3322T>A]; p.[G749C]+[Y1108N]**
Family3, Mother 3-2	n.a.	W3-2	sequencing data	***OBSCN*: c.[3322T>A]+[=]; p.[Y1108N]+[=]**
Family3, Father 3-3	n.a.	W3-3	sequencing data	***TTN*: c.[84130A>T]+[=]; p.[K28044X]+[=]**<br><br>***OBSCN*: c.[2245G>T]+[=]; p.[G749C]+[=]**

Dear CLARITY Team,

Thank you very much for giving us the opportunity to analyze the exome and genome sequencing data of the family mentioned above with respect to the primary diagnosis of nemaline myopathy. We received the data on 06/06/2012 and searched for de novo and X-chromosomal mutations in the index patient (W3-1) as well as for genes containing a homozygous or compound heterozygous mutation in the patient (W3-1), but not in the unaffected parents (W3-2 or W3-3).

Results: In your patient (W3-1) we detected the heterozygous mutation **c.84130; p.K28044X in exon 283 of the *TTN* gene** (Genbank: NM_001256850.1; NC_000002.11), and the heterozygous variants **c.2245G>T; p.G749C and c.3322T>A; p.Y1108N in exons 7 and 11 of the *OBSCN* gene** (Genbank: NM_001098623.1, NM_052843.2; NC_000001.10).

In the patient's mother (W3-2) we detected the variant **c.3322T>A; p.Y1108N in exon 11 of the *OBSCN* gene**, the patient's father (W3-3) carries the mutation **c.84130; p.K28044X in exon 283 of the *TTN* gene and the variant c.2245G>T; p.G749C in exon 7 of the *OBSCN* gene**. These results

have been confirmed with the genomic sequencing data of all three family members (W3-1, W3-2 and W3-3). As there is no DNA available to us, the mutations have not been validated by Sanger Sequencing. We strongly recommend to confirm these variants by conventional methods.

Interpretation: We suspect that the nemaline myopathy in your patient (W3-1) may be due to mutations within the *TTN* and/or *OBSCN* genes. We identified the heterozygous mutation **c.84130; p.K28044X in exon 283 of the *TTN* gene**, which has, to the best of our knowledge, not been described in the literature**.** The mutation c.84130; p.K28044X in exon 283 of the *TTN* gene is very likely to be pathogenic as it leads to a premature stop codon. This amino acid exchange severely truncates the TTN protein and leads to a loss of the binding site of the OBSCN protein (Fukuzawa *et. al.,* J Cell Science 2008).

We also detected the heterozygous variants **c.2245G>T; p.G749C and c.3322T>A; p.Y1108N in exons 7 and 11 of the *OBSCN* gene**. The variant c.3322T>A; p.Y1108N in exon 11 of the *OBSCN* gene is listed in the dbSNP database (rs199696332), but has been found only in a single study with an allele frequency of 0.4% and has not been found within the 1000 genomes project. It exchanges an amino acid in the Ig-like domain 11 of the OBSCN protein. “MutationTaster” predicts this variant to be a polymorphism, albeit only with a probability of 71%. The variant c.2245G>T; p.G749C has not been described in the literature and has not been observed in the 1000 genomes project or the NHLBI Exome Sequencing Project (ESP). It exchanges an amino acid in the Ig-like domain 7 of the OBSCN protein. “MutationTaster” predicts this variant to be a polymorphism with a high probability. The amino acid at this position is phylogenetically not conserved.

Mutations of a single *TTN* allele may cause dilated cardiomyopathy or tibial muscular dystrophy (Carmignac *et. al.*, Ann Neurol 2007; Hackman *et. al.* Neuromuscul Disord 2008 and others). The mutation c.84130; p.K28044X detected in your patient (W3-1) and his father (W3-3) may cause dilated cardiomyopathy or tibial muscular dystrophy. Other mutations introducing premature stop codons in the *TTN* gene have been described to cause dilated cardiomyopathy or tibial muscular dystrophy (Carmignac *et. al.*, Ann Neurol 2007; Hackman *et. al.* Neuromuscul Disord 2008). As the father (W3-3) has a heart murmur, we suggest he should be monitored for signs of dilated cardiomyopathy. The index patient (W3-1) should be monitored for dilated cardiomyopathy as well, although the age of onset of dilated cardiomyopathy is usually in adulthood.

Pathogenic mutations in the *OBSCN* gene have up until now not been described in literature at all. However, Fukuzawa *et. al.* showed that several pathogenic *TTN* mutations cause a disruption of OBSCN protein localization, suggesting that OBSCN may play a role in neuromuscular disorders (Fukuzawa *et. al.,* J Cell Science 2008). Animal models are not fully supporting this suggestion as an *Obscn* knock-out mouse has only very mild signs of myopathy (Lange *et. al.*, J Cell Science 2009). A zebrafish model, however, that lacks the OBSCN RhoGEF domain develops severe neural and muscular abnormalities (Raeker et. al., Dev Biol 2010).

We think that the combination of the *TTN* mutation and one or both *OBSCN* variants in the index patient may be responsible for the clinical diagnosis of nemaline myopathy. Due to the lack of information on the pathogenicity of *OBSCN* mutations this is a speculative result that requires support

from further in vitro and in vivo studies (e.g. immunohistochemistry on muscle biopsies of your patient).

Mutations in intronic, promoter and enhancer regions as well as larger deletions and duplications have not been investigated by our methods and can therefore not be excluded. Furthermore, we cannot rule out that additional mutations could have been found by conventional sequencing methods or sequencing with higher coverage. Our quality criteria demand a coverage of at least 10 reads per base pair, which has not been reached in all relevant regions of potentially causative genes by the provided data. Massively next generation high throughput sequencing is a very new and cost efficient screening method to test for known mutations in disease associated genes in parallel.

These results should be communicated by a human geneticist or by a genetic counselor. If you have any further questions please do not hesitate to contact us.

With kind regards,

Saskia Biskup, MD PhD   Prof. Peter Freisinger, MD

Consultant for Human Genetics   Pediatrician

Scientific use of these results requires permission by the investigators. The Center for Genomics and Transcriptomics Tübingen follows the quality guidelines for molecular genetic testing set up by the European Molecular Genetics Quality Network (EMQN).
