# Supplementary material for: An international effort towards developing standards for best practices in analysis, interpretation and reporting of clinical genome sequencing results in the CLARITY Challenge
Source: Genome Biol. 2014 Mar 25;15(3):R53. doi: 10.1186/gb-2014-15-3-r53 (PMC4073084; doi:10.1186/gb-2014-15-3-r53)
Supplement: Additional file 3 — The entry from the University of Iowa. [file gb-2014-15-3-r53-S3.pdf]

UNIVERSITY of IOWA
HEALTH CARE

University of Iowa Hospitals and Clinics

Department of Otolaryngology – Head & Neck Surgery
200 Hawkins Drive, 21151-PFP
Iowa City, IA 52242-1078
319-356-3612 ***Tel***
319-356-4108 ***Fax***
www.uihealthcare.com

September 24, 2012

Katherine Flannery
Program Manager
Harvard Medical School
Center for Biomedical Informatics
Email: katherine_flannery@hms.harvard.edu

RE: CLARITY Challenge

Dear Katherine,

Accompanying this letter is our CLARITY Challenge reports. As stipulated in the protocol and instructions, our reports are not focused on identifying unrelated findings associated with other health issues or diseases. Pursuant to these specific aims and objectives, we have completed the following:

1. Analysis of whole genome and whole exome sequence data on three families;
2. Identification of potential alterations associated with the phenotype in the proband;
3. Determination and identification of key components for reporting these results;
4. Correlation of the phenotype in the patient with the variant detected.

Note that based on the **Challenge Parameters** (IV, page 29), we have limited our report and taken a narrow view of the Data/Data Sets, focusing solely on that which is required for completion of the challenge. Specifically, **only variants/findings with a reasonable association with the patient phenotype have been reported.** Incidental findings are NOT reported.

Speaking on behalf of the participants at the University of Iowa, thank you for the opportunity to participate in the CLARITY Challenge. It has been an outstanding opportunity.

With kind regards,

Richard JH Smith, MD
Director, Iowa Institute of Human Genetics

(On behalf of the Institutional Team Members: Jean Andorf, Hela Azaiez, Thomas Bair, Ann Black, Kevin Booth, Aaron Bossler, Terry Braun, Colleen Campbell, Benjamin Darbro, Jonathan Heusel, Jian Huang, Kim Keppler-Noreuil, Anne Kwitek, Nicole Meyer, Jeffrey Murray, Karin Panzer, Kelli Ryckman, Todd Scheetz, Oleg Shchelochkov, Eliot Shearer, Val Sheffield)

Included Materials

1) Introduction	Page 1
2) Guiding Philosophy	Page 3
3) CLARITY Challenge Reporting Parameters	Page 7
4) Certificate of Destruction	Page 8
5) University of Iowa Clinical Exome Requisition Form	Page 9
6) University of Iowa Informed Consent	Page 13
7) University of Iowa Genetics Primer	Page 25
8) Clarity Challenge Family 1	Page 27
9) Clarity Challenge Family 2	Page 47
10) Clarity Challenge Family 3	Page 65

Introduction

Keeping in mind the Clarity Challenge goal of defining norms, standards and models for reporting exome and genome findings, as well as the ACMG Policy Statement on genomic sequencing (ACMG 2012) we created the documents listed below. The results and interpretation reflect the American culture and healthcare system and values.

Requisition Form

The information will be entered electronically and will populate the corresponding healthcare provider report fields.

Consent Form

The information will be entered electronically and will populate the corresponding healthcare provider report fields. Any information a patient does **not** wish to learn will be masked during the bioinformatics analysis so that the information does not enter the healthcare provider report.

a. Ordering

The test can only be ordered online through the Iowa Institute of Human Genetics (IIHG) Clinical Diagnostic Service Laboratory website. **The test cannot be ordered without confirmation that the client has received pre-test genetic counseling and arrangements have been made for post-test genetic counseling.** We suggest for the test request to be processed by the laboratory, a genetic counselor must login to the website and verify the patient has received pre-test genetic counseling. We suggest a collaboration with the American Board of Genetic Counselors to develop and provide board certified genetic counselors a national personal identification number (ABGC PIN), which can serve as an electronic signature. If a genetic counselor is not available the IIHG Clinical Diagnostic Service Laboratory will provide one.

b. Research Arm

The research arm of the testing involves the development of a phenotype-genotype central repository. We suggest that all patients who have this test be **offered** the opportunity to have their genetic sequence data stored in a database that also will include detailed clinical phenotypes, which are ascertained from the patient's medical record. Qualified researchers who obtain permission may gain access to **de-identified** data in the repository. We agree with previous reports (Kohane 2012; Aronson 2012) such a database on a local or national level is crucial to determine population allele frequencies, determine disease penetrance within a given population, identify novel disease-gene relationships, and understand how genetic variants contribute to health and disease.

c. Known Disease-Causing/Associated Variants and Genes

We define known disease-causing/associated variants and genes as those that **have been previously reported with a high degree of consensus** as to their clinical relevance. Known variants will be reported for both primary and secondary findings **if** the patient selects to learn of these findings.

d. Novel Variants and Novel Genes

Novel variants can be reported in **known** genes as long as the variant meets the appropriate quality, frequency and pathogenicity criteria. **Novel genes are genes that have not been shown to be causative or modifying of an existing condition, disease or syndrome.** Variants in **novel genes** are considered **research findings**. All research findings require in-depth studies to determine if the gene is causally related to the disease in question. Therefore these variants **do not fulfill our reporting criteria of being medically actionable** (Fabsitz 2010).

e. Longevity Reporting

If a patient elects to participate in longevity reporting, they may be informed of such research findings once these findings have been scientifically validated.

f. Medically Actionable Results

Medically actionable results are those for which there is an established therapeutic or preventative intervention or other action.

g. False-Positive Incidental Findings

With consideration of false-positive incidental findings, we believe there are four sources of false-positive results in whole genome and exome sequencing data: erroneous annotations, sequencing error, incorrect penetrance estimates, and multiple hypothesis testing (Kohane 2012). False-positive findings are of concern because these results will burden patients, healthcare providers, and insurers. We want to reduce the number of false-positive findings so that genetic sequencing data is a useful tool for the patient and healthcare provider in making informed medical care decisions. To limit the number of false-positive results, we suggest all variants should be annotated for allele frequency and functional impact estimates. A patient's genetic background, ethnicity and race should be considered when applying allele frequency filters as genetic background may impact disease penetrance. If genetic background is not considered the number of false-positive results will increase.

Guiding Philosophy

All of the documents were created with the following principles in mind (Zawati et al 2012).

- Do no harm
- Findings reported to the patient should be:
  - Analytically valid (known disease causing genes)
  - Medically actionable
  - Clinically significant
  - Reveal established and substantial risk of serious health conditions or of reproductive importance
- Respect patient autonomy and allow patient to decide which results they would like to receive through the consent process
- Consistent with College of American Pathologists (CAP) guidelines
- Compliant with applicable law
- Findings will not replace medical examinations or clinical expertise necessary for accurate diagnosis and medical management.

Genetics Primer

This basic brochure can be provided by any healthcare provider to their patient after discussing the possibility of genetic sequencing tests. This primer is intended only to provide basic information about the test **prior to meeting** a genetic counselor.

At a pre-test genetic counseling appointment the genetic counselor will

1. Go into greater detail about the testing including outcomes of testing, benefits, limitations, what will or will not be disclosed, and incidental findings.
2. Answer any questions the patient may have regarding the test
3. Once a patient decides if they would like to have testing or not have testing coordinate any follow up.

Requisition Form

A basic intake form will be completed online by the healthcare provider and/or genetic counselor. The lab will **not** process the test until a complete requisition form is submitted. This form is to be used to assist the lab in filtering genetic variants to identify the causative variant in the patient.

Informed Consent

We created an informed consent document, which is an all-encompassing document for all ages, various forms of genetic testing (targeted sequencing, whole exome, and whole genome), clinical diagnostic testing and research participation. We wanted to create a

document that would be the least overwhelming for healthcare providers, genetic counselors, and patients.

Our consent form allows the individual personalize their test results by selecting which classes of information they would like to learn as to protect their autonomy. In addition, there are places where the patient can write in specific conditions or genes for which they wish to learn or not learn their findings.

We have broadly defined conditions to include: primary condition (reason for testing), and secondary findings (incidental findings). Any patient consenting to have whole exome and/or whole genome sequencing **will** learn of variants known to cause their primary condition including novel variants in genes previously associated with the patient's [differential] diagnosis. **In addition, variants which may affect patient response to medication, coagulation, or other treatment such as radiation therapy will always be included in the test report. Only known, medically actionable variants will be included in the report.**

Patients will have the choice to enroll in longevity reporting for their primary condition and receive updates on genetic variants pertaining to their primary condition for up to 7 years (see Longevity Reporting Below).

<u>Secondary findings</u> are broadly divided by age of onset into pediatric-onset conditions and adult-onset conditions. **To limit potential stress from a false-positive incidental findings, only known variants in known disease causing genes will be reported for secondary findings.** The report of secondary findings will be provided to the healthcare provider as a report addendum which would not be included in the patient's electronic medical record but can be accessed by the healthcare provider through the Iowa Institute of Human Genetics (IIHG) Clinical Diagnostic Service Laboratory website. All reports and addendums are dated to reflect knowledge and interpretation at the time of analysis.

Pediatric and adult-onset conditions are further divided into treatable and untreatable. As these categories only apply to **<u>secondary findings</u>**, we do not report pediatric onset, untreatable disorders. Parents can select to learn if their child is at risk for pediatric onset, treatable disorders. Individuals over the age of 18 can select to learn secondary findings for adult onset treatable conditions, and adult-onset untreatable conditions. In addition, adults may learn if they are carriers for autosomal recessive and X-linked recessive conditions (Bell et al 2011), or other phamacogenetic variants.

All individuals will be able to select if they would like to receive longevity reporting for secondary findings. As the University of Iowa is located in a small academic community, we must consider some highly educated individuals may wish to receive their raw genomic data. Alternatively, if no disease causing variants are identified and the patient wishes to provide their results to an investigator who specializes in their disease, patients may select this option so they can provide the investigator with a copy of their variant call file. We realize this will not be a frequent request but we decided to proactively explain that if an individual wishes to receive their raw data for their own

personal bioinformatics analysis they will be required to have an additional genetic counseling appointment to discuss in more detail what they may learn from the raw data.

Longevity Reporting

As part of the clinical interpretation of exome data, a system is required to update the analysis process/pipeline for both new patients and for patients whose exomes were previously sequenced but a causal mutation was not identified. Next-generation sequence technologies have made it possible to use exome sequencing for identifying rare variants underlying disease. Currently this approach is most applicable to Mendelian diseases, but wide-spread clinical diagnostic applications are imminent. As clinical and population exome sequencing expands, it is important to use the most recent data available for clinical interpretation. Several public databases are available that are regularly updated. This supply of new genomic data and improvements to the analysis tools will require the participation of the informatics group, the clinical interpretation group and the genetic counseling/reporting group to regularly revise the variant lists and analysis process.

Bioinformatic Resources

- A comprehensive database that can store local sequence data and phenotypic data
- An automated or semi-automated query to public sequence databases outlined in workflow
    - Comprehensive population sequence data
        - 1000 Genomes
        - NHLBI Exome Projects Exome variant server (University of Washington)
    - Cancer GenomeVariant associations and disease-causing variants from public sources
        - HGMD
        - OMIM morbid map
        - GAD
        - GWAS catalog
        - PharmGKB
        - dBSNP
        - Disease focused databases, e.g. http://deafnessvariationdatabase.com/, Leiden Open Variation Database (http://www.lovd.nl/2.0/), T1dbase(http://www.t1dbase.org/), PKD mutation database (http://pkdb.mayo.edu)
- Ability to perform annual queries and flag new variants, update population allele frequencies and putative variant function that may have clinical relevance

Interpretation

- A workflow to re-interpret previously discovered variants and their putative clinical importance in our local datasets.
- Assess variants across all patient data rather than per-patient
- Any ‘clinically relevant’ variants annotated in our full local data set

Reporting

- A patient may choose to have longevity reporting for variants related to the primary indication.
- If they have opted in, their data will be reanalyzed on an annual basis for 7 years to determine whether any variants identified from initial test results have undergone a change in reporting status with respect to primary condition.
- To ensure we are analyzing only the highest quality data, after 7 years analyses may require repeat sequencing.
- Variants will be updated by processing through the current validated workflow.
- Patients opting to receive updated information would be flagged if a causal mutation has been identified and an updated report sent to the patient's physician.
- Upon visiting primary physician during annual examination, the patient will be given the option of learning about the change in variant status for this test.
- Allow for an 'update' flag on any patient web interface so that they might contact their primary care physician or genetic counselor for further counseling and disease management if the status has changed on primary finding.

References utilized to generate our guiding philosophy

Aronson SJ, Clark EH, Varugheese M, Baxter S, Babb LJ, Rehm HL. Communicating new knowledge on previously reported genetic variants. Genet Med. 2012 Apr 5.

Bell CJ, et al. (2011) Carrier Testing for Severe Childhood Recessive Diseases by Next-Generation Sequencing. Sci Transl Med. 3(65):65ra4.

Bush LW, Rothenberg KH. Dialogues, dilemmas, and disclosures: genomic research and incidental findings. Genet Med. 2012 Mar;14(3):293-5.

Kohane IS, Hsing M, Kong SW. Taxonomizing, sizing, and overcoming the incidentalome. Genet Med. 2012 Apr;14(4):399-404.

National Heart, Lung, and Blood Institute working group, Fabsitz RR, McGuire A, Sharp RR, Puggal M, Beskow LM, Biesecker LG, Bookman E, Burke W, Burchard EG, Church G, Clayton EW, Eckfeldt JH, Fernandez CV, Fisher R, Fullerton SM, Gabriel S, Gachupin F, James C, Jarvik GP, Kittles R, Leib JR, O'Donnell C, O'Rourke PP, Rodriguez LL, Schully SD, Shuldiner AR, Sze RK, Thakuria JV, Wolf SM, Burke GL. Ethical and practical guidelines for reporting genetic research results to study participants: updated guidelines from a National Heart, Lung, and Blood Institute working group. Circ Cardiovasc Genet. 2010 Dec;3(6):574-80.

Points to consider in the clinical application of genomic sequencing. Genet Med. 2012 Aug;14(8):759-61.

Zawati MH, Knoppers BM. International normative perspectives on the return of individual research results and incidental findings in genomic biobanks. Genet Med. 2012 Apr;14(4):484-9.

<u>CLARITY CHALLENGE REPORTING</u>

For the purpose of the Clarity Challenge, variants which may affect patient response to medication, coagulation, or other treatment such as radiation therapy are NOT included in the test report in response to the Challenge Parameters (IV, page 29): only variants/findings with a reasonable association with the <u>patient phenotype</u> have been reported.

For the purpose of the Clarity Challenge, we DID include an option for the patients to learn of <u>research</u> results for the primary phenotype. These results include variants in genes that may or may NOT be involved with the primary condition, and are considered strictly research findings. These results should not be used for making decisions regarding medical care, but may be used to find potential investigators who may be interested in studying these results further.

CERTIFICATE OF DESTRUCTION

University of Iowa Hospital and Clinics

The information described below was destroyed in the normal course of business pursuant to a proper retention schedule and destruction policies and procedures.

Date of destruction: 09/26/2012

Description of records or record series disposed of: Hard and digital copies of all Data and datasets as well as patients records and information owned by Boston Childeren's Hospitals set out in Participation agreement to the CLARITY Challenge.

Inclusive dates covered: ______________________________________

Method of destruction:

( ) Burning (X) Shredding ( ) Pulping
( ) Demagnetizing (X) 35-pass secure erase ( ) Pulverizing
( ) Other:____

Records destroyed by: ______________________________________

Witness signature: ____

Department manager: ____

University of Iowa Clinical Exome Requisition Form

Clinical Requirements:
Due to the complexity of exome sequencing, it is essential that we are informed of the complete clinical picture for each patient.

The following items are required before testing is initiated:

Patient clinical history and clinic notes	
Results of prior testing	
Family history (in the form of a pedigree)	
Identification of family members available for testing. Please identify these individuals on the pedigree and indicate if symptomatic.	

*A pedigree tool is available at:
http://www.medicine.uiowa.edu/uploadedFiles/Research/Human_Genetics/Pages/Clinical_Genetics/IIHG%20How%20to%20Draw%20a%20Pedigree.pdf

Differential Diagnosis
Please provide your differential diagnoses for the patient.

Candidate Genes
If you have identified any gene candidates or genetic pathways of interest, please list them below.

Phenotypic Checklist
Please complete the boxes below for your patient. For any box checked yes, please provide additional information below or under separate cover.

	Yes	No	Not assessed
Intellectual Disability			
Speech/Language Delay			
Gross/Fine Motor Delay			
Autism/Autism Spectrum Disorder			
ADHD/ADD			
Other psychiatric diagnoses			
Hypotonia			
Hypertonia			
Ataxia/Tremors			
Seizures			
Vision Loss			
Hearing Loss			
Microcephaly			
Macrocephaly			
Short Stature			
Tall Stature			
Poor weight gain			
Obesity			
Dysmorphic features			
Heart Defect			
Kidney abnormality			
Genital abnormality			
Skeletal abnormality			
Skin pigmentary changes			

Additional Phenotypic Information

Ethnicity and Race of Patient:

Pertinent Laboratory/Imaging Studies

For any box checked yes, please provide findings below or attach the report. For genetic testing, please include date completed and laboratory name.

	Yes	No	Not Done
MRI/CT brain			
MRI/CT other body part			
EEG			
Echocardiogram			
ECG			
Abdominal/Retroperitoneal ultrasound			
Skeletal survey			
Other plain films			
Karyotype			
Chromosome microarray			
FISH studies			
Methylation studies			
Single gene sequencing and/or del/dup			
Gene panels			

Additional laboratory/imaging Information

INFORMED CONSENT DOCUMENT

Project Title: Clinical Exome and Whole Genome Sequencing

Principal Investigator: Richard Smith, MD

Research Team Contact: Aaron Bossler, MD, PhD; Colleen Campbell, PhD, MS, CGC; Jonathan W. Heusel, MD, PhD; Anne E. Kwitek PhD; Tara Maga, PhD; Karin Panzer, MS, CGC; Tom Wassink, MD.

This consent form describes the clinical DNA sequencing test to help you decide if you would like this test. This form provides important information about what you will be asked to do for the test, the risks and benefits of the test, and your rights as a patient. This document also describes how the results from this test might be used in research, and how you may choose to participate.

If you have any questions about or do not understand something in this form, you should ask your healthcare provider for more information. You can discuss your participation with anyone you choose such as family or friends. Do <u>not</u> agree to this test unless your healthcare provider has answered your questions and you decide that you want to have this test.

- If you are the parent/guardian of a child under 18 years old who is being invited to be in this study, the word "you" in this document refers to your child. You will be asked to read and sign this document to give permission for your child to participate.
- If you are under 18 years of age, the word "you" in this document refers to you. You will be asked to read and sign this document to indicate your willingness to participate.
- If you are the legally authorized representative of a person who is being invited to be in this study, the word "you" in this document refers to the person you represent. You will be asked to read and sign this document to give permission for the person you represent to participate.

<u>CLINICAL DNA SEQUENCING</u>

A. WHAT IS THE PURPOSE OF THIS TEST?

1. <u>To Provide Information to Assist with Your Clinical Care</u>

The purpose of this test is to identify one or more genetic variants that may underlie the medical condition for which you were referred. The identification of such variants <u>may or may not</u> impact your medical care in the following ways:

- Identify a cause for your health condition or symptoms

- Assist you and your healthcare provider in choosing the best treatment for you
- Determine recurrence risks of disease in your family
- Identify predisposition to disease

2. <u>To Provide Information for a Genetic Research Study</u>

By undergoing this test, you are also invited to participate in a research study investigating the relationship between genetic variation and human diseases. You may choose to opt out of the research portion of the study and just receive clinical information. No extra time or procedures are required for participation in the research study. Identifying information about you, such as your name and birth date will not be included in the research study, but we will need your permission to use information about your medical history and your test results. The research portion of this testing is described beginning on page 8.

B. WHAT WILL HAPPEN DURING THIS TEST?

1. <u>Genetic Counseling</u>

A genetic counselor is a healthcare professional trained to explain genetic information to you in a way that is easy to understand. You will meet with a genetic counselor throughout the testing process. Your time with the counselor will include:

- A pre-test genetic counseling appointment. At this appointment, the counselor will discuss how the test is performed, what the test results can and cannot tell you, limitations and risks of the test, and any benefits of the test. The genetic counselor will answer any questions you have about the test. The counselor will also gather information about your personal and family health history.
- You are then free to take time to think and speak with family members and friends before deciding if you would like to have the test. If you decide to have the test, the genetic counselor will complete the rest of this consent form with you and a sample of blood will be drawn as described below. Completion of consent and the blood draw can occur either at the pre-test visit or, if you prefer, at a later appointment that you would schedule.
- When the test results are available, you will meet with the genetic counselor to review the results.
- If at a later date you would like the results of this test to be re-analyzed for a new primary health condition a brief visit with a genetic counselor will be needed to discuss the test results for that condition.

2. <u>Cost of the Test</u>

During your genetic counseling appointment the cost of the test will be outlined for you and insurance coverage will be discussed. If you decide you do not want the test before processing of your sample begins, payment will be refunded in full. Participation in the research study will not result in any additional costs.

3. Obtaining DNA

We will usually obtain DNA from a sample of blood. We will draw two tablespoons of blood (~1 teaspoon for infants) usually from a vein in your arm using a needle. This will take about 15 minutes of your time. From this blood, we will extract DNA which will be used to perform the genetic test. You may be asked to give an additional sample of blood if the initial sample does not provide enough DNA for the test.

Sometimes we may choose to obtain DNA from tissues other than blood, such as saliva, skin, or a surgery tissue sample. For saliva, we would obtain one teaspoon by having you spit into a provided collection kit. Collecting from skin or surgery tissues will require additional conversations.

4. Consulting Your Medical Records

To interpret the results of this genetic test we will need to access your medical records, including information about your medical history and any medical tests or procedures you have had.

5. Sequencing Your DNA

This consent is for a genetic test that involves sequencing your DNA. Several types of sequencing tests are available. Your healthcare provider will decide which type of DNA sequencing is best for you, and may order additional DNA testing if necessary. The human genome includes all of the information in our DNA. The exome is the portion of the human genome that encodes proteins, and includes only 1-2% of the human genome. The types of sequencing tests covered by this consent include:

Targeted Capture Sequencing
A subset of genes which are known to cause disease can be tested, such as all genes known to cause hearing loss. The advantage of this type of testing is that all variants identified will be in known disease causing genes. The disadvantage is if the genetic variant causing your disease is in a gene not yet known to cause your disease, the variant will not be identified.

Exome sequencing (WES)
Exome sequencing is targeted capture sequencing of all exons. Exons are the parts of our genome that code for proteins. To date, the majority of disease-causing mutations are in the exons of genes. Exome sequencing thus screens the coding portion of almost every known gene, including the mitochondrial genome. The advantage of exome sequencing is that this test should detect DNA variants in the majority of the coding portions of the human genome. The disadvantages are: 1) the test does not yet cover the entire exome, so follow-up testing of regions which were not covered may be necessary, and 2) non-coding regions of the genome may have disease variants that exome sequencing will miss.

Whole genome sequencing (WGS)
Whole genome sequencing screens the entire human genome for genetic variants. The advantage of whole genome sequencing is it will detect DNA variants throughout the human genome, not just in exons, and all variants have the potential to be medically important. The disadvantages are: 1) the test does not yet cover the entire genome, so some regions may be missed, and 2) WGS will identify many variants, and we may not yet be able to determine which are medically important.

6. Analyzing and Reporting on Your Sequencing Data

Once we have your sequence data, we need to determine if it contains any genetic variants which may be causing your health condition. To do this, we will use computers to compare your sequence data with sequence data from people with medical conditions similar to yours and to people with no medical conditions. We will also assess whether variants could affect genes and proteins in ways that are medically important.

C. WHAT INFORMATION WILL I RECEIVE FROM THIS TEST?

1. Findings Related to Your Primary Condition

All individuals who are tested will receive a report describing genetic findings related to their primary condition. A genetic counselor and your physician will explain these results to you. The report will in all cases include current information related to your primary condition. You may also choose to enroll in Longevity Reporting related to your primary condition (see below). **Initial next to each category about which you wish to be notified:**

**Findings Related to Primary Condition**	**Notify? Yes**	**Notify? No**
• **Variants that are known to cause your primary condition.**<br>• **Novel variants that affect genes related to your primary condition.** This includes previously undiscovered variants that impair genes previously associated with your medical condition.<br>• **Variants that affect medications used to treat your primary condition.** Some genetic variants affect various aspects of medications, such as effectiveness, side effects, and dosing. We will report these variants if they are related to medications used to treat your primary condition. We will only report on variants for which there is an established therapeutic or preventative intervention or other action. We recommend you do not change your medications or supplements without speaking to your healthcare provider.		*(you must select Yes for these items)*
• **Longevity reporting related to your primary condition.** Our report will be based on currently available genetic data. We will also update our database as new genetic information		

becomes available. We may therefore learn that a variant you carry has become associated with your primary condition. We may also learn that a variant we identified as important to your condition has instead been shown to be benign. If you say "Yes" to Longevity Reporting, we will update your data on a yearly basis ***for the next seven years*** and notify you and your healthcare provider if such changes occur. ______ ______

2. Secondary Findings (Findings Unrelated to Your Primary Condition)

The testing will reveal genetic variants that are medically meaningful but unrelated to your primary condition. You may currently not have any symptoms of these diseases. Regardless of your genetic test results, your family history is the strongest indicator for your risk to develop disease. We will only report back known variants in known disease genes. These results will be returned to you and your healthcare provider as a report addendum available on the Iowa Institute of Human Genetics (IIHG) Clinical Diagnostic Service Laboratory website, and not placed in your medical record. You must choose whether or not to be notified of these findings. **Initial either Yes or No next to each category of Secondary Finding about which you wish to be notified:**

	Notify?	
**Secondary Finding Category**	**Yes**	**No**
• I **do not** wish to learn of any secondary results of my genetic test.	______	______
• **Pediatric Onset, Treatable.** These variants place your child at a higher risk for development of pediatric onset diseases that are treatable. Diseases in this category have medical management options available such as screening, treatment, or prevention.	______	______
• **Adult Onset, Treatable.** These variants place you at higher risk for development of adult onset diseases that are treatable. Diseases in this category have medical management options available such as screening, treatment, or prevention.	______	______
• **Adult Onset, Untreatable.** These variants place you at higher risk for development of adult onset diseases that are currently not treatable. ***We will not report these types of variants for any individual under the age of 18.***	______	______
• **Carrier Status.** A carrier is someone who carries a risk variant for an autosomal recessive disorder, or an X-linked recessive disorder. Carriers do not typically have symptoms of the disorder. For autosomal recessive disorders, if both parents carry risk variants in the same gene, the likelihood of a child developing the disorder is typically 25%. Most individuals carry 0-10 autosomal recessive risk variants.	______	______
• **Pharmacogenetic.** These variants may alter your drug response to medications other than those used for your primary condition.	______	______

- **Longevity reporting related to secondary findings.** If you say "Yes" to Longevity Reporting for secondary findings, we will update these findings with new genetic data on a yearly basis ***for the next seven years*** and notify you and your healthcare provider if such changes occur. ______ ______
- **Obtain Raw Data.** Most individuals will not wish to obtain all of their variants. To receive the raw data you MUST have an additional genetic counseling appointment to discuss what you may learn from these results. Initial if you would like to request a follow up genetic counseling appointment to discuss obtaining your raw sequence data. ______ ______
- **Research Results for the Primary Indication.** We may not be able to identify the cause for your disease. In this situation, we can try to identify other candidate variants that may or may not be involved with your disease. These variants are considered research findings are NOT to be used to make any decisions regarding your medical care as their role in human disease is UNKNOWN. ______ ______
- There are specific genes or diseases for which I DO NOT want to receive information (indicate them below or attached additional sheet). ______ ______

____

____

- Special Instructions: ____

____

D. WHAT INFORMATION WILL NOT BE PROVIDED BY THIS TEST?

- This test will not report on issues of paternity/non-paternity.
- For minors under-going this test, we will NOT report genetic variants that cause untreatable pediatric or adult diseases.
- Some areas of the human genome will not be fully sequenced through the technology used in this study.
- Because we do not yet fully understand the human genome, we will not be able to interpret your genetic data with complete accuracy.

E. WHAT ARE THE RISKS OF THIS TEST?

You may experience physical discomfort or bruising from the needle prick if blood is drawn from your arm. Potential risks from a skin biopsy include infection, bleeding, pain or scarring at the biopsy site.

Given the nature of this test we will find thousands of genetic variants. These variants will not all be related to your primary condition. We will not report such variants unless you chose to learn of those results.

E. ARE THERE OTHER TESTING OPTIONS?

Other testing options available to you depend on your medical condition and the specific clinical question. Before you decide whether or not to be in this study, your doctor and/or genetic counselor will discuss the other options and help you make an informed choice.

F. MEDICAL ADVICE

Our report will not provide medical advice. The results will include information your healthcare provider can use in combination with professional knowledge and clinical information to determine what might be causing your symptoms, and the best medical course of action. The results of this test should not be used in isolation to make a medical diagnosis or treatment decision. You should not ignore any symptoms you experience or discontinue treatment based on content of your report.

G. SIGNATURES

This Informed Consent Document is not a contract. It is a written explanation of what this testing involves if you decide to participate. You are not waiving any legal rights by signing this Informed Consent Document. Your signature indicates that this testing has been explained to you, that your questions have been answered, and that you agree to participate. You will receive a copy of this form.

Subject's Name (printed): ____

Signature: _______________________________________Date: ________________

Parent/Guardian or Legally Authorized Representative's Name and Relation to Subject:

Name: ___________________________Relationship to Subject:_________________

Signature: _______________________________________Date: ________________

Statement of Person Who Obtained Consent

I have discussed the above points with the subject or, where appropriate, with the subject's legally authorized representative. It is my opinion that the subject understands the risks, benefits, and procedures involved with participation in this testing.

Signature of person who obtained consent: ____________________Date: ___________

GENETIC RESEARCH STUDY

In addition to this test providing clinical information for your medical condition, we are also inviting you to participate in a research study. We are inviting you because you are undergoing a genetic sequencing test, and we wish to use your sequencing data for genetic research. The amount of genetic information obtained from this test is vast, including relevant findings that are not associated with your current condition. As such, the results from your test are an important resource for investigators focused on the genetic components of human diseases. The rest of this document describes the research study and what it would mean for you to participate.

A. WHAT IS THE PURPOSE OF THE RESEARCH STUDY?

The purpose of this research study is to investigate how genes affect health and disease.

B. HOW MANY PEOPLE WILL PARTICIPATE?

Approximately 10,000 people will take part in this study at the University of Iowa.

C. HOW LONG WILL I BE IN THIS STUDY?

Participation in the research study does not require any extra time or effort beyond what you have already given for the clinical sequencing test. If you agree to take part in this study, however, the length of your involvement will be indefinite, as we will continue to use the genetic data and DNA that you provide into the future.

D. WHAT WILL HAPPEN DURING THIS RESEARCH STUDY?

1. Your Sequencing Data Will Be Stored in a Database and Analyzed

By participating in this research study you are allowing the genetic information from your sequencing test to be placed in a database that we have created. We will also collect clinical information related to your medical condition both from you directly and from your medical records. We will connect the genetic data to the clinical information to look for relationships that will help us better understand your medical condition. We will also compare your genetic and clinical data to data from other people with and without your medical condition to try to identify genetic patterns related to various diseases. The results of any research study performed on your data will not be included in your medical record. Only a select group of individuals will have access to your name and medical records.

2. We Will Store Your DNA and Genetic Data for Future Use

As part of the clinical sequencing test, we are obtaining DNA from you. We may wish to study your DNA in the future, after the sequencing is complete. Therefore, we are asking for your permission to store your DNA and genetic information so we can study them in

the future. It is possible that your genetic data might be used to develop products or tests that could be patented and licensed. There are no plans to provide financial compensation to you should this occur.

Blood cells removed from the blood samples can be used to make a cell line and DNA. Cell lines are produced by growing blood cells in a laboratory and allow us to have a source of the DNA without having to redraw your blood. These blood cells can be stored for decades or more. The cell lines and DNA and data will be made available to researchers trying to learn more about the cause of diseases.

Your sample, information, and/or data may also be placed in a national repository sponsored by the National Institutes of Health or another Federal agency. If this occurs, your data will stripped of identifying information such as name, date of birth, address, etc. Other qualified researchers who obtain proper permission may gain access to your sample and/or data for use in approved research studies that may or may not be related to the purpose of this study.

If you agree now to future use of your DNA and genome data, but decide in the future that you would like to have it removed from future research, you should contact Richard Smith (319)336-2177. However, if some research with your DNA and genome data has already been completed, the information from that research may still be used.

E. GENETIC INFORMATION NONDISCRIMINATION ACT (GINA)

A new federal law called the Genetic Information Nondiscrimination Act (GINA) generally makes it illegal for health insurance companies, group health plans, and employers of 15 or more persons to discriminate against you based on your genetic information. Based on this new law, health insurance companies and group health plans are prohibited from requesting your genetic information that we get from this research. This means that they may not use your genetic information when making decisions regarding your eligibility for insurance coverage or the amount of your insurance premiums. Be aware that this new federal law will not protect you against genetic discrimination by companies that sell life insurance, disability insurance, or long-term care insurance. The law also does not prohibit discrimination if you already have a manifest genetic disease or disorder.

F. WHAT ARE THE RISKS OF THIS TEST AND RESEARCH?

One risk of participation may be feelings of pressure from other family members. Your participation is voluntary and will not impact your medical care.

Another risk of giving samples for this research may be the release of your name and other identifying information that could link you to the stored samples and/or the results of the tests run on your sample. To prevent this, your samples and genetic data will be given a unique code number. Only the study staff will know the code number. The name

that belongs to the code number will be kept in a locked file or in a computer with a password. Only Richard JH Smith will be able to match your name and code number.

G. BENEFITS OF THIS TEST AND RESEARCH

You are not likely to benefit personally from being in this study. We hope that the database will be a helpful tool for determining how specific genetic variants cause or prevent human disease. The knowledge gained may therefore lead to improved diagnostic tests and treatments for a variety of human diseases.

H. WILL IT COST ME ANYTHING TO BE IN THIS STUDY?

- You will have **no additional costs** for being in the research study.
- You will not receive compensation for participating in this research study.

I. WHAT ABOUT CONFIDENTIALITY?

We will keep your participation in this research project confidential to the extent permitted by law. However, it is possible that other people may become aware of your participation in this study. For example, federal government regulatory agencies and Institutional Review Boards (committees that review and approve research studies) at the University of Iowa and the National Institute of Health may inspect and copy records pertaining to this research. Some of these records could contain information that personally identifies you.

Your DNA will be assigned a code number so that you cannot be identified from data analyses or published reports. Your code number can be traced to your identifying information, but only by people working directly on the project. If we share DNA samples with other laboratories, your identity will not be disclosed. If you withdraw from the study, we will destroy the DNA from your original blood sample, the tissue from your biopsy, and any cell lines that we have made. Because your identity will not have been included with DNA or cells that we have shared with others, however, such DNA and cells would not be able to be destroyed.

J. WILL MY HEALTH INFORMATION BE USED DURING THIS STUDY?

The Federal Health Insurance Portability and Accountability Act (HIPAA) requires University of Iowa Health Care to obtain your permission for the research team to access or create “protected health information” about you for purposes of this research study. Protected health information is information that personally identifies you and relates to your past, present, or future physical or mental health condition or care. We will access or create health information about you, as described in this document, for purposes of this research study and for your healthcare. Once your health care provider has disclosed your protected health information to us, it may no longer be protected by the Federal HIPAA privacy regulations, but we will continue to protect your confidentiality as described under “Confidentiality.”

We may share your health information related to this study with other parties including federal government regulatory agencies, the University of Iowa Institutional Review Boards and support staff.

You cannot participate in this research study unless you permit us to use your protected health information. If you choose *not* to allow us to use your protected health information, we will discuss any non-research alternatives available to you. Your decision will not affect your right to medical care that is not research-related. Your signature on this Consent Document authorizes your health care provider to give us permission to use or create health information about you.

Although you may not be allowed to see study information until after this study is over, you may be given access to your health care records by contacting your health care provider. Your permission for us to access or create protected health information about you for purposes of this study has no expiration date. You may withdraw your permission for us to use your health information for this research study by sending a written notice to Richard Smith, MD, Sterba Hearing Research Professor, Director – Iowa Institute of Human Genetics, Vice Chair - Department of Otolaryngology, 200 Hawkins Drive, University of Iowa, Iowa City, IA 52242. However, we may still use your health information that was collected before withdrawing your permission. Also, if we have sent your health information to a third party, such as the study sponsor, or we have removed your identifying information, it may not be possible to prevent its future use.

K. IS BEING IN THIS STUDY VOLUNTARY?

Taking part in this test and research study is completely voluntary. You may choose not to take part at all. If you decide to be in this study, you may stop participating at any time. If you decide not to be in this study, or if you stop participating at any time, you won't be penalized or lose any benefits for which you otherwise qualify.

L. WHAT IF I DECIDE TO DROP OUT OF THE STUDY?

There are not any adverse consequences if you decide to withdraw from the research study. Withdrawal from the research study will not impact your participation in the sequencing test for the primary indication. If you decide to leave the study early, we will ask you to contact Richard Smith, MD, Sterba Hearing Research Professor, Director – Iowa Institute of Human Genetics, Vice Chair - Department of Otolaryngology, 200 Hawkins Drive, University of Iowa, Iowa City, IA 52242.

M. WHAT IF I HAVE QUESTIONS?

We encourage you to ask questions. If you have any questions about the research study itself, please contact: Dr. Richard JH Smith at (319) 356-3612.

If you have questions, concerns, or complaints about your rights as a research subject or

about research related injury, please contact the Human Subjects Office, 105 Hardin Library for the Health Sciences, 600 Newton Rd, The University of Iowa, Iowa City, IA 52242-1098, (319) 335-6564, or e-mail irb@uiowa.edu. General information about being a research subject can be found by clicking "Info for Public" on the Human Subjects Office web site, http://research.uiowa.edu/hso. To offer input about your experiences as a research subject or to speak to someone other than the research staff, call the Human Subjects Office at the number above.

N. SIGNATURES

This Informed Consent Document is not a contract. It is a written explanation of what this testing involves if you decide to participate. You are not waiving any legal rights by signing this Informed Consent Document. Your signature indicates that this research has been explained to you, that your questions have been answered, and that you agree to participate. You will receive a copy of this form.

Subject's Name (printed): ____

Signature: _______________________________________Date: ________________

Parent/Guardian or Legally Authorized Representative's Name and Relation to Subject:

Name: ___________________________Relationship to Subject:_________________

Signature: _______________________________________Date: ________________

Statement of Person Who Obtained Consent

I have discussed the above points with the subject or, where appropriate, with the subject's legally authorized representative. It is my opinion that the subject understands the risks, benefits, and procedures involved with participation in this testing.

Signature of person who obtained consent: ____________________Date: __________

What is exome sequencing?
In the past it was only possible to screen one gene at a time to determine the cause of a disease. While this method is still relevant for many diseases it can be costly and time consuming. However, recent advances in technology make it possible to screen a person's entire "exome" at once.

What is your exome?
The exome is the part of the genome (all your DNA) that includes all the exons, or the portions of each gene that are expressed as proteins.

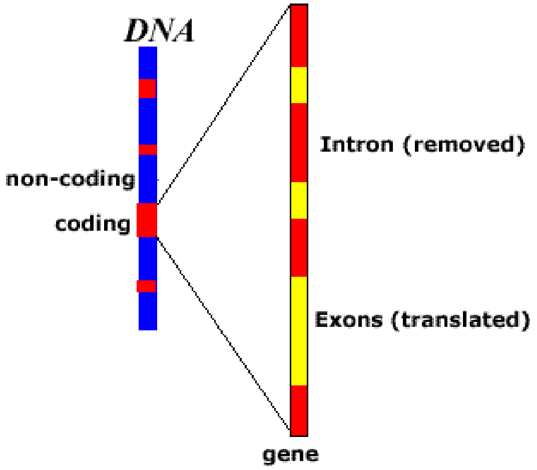

Exons are the most functionally relevant part of the genome. Exons make up about 1% of the human genome. Importantly, majority of known disease-causing mutations are currently found in the exome.

Why is my doctor suggesting this test?
Genes passed down to us from our parents are not only responsible for things like our height or hair color, they can also cause or predispose us to disease. By sequencing your DNA doctors may be able to find a genetic cause for your disease or health condition.

The identification of a genetic cause of a disease **may or may not** impact your medical care in the following ways;

- Provide a cause for your disease or symptoms
- Assist you and your healthcare provider chose the best treatment for you.
- Determine recurrence risks of disease in you and your family

What will happen to my sample?
We will need a one-time blood draw so that we can extract DNA to be sequenced.

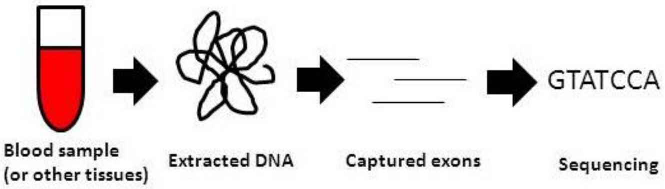

You may be asked to give an additional sample of blood or saliva if the initial sample does not provide enough DNA for the test.

How will the results be given to me?
A doctor and/or a genetic counselor will contact you with your results so they can explain the results and answer any questions you may have. The report physicians will receive will contain a recommendation section which will provide screening and test recommendations based on the genetic findings.

In addition, if you decide and indicate on the consent form there is other information you would like to know regarding health conditions other than your initial disease the report will also contain that information.

Genetic Testing Possible Results

Types of results

1) Positive: A change in the DNA that is well characterized and known to cause disease has been detected.

2) Negative: There are no changes in the genes that were screened.

3) Variant of uncertain (clinical) significance (VUS): A change in a gene known to cause disease was identified. However, this change has never been seen before. Alternatively, a change in a gene which is not yet known to cause disease. These are usually *not* reported.

Types of findings

1) Primary findings: Changes in DNA that either likely cause or predispose to the condition for which you were tested.

2) Secondary findings: Changes in DNA that could influence future medical care that are unrelated to the reason you received this test. You will be able to choose whether or not to receive this type of genetic information in the consent form.

Limitations

Your genetic counselor will explain the technical and biological limitations of exome sequencing to you during your counseling appointment. These limitations may affect the need to perform additional testing.

Will my insurance company be able to access this information?

Yes, any information in your medical record can be accessed by your insurance company. However, any research results will not be placed in your medical record and will not be accessible to your insurance company. Your genetic counselor can answer any questions you have regarding insurance companies and genetic information.

The Genetic Information Nondiscrimination Act of 2008 (GINA) is a federal law that protects people from genetic discrimination in health insurance and employment. Genetic discrimination is the misuse of genetic information.

Informative Websites

genome.gov
National Human Genome Research Institute
National Institutes of Health

http://www.genome.gov

National Human Genome Research Institute provides information on the Human Genome project including the latest research being performed, educational materials (for students, teachers and patients), and a comprehensive explanation of GINA (Genetic Information Nondiscrimination Act) and your rights concerning genetic discrimination.

Genetics Home Reference
Your Guide to Understanding Genetic Conditions

http://ghr.nlm.nih.gov

Genetics Home Reference provides consumer-friendly information about the effects of genetic variations on human health. There are also helpful tutorials explaining what genes are, how they work, and how they cause disease.

University of Iowa Genome Sequencing Clinical Diagnostic Service Laboratory
http://www.medicine.uiowa.edu/humangenetics/

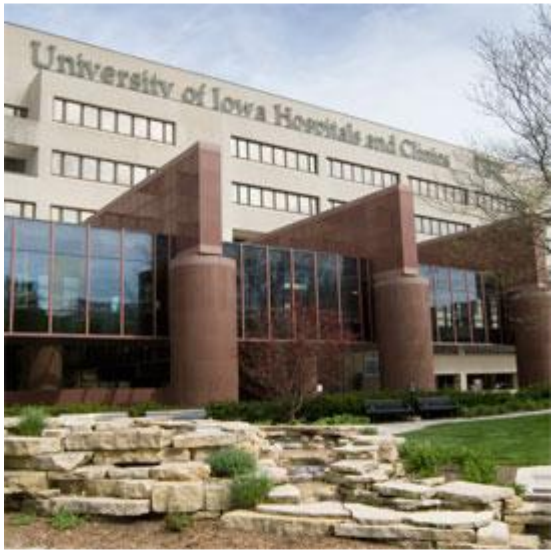

Exome Sequencing: Information for Families

Iowa Institute of Human Genetics

UNIVERSITY of IOWA HEALTH CARE

*Note: This pamphlet may be provided to patients and their families to review prior to meeting with a genetic counselor. Your genetic counselor will explain this information to you and answer any questions you may have.

IOWA INSTITUTE OF
HUMAN GENETICS

1000 NEWTON ROAD
IOWA CITY, IA 52242-1442
TEL 319-335-8000/ 800-335-8100
WWW.CCOM.IIHG.ORG/

Genetic Sequencing Report

Patient/Subject Name: Johnny Q. Doe (FAMILY 1 PROBAND) Date of Birth: not provided_ Gender: M

Ethnicity/Race: White/non-Hispanic Consent category: √minor/□assent/□adult

Medical Record ID#: ______________ Lab ID#: ______________Other DB ID#: ______________

Specimen type: ________________ Date Received: _7/01/2012________ Date Reported: **9/01/2012**

Referring physician: _________________ Referring Genetic Counselor:_________________________

Type of test ordered: x WGS x WES □ Targeted Capture/Amplicon Sequencing □ Other: _______________

Indication for Testing

Patient synopsis: *conceived via IVF with FT NSVD at 38 wks.* Pregnancy complicated by treated hypothyroidism (synthroid, prednisone). *Newborn screen negative; initial difficulty with breastfeeding and delayed fine and gross motor skills since infancy with normal speech and cognitive development. History of recurrent vomiting at age 3-4, now resolved; history of recurrent URI and chronic cough; history of headaches/migraines, possibly related to hypercapnia; progressive scoliosis treated with nighttime brace; mild oromotor dysphagia; flexible flat feet requiring orthotics. Generalized weakness with head lag from birth, exacerbated by stress or illness. Muscle biopsy at age 13 months (hypotonia) showed centronuclear myopathy; feeding difficulties from birth, requires G-tube supplementation. Intermittent hypoventilation treated at nighttime with I-PAP. Mildly elevated carnitine (110; 24-100), and lactic acid (13; 3-12) at age 12 months. Sensorineural hearing loss.*

Clinical Diagnoses Under Consideration (in order of likelihood): congenital myopathy, centronuclear, congenital fiber-type disproportion (CFTD) myopathy, rigid spine; congenital myasthenic syndrome; mitochondrial disorder; FSHD*, Col 6, LMNA, DM1, and DM2*; hearing loss

Known Genetic Associations with the Clinical Diagnosis under Consideration (Known Genes): 18 total - *ACTA1, BIN1, CCDC78, COL6A1, COL6A2, COL6A3, DM1, DM2, DNM2, DOK7, LMNA, MTM1, MTMR14, MYF6, MYH7, RYR1, SEPN1, TPM3* (Note: the phenotype includes hearing loss although the severity is NOT given. Genes associated with non-syndromic hearing should be considered IF the phenotype is considered complex and due to multiple genetic causes.)

Inheritance Models Considered: autosomal recessive, X-linked recessive, and *de novo*

Patient is Consented to Receive:

x Primary Finding
□ Longevity Reporting
□ Secondary – Pharmacogenetics (Other)
□ Findings Relevant to General Medical Care
□ Secondary Findings – Pediatric Onset Treatable
□ Secondary – Carrier Status

□ Secondary – Adult Treatable (Onset) □ Secondary – Adult Non-Treatable (Onset) (Must be Over 18)
□ Obtain Raw Data (Requires Additional Genetic Counseling)
x Research (Participation in Phenome-Genome Database)

Results—Primary Findings & Interpretation

Summary: No pathogenic or likely pathogenic variants were identified in the genes known to be related to the complex clinical condition described on Page 1, however two variants were identified in *GJB2* that are causally associated with autosomal recessive non-syndromic hearing loss at the DFNB1 locus (see Appendix II for details).

These variants are:

1. **Allele 1: *GJB2*: c.35delG**
2. **Allele 2:*GJB2*: c.101T>C; p.M34T**

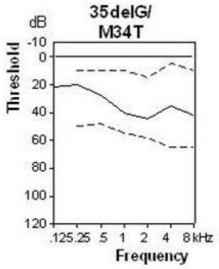

This figure shows the audioprofile associated with the *GJB2* M34T/35delG genotype. The dashed lines represent the audiograms for the 10th and 90th percentiles (adapted from Snoeckx et al AJHG 77:945-958, 2005). Clinical correlation with your patient is recommended.

The above finding suggests that the complex phenotype in this patient MAY NOT be caused by mutations in a single gene. Clinical correlation is recommended. (These results will be available in your patient's electronic medical record.)

The inability to identify causal variants in the 19 genes listed on page 1 that are related to the complex clinical condition may reflect genotype diversity associated with this phenotype, an incomplete understanding of this phenotype, and/or technical limitations of the platform/data used for variant identification (see Data Quality Below). Detailed methodology can be found in **Appendix I: Methods for Analysis and Interpretation; o**ther variants identified in genes related to the clinical phenotype listed on Page 1 are shown in Appendix II.

Genetic Counseling

Genetic counseling is necessary for the patient and family to discuss the issues associated with the absence of reportable primary findings.

If your patient elected to participate in **research related to their primary condition**, please have them contact the University of Iowa Institute for Human Genetics Research Genetic Counselor to discuss research findings.

Data Quality

The detection of variants is dependent on data quality. Only data exceeding a specific threshold are evaluated (see Appendix I). Inadequately covered regions in the coding sequence of known genes are found in Appendix I. In aggregate, the data quality was considered:

□ Excellent 98% or greater of all coding sequence covered by greater than or equal to 4 reads.

□ Very Good 95-97.99% of all coding sequence covered by greater than or equal to 4 reads.

x Good 90-94.99% of all coding sequence covered by greater than or equal to 4 reads.

□ Fair 85-89.99% of all coding sequence covered by greater than or equal to 4 reads.

□ Poor <85% of all coding sequence covered by greater than or equal to 4 reads.

Note: Poor data do not preclude identifying a disease-causing variant but do mean that for a sizable percentage of coding sequence, there was NO analysis and therefore NO conclusions can be made for these regions.

Results- Secondary Findings Relevant to General Medical Care

Variants in some genes may affect a person's response to different medical therapies. At the request of your patient, variants that can impact response to specific medications, radiation therapy or coagulation/clotting are provided. For assistance in interpreting the significance of these variants, please consult the referring Genetic Counselor. These results may or may not be available in your patient's electronic medical record depending if your patient requested these results be included in their electronic medical record.

Pharmacogenomics
As per the challenge we are not reporting these results.

Radiation Therapy
As per the challenge we are not reporting these results.

Coagulation / Clotting
As per the challenge we are not reporting these results.

Longevity Reporting

As more is learned about the role of genetic variants in human health and disease, the interpretation of some of the variants your patient carries may change. Examples of changes include the recognition that a variant believed to be pathogenic is in fact benign, and reclassifying a benign or uncertain variant as pathogenic. At the request of your patient, the Iowa Institute of Human Genetics (IIHG) will (or will not) provide Longevity Reporting by re-analyzing genomic data for the **PRIMARY CONDITION** on an annual basis. These reports will be provided as in the medical record as addendums to the original report and will be available to you when you see your patient in annual follow-up. Please see Addendum III for report updates. For assistance in the interpretation of new data, please consult the referring Genetic Counselor.

Disclaimer/Legal

Genetic testing was conducted with informed consent following certified genetic counseling, including the use of deidentified information for current and future investigational purposes. Patients have the option of changing the status of their consent at any time, which may result in the permanent removal of their information from the IIHG Clinical Diagnostic Service Laboratory information system. If Longevity Reporting has been requested, data are annually reviewed to determine whether there is a change of status for any variants associated with the primary condition. Patients requesting Longevity Reporting will be notified of any change in the status of variants at their annual medical visit.

The performance characteristics of this test were determined by the IIHG Clinical Diagnostic Service. This test has not been cleared or approved by the U.S. Food and Drug Administration (FDA), however the FDA has determined that clearance or approval is unnecessary. This test is for clinical purposes and should not be

regarded as investigational or for research. The IIHG Clinical Diagnostic Service is certified under the Clinical Laboratory Improvement Amendments of 1988 (CLIA; revised 2003) as qualified to perform high complexity clinical laboratory testing.

The results for the Primary Finding and Longevity Reporting for the Primary Finding will be included in the patient's electronic medical record. All other results may or may not be available in your patient's electronic medical record depending if your patient requested these results be included in their electronic medical record.

References

1. OMIM: http://omim.org/

2. NCBI dbSNP135 (http://www.ncbi.nlm.nih.gov/projects/SNP/)

3. 1000 Genomes project: October 2011 release (http://www.1000genomes.org/)

4. Exome Variation Server 6500: accessed on June 20, 2012 (http://evs.gs.washington.edu/EVS/)

Supplemental/Addendum Reports

Please find the following attached, dated documents on the IIHG Clinical Diagnostic Service Laboratory website. Appendices are additional information included in all reports and are part of the electronic medical record. Addendums are optional supplemental reports based on patient consent choices and are not part of the electronic medical record. Addendums are only available on the IIHG Clinical Diagnostic Service Laboratory web portal. All appendices and addendums are dated to reflect knowledge and interpretation at the time of dating.

Appendices

Appendix I. Methods for Analysis and Interpretation

Appendix II. Primary Findings Variant Details

Appendix III. Consent Updates

Addendums – Consent Based Findings Only Available on NGS Dashboard, Not Part of this Report

Addendum I. Research Findings Related to the Primary Condition

Addendum II. Secondary Findings (Optional)

Addendum III. Longevity Reports (Consent Driven)

Addendum IV. Obtain Raw Data (Requires Additional Genetic Counseling)

APPENDIX I. METHODS FOR ANALYSIS AND INTERPRETATION

DNA sequencing—0.5-1.0 µg DNA was prepared from the specimen and processed to generate a library of DNA fragments approximately 150 bp in length. These fragments were attached to microspheres and amplified prior to DNA sequencing using paired-end 75bp chemistry on the ABI SOLiD 5500XL platform (Life Technologies/ Applied Biosystems, Carlsbad, CA).

Raw data processing: Quality control parameters applied to raw sequence data include individual base calling (>99% accuracy) and read length (>50 bp) using software provided by the manufacturer. Raw data output files were archived on a protected storage server and routed into an optimized Galaxy Analysis pipeline for downstream analysis.

Bioinformatics analysis: Sequencing reads were aligned to the human reference genome (GRch37, hg19) using the mapreads tool in Lifescope v2.5 to generate BAM files. BAM files were then processed through a custom analysis pipeline in the Galaxy framework including read duplicate identification (Picard tools), indel realignment (GATK), and variant calling with GATK. Coverage analysis for QC was completed using Bedtools. Variant annotation and filtering was completed using a custom variant annotation pipeline that incorporates data from several repositories (1000 Genomes October 2011 release, EVS accessed on June 20, 2012, and dbSNP135) as well as several pathogenicity prediction methods (PolyPhen2, MutationTaster, SIFT, LRT and dbNSFP).

Data quality analysis and filtering: We required 4 sequencing reads to cover each variant position (4X depth of coverage), and the variant to be present in at least 25% of reads. We filtered out variants with < 30 Phred-like quality score (1/100 chance of error) and/or a quality divided by depth (Q/D) of <5. Variants were filtered based on several inheritance models, but we tolerated missing data in 1 of the parents in a trio without exclusion. Variants were also filtered based on allele frequencies in population-level databases specific to each disease or suspected disease under consideration. For centronuclear myopathy, all variants with a MAF ≥0.01 in 1000Genomes and ≥0.006 in EVS were filtered out.

Inheritance models considered: (*select appropriate)*

□ Autosomal dominant	x X-linked recessive	□ Mitochondrial
x Autosomal recessive	□ X-linked dominant	x *de novo*

Copy Number Variant identification: Copy number variant (CNV) locations were ascertained from inspection of the annotated whole genome sequencing data provided. Several sources of information were used to classify CNVs as either clinically benign or potentially pathogenic including the Database of Genomic Variants (DGV; http://projects.tcag.ca/variation/), the Wellcome Trust Sanger Institute's DECIPHER database (http://decipher.sanger.ac.uk/), the International Standards for Cytogenetic Arrays (ISCA) consortium (https://www.iscaconsortium.org/), the CNV datasets published by Conrad et al. 2009 and Cooper et al. 2011, as well as our internal CNV database. If a CNV has been classified as benign, it is not considered to be related to the patient's phenotype. CNVs are classified as benign when:

1. Present in the DGV at a frequency of >1% in >1 studies
2. Present in DGV at a frequency of >0.5% in >2 studies
3. Present in our internal clinical chromosomal microarray database at a frequency of >1% (>1500 cases and >200 unaffected parents)
4. Interpreted as BENIGN in >5 cases in the ISCA database
5. Present in the dataset released in Conrad et al. 2009 and in the same orientation
6. Present in a substantial number of control individuals (>50-100) compared to cases in Cooper et al. 2011.

Any CNV classified as benign is NOT investigated further. Remaining CNVs that contain at least one validated or reviewed RefSeq gene are classified as either ABNORMAL or as a variant of uncertain significance (VUS). ABNORMAL CNVs are known to be pathogenic (such as a 22q11.2 deletion) or novel and >1Mb in size. VUS are either known susceptibility loci (such as 16p11.2 locus) or otherwise NOT classified as ABNORMAL.

ABNORMAL and VUS CNVs are tested for segregation in the family. CNVs that are NOT benign and segregate with the clinical phenotype are reported as LIKELY PATHOGENIC.

Limitations:

1. Variations larger than 30bp and less than several kilobases are undetectable by both the small insertion/deletion calling and the CNV calling pipelines. This would cause, for instance, an insertion of a retrotransposon to not be reported.
2. Phase information is inferred by sequencing family members, it is not directly observed. This will cause the over-reporting of genes with compound heterozygous mutations when only a single individual is sequenced.
3. Small variations in introns, in UTR, and in regulatory regions are not considered except in cases where splicing would be affected.
4. Identified variations are of lower quality in regions that are highly similar to other parts of the genome. This can cause variations to be missed.
5. Gene annotations and exome definitions are incomplete; this causes disease-causing mutations to be missed.

Inadequately Covered Loci (genes, exons that did not meet Q/C for coverage and are relevant to DDx):

The following regions in the coding sequence of genes known to be related to the clinical diagnosis under consideration were inadequately covered and therefore clinical grade variants in these regions may be present but were not detected.

Gene	Position	Median Cover	Minimum Cover
ACTA1	chr1:229568016-229568178	7.1	1
BIN1	chr2:127809830-127809938	6.0	1
BIN1	chr2:127811480-127811588	68.7	1
BIN1	chr2:127816586-127816731	9.7	3
BIN1	chr2:127864435-127864519	0.0	0
CCDC78	chr16:774321-774509	8.9	1
CCDC78	chr16:774910-774989	7.1	1
CCDC78	chr16:775077-775145	18.6	1
CCDC78	chr16:775965-776085	10.5	1
CCDC78	chr16:776307-776367	7.9	3
DNM2	chr19:10828918-10829079	1.6	1
DNM2	chr19:10893635-10893796	11.5	2
DNM2	chr19:10940802-10941054	15.8	2
MTMR14	chr3:9691267-9691426	21.0	1
MTMR14	chr3:9730627-9730766	6.3	2
MYF6	chr12:81101498-81102017	31.8	1
MYH7	chr14:23885212-23885521	56.2	1
MYH7	chr14:23893977-23894233	24.4	2
RYR1	chr19:38938994-38939151	9.1	1
RYR1	chr19:38948690-38948932	7.3	2
RYR1	chr19:38951014-38951231	44.0	2

RYR1	chr19:38956730-38957038	10.1	1
RYR1	chr19:38959605-38959780	18.8	1
RYR1	chr19:38964016-38964411	9.9	1
RYR1	chr19:38973929-38974156	9.3	1
RYR1	chr19:38976229-38976842	8.6	1
RYR1	chr19:38979816-38980083	19.6	1
RYR1	chr19:38984991-38985265	11.1	2
RYR1	chr19:38990274-38990461	8.1	2
RYR1	chr19:38993519-38993610	4.6	2
RYR1	chr19:39002884-39003123	16.6	2
RYR1	chr19:39007998-39008331	18.0	1
RYR1	chr19:39009853-39010094	13.5	1
RYR1	chr19:39013667-39013755	7.0	1
RYR1	chr19:39017632-39017692	5.3	3
RYR1	chr19:39051752-39052094	13.5	1
RYR1	chr19:39055598-39056411	5.2	1
SEPN1	chr1:26126721-26126904	0.0	0
SEPN1	chr1:26128506-26128608	0.0	0
TPM3	chr1:154131444-154131524	5.4	2
TPM3	chr1:154155463-154155595	8.3	1

REFERENCES

1000 Genomes Project Consortium. A map of human genome variation from population-scale sequencing. Nature **467**, 1061-1073, 2010. Data release October 2011.

Adzhubei IA, Schmidt S, Peshkin L, Ramensky VE, Gerasimova A, Bork P, Kondrashov AS, Sunyaev SR. A method and server for predicting damaging missense mutations. Nat Methods **7**(4):248-249 (2010).

Chun and Fay. Identification of deleterious mutations within three human genomes. *Genome Research* (2009) 19:1553-1561.

Conrad DF, Pinto D, Redon R, Feuk L, Gokcumen O, Zhang Y, Aerts J, Andrews TD, Barnes C, Campbell P, Fitzgerald T, Hu M, Ihm CH, Kristiansson K, Macarthur DG, Macdonald JR, Onyiah I, Pang AW, Robson S, Stirrups K, Valsesia A, Walter K, Wei J; Wellcome Trust Case Control Consortium, Tyler-Smith C, Carter NP, Lee C, Scherer SW, Hurles ME. Origins and functional impact of copy number variation in the human genome. Nature. 2010 Apr 1;464(7289):704-12.

Cooper GM, Coe BP, Girirajan S, Rosenfeld JA, Vu TH, Baker C, Williams C, Stalker H, Hamid R, Hannig V, Abdel-Hamid H, Bader P, McCracken E, Niyazov D, Leppig K, Thiese H, Hummel M, Alexander N, Gorski J, Kussmann J, Shashi V, Johnson K, Rehder C, Ballif BC, Shaffer LG, Eichler EE. A copy number variation morbidity map of developmental delay. Nat Genet. 2011 Aug 14;43(9):838-46.

Exome Variant Server, NHLBI GO Exome Sequencing Project (ESP), accessed June 20, 2012. Seattle, WA (URL: http://evs.gs.washington.edu/EVS/) [08/2012].

Kumar P, Henikoff S, Ng PC. Predicting the effects of coding non-synonymous variants on protein function using the SIFT algorithm. *Nat Protoc. 2009*;4(7):1073-81.

Liu X, Jian X, Boerwinkle E. dbNSFP: a lightweight database of human nonsynonymous SNPs and their functional predictions. Hum Mutat. 2011 Aug;32(8):894-9.

Schwarz JM, Rödelsperger C, Schuelke M, Seelow D**.** MutationTaster evaluates disease-causing potential of sequence alterations. Nat Methods. 2010 Aug;7(8):575-6*.*

APPENDIX II. PRIMARY FINDINGS VARIANTS DETAILS (Known Genetic Associations with the Clinical Diagnosis)

Patient/Subject Name: Johnny Q. Doe (FAMILY 1 PROBAND) Date of Birth: not provided Gender: M

Ethnicity/Race: White/non-Hispanic Consent category: √minor/□assent/□adult

Medical Record ID#: ______________ Lab ID#: ______________Other DB ID#: ______________

Specimen type: ________________ Date Received: _7/01/2012________ Date Reported: **9/01/2012**

Referring physician: _________________ Referring Genetic Counselor:_________________________

Type of test ordered: x WGS x WES □ Targeted Capture/Amplicon Sequencing □ Other: ______________

Variants Identified in Genes Associated with the Complex Primary Phenotype

No variants were identified in primary disease genes that met inheritance criteria.

Variants Identified in Genes Associated with Non-Syndromic Hearing Loss

Two variants were identified in GJB2 that are associated with autosomal recessive non-syndromic hearing loss.

GENE	Proband Het/Homo	Type	HGVS_NT	HGVS_PROTEIN	EVS 5400 MAF	1000G MAF	dbSNP135	Chr	Start	End	Ref	Obs	Proband %Obs	Proband Cov	Q_VAR	QD
GJB2	heterozygote	frameshift	NM_004004:c.35delG	NP_003995.2: p.G12fs	na	0.0024	rs80338939	Chr13	20763686	20763686	C	-	37.5%	24	607.88	7.89
GJB2	heterozygote	missense	NM_004004:c.101T>C	NP_003995.2: p.M34T	0.009946	0.0082	rs35887622	Chr13	20763620	20763620	A	G	35.3%	34	882.56	12.26

APPENDIX III. CONSENT, CONSENT UPDATES, NEW HEALTH CONDITIONS

This section includes ORIGINAL consent information and CONSENT UPDATES. Consent updates reflect a change in the information the patient does or does NOT wish to receive. Also included are new health conditions for which the patient is requesting genome analysis. A new abbreviated consent document is required to provide results pertaining to the new condition.

Patient/Subject Name: Johnny Q. Doe (FAMILY 1 PROBAND) Date of Birth: not provided Gender: M

Ethnicity/Race: White/non-Hispanic Consent category: √minor/□assent/□adult

Medical Record ID#: ______________ Lab ID#: ______________Other DB ID#: ______________

Specimen type: ________________ Date Received: _7/01/2012_________ Date Reported: **9/01/2012**

Referring physician: _________________ Referring Genetic Counselor:________________________

Type of test ordered: x WGS x WES □ Targeted Capture/Amplicon Sequencing □ Other: ______________

Original Consent:

_________________ consented to receive the following results on ___ /_____ / ____, for ________________.

Patient Name Day / Month / Year Condition

Results Patient is Consented to Receive:

x Primary Finding
□ Secondary Findings Relevant to General Medical Care
□ Longevity Reporting
□ Secondary Findings – Pediatric Onset Treatable
□ Secondary – Pharmacogenetics (Other)
□ Secondary – Carrier Status
□ Secondary – Adult Treatable (Onset)
□ Secondary – Adult Non-Treatable (Onset) (Must be Over 18)
x Research (Participation in Phenome-Genome Database)
□ Obtain Raw Data (Requires Additional Genetic Counseling)

Special Instructions: ____

____

Consent Updated ___ /_____ / ____, for ________________.

Day / Month / Year Condition

Results Patient is Consented to Receive:

□ Primary Finding
□ Secondary Findings Relevant to General Medical Care
□ Longevity Reporting
□ Secondary Findings – Pediatric Onset Treatable
□ Secondary – Pharmacogenetics (Other)
□ Secondary – Carrier Status
□ Secondary – Adult Treatable (Onset)
□ Secondary – Adult Non-Treatable (Onset) (Must be Over 18)
□ Research (Participation in Phenome-Genome Database)
□ Obtain Raw Data (Requires Additional Genetic Counseling)

Special Instructions: ____

ADDENDUM I. RESEARCH FINDINGS RELATED TO THE PRIMARY CONDITION

(ONLY AVAILABLE THROUGH WEBSITE HEALTHCARE PROVIDER PORTAL)

Patient/Subject Name: Johnny Q. Doe (FAMILY 1 PROBAND) Date of Birth: not provided Gender: M

Ethnicity/Race: White/non-Hispanic Consent category: √minor/□assent/□adult

Medical Record ID#: _____________ Lab ID#: _____________Other DB ID#: _____________

Specimen type: _______________ Date Received: _7/01/2012________ Date Reported: **9/01/2012**

Referring physician: ________________ Referring Genetic Counselor:_______________________

Type of test ordered: x WGS x WES □ Targeted Capture/Amplicon Sequencing □ Other: ______________

Whole exome variant analysis for this family was performed by filtering out all synonymous and intronic variants (except for splice site variants) and those that do not meet the mode of inheritance and MAF requirements. Variants in 5 genes (*PRAMEF22, TIGD6, TTN, OR2T12 and ZNF804A*) for the recessive mode of inheritance and 162 genes for the *de novo* model were found in this family.

In order to identify a subset of promising candidate genes and variants with biological relevance to the disorder in this cohort of 167 genes, we generated in parallel a secondary list of genes. To generate this list, we used the primary genes involved in this disorder as seeds to mine for interacting genes using a panel of databases and analysis tools that would generate a dataset of known and predicted protein-protein interactions as well as shared networks and pathways.

1- STRING: http://string-db.org/newstring_cgi/show_input_page.pl?UserId=bI0i5NfHQOSk&sessionId=zv0H2bheWIlW
2- KEGG PATHWAY Database: http://www.genome.jp/kegg/pathway.html
3- GeneGo: http://www.genego.com/
4- GeneDecks V3/ Partner Hunter: http://www.genecards.org/index.php?path=/GeneDecks

We also used the Mammalian phenotype browser for mouse models with similar phenotype to the disorder investigated in this study (http://www.informatics.jax.org/searches/MP_form.shtml).

The secondary gene list for centronuclear myopathy comprises 92 genes:

ABL1, ACTC1, ACTG1, ADARB1, AFG3L2, AKT1, ALS2, AMPH, AP2A1, AP2M1, AP2S1, APP, AR, ARF6, BELLA, CALM1, CAPN3, CAV1, CAV2, CHKB, CLTB, CNTN1,COMP, COX15, CRYAB, CTNNB1, DAB2, DAG1, DES, DMD, DNM1, DNM3, DYNC1H1,DYSF, E2F1, EGFR, EPN1, EPS15, ERBB2, ERBB3, FBN2, FKBP1A, FKRP, FLNC, GRB2, HIP1, HRAS, HSPG2, IGHMBP2, ITGA7, KIT, LAMA2, LARGE, LMNA, MAPK1, MBNL1, MBNL2, MEOX2, MMP14, MTOR, MYC, MYF5, MYH4, MYOD1, NRG1, OBSCN, PFKM, PLEC, POMGNT1, PTK2, PTPRD, RAB5A, RYR3, SEPN1, SGCA, SGCB, SH3GL2, SHC1, SMN1, SRC, SRPK3, SYNE1, TCAP, TERC, TGFB1, TLN2, TP53, TRIM32, TRIM72, TTN, UTRN and *VCP.*

A subset of 5 genes is shared between both lists: *TTN, TCAP, CNTN1, DYNC1H1 and RYR3.*

List of gene variants found in Family 1

Gene	Porband Het/Homo	Type	HGVS_NT	HGVS_PROTEIN	EVS 5400 MAF	1000 G MAF	db SNP	PhyloP	SIFT	PolyPhen2	LRT	MutationTaster	GERP++	Chr	Start	End	Ref	Obs	Proband % Obs	Proband Cov	Q_Var	QD
**de novo mode of inheritance**																						
RYR3	Het	NS	NM_001036:c.9433T>C	NM_001036:p.Trp3145Arg				C		D	D	D	5.13	chr15	34078027	34078027	T	C	40%	5	30.21	6.04
TCAP	Het	NS	NM_003673:c.445C>T	NM_003673:p.Pro149Ser				C	D	D	D	D	5.12	chr17	37822303	37822303	C	T	40%	5	30.45	6.09
CNTN1	Het	NS	NM_001843:c.1730A>G	NM_001843:p.His577Arg				C	D	D	D	D	4.88	chr12	41352962	41352962	A	G	31%	16	106.7	6.67
DYNC1H1	Het	NS	NM_001376:c.1871A>G	NM_001376:p.Lys624Arg				C	D	P	D	D	5.5	chr14	102452433	102452433	A	G	31%	16	117.1	7.32
**Recessive mode of inheritance**																						
TTN	Het	NS	NM_133378:c.30443C>T	NM_133378:p.Pro10148Leu						B			5.13	chr2	179542464	179542464	G	A	38%	26	593	6.98
TTN	Het	NS	NM_133437:c.13939G>C	NM_133437:p.Gly4647Arg				C		D			5.55	chr2	179506964	179506964	C	G	42%	33	1004	15.45
TTN	Het	splice site	NM_133437:c.18197-1G>A	-				C					5.54	chr2	179487495	179487495	C	T	47%	38	1065	16.39
TTN	Homo	NS	NM_133437:c.9719A>G	NM_133437:p.Lys3240Arg	0.0003			C	NA	D	D	N	5.34	chr2	179629385	179629385	T	C	78%	9	268.8	19.2

C: conserved. D: damaging, P: possibly damaging, B: benign, T: tolerated, N: neutral, NA: not applicable, Het: heterozygote, Homo: homozygote, NS: non-synonymous

***TTN* Gene**

Four variants were found in *TTN* (Titin), a gene encoding a protein abundant in striated muscle and key to muscle assembly, force transmission and maintenance of resting tension. Titin molecules are composed of four main domains, the N-terminal Z-disc and I-band, and the C-terminal A-band and M-line. The Z-disc and M-line regions bind to the Z-line and M-line of the sarcomere so that a single titan molecule spans half of the sarcomere, the repeating contractile unit of muscle. Mutations in *TTN* cause dilated (MIM:604145) and familial hypertrophic (MIM:613765) cardiomyopathies as well as limb-girdle muscular dystrophy (MIM:608807), early-onset myopathy with fatal cardiomyopathy (MIM:611705), proximal myopathy with early respiratory muscle involvement (MIM:603689) and tibial muscular dystrophy (MIM:600334).

Two mutations in *TTN* are predicted to result in truncated proteins. One variant, NM_133437:c.18197-1 G>A is a splicing error and results in a truncated protein, which contains the Z-disc and I-band, but is lacking most of the A-band and all of the M-line. The A-band of titin is an integral part of the myosin filament in the sarcomere (Figure 1, Tskhovrebova L and Trinick J. 2004). This absence, with also the lack of the M-line, would likely lead to decreased titin levels which would limit sarcomere formation. Immunohistochemical studies have shown that some carboxy-terminal truncated titin proteins are integrated in the sarcomere and cause recessive, early onset skeletal and cardiac myopathy (Carmignac V et al. 2007). However, in subjects with dilated cardiomyopathy an unequal mutation distribution of truncated titin proteins are integrated into the sarcomere and are thought to cause dilated cardiomyopathy by means of a dominant-negative mechanism (Herman DS et al. 2012).

The second mutation, NM_13347:c.13939G>C, has been reported in a patient with dilated cardiomyopathy. This nonsense mutation is thought to have a profound effect on the structure of full length titin polypeptides (Herman DS et al. 2012). Both the Analyzer Splice Tool and Human Splice Finder predict that this variant would negatively affect splicing. Taken together, these variants are likely to affect splicing, reduce titin content in the sarcomere, and affect muscle assembly and force transmission.

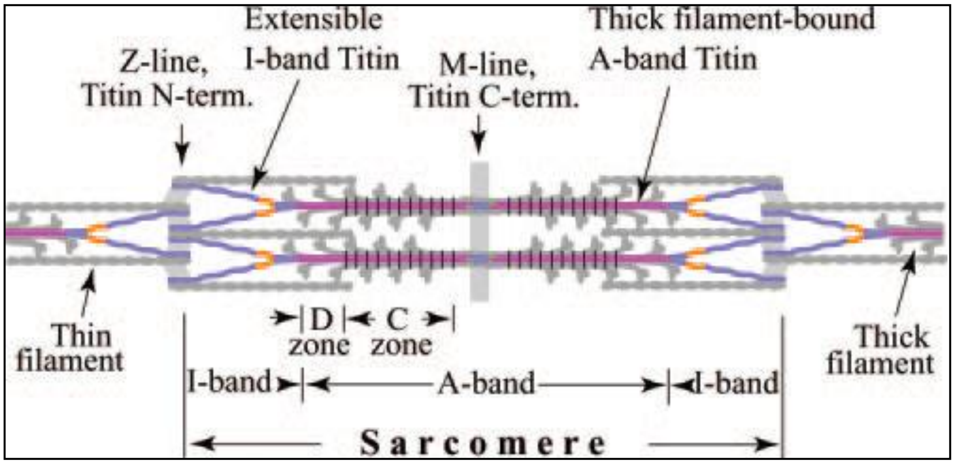

Figure1: Organization of TTN domains in the sarcomere.(Tskhovrebova L and Trinick J. J Biol Chem 2004).

The third variant, NM_133437:c.9719A>G represents a nonsynonymous variant at a conserved residue that is predicted by PolyPhen, LRT and GERP to be damaging. The variant resides in the I-band which is responsible for the elastic connection between the end of the thick filament and the Z-line. The I-band changes its end-to-end distance during muscle contraction and extension (Tskhovrebova L and Trinick J. 2004).

The final variant, NM_133378:c.30443C>T, is likely a benign polymorphism as it not conserved and is not predicted to be pathogenic by several tools (SIFT, PolyPhen, MutationTaster, and LRT).

***RYR3* Gene**

Ryanodine receptors (RyRs) are ion channels located in the sarcoplasmic/endoplasmic reticulum membrane and are responsible for the release of Ca^{2+} during excitation-contraction coupling in both cardiac and skeletal muscle. In skeletal muscle this coupling is dependent on the interaction of RyR with a voltage sensitive dihydropyridine receptors *(DHPR*) also known as L-type Ca^{2+} channels ($Ca_{v1.1}$).(figure 2 and Sheridan DC et al. 2006)

Ryanodine receptors are the largest known ion channels that assemble in homotetrameric proteins. Three isoforms that share both sequence and structural homology have been identified: *RYR1* (expressed in the skeletal muscle), *RYR2* (expressed in the heart) and *RYR3* (expressed in skeletal muscle and brain) (Hakamata et al. 1992).

Given the important physiologic role RyRs play in muscle, alterations in these genes have been linked to several disorders. Both dominant and recessive mutations in *RYR1* have been associated with centronuclear myopathy (MIM:180901) and *RYR2* mutations underlie cardiac diseases (MIM:180902). No disorders have been linked to *RYR3* although in *Ryr3* knockout mice, skeletal muscle contraction is impaired during the first weeks after birth suggesting a role for *RYR3* in excitation-contraction coupling of neonatal skeletal muscles (Bertocchini et al. 1997).

The structural and functional homology between *RYR1* and *RYR3* as well as muscle expression and impaired muscle physiology in the knockout mouse make *RYR3* a good candidate for centronuclear myopathy.

The nonsynonymous *de novo* mutation in *RYR3* found in the proband, NM_001036:c.9433T>C, changes a conserved amino acid tryptophan to an arginine at position 3145. This change occurs in a domain that is believed to interact with the skeletal muscle *DHPR* and is predicted to be pathogenic (PolyPhen, LRT and MutationTaster). It is possible the variant allele might act through a dominant-negative mechanism to alter either the formation of a functional tetrameric channel or the interaction between *RYR3* and *DHPR* thus impairing Ca^{2+} current and functional excitation-contraction coupling.

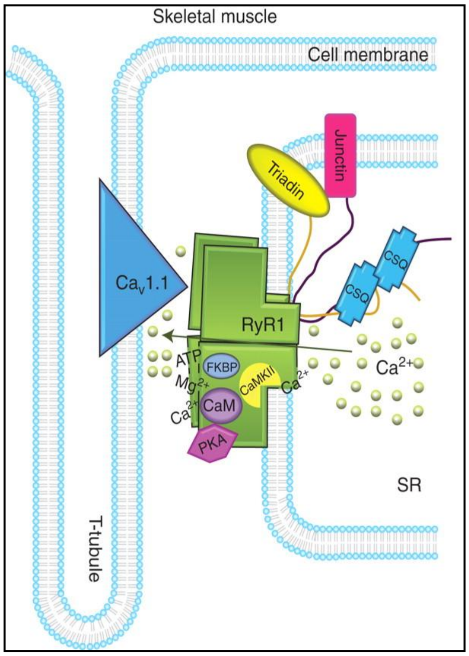

Figure 2: Schematic figure of the interaction between RyR and various modulators in skeletal muscle (Lanner J et al. 2010).

***CNTN1* Gene**

CNTN1 encodes for contactin-1, a neural adhesion and neuromuscular junction protein (NMJ) of the immunoglobulin (Ig) superfamily. Defects in *CNTN1* are the cause of Compton-North congenital myopathy (CNCM) (MIM:612540) (Compton AG et al. 2008). This disorder is a lethal form of recessive congenital onset muscle weakness in humans. It is characterized by a secondary loss of beta2-syntrophin and alpha-dystrobrevin from muscle sarcolemma. Loss of contactin-1 from the NMJ impairs communication or adhesion between nerve and muscle resulting in the severe myopathic phenotype. The *Cntn1* null mouse presents with ataxia and progressive muscle weakness (Berglund EO et al.1999).

The *de novo* variant found in *CNTN1* gene in the proband is NM_001843:c.1730A>G replacing a conserved histidine at position 577 to an arginine. It is predicted to be pathogenic (SIFT, PolyPhen, LRT and MutationTaster) although it remains to be shown how the variant allele could have a dominant-negative effect.

***TCAP* Gene**

TCAP is a muscle-specific titin-capping protein at the Z-disc. Within the sarcomere of muscle, the Titin/Tcap/MLP complex serves as a mechanical stretch sensor. Additionally, the Tcap protein interacts with calsarcin, which tethers the calcineurin to the Z-disc in muscle sarcomere (Mayans O et al. 1998). Mutations in *TCAP* are associated with two different types of diseases: dilated cardiomyopathy and limb-girdle muscular dystrophy, type 2G (MIM:604488) (Hayashi T et al. 2004 and Bos JM et al. 2006). Additionally, *Tcap*-null mice have centrally nucleated skeletal muscle fibers similar to findings in the muscles of LGMD 2G patients (Markert CD et al.2010).

The mutation found in the patient, NM_003673:c.445C>T (p.Pro149Ser) is located between the two known *TCAP* mutations; p.Thr137Ile and p.Arg153His, which have been found in patients with hypertrophic cardiomyopathy (Bos et al. 2006). Another mutation, p.Glu132Gln, has been found in a patient with DCM. It is also known that *TCAP* interacts with *TTN* (figure 3, Anderson PS et al. 2009); it is possible that such interactions could be disrupted by the patient’s variant.

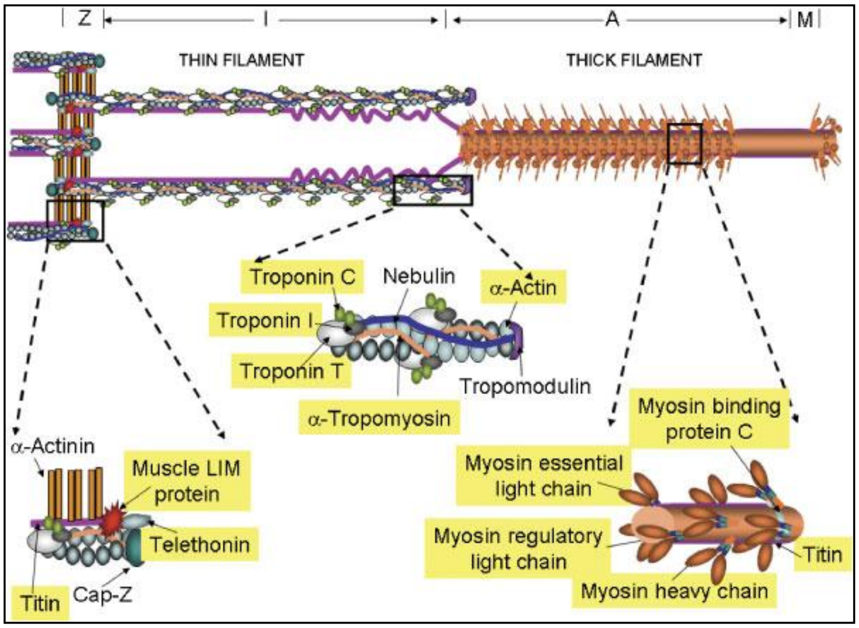

Figure 3: Schematic representation of a half-sarcomere showing arrangement and localization of thick and thin filament proteins. Sarcomere zones and bands are indicated above the diagram, while proteins encoded by genes implicated in HCM are highlighted in yellow (Anderson PS et al. 2009).

***DYNC1H1* Gene**

Mutations in the *DYNC1H1* gene are associated with autosomal dominant Spinal muscular atrophy (SMA) (MIM158600) and Charcot-Marie-Tooth disease (MIM614228). The novel *de novo* variant detected in the proband, NM_001376:p.Lys624Arg, is conserved across species and is predicted to be pathogenic (SIFT, PolyPhen, MutationTaster, and LRT). The variant is within the *DYNC1H1* dimerization domain; missense mutations in this domain have been identified in human cases of CMT or SMA-LED (Tsurusaki Y et al. 2012). The tail domain is thought to be essential for the dimerization of dynein heavy chains and thus missense mutations in this domain may disrupt function of dynein complex formation in a dominant-negative manner (Tsurusaki Y et al. 2012).

INTERPRETATION

The genetic variants described above **may or may not** be associated with the clinical phenotype in this patient. **The relationship of these genes to your patient's phenotype is UNKNOWN.**

These results are considered **RESEARCH FINDINGS** because a definite association of these genes with the condition has not been verified and the results have not been validated with Sanger sequencing. There is NO current robust genetic evidence to implicate these genes with disease.

Based on the information currently available, these are **variants of unclear clinical significance**, which may or may NOT be involved with disease in your patient. We do NOT recommend a change of medical management based on this variant at this time. We encourage your patient to have confirmatory testing by Sanger sequence verification of this variant. In addition, your patient may be interested and/or eligible for participation in further research on this disease.

NOTE

Patients cannot receive these results without first speaking to the IIHG Research Genetic Counselor. If upon speaking with the IIHG Research Genetic Counselor the patient indicates they would like to participate in an active line of research at the University of Iowa, the IIHG Research Genetic Counselor will provide the patient with the contact information of an appropriate Principle Investigator. If the patient would like us to transfer the results of their sequencing test to the researcher, the patient must call or write to the IIHG and request the IIHG Clinical Diagnostic Service Laboratory provide a copy of the genetic sequencing results to the researcher.

REFERENCES

Andersen PS, Havndrup O, Hougs L, Sørensen KM, Jensen M, Larsen LA, Hedley P, Thomsen AR, Moolman-Smook J, Christiansen M, Bundgaard H. Diagnostic yield, interpretation, and clinical utility of mutation screening of sarcomere encoding genes in Danish hypertrophic cardiomyopathy patients and relatives. Hum Mutat 30(3):363-70, 2009.

Berglund EO, Murai KK, Fredette B, Sekerková G, Marturano B, Weber L, Mugnaini E, Ranscht B. Ataxia and abnormal cerebellar microorganization in mice with ablated contactin gene expression. Neuron 24:739-750, 1999.

Bertocchini F, Ovitt CE, Conti A, Barone V, Schöler HR, Bottinelli R, Reggiani C, Sorrentino V. Requirement for the ryanodine receptor type 3 for efficient contraction in neonatal skeletal muscles. EMBO J 16(23):6956-63,

Tskhovrebova L and Trinick J. Properties of titin immunoglobulin and fibronectin-3 domains. J Biol Chem 279(45):46351-4, 2004.

Bos JM, Poley RN, Ny M, Tester DJ, Xu X, Vatta M, Towbin JA, Gersh BJ, Ommen SR, Ackerman MJ. Genotype-phenotype relationships involving hypertrophic cardiomyopathy-associated mutations in titin, muscle LIM protein, and telethonin. Mol Genet Metab 88(1):78-85, 2006.

Carmignac V, Salih MA, Quijano-Roy S, Marchand S, Al Rayess MM, Mukhtar MM, Urtizberea JA, Labeit S, Guicheney P, Leturcq F, Gautel M, Fardeau M, Campbell KP, Richard I, Estournet B, Ferreiro A. C-terminal titin deletions cause a novel early-onset myopathy with fatal cardiomyopathy. Ann Neurol 61(4):340-51, 2012.

Compton AG, Albrecht DE, Seto JT, Cooper ST, Ilkovski B, Jones KJ, Challis D, Mowat D, Ranscht B, Bahlo M, Froehner SC, North KN. Mutations in contactin-1, a neural adhesion and neuromuscular junction protein, cause a familial form of lethal congenital myopathy. Am J Hum Genet 83(6):714-24, 2008.

Hakamata Y, Nakai J, Takeshima H, Imoto K. Primary structure and distribution of a novel ryanodine receptor/calcium release channel from rabbit brain. FEBS Lett 312(2-3):229-35, 1992.

Hayashi T, Arimura T, Itoh-Satoh M, Ueda K, Hohda S, Inagaki N, Takahashi M, Hori H, Yasunami M, Nishi H, Koga Y, Nakamura H, Matsuzaki M, Choi BY, Bae SW, You CW, Han KH, Park JE, Knöll R, Hoshijima M, Chien KR, Kimura A. Tcap gene mutations in hypertrophic cardiomyopathy and dilated cardiomyopathy. J Am Coll Cardiol 44(11):2192-201, 2004

Herman DS, Lam L, Taylor MR, Wang L, Teekakirikul P, Christodoulou D, Conner L, DePalma SR, McDonough B, Sparks E, Teodorescu DL, Cirino AL, Banner NR, Pennell DJ, Graw S, Merlo M, Di Lenarda A, Sinagra G, Bos JM, Ackerman MJ, Mitchell RN, Murry CE, Lakdawala NK, Ho CY, Barton PJ, Cook SA, Mestroni L, Seidman JG, Seidman CE. Truncations of titin causing dilated cardiomyopathy. N Engl J Med 366(7):619-28, 2012.

Lanner JT, Georgiou DK, Joshi AD, Hamilton SL. Ryanodine receptors: structure, expression, molecular details, and function in calcium release. Cold Spring Harb Perspect Biol 2(11):a003996, 2010.

Markert CD, Meaney MP, Voelker KA, Grange RW, Dalley HW, Cann JK, Ahmed M, Bishwokarma B, Walker SJ, Yu SX, Brown M, Lawlor MW, Beggs AH, Childers MK. Functional muscle analysis of the Tcap knockout mouse. Hum Mol Genet 19(11):2268-2283, 2010.

Mayans O, van der Ven PF, Wilm M, Mues A, Young P, Fürst DO, Wilmanns M, Gautel M. Structural basis for activation of the titin kinase domain during myofibrillogenesis. Nature 395(6705):863-9, 1998.

Sheridan DC, Takekura H, Franzini-Armstrong C, Beam KG, Allen PD, Perez CF. Bidirectional signaling between calcium channels of skeletal muscle requires multiple direct and indirect interactions. Proc Natl Acad Sci USA 103(52):19760-5, 2006.

Tsurusaki Y, Saitoh S, Tomizawa K, Sudo A, Asahina N, Shiraishi H, Ito JI, Tanaka H, Doi H, Saitsu H, Miyake N, Matsumoto N. A *DYNC1H1* mutation causes a dominant spinal muscular atrophy with lower extremity predominance. Neurogenetics. 2012 Jul 31 Epub ahead of print.

ADDENDUM II. UNRELATED CONSENTED SECONDARY FINDINGS

The following categories of results are consent-based options that are ONLY available if your patient has requested these results.

Patient/Subject Name: Johnny Q. Doe (FAMILY 1 PROBAND) Date of Birth: not provided Gender: M

Ethnicity/Race: White/non-Hispanic Consent category: √minor/□assent/□adult

Medical Record ID#: ______________ Lab ID#: ______________Other DB ID#: ______________

Specimen type: ________________ Date Received: _7/01/2012________ Date Reported: **9/01/2012**

Referring physician: _________________ Referring Genetic Counselor:________________________

Type of test ordered: x WGS x WES □ Targeted Capture/Amplicon Sequencing □ Other: _______________

A. SECONDARY FINDINGS: PEDIATRIC ONSET TREATABLE CONDITIONS

As per the challenge we are not reporting these results.

B. SECONDARY FINDINGS: PHARMACOGENETICS (OTHER)

As per the challenge we are not reporting these results.

C. SECONDARY FINDINGS: CARRIER STATUS

As per the challenge we are not reporting these results.

D. SECONDARY FINDINGS: ADULT ONSET TREATABLE CONDITIONS

As per the challenge we are not reporting these results.

E. SECONDARY FINDINGS: ADULT ONSET NON-TREATABLE CONDITIONS (MUST BE OVER 18)

As per the challenge we are not reporting these results.

ADDENDUM III. LONGEVITY REPORTS

Patient/Subject Name: Johnny Q. Doe (FAMILY 1 PROBAND) Date of Birth: not provided Gender: M

Ethnicity/Race: White/non-Hispanic Consent category: √minor/□assent/□adult

Medical Record ID#: ______________ Lab ID#: ______________Other DB ID#: _____________

Specimen type: ________________ Date Received: _7/01/2012________ Date Reported: **9/01/2012**

Referring physician: _________________ Referring Genetic Counselor:_________________________

Type of test ordered: x WGS x WES □ Targeted Capture/Amplicon Sequencing □ Other: _______________

If your patient has requested Longevity reporting, the Iowa Institute of Human Genetics (IIHG) will re-analyze genomic data for the **PRIMARY CONDITION** on an annual basis for 7 years. After 7 years testing on a new sample should be considered due to modernized sequencing technology. These reports will be provided as in the medical record as addendums to the original report and will be available to you when you see your patient in annual follow-up. For assistance in interpreting the significance of these variants, please consult the referring Genetic Counselor.

The following variants are now known to be disease causing:

	Gene	Variant (cDNA & Protein annotation)	State (heterozygous vs homozygous)	Frequency European Americans	Frequency African Americans	Disease
1						
2						
3						
4						
5						

The following variants were originally reported to be disease causing, but are now known to be benign polymorphisms:

	Gene	Variant (cDNA & Protein annotation)	State (heterozygous vs homozygous)	Frequency European Americans	Frequency African Americans	Disease
1						
2						
3						
4						
5						

ADDENDUM IV. OBTAIN RAW DATA (REQUIRES ADDITIONAL GENETIC COUNSELING)

Patient/Subject Name: Johnny Q. Doe (FAMILY 1 PROBAND) Date of Birth: not provided Gender: M
Ethnicity/Race: White/non-Hispanic Consent category: √minor/□assent/□adult
Medical Record ID#: ______________ Lab ID#: ______________Other DB ID#: ______________
Specimen type: ________________ Date Received: _7/01/2012________ Date Reported: **9/01/2012**
Referring physician: _________________ Referring Genetic Counselor:________________________
Type of test ordered: x WGS x WES □ Targeted Capture/Amplicon Sequencing □ Other: ______________

_________________ received the following data files on ___ /_____ / ____.

Patient Name Day / Month / Year

Bioinformatics fill in fill type options

□ .BAM files

□ VCF files

□ Other: ____

IOWA INSTITUTE OF
HUMAN GENETICS

1000 NEWTON ROAD
IOWA CITY, IA 52242-1442
TEL 319-335-8000/ 800-335-8100
WWW.CCOM.IIHG.ORG/

Genetic Sequencing Report

Patient/Subject Name: Susan Q. Dough (Family 2 proband) Date of Birth: Not provided Gender: F

Ethnicity/Race: White/non-Hispanic Consent category: √minor/□assent/□adult

Medical Record ID#: ________________ Lab ID#: ____________ Other DB ID#: ____________

Specimen type: Whole Blood Date Received: 7/01/2012 Date Reported: **9/01/2012**

Referring physician: ________________ Referring Genetic Counselor: ______________________

Type of test ordered: x WGS x WES □ Targeted Capture/Amplicon Sequencing □ Other: _____________

Indication for Testing

***Patient synopsis*:** *SD is a 4 year, 10-month old girl presenting with a history of heart malformations, cardiac arrhythmia, and aortic malformations. SD was born at full-term (40 wks) by spontaneous vaginal delivery. Birth weight 6 lbs. 15 oz.(length: 20 in.) Post-delivery, BPM dropped to 40 ppm and she was admitted to NICU for bradycardia and apneic spells. Echocardiogram revealed a right ventricular apical mass and abnormal ventricular septal motion due to a right ventricular conduction delay. Diagnosis of right bundle branch block established on DOL1 confirmed by 24-hr. Holter Monitoring at age 1 month (ave sinus rate 142/min., range: 101-203/min.); no significant ectopy or pathologic tachycardia was detected. Echocardiograms at 3 months and age 4 years, 10 months revealed spontaneous resolution of the apical mass, normal heart size, structure and normal septal motion, although a slightly prominent ascending aorta (2.5 cm aortic root; >95% on pediatric Roman Reference graph) was noted on the most recent study at age 4 years 10 months.*

Currently, SD has no physical limitations and has no neurological, social, or cognitive deficits. Weight (16.2 kg) and height (106 cm) are in the 25th and 50th percentiles, respectively. There are no dysmorphic features, skeletal or cutaneous abnormalities. She is asymptomatic with regard to her RBBB, heart rate normal with regular rhythm, normal S1, split S2 that varies normally with respiration, but no other extra sounds or murmurs. There is a strong family history of cardiac abnormalities, including the mother (also has RBBB), maternal aunt (deceased at age 7 days with tricuspid atresia and hypoplastic R heart), and maternal uncle (age 32 with coarctation, type II AV block and pulmonary stenosis). The affected uncle had a son who died at age 12 days with severe pulmonary stenosis, hypoplastic R heart and type II AV block.

Clinical Diagnoses Under Consideration (in order of likelihood): arrhythmogenic right ventricular cardiomyopathy; cardiac malformations resulting from genes implicated in heart development; tuberous sclerosis; thoracic aortic aneurysm syndromes.

Known Genetic Associations with the Clinical Diagnosis under Consideration (Known Genes): 25 total - *ACTA2*, *CRELD1*, *DSC2*, *DSG2, DSP, FBN1*, *FOG2*, *GATA4*, *HAND1*, *HAND2, JUP, MYH11*, *MYLK*, *NKX2.5*, *PKP2, PROSIT240*, *RYR2*, *SMAD3, TGFB3, TGFBR1*, *TGFBR2*, *TMEM43, TSC1, TSC2, TTN*

Inheritance Models Considered: autosomal dominant

Patient is Consented to Receive:

x Primary Finding
x Findings Relevant to General Medical Care
◻ Longevity Reporting
◻ Secondary Findings – Pediatric Onset Treatable
◻ Secondary – Pharmacogenetics (Other)
◻ Secondary – Carrier Status
◻ Secondary – Adult Treatable (Onset)
◻ Secondary – Adult Non-Treatable (Onset) (Must be Over 18)
◻ Obtain Raw Data (Requires Additional Genetic Counseling)
x Research (Participation in Phenome-Genome Database)

Results—Primary Findings & Interpretation

Summary: No pathogenic or likely pathogenic variants were identified in the genes known to be related to the clinical condition described on Page 1.

The inability to identify causal variants in the 24 genes listed on page 1 may reflect genotype diversity associated with this phenotype, an incomplete understanding of this phenotype, and/or technical limitations of the platform/data used for variant identification (see Data Quality Below). Detailed methodology can be found in **Appendix I: Methods for Analysis and Interpretation;** other variants identified in genes related to the clinical phenotype listed on Page 1 are shown in Appendix II.

Genetic Counseling

Genetic counseling is necessary for the patient and family to discuss the issues associated with the absence of reportable primary findings.

If your patient elected to participate in **research related to their primary condition**, please have them contact the University of Iowa Institute for Human Genetics Research Genetic Counselor to discuss research findings.

Data Quality

The detection of variants is dependent on data quality. Only data exceeding a specific threshold are evaluated (see Appendix I). Inadequately covered regions in the coding sequence of known genes are found in Appendix II. In aggregate, the data quality was considered:

◻ Excellent 98% or greater of all coding sequence covered by greater than or equal to 4 reads.

x Very Good 95-97.99% of all coding sequence covered by greater than or equal to 4 reads.

◻ Good 90-94.99% of all coding sequence covered by greater than or equal to 4 reads.

◻ Fair 85-89.99% of all coding sequence covered by greater than or equal to 4 reads.

◻ Poor <84% of all coding sequence covered by greater than or equal to 4 reads.

Note: Poor data do not preclude identifying a disease-causing variant but do mean that for a sizable percentage of coding sequence, there was NO analysis and therefore NO conclusions can be made for these regions.

Results- Secondary Findings Relevant to General Medical Care

Variants in some genes may affect a person's response to different medical therapies. At the request of your patient, variants that can impact response to specific medications, radiation therapy or coagulation/clotting are provided. For assistance in interpreting the significance of these variants, please consult the referring Genetic Counselor. These results may or may not be available in your patient's electronic medical record depending if your patient requested these results be included in their electronic medical record.

Pharmacogenomics
As per the challenge we are not reporting these results.

Radiation Therapy
As per the challenge we are not reporting these results.

Coagulation / Clotting
As per the challenge we are not reporting these results.

Longevity Reporting

As more is learned about the role of genetic variants in human health and disease, the interpretation of some of the variants your patient carries may change. Examples of changes include the recognition that a variant believed to be pathogenic is in fact benign, and reclassifying a benign or uncertain variant as pathogenic. At the request of your patient, the Iowa Institute of Human Genetics (IIHG) will (or will not) provide Longevity Reporting by re-analyzing genomic data for the **PRIMARY CONDITION** on an annual basis. These reports will be provided as in the medical record as addendums to the original report and will be available to you when you see your patient in annual follow-up. Please see Addendum III for report updates. For assistance in the interpretation of new data, please consult the referring Genetic Counselor.

Disclaimer/Legal

Genetic testing was conducted with informed consent following certified genetic counseling, including the use of deidentified information for current and future investigational purposes. Patients have the option of changing the status of their consent at any time, which may result in the permanent removal of their information from the IIHG Clinical Diagnostic Service Laboratory information system. If Longevity Reporting has been requested, data are annually reviewed to determine whether there is a change of status for any variants associated with the primary condition. Patients requesting Longevity Reporting will be notified of any change in the status of variants at their annual medical visit.

The performance characteristics of this test were determined by the IIHG Clinical Diagnostic Service. This test has not been cleared or approved by the U.S. Food and Drug Administration (FDA), however the FDA has determined that clearance or approval is unnecessary. This test is for clinical purposes and should not be regarded as investigational or for research. The IIHG Clinical Diagnostic Service is certified under the Clinical Laboratory Improvement Amendments of 1988 (CLIA; revised 2003) as qualified to perform high complexity clinical laboratory testing.

The results for the Primary Finding and Longevity Reporting for the Primary Finding will be included in the patient's electronic medical record. All other results may or may not be available in your patient's electronic medical record depending if your patient requested these results be included in their electronic medical record.

References

1. OMIM: http://omim.org/

2. NCBI dbSNP135 (http://www.ncbi.nlm.nih.gov/projects/SNP/)

3. 1000 Genomes project: October 2011 release (http://www.1000genomes.org/)

4. Exome Variation Server 6500: accessed on June 20, 2012 (http://evs.gs.washington.edu/EVS/)

Supplemental/Addendum Reports

Please find the following attached, dated documents on the IIHG Clinical Diagnostic Service Laboratory website. Appendices are additional information included in all reports and are part of the electronic medical record. Addendums are optional supplemental reports based on patient consent choices and are not part of the electronic medical record. Addendums are only available on the IIHG Clinical Diagnostic Service Laboratory web portal. All appendices and addendums are dated to reflect knowledge and interpretation at the time of dating.

Appendices

Appendix I. Methods for Analysis and Interpretation

Appendix II. Primary Findings Variant Details

Appendix III. Consent Updates

Addendums – Consent Based Findings Only Available on NGS Dashboard, Not Part of this Report

Addendum I. Research Findings Related to the Primary Condition

Addendum II. Secondary Findings (Optional)

Addendum III. Longevity Reports (Consent Driven)

Addendum IV. Obtain Raw Data (Requires Additional Genetic Counseling)

APPENDIX I. METHODS FOR ANALYSIS AND INTERPRETATION

DNA sequencing—0.5-1.0 µg DNA was prepared from the specimen and processed to generate a library of DNA fragments approximately 150 bp in length. These fragments were attached to microspheres and amplified prior to DNA sequencing using paired-end 75bp chemistry on the ABI SOLiD 5500XL platform (Life Technologies/ Applied Biosystems, Carlsbad, CA).

Raw data processing: Quality control parameters applied to raw sequence data include individual base calling (>99% accuracy) and read length (>50 bp) using software provided by the manufacturer. Raw data output files were archived on a protected storage server and routed into an optimized Galaxy Analysis pipeline for downstream analysis.

Bioinformatics analysis: Sequencing reads were aligned to the human reference genome (GRch37, hg19) using the mapreads tool in Lifescope v2.5 to generate BAM files. BAM files were then processed through a custom analysis pipeline in the Galaxy framework including read duplicate identification (Picard tools), indel realignment (GATK), and variant calling with GATK. Coverage analysis for QC was completed using Bedtools. Variant annotation and filtering was completed using a custom variant annotation pipeline that incorporates data from several repositories (1000 Genomes October 2011 release, EVS accessed on June 20, 2012, and dbSNP135) as well as several pathogenicity prediction methods (PolyPhen2, MutationTaster, SIFT, LRT and dbNSFP) .

Data quality analysis and filtering: We required 4 sequencing reads to cover each variant position (4X depth of coverage), and the variant to be present in at least 25% of reads. We filtered out variants with < 30 Phred-like quality score (1/100 chance of error) and/or a quality divided by depth (Q/D) of <5. Variants were filtered based on several inheritance models, but we tolerated missing data in 1 of the parents in a trio without exclusion. Variants were also filtered based on allele frequencies in population-level databases specific to each disease or suspected disease under consideration. For cardiomyopathy under the dominant mode of inheritance, all variants with a MAF ≥0.001 in 1000Genomes and ≥0.0006 in EVS were filtered out.

Inheritance models considered: (*select appropriate)*

x Autosomal dominant	□ X-linked recessive	□ Mitochondrial
□ Autosomal recessive	□ X-linked dominant	□ *de novo*

Copy Number Variant identification: Copy number variant (CNV) locations were ascertained from inspection of the annotated whole genome sequencing data provided. Several sources of information were used to classify CNVs as either clinically benign or potentially pathogenic including the Database of Genomic Variants (DGV; (http://projects.tcag.ca/variation/), the Wellcome Trust Sanger Institute's DECIPHER database (http://decipher.sanger.ac.uk/), the International Standards for Cytogenetic Arrays (ISCA) consortium (https://www.iscaconsortium.org/), the CNV datasets published by Conrad et al. 2009 and Cooper et al. 2011, and our internal CNV database. If a CNV has been classified as benign, it is not considered to be related to the patient's phenotype. CNVs are classified as benign when:

1. Present in the DGV at a frequency of >1% in >1 studies
2. Present in DGV at a frequency of >0.5% in >2 studies
3. Present in our internal clinical chromosomal microarray database at a frequency of >1% (>1500 cases and >200 unaffected parents)
4. Interpreted as BENIGN in >5 cases in the ISCA database
5. Present in the dataset released in Conrad et. al. 2009 and in the same orientation
6. Present in a substantial number of control individuals (>50-100) compared to cases in Cooper et. al. 2011.

Any CNV classified as benign is NOT investigated further. Remaining CNVs that contain at least one validated or reviewed RefSeq gene are classified as either ABNORMAL or as a variant of uncertain significance (VUS). ABNORMAL CNVs are known to be pathogenic (such as a 22q11.2 deletion) or novel and >1Mb in size. VUS are either known susceptibility loci (such as 16p11.2 locus) or otherwise NOT classified as ABNORMAL.

ABNORMAL and VUS CNVs are tested for segregation in the family. CNVs that are NOT benign and segregate with the clinical phenotype are reported as LIKELY PATHOGENIC.

Limitations:

1. Variations larger than 30bp and less than several kilobases are undetectable by both the small insertion/deletion calling and the CNV calling pipelines. This would cause, for instance, an insertion of a retrotransposon to not be reported.
2. Phase information is inferred by sequencing family members, it is not directly observed. This will cause the over-reporting of genes with compound heterozygous mutations when only a single individual is sequenced.
3. Small variations in introns, in UTR, and in regulatory regions are not considered except in cases where splicing would be affected.
4. Identified variations are of lower quality in regions that are highly similar to other parts of the genome. This can cause variations to be missed.
5. Gene annotations and exome definitions are incomplete; this causes disease-causing mutations to be missed.

Inadequately Covered Loci (genes, exons that did not meet Q/C for coverage and are relevant to DDx):

The following regions in the coding sequence of genes known to be related to the clinical diagnosis under consideration were inadequately covered and therefore clinical grade variants in these regions may be present but were not detected.

Gene	Position	Median Cover	Minimum Cover
DSG2	chr18:29078214-29078259	2.1	2
DSG2	chr18:29125683-29126706	44.4	2
DSP	chr6:7542148-7542318	3.1	1
DSP	chr6:7578011-7578119	13.4	1
DSP	chr6:7579507-7580005	35.4	1
DSP	chr6:7579507-7581802	31.3	1
DSP	chr6:7582874-7586111	37.6	2
FOG2	chr8:106813274-106815766	32.0	1
GATA4	chr8:11565821-11566437	1.0	1
HAND1	chr5:153857025-153857568	25.3	1
HAND2	chr4:174449885-174450440	46.9	1
JUP	chr17:39913885-39914036	14.5	2
JUP	chr17:39914650-39914770	10.6	2
JUP	chr17:39919234-39919573	12.7	1
JUP	chr17:39923630-39923832	19.9	1
JUP	chr17:39925220-39925459	15.3	1
JUP	chr17:39925669-39925929	7.5	1
NKX2-5	chr5:172659571-172660212	9.6	1
NKX2-5	chr5:172660345-172660467	2.8	1
NKX2-5	chr5:172660499-172660504	1.4	1

NKX2-5	chr5:172661752-172662086	16.7	1
PKP2	chr12:32993961-32994139	20.5	1
PKP2	chr12:32996115-32996247	1.7	1
PKP2	chr12:33030779-33031477	25.0	1
PKP2	chr12:33049442-33049665	1.0	1
PROSIT240	chr12:116420921-116421345	58.4	1
PROSIT240	chr12:116421984-116422177	31.4	3
PROSIT240	chr12:116428824-116429762	75.0	1
PROSIT240	chr12:116714864-116714936	2.1	1
RYR2	chr1:237753939-237754292	30.0	3
RYR2	chr1:237777338-237778143	44.9	2
RYR2	chr1:237791106-237791380	65.5	3
RYR2	chr1:237870248-237870569	28.3	1
RYR2	chr1:237919587-237919687	14.0	3
RYR2	chr1:237946974-237948272	31.9	1
TGFB3	chr14:76446884-76447236	29.3	1
TTN	chr2:179393254-179393946	73.2	1
TTN	chr2:179394967-179400576	54.9	1
TTN	chr2:179400708-179401302	51.6	1
TTN	chr2:179403266-179403566	41.7	3
TTN	chr2:179407795-179408389	38.3	2
TTN	chr2:179414712-179415000	83.3	1
TTN	chr2:179416356-179418123	47.6	1
TTN	chr2:179418640-179418943	25.3	1
TTN	chr2:179423067-179423364	40.8	2
TTN	chr2:179424037-179441143	60.9	1
TTN	chr2:179443532-179444120	43.7	1
TTN	chr2:179446632-179446935	27.3	1
TTN	chr2:179449002-179449305	55.1	1
TTN	chr2:179453264-179456231	73.8	2
TTN	chr2:179459070-179459373	55.6	2
TTN	chr2:179473934-179474297	35.5	2
TTN	chr2:179489191-179489458	32.3	1
TTN	chr2:179501124-179501526	52.6	3
TTN	chr2:179518153-179518234	0.0	0
TTN	chr2:179518347-179518428	0.0	0
TTN	chr2:179518543-179518624	0.0	0
TTN	chr2:179518738-179518822	1.1	1
TTN	chr2:179518934-179519018	5.4	1
TTN	chr2:179519171-179519261	0.0	0
TTN	chr2:179519471-179519555	6.4	1
TTN	chr2:179522803-179522884	2.0	1
TTN	chr2:179522998-179523082	4.1	1

TTN	chr2:179523194-179523278	9.2	1
TTN	chr2:179527064-179527145	1.7	1
TTN	chr2:179527259-179527343	5.6	3
TTN	chr2:179530434-179530518	2.0	1
TTN	chr2:179532168-179532252	3.1	1
TTN	chr2:179532364-179532448	1.6	1
TTN	chr2:179534099-179534174	4.9	1
TTN	chr2:179540397-179540481	2.2	1
TTN	chr2:179541927-179542014	1.5	1
TTN	chr2:179549056-179549140	29.9	3
TTN	chr2:179560591-179560996	93.8	3
TTN	chr2:179568873-179569134	46.4	2
TTN	chr2:179574292-179574583	45.6	1
TTN	chr2:179580219-179580501	39.4	1
TTN	chr2:179585111-179585390	35.9	1
TTN	chr2:179585647-179585929	30.7	3
TTN	chr2:179591816-179592098	44.4	1
TTN	chr2:179592836-179593124	54.2	3
TTN	chr2:179593617-179593896	33.3	1
TTN	chr2:179596031-179596310	48.9	2
TTN	chr2:179596419-179596698	43.5	1
TTN	chr2:179600237-179600801	56.4	1
TTN	chr2:179603867-179606648	50.6	1
TTN	chr2:179610311-179616766	73.8	1
TTN	chr2:179629253-179629538	17.7	3
TTN	chr2:179634405-179634666	81.3	1
TTN	chr2:179640082-179641776	60.0	2
TTN	chr2:179642430-179642702	45.6	3
TTN	chr2:179648796-179649078	53.4	3
TTN	chr2:179650574-179650868	52.3	1
TTN	chr2:179659648-179659979	14.2	1
TTN	chr2:179665121-179665409	37.5	2

REFERENCES

Adzhubei IA, Schmidt S, Peshkin L, Ramensky VE, Gerasimova A, Bork P, Kondrashov AS, Sunyaev SR. A method and server for predicting damaging missense mutations. Nat Methods 7(4):248-249 (2010).

Chun and Fay. Identification of deleterious mutations within three human genomes. *Genome Research* (2009) 19:1553-1561.

Conrad DF, Pinto D, Redon R, Feuk L, Gokcumen O, Zhang Y, Aerts J, Andrews TD, Barnes C, Campbell P, Fitzgerald T, Hu M, Ihm CH, Kristiansson K, Macarthur DG, Macdonald JR, Onyiah I, Pang AW, Robson S, Stirrups K, Valsesia A, Walter K, Wei J; Wellcome Trust Case Control Consortium, Tyler-Smith C, Carter NP,

Lee C, Scherer SW, Hurles ME. Origins and functional impact of copy number variation in the human genome. Nature. 2010 Apr 1;464(7289):704-12.

Cooper GM, Coe BP, Girirajan S, Rosenfeld JA, Vu TH, Baker C, Williams C, Stalker H, Hamid R, Hannig V, Abdel-Hamid H, Bader P, McCracken E, Niyazov D, Leppig K, Thiese H, Hummel M, Alexander N, Gorski J, Kussmann J, Shashi V, Johnson K, Rehder C, Ballif BC, Shaffer LG, Eichler EE. A copy number variation morbidity map of developmental delay. Nat Genet. 2011 Aug 14;43(9):838-46.

Exome Variant Server, NHLBI GO Exome Sequencing Project (ESP), Seattle, WA (URL: http://evs.gs.washington.edu/EVS/) [08/2012].

Kumar P, Henikoff S, Ng PC. Predicting the effects of coding non-synonymous variants on protein function using the SIFT algorithm. *Nat Protoc. 2009*;4(7):1073-81.

Liu X, Jian X, Boerwinkle E. dbNSFP: a lightweight database of human nonsynonymous SNPs and their functional predictions. Hum Mutat. 2011 Aug;32(8):894-9.

Schwarz JM, Rödelsperger C, Schuelke M, Seelow D. MutationTaster evaluates disease-causing potential of sequence alterations. *Nat Methods. 2010 Aug;7(8):575-6.*

APPENDIX II. PRIMARY FINDINGS VARIANTS DETAILS (Known Genetic Associations with the Clinical Diagnosis)

Patient/Subject Name: Susan Q. Dough (Family 2 proband) Date of Birth: Not provided Gender: F

Ethnicity/Race: White/non-Hispanic Consent category: √minor/□assent/□adult

Medical Record ID#: ____________________ Lab ID#: _______________ Other DB ID#: ______________

Specimen type: _Whole Blood______ Date Received: _7/01/2012________ Date Reported: **9/01/2012**

Referring physician: _________________ Referring Genetic Counselor: _________________________

Type of test ordered: x WGS x WES □ Targeted Capture/Amplicon Sequencing □ Other: _______________

Variants Identified in Genes Associated with the Primary Indication.

No variants were identified in primary disease genes that met inheritance criteria

APPENDIX III. CONSENT, CONSENT UPDATES, NEW HEALTH CONDITIONS

This section includes ORIGINAL consent information and CONSENT UPDATES. Consent updates reflect a change in the information the patient does or does NOT wish to receive. Also included are new health conditions for which the patient is requesting genome analysis. A new abbreviated consent document is required to provide results pertaining to the new condition.

Patient/Subject Name: Susan Q. Dough (Family 2 proband) Date of Birth:Not provided Gender: F

Ethnicity/Race: White/non-Hispanic Consent category: √minor/□assent/□adult

Medical Record ID#: __________________ Lab ID#: _____________Other DB ID#: _____________

Specimen type: _Whole Blood______ Date Received: _7/01/2012________ Date Reported: **9/01/2012**

Referring physician: _________________ Referring Genetic Counselor:________________________

Type of test ordered: x WGS x WES □ Targeted Capture/Amplicon Sequencing □ Other: ______________

Original Consent:

Family 2 Proband consented to receive the following results on ___ /_____ / ____, for history of heart malformations, cardiac arrhythmia, and aortic malformations.

Patient Name Day / Month / Year Condition

Results Patient is Consented to Receive:

x Primary Finding
x Longevity Reporting
□ Secondary – Pharmacogenetics (Other)
□ Secondary – Adult Treatable (Onset)
x Secondary Findings Relevant to General Medical Care
□ Secondary Findings – Pediatric Onset Treatable
□ Secondary – Carrier Status
□ Secondary – Adult Non-Treatable (Onset) (Must be Over 18)
x Research (Participation in Phenome-Genome Database)
□ Obtain Raw Data (Requires Additional Genetic Counseling)

Special Instructions: ____

____

Consent Updated ___ /_____ / ____, for _______________.

Day / Month / Year Condition

Results Patient is Consented to Receive:

□ Primary Finding
□ Longevity Reporting
□ Secondary – Pharmacogenetics (Other)
□ Secondary – Adult Treatable (Onset)
□ Secondary Findings Relevant to General Medical Care
□ Secondary Findings – Pediatric Onset Treatable
□ Secondary – Carrier Status
□ Secondary – Adult Non-Treatable (Onset) (Must be Over 18)
□ Research (Participation in Phenome-Genome Database)
□ Obtain Raw Data (Requires Additional Genetic Counseling)

Special Instructions: ____

ADDENDUM I. RESEARCH FINDINGS RELATED TO THE PRIMARY CONDITION

(ONLY AVAILABLE THROUGH WEBSITE HEALTHCARE PROVIDER PORTAL)

Patient/Subject Name: Susan Q. Dough (Family 2 proband) Date of Birth: Not provided Gender: F

Ethnicity/Race: White/non-Hispanic Consent category: √minor/□assent/□adult

Medical Record ID#: ________________ Lab ID#: ____________Other DB ID#: _____________

Specimen type: _Whole Blood______ Date Received: _7/01/2012________ Date Reported: **9/01/2012**

Referring physician: ________________ Referring Genetic Counselor:________________________

Type of test ordered: x WGS x WES □ Targeted Capture/Amplicon Sequencing □ Other: _____________

A secondary list of genes was generated and filtered for rare/novel variants. To generate this list, we used the primary genes involved in this disorder as seeds to mine for interacting genes using a panel of databases and analysis tools that would generate a dataset of known and predicted protein-protein interactions as well as shared networks and pathways.

1- STRING: http://string-db.org/newstring_cgi/show_input_page.pl?UserId=bI0i5NfHQOSk&sessionId=zv0H2bheWIlW
2- KEGG PATHWAY Database: http://www.genome.jp/kegg/pathway.html
3- GeneGo: http://www.genego.com/
4- GeneDecks V3/ Partner Hunter: http://www.genecards.org/index.php?path=/GeneDecks

We also used the Mammalian phenotype browser for mouse models with similar phenotype to the disorder investigated in this study: http://www.informatics.jax.org/searches/MP_form.shtml

The secondary gene list for arrhythmogenic right ventricular cardiomyopathy comprises 79 genes:

CDH2, ARVCF, CTNNA1, CTNNA2, CTNNA3, CTNNB1, CTNND1, ACTN1, ACTN2, ACTN3, ACTN4, ATP2A2, SLC8A1, MRGPRE, SCN5A, ISM2, FKBP1B, RYR1, PTPLA, CELF2, CDH3, CDH1, CDH4, NPPB, ACE, PKP1, PKP4, CDH17, DSG1, NFKB1, CDH5, PKP3, DSG3, COL17A1, DOCK5, DSC1, DUSP13, IVL, DSPP, DSC3, DSG4, PSEN1, NCSTN, CASP3, CDSN, PNN, PPL, AJAP1, DLG5, MLLT4, PARD3, P2RX6, PERP, TJP1, VEZT, PVRL1, PVRL2, PVRL3, PVRL4, ANAPC1, BAIAP2, CDC27, EXOC2, FARP2, FLNA, FLNB, LMO7, MAPRE1, MAPRE2, NME1, SORBS1, SSX2IP, TLN1, TLN2, VCL, WAS, WASF1, WASL and *ZYX.*

None of the genes listed above carried variants that satisfy the inheritance model or the MAF cutoff.

Whole exome variant analysis for this family was performed by filtering out all synonymous and intronic variants (except for splice site variants) and those that do not meet the mode of inheritance and MAF requirements. Variants in 7 genes were obtained: *HOOK1, BBS12, PIWIL3, RAD51C, FRAS1, CAGE1,* and *PPFIA2.* The variants in these genes are shown in the table below.

List of heterozygote gene variants found in Family 2

GENE	Proband Het/Homo	Type	HGVS_NT	HGVS_PROTEIN	EVS 5400 MAF	1000G MAF	dbSNP135	PhyloP	SIFT	PolyPhen2	LRT	MutationTaster	GERP++	Chr	Start	End	Ref	Obs	Proband %Obs	Proband Cov	Q_VAR	QD
HOOK1	Het	NS	NM_015888:c.2129A>G	NM_015888:p.His710Arg	0.000596		rs34360310	C	T	D	D	D	4.32	chr1	60338579	60338579	A	G	28.60%	7	488.68	14.37
BBS12	Het	NS	NM_152618:c.979T>A	NM_152618:p.Ser327Thr	0.000465		rs116805550	N	T	B	N	N	0.196	chr4	123664026	123664026	T	A	37.50%	8	560.86	15.16
PIWIL3	Het	frameshift	NM_001008496:c.6_7insT											chr22	25158461	25158461	-	A	42.90%	7	393.21	10.92
RAD51C	Het	splice-site	NM_058216:c.571+1G>T											chr17	56774221	56774221	G	T	46.70%	30	1816.2	14.53
FRAS1	Het	NS	NM_025074:c.4293C>A	NM_025074:p.Asp1431Glu										chr4	79328980	79328980	C	A	50.00%	6	605.48	15.14
CAGE1	Het	NS	NM_205864:c.433C>T	NM_205864:p.Arg145Trp										chr6	7374211	7374211	G	A	50.00%	10	933.51	16.97
PPFIA2	Het	NS	NM_003625:c.302C>T	NM_003625:p.Pro101Leu	0.000209	0.001	rs147865064							chr12	82070571	82070571	G	A	66.70%	9	545.89	12.41

C: conserved. D: damaging, P: possibly damaging, B: benign, T: tolerated, N: neutral, NA: not applicable, NS: non-synonymous, Het: heterozygote, Homo: homozygote

Mutations in several of these genes have been linked to other disorders. *BBS12* is involved in Bardet-Biedl syndrome (BBS; MIM:209900) (Stoetzel C et al, 2007); *RAD51C* and *FRAS1* are implicated in Fanconi anemia (FANCO; MIM:613390) and Fraser syndrome (MIM:219000), respectively. Loss of *Hook1* function in mice leads to ectopic positioning of microtubular structures within the spermatid causing the “abnormal-spermatozoon-head-shape” phenotype (Mendoza-Lujambio I et al, 2002), while *PIWIL3* is involved in development and maintenance of germline stem cells and is expressed mainly in adult testis (Sasaki T et al, 2003). *CAGE1* encodes for cancer/testis antigens 3. Finally, *PPFIA2* encodes for liprin-alpha-2 protein expressed only in brain (Serra-Pages C et al, 1998).

INTERPRETATION

These variants **may or may not be associated with the clinical phenotype and the relationship of these genes to your patient’s phenotype is UNKNOWN.** However, variants in these genes are associated with **other diseases** that have no overlapping phenotype with Cardiomyopathies. Therefore, we do not predict them to be disease-causing in this family.

These results are considered **RESEARCH FINDINGS** because a definite association of these genes with the condition has not been verified and the results have not been validated with Sanger sequencing. There is **NO** current robust genetic evidence to implicate these genes with Cardiomyopathies.

Based on the information currently available, these are **variants of unclear clinical significance,** which may or may NOT be involved with disease in your patient. We do **NOT** recommend a change of medical management based on these variants at this time. We encourage your patient to have confirmatory testing by Sanger sequence verification of this variant. In addition, your patient may be interested and/or eligible for participation in further research on this disease.

NOTE

Patients cannot receive these results without first speaking to the IIHG Research Genetic Counselor. If upon speaking with the IIHG Research Genetic Counselor the patient indicates they would like to participate in an active line of research at the University of Iowa, the IIHG Research Genetic Counselor will provide the patient with the contact information of an appropriate Principle Investigator. If the patient would like us to transfer the results of their sequencing test to the researcher, the patient must call or write to the IIHG and request the IIHG Clinical Diagnostic Service Laboratory provide a copy of the genetic sequencing results to the researcher.

REFERENCES

Mendoza-Lujambio I, Burfeind P, Dixkens C, Meinhardt A, Hoyer-Fender S, Engel W, Neesen J. The Hook1 gene is non-functional in the abnormal spermatozoon head shape (azh) mutant mouse. Hum Molec Genet 11:1647-1658, 2002

Sasaki T, Shiohama A, Minoshima S, Shimizu N. Identification of eight members of the Argonaute family in the human genome small star, filled. Genomics 82(3):323-30. 2003

Serra-Pages C, Medley Q G, Tang M, Hart A, Streuli M. Liprins, a family of LAR transmembrane protein-tyrosine phosphatase-interacting proteins. J Biol Chem 273:15611-20, 1998.

Stoetzel C, Muller J, Laurier V, Davis EE, Zaghloul NA, Vicaire S, Jacquelin C, Plewniak F, Leitch CC, Sarda P, Hamel C, de Ravel TJ, Lewis RA, Friederich E, Thibault C, Danse JM, Verloes A, Bonneau D, Katsanis N, Poch O, Mandel JL, Dollfus H. Identification of a novel BBS gene (BBS12) highlights the major role of a vertebrate-specific branch of chaperonin-related proteins in Bardet-Biedl syndrome. Am J Hum Genet 80:1-11, 2007.

ADDENDUM II. UNRELATED CONSENTED SECONDARY FINDINGS

The following categories of results are consent-based options that are ONLY available if your patient has requested these results.

Patient/Subject Name: Susan Q. Dough (Family 2 proband) Date of Birth: Not provided Gender: F
Ethnicity/Race: White/non-Hispanic Consent category: √minor/□assent/□adult
Medical Record ID#: ________________ Lab ID#: ____________ Other DB ID#: ____________
Specimen type: _Whole Blood______ Date Received: _7/01/2012________ Date Reported: **9/01/2012**
Referring physician: ________________ Referring Genetic Counselor: ________________________
Type of test ordered: x WGS x WES □ Targeted Capture/Amplicon Sequencing □ Other: ______________

A. SECONDARY FINDINGS: PEDIATRIC ONSET TREATABLE CONDITIONS

As per the challenge we are not reporting these results.

B. SECONDARY FINDINGS: PHARMACOGENETICS (OTHER)

As per the challenge we are not reporting these results.

C. SECONDARY FINDINGS: CARRIER STATUS

As per the challenge we are not reporting these results.

D. SECONDARY FINDINGS: ADULT ONSET TREATABLE CONDITIONS

As per the challenge we are not reporting these results.

E. SECONDARY FINDINGS: ADULT ONSET NON-TREATABLE CONDITIONS (MUST BE OVER 18)

As per the challenge we are not reporting these results.

ADDENDUM III. LONGEVITY REPORTS

Patient/Subject Name: Susan Q. Dough (Family 2 proband) Date of Birth: Not provided Gender: F

Ethnicity/Race: White/non-Hispanic Consent category: √minor/□assent/□adult

Medical Record ID#: ________________ Lab ID#: ____________ Other DB ID#: ____________

Specimen type: _Whole Blood______ Date Received: _7/01/2012________ Date Reported: **9/01/2012**

Referring physician: ________________ Referring Genetic Counselor: ________________________

Type of test ordered: x WGS x WES □ Targeted Capture/Amplicon Sequencing □ Other: ______________

If your patient has requested Longevity reporting, the Iowa Institute of Human Genetics (IIHG) will re-analyze genomic data for the **PRIMARY CONDITION** on an annual basis for 7 years. After 7 years testing on a new sample should be considered due to modernized sequencing technology. These reports will be provided as in the medical record as addendums to the original report and will be available to you when you see your patient in annual follow-up. For assistance in interpreting the significance of these variants, please consult the referring Genetic Counselor.

The following variants are now known to be disease causing:

	Gene	Variant (cDNA & Protein annotation)	State (heterozygous vs homozygous)	Frequency European Americans	Frequency African Americans	Disease
1						
2						
3						
4						
5						

The following variants were originally reported to be disease causing, but are now known to be benign polymorphisms:

	Gene	Variant (cDNA & Protein annotation)	State (heterozygous vs homozygous)	Frequency European Americans	Frequency African Americans	Disease
1						
2						
3						
4						
5						

ADDENDUM IV. OBTAIN RAW DATA (REQUIRES ADDITIONAL GENETIC COUNSELING)

Patient/Subject Name: Susan Q. Dough (Family 2 proband) Date of Birth: Not provided Gender: F

Ethnicity/Race: White/non-Hispanic Consent category: √minor/□assent/□adult

Medical Record ID#: ___________________ Lab ID#: ______________Other DB ID#: _____________

Specimen type: _Whole Blood______ Date Received: _7/01/2012________ Date Reported: **9/01/2012**

Referring physician: ________________ Referring Genetic Counselor:_______________________

Type of test ordered: x WGS x WES □ Targeted Capture/Amplicon Sequencing □ Other: _____________

________________ received the following data files on ___ /_____ / ____.

Patient Name Day / Month / Year

Bioinformatics fill in fill type options

□ .BAM files

□ VCF files

□ Other: ______________________________________

IOWA INSTITUTE OF HUMAN GENETICS

1000 NEWTON ROAD
IOWA CITY, IA 52242-1442
TEL 319-335-8000/ 800-335-8100
WWW.CCOM.IIHG.ORG/

Genetic Sequencing Report

Patient/Subject Name: Johnny Q. Doh (Family 3 proband) Date of Birth: Not provided Gender: M

Ethnicity/Race: White/non-Hispanic Consent category: √minor/□assent/□adult

Medical Record ID#: ______________Lab ID#: ______________Other DB ID#:________________

Specimen type: Whole Blood Date Received: 7/01/2012 Date Reported: **9/01/2012**

Referring physician: ___________________ Referring Genetic Counselor:___________________

Type of test ordered: x WGS x WES □ Targeted Capture/Amplicon Sequencing □ Other:______________

Indication for Testing

***Patient synopsis**: JD is a 6 year 9 month boy presenting with muscle weakness. His prenatal history is significant for conception facilitated by Clomid, decreased fetal movements, and chronic oligohydramnios. He was born at 35 weeks and 2 days gestation with Apgar scores 6 at one minute and 7 at 5 minutes. His growth parameters at birth were notable for birth length below the third centile, head circumference at the 10th percentile and birthweight at the 75th percentile. His immediate neonatal history was significant for severe hypotonia, respiratory failure, significant feeding difficulties, congenital unilateral elbow dislocation, flexion contractures of arm and legs, and minor facial dysmorphic features. His developmental history was significant for gross motor delays and preserved speech, cognitive and social development.*

Physical examination at 5 months of age revealed myopathic facies, decreased muscle bulk and strength, decreased range of motion in hips and ankles, mild finger contractures, osteoporosis. A muscle biopsy of skeletal muscle at 7 months of age revealed the presence of magenta material in the form of small dots or rod like structures in small fibers. The findings were not classic for ragged red fibers but suggested the presence of internal filamentous structures within the muscle fibers. A muscle biopsy contained no evidence of denervation. Collectively, clinical and pathological findings were most consistent with the diagnosis of nemaline myopathy.

***Clinical Diagnoses Under Consideration (in order of likelihood):** nemaline myopathy, distal arthrogryposis, and mitochondrial myopathies, although the medical history is negative for biochemical markers of these conditions.*

Known Genetic Associations with the Clinical Diagnosis under Consideration (Known Genes): 11 total - *ACTA1, CFL2, KBTBD13, MYH3, MYH8, NEB, TPM2, TMP3, TNNT1, TNNI2, TNNT3*

Inheritance Models Considered: Family history is consistent with an **autosomal recessive** mode of inheritance, although **autosomal dominant de novo changes or X-linked recessive disorders cannot be ruled out**.

Patient is Consented to Receive:

x Primary Finding
□ Longevity Reporting
□ Findings Relevant to General Medical Care
□ Secondary Findings – Pediatric Onset Treatable

□ Secondary – Pharmacogenetics (Other) □ Secondary – Carrier Status
□ Secondary – Adult Treatable (Onset) □ Secondary – Adult Non-Treatable (Onset) (Must be Over 18)
□ Obtain Raw Data (Requires Additional Genetic Counseling)
x Research (Participation in Phenome-Genome Database)

Results—Primary Findings & Interpretation

Summary: The *TPM2* variant c.773-3_773-2insC was detected in a homozygous state (see Table, Appendix II). Heterozygous as well as homozygous null mutations in *TPM2* are causally related to Nemaline Myopathy. The *TPM2* variant c.773-3_773-2insC has been previously reported as a benign polymorphism; rs149115565 (Donner K et al, 2002). *In silico* prediction using Alamut v.2.2 application (Interactive Biosoftware) classified this variant as neutral and not pathogenic.

<u>References</u>

Alamut-HT. http://www.interactive-biosoftware.com/

Donner K, Ollikainen M, Ridanpää M, Christen HJ, Goebel HH, de Visser M, Pelin K, Wallgren-Pettersson C. Mutations in the beta-tropomyosin (TPM2) gene--a rare cause of nemaline myopathy. Neuromuscul Disord. 2002 Feb;12(2):151-8.

CLINICAL CORRELATION is required.

Detailed methodology can be found in **Appendix I: Methods for Analysis and Interpretation;** other variants identified in genes related to the clinical phenotype listed on Page 1 are shown in Appendix II.

Genetic Counseling

Genetic counseling is necessary for the patient and family to discuss the issues associated with the absence of reportable primary findings.

If your patient elected to participate in **research related to their primary condition**, please have them contact the University of Iowa Institute for Human Genetics Research Genetic Counselor to discuss research findings.

Data Quality

The detection of variants is dependent on data quality. Only data exceeding a specific threshold are evaluated (see Appendix I). Inadequately covered regions in the coding sequence of known genes are found in Appendix I. In aggregate, the data quality was considered:

□ Excellent >98% of all coding sequence covered by greater than or equal to 4 reads.

□ Very Good 95-98% of all coding sequence covered by greater than or equal to 4 reads.

x Good 90-94% of all coding sequence covered by greater than or equal to 4 reads.

□ Fair 85-89% of all coding sequence covered by greater than or equal to 4 reads.

□ Poor <84% of all coding sequence covered by greater than or equal to 4 reads.

Note: Poor data do not preclude identifying a disease-causing variant but do mean that for a sizable percentage of coding sequence, there was NO analysis and therefore NO conclusions can be made for these regions.

Results- Secondary Findings Relevant to General Medical Care

Variants in some genes may affect a person's response to different medical therapies. At the request of your patient, variants that can impact response to specific medications, radiation therapy or coagulation/clotting are provided. For assistance in interpreting the significance of these variants, please consult the referring Genetic Counselor. These results may or may not be available in your patient's electronic medical record depending if your patient requested these results be included in their electronic medical record.

Pharmacogenomics
As per the challenge we are not reporting these results.

Radiation Therapy
As per the challenge we are not reporting these results.

Coagulation / Clotting
As per the challenge we are not reporting these results.

Longevity Reporting

As more is learned about the role of genetic variants in human health and disease, the interpretation of some of the variants your patient carries may change. Examples of changes include the recognition that a variant believed to be pathogenic is in fact benign, and reclassifying a benign or uncertain variant as pathogenic. At the request of your patient, the Iowa Institute of Human Genetics (IIHG) will (or will not) provide Longevity Reporting by re-analyzing genomic data for the **PRIMARY CONDITION** on an annual basis. These reports will be provided as in the medical record as addendums to the original report and will be available to you when you see your patient in annual follow-up. Please see Addendum II for report updates. For assistance in the interpretation of new data, please consult the referring Genetic Counselor.

Disclaimer/Legal

Genetic testing was conducted with informed consent following certified genetic counseling, including the use of deidentified information for current and future investigational purposes. Patients have the option of changing the status of their consent at any time, which may result in the permanent removal of their information from the IIHG Clinical Diagnostic Service Laboratory information system. If Longevity Reporting has been requested, data are annually reviewed to determine whether there is a change of status for any variants associated with the primary condition. Patients requesting Longevity Reporting will be notified of any change in the status of variants at their annual medical visit.

The performance characteristics of this test were determined by the IIHG Clinical Diagnostic Service. This test has not been cleared or approved by the U.S. Food and Drug Administration (FDA), however the FDA has determined that clearance or approval is unnecessary. This test is for clinical purposes and should not be regarded as investigational or for research. The IIHG Clinical Diagnostic Service is certified under the Clinical Laboratory Improvement Amendments of 1988 (CLIA; revised 2003) as qualified to perform high complexity clinical laboratory testing.

The results for the Primary Finding and Longevity Reporting for the Primary Finding will be included in the patient's electronic medical record. All other results may or may not be available in your patient's electronic medical record depending if your patient requested these results be included in their electronic medical record.

References

1. OMIM: http://omim.org/

2. NCBI dbSNP135 (http://www.ncbi.nlm.nih.gov/projects/SNP/)

3. 1000 Genomes project: October 2011 release (http://www.1000genomes.org/)

4. Exome Variation Server 6500: accessed on June 20, 2012 (http://evs.gs.washington.edu/EVS/)

Supplemental/Addendum Reports

Please find the following attached, dated documents on the IIHG Clinical Diagnostic Service Laboratory website. Appendices are additional information included in all reports and are part of the electronic medical record. Addendums are optional supplemental reports based on patient consent choices and are not part of the electronic medical record. Addendums are only available on the IIHG Clinical Diagnostic Service Laboratory web portal. All appendices and addendums are dated to reflect knowledge and interpretation at the time of dating.

Appendices

Appendix I. Methods for Analysis and Interpretation

Appendix II. Primary Findings Variant Details

Appendix III. Consent Updates

Addendums – Consent Based Findings Only Available on NGS Dashboard, Not Part of this Report

Addendum I. Research Findings Related to the Primary Condition

Addendum II. Secondary Findings (Optional)

Addendum III. Longevity Reports (Consent Driven)

Addendum IV. Obtain Raw Data (Requires Additional Genetic Counseling)

APPENDIX I. METHODS FOR ANALYSIS AND INTERPRETATION

DNA sequencing—0.5-1.0 µg DNA was prepared from the specimen and processed to generate a library of DNA fragments approximately 150 bp in length. These fragments were attached to microspheres and amplified prior to DNA sequencing using paired-end 75bp chemistry on the ABI SOLiD 5500XL platform (Life Technologies/ Applied Biosystems, Carlsbad, CA).

Raw data processing: Quality control parameters applied to raw sequence data include individual base calling (>99% accuracy) and read length (>50 bp) using software provided by the manufacturer. Raw data output files were archived on a protected storage server and routed into an optimized Galaxy Analysis pipeline for downstream analysis.

Bioinformatics analysis: Sequencing reads were aligned to the human reference genome (GRch37, hg19) using the mapreads tool in Lifescope v2.5 to generate BAM files. BAM files were then processed through a custom analysis pipeline in the Galaxy framework including read duplicate identification (Picard tools), indel realignment (GATK), and variant calling with GATK. Coverage analysis for QC was completed using Bedtools. Variant annotation and filtering was completed using a custom variant annotation pipeline that incorporates data from several repositories (1000 Genomes October 2011 release, EVS accessed on June 20, 2012, and dbSNP135) as well as several pathogenicity prediction methods (PolyPhen2, MutationTaster, SIFT, LRT and dbNSFP).

Data quality analysis and filtering: We required 4 sequencing reads to cover each variant position (4X depth of coverage), and the variant to be present in at least 25% of reads. We filtered out variants with < 30 Phred-like quality score (1/100 chance of error) and/or a quality divided by depth (Q/D) of <5. Variants were filtered based on several inheritance models, but we tolerated missing data in 1 of the parents in a trio without exclusion. Variants were also filtered based on allele frequencies in population-level databases specific to each disease or suspected disease under consideration. For Nemaline myopathy, all variants with a MAF ≥0.01 in 1000Genomes and ≥0.006 in EVS were filtered out.

Inheritance models considered: (*select appropriate)*

□ Autosomal dominant	x X-linked recessive	□ Mitochondrial
x Autosomal recessive	□ X-linked dominant	x *de novo*

Copy Number Variant identification: Copy number variant (CNV) locations were ascertained from inspection of the annotated whole genome sequencing data provided. Several sources of information were used to classify CNVs as either clinically benign or potentially pathogenic including the Database of Genomic Variants (DGV; http://projects.tcag.ca/variation/), the Wellcome Trust Sanger Institute's DECIPHER database (http://decipher.sanger.ac.uk/), the International Standards for Cytogenetic Arrays (ISCA) consortium (https://www.iscaconsortium.org/), the CNV datasets published by Conrad et. al. 2009 and Cooper et. al. 2011, and our internal CNV database. If a CNV has been classified as benign, it is not considered to be related to the patient's phenotype. CNVs are classified as benign when:

1. Present in the DGV at a frequency of >1% in >1 studies
2. Present in DGV at a frequency of >0.5% in >2 studies
3. Present in our internal clinical chromosomal microarray database at a frequency of >1% (>1500 cases and >200 unaffected parents)
4. Interpreted as BENIGN in >5 cases in the ISCA database
5. Present in the dataset released in Conrad et. al. 2009 and in the same orientation
6. Present in a substantial number of control individuals (>50-100) compared to cases in Cooper et. al. 2011.

Any CNV classified as benign is NOT investigated further. Remaining CNVs that contain at least one validated or reviewed RefSeq gene are classified as either ABNORMAL or as a variant of uncertain significance (VUS). ABNORMAL CNVs are known to be pathogenic (such as a 22q11.2 deletion) or novel and >1Mb in size. VUS are either known susceptibility loci (such as 16p11.2 locus) or otherwise NOT classified as ABNORMAL.

ABNORMAL and VUS CNVs are tested for segregation in the family. CNVs that are NOT benign and segregate with the clinical phenotype are reported as LIKELY PATHOGENIC.

Limitations:

1. Variations larger than 30bp and less than several kilobases are undetectable by both the small insertion/deletion calling and the CNV calling pipelines. This would cause, for instance, an insertion of a retrotransposon to not be reported.
2. Phase information is inferred by sequencing family members, it is not directly observed. This will cause the over-reporting of genes with compound heterozygous mutations when only a single individual is sequenced.
3. Small variations in introns, in UTR, and in regulatory regions are not considered except in cases where splicing would be affected.
4. Identified variations are of lower quality in regions that are highly similar to other parts of the genome. This can cause variations to be missed.
5. Gene annotations and exome definitions are incomplete; this causes disease-causing mutations to be missed.

Inadequately Covered Loci (genes, exons that did not meet Q/C for coverage and are relevant to DDx):

The following regions in the coding sequence of genes known to be related to the clinical diagnosis under consideration were inadequately covered and therefore clinical grade variants in these regions may be present but were not detected.

Gene	Position	Median Coverage	Minimum Coverage
ACTA1	chr1:229568016-229568178	8.3	1
ACTA1	chr1:229568733-229568862	24.6	1
CFL2	chr14:35183743-35183746	2.0	2
KBTBD13	chr15:65369153-65370530	6.0	1
MYH3	chr17:10541359-10541749	46.8	2
NEB	chr2:152348900-152349008	16.5	1
NEB	chr2:152410341-152410539	70.0	2
NEB	chr2:152435851-152436163	36.4	1
NEB	chr2:152437310-152437418	0.0	0
NEB	chr2:152437996-152438101	0.0	0
NEB	chr2:152438977-152439181	0.0	0
NEB	chr2:152439968-152440280	0.0	0
NEB	chr2:152442020-152442128	0.0	0
NEB	chr2:152442918-152443023	0.0	0
NEB	chr2:152443887-152444091	0.0	0
NEB	chr2:152446402-152446714	193.8	1
NEB	chr2:152450520-152450832	199.1	1
NEB	chr2:152452573-152452681	2.0	1
NEB	chr2:152453471-152453576	1.2	1
NEB	chr2:152454440-152454644	1.0	1
NEB	chr2:152463119-152463227	1.5	1

NEB	chr2:152464017-152464122	2.8	1
NEB	chr2:152464986-152465190	2.1	1
NEB	chr2:152470789-152471101	98.5	2
NEB	chr2:152500331-152500643	151.1	2
NEB	chr2:152507087-152507399	60.3	3
TNNI2	chr11:1861630-1861674	4.8	3
TNNI2	chr11:1861757-1861886	19.9	2
TNNI2	chr11:1862260-1862437	12.2	3
TNNT1	chr19:55652553-55652670	26.4	1
TNNT1	chr19:55657801-55657834	1.2	1
TNNT1	chr19:55658048-55658075	0.0	0
TPM3	chr1:154155463-154155595	10.1	1

References:

1000 Genomes Project Consortium. A map of human genome variation from population-scale sequencing. Nature **467**, 1061-1073, 2010. Data release October 2011.

Adzhubei IA, Schmidt S, Peshkin L, Ramensky VE, Gerasimova A, Bork P, Kondrashov AS, Sunyaev SR. A method and server for predicting damaging missense mutations. Nat Methods **7**(4):248-249 (2010).

Chun and Fay. Identification of deleterious mutations within three human genomes. *Genome Research* (2009) 19:1553-1561.

Conrad DF, Pinto D, Redon R, Feuk L, Gokcumen O, Zhang Y, Aerts J, Andrews TD, Barnes C, Campbell P, Fitzgerald T, Hu M, Ihm CH, Kristiansson K, Macarthur DG, Macdonald JR, Onyiah I, Pang AW, Robson S, Stirrups K, Valsesia A, Walter K, Wei J; Wellcome Trust Case Control Consortium, Tyler-Smith C, Carter NP, Lee C, Scherer SW, Hurles ME. Origins and functional impact of copy number variation in the human genome. Nature. 2010 Apr 1;464(7289):704-12.

Cooper GM, Coe BP, Girirajan S, Rosenfeld JA, Vu TH, Baker C, Williams C, Stalker H, Hamid R, Hannig V, Abdel-Hamid H, Bader P, McCracken E, Niyazov D, Leppig K, Thiese H, Hummel M, Alexander N, Gorski J, Kussmann J, Shashi V, Johnson K, Rehder C, Ballif BC, Shaffer LG, Eichler EE. A copy number variation morbidity map of developmental delay. Nat Genet. 2011 Aug 14;43(9):838-46.

Exome Variant Server, NHLBI GO Exome Sequencing Project (ESP), accessed June 20, 2012. Seattle, WA (URL: http://evs.gs.washington.edu/EVS/) [08/2012].

Kumar P, Henikoff S, Ng PC. Predicting the effects of coding non-synonymous variants on protein function using the SIFT algorithm. *Nat Protoc. 2009*;4(7):1073-81.

Liu X, Jian X, Boerwinkle E. dbNSFP: a lightweight database of human nonsynonymous SNPs and their functional predictions. Hum Mutat. 2011 Aug;32(8):894-9.

Schwarz JM, Rödelsperger C, Schuelke M, Seelow D. MutationTaster evaluates disease-causing potential of sequence alterations. Nat Methods. 2010 Aug;7(8):575-6.

APPENDIX II. PRIMARY FINDINGS VARIANTS DETAILS (Known Genetic Associations with the Clinical Diagnosis)

Patient/Subject Name: Johnny Q. Doh (Family 3 proband) Date of Birth: Not provided Gender: M

Ethnicity/Race: White/non-Hispanic Consent category: √minor/□assent/□adult

Medical Record ID#: ______________Lab ID#: ______________Other DB ID#:________________

Specimen type: Whole Blood Date Received: 7/01/2012 Date Reported: **9/01/2012**

Referring physician: ___________________ Referring Genetic Counselor:___________________

Type of test ordered: x WGS x WES □ Targeted Capture/Amplicon Sequencing □ Other:______________

Variants Identified in Genes Associated with the Primary Indication.

GENE	Proband Het/Homo	Type	HGVS_NT	HGVS_PROTEIN	EVS 5400 MAF	1000G MAF	dbSNP135	Chr	Start	End	Ref	Obs	Proband %Obs	Proband Cov	Q_VAR	QD
TPM2	homozygote	splice_site	NM_003289: c.773-3_773-2insC	n/a	n/a	n/a	rs149115565	chr9	35683241	35683241	-	G	78%	18	1088.38	15.33

APPENDIX III. CONSENT, CONSENT UPDATES, NEW HEALTH CONDITIONS

This section includes ORIGINAL consent information and CONSENT UPDATES. Consent updates reflect a change in the information the patient does or does NOT wish to receive. Also included are new health conditions for which the patient is requesting genome analysis. A new abbreviated consent document is required to provide results pertaining to the new condition.

Patient/Subject Name: Johnny Q. Doh (Family 3 proband) Date of Birth: Not provided Gender: M

Ethnicity/Race: White/non-Hispanic Consent category: √minor/□assent/□adult

Medical Record ID#: _____________Lab ID#: _____________Other DB ID#:________________

Specimen type: Whole Blood Date Received: 7/01/2012 Date Reported: **9/01/2012**

Referring physician: __________________ Referring Genetic Counselor:__________________

Type of test ordered: x WGS x WES □ Targeted Capture/Amplicon Sequencing □ Other:_____________

Original Consent:

________________ consented to receive the following results on ___ /_____ / ____, for _______________.

Patient Name Day / Month / Year Condition

Results Patient is Consented to Receive:

x Primary Finding
□ Longevity Reporting
□ Secondary – Pharmacogenetics (Other)
□ Secondary – Adult Treatable (Onset)
□ Secondary Findings Relevant to General Medical Care
□ Secondary Findings – Pediatric Onset Treatable
□ Secondary – Carrier Status
□ Secondary – Adult Non-Treatable (Onset) (Must be Over 18)
x Research (Participation in Phenome-Genome Database)
□ Obtain Raw Data (Requires Additional Genetic Counseling)

Special Instructions: ____

____

Consent Updated ___ /_____ / ____, for _______________.

Day / Month / Year Condition

Results Patient is Consented to Receive:

□ Primary Finding
□ Longevity Reporting
□ Secondary – Pharmacogenetics (Other)
□ Secondary – Adult Treatable (Onset)
□ Secondary Findings Relevant to General Medical Care
□ Secondary Findings – Pediatric Onset Treatable
□ Secondary – Carrier Status
□ Secondary – Adult Non-Treatable (Onset) (Must be Over 18)
□ Research (Participation in Phenome-Genome Database)
□ Obtain Raw Data (Requires Additional Genetic Counseling)

Special Instructions: ____

ADDENDUM I. RESEARCH FINDINGS RELATED TO THE PRIMARY CONDITION

(ONLY AVAILABLE THROUGH WEBSITE HEALTHCARE PROVIDER PORTAL)

Patient/Subject Name: Johnny Q. Doh (Family 3 proband) Date of Birth: Not provided Gender: M

Ethnicity/Race: White/non-Hispanic Consent category: √minor/□assent/□adult

Medical Record ID#: _____________ Lab ID#: _____________ Other DB ID#: _______________

Specimen type: Whole Blood Date Received: 7/01/2012 Date Reported: **9/01/2012**

Referring physician: __________________ Referring Genetic Counselor: __________________

Type of test ordered: x WGS x WES □ Targeted Capture/Amplicon Sequencing □ Other: _____________

Whole exome variant analysis for this family was performed by filtering out all synonymous and intronic variants (except for splice site variants) and those that do not meet the mode of inheritance and MAF requirements. Variants in 3 genes (CDC27, HOMEZ and TCHH) for the recessive mode of inheritance and 101 genes for the *de novo* model were found in this family.

In order to identify a subset of promising candidate genes and variants with biological relevance to the disorder in this cohort of 101 genes, we generated in parallel a secondary list of genes. To generate this list, we used the primary genes involved in this disorder as seeds to mine for interacting genes using a panel of databases and analysis tools that would generate a dataset of known and predicted protein-protein interactions as well as shared networks and pathways.

1- STRING: http://string-db.org/newstring_cgi/show_input_page.pl?UserId=bI0i5NfHQOSk&sessionId=zv0H2bheWIlW

2- KEGG PATHWAY Database: http://www.genome.jp/kegg/pathway.html

3- GeneGo: http://www.genego.com/

4- GeneDecks V3/ Partner Hunter: http://www.genecards.org/index.php?path=/GeneDecks

We also used the Mammalian phenotype browser for mouse models with similar phenotype to the disorder investigated in this study (http://www.informatics.jax.org/searches/MP_form.shtml).

The secondary gene list for Nemaline myopathy comprises 195 genes:

ACTG2, ACTC1, ACTG1, ACTA2, ACTB, EMD, ACTR2, VCL, RAB4A, CDC42, SRF, CAV1, MYH14, TJP3, DMD, OXSR1, EEA1, TUBA1A, TUBG1, FLNA, BTD, DHCR7, EHHADH, HSD17B4, HTR2C, KCNJ6, NDN, AFG3L2, AR, CRYAB/HSPB2, VCP, TNNT2, UTM, DMPK, DNAJC5, DYSF, GAA, GALC, TCHH , GAN, ITGB1, KIF1B, LAMA2, PTGS1, CAV2, CRKL, NEBL, DNMBP, MAP3K10, SH3RF2, TNNI2, NCF1, NCF4, DOCK1, ACTR3, LIMK2, AP2M1, PPP1R12A, APPL1, TNNI3, ARHGAP42, NFATC2, NFATC3, NFATC4, OLIG2, PLOD1, SEC24B, NUTF2, PRB4, MTMR14, GSN, CDH1, DIAPH2, MARK3, TPM1, RAB25, MYH9, MYH10, CTTN, DNM2, SLC6A9, SLC7A7, SMN1, SOD2, ZIC1, GAS2, APC2, CDH2, PRKCA, MACF1, TNNC2, ABI1, CSK, ARHGEF7, HOMEZ, GYS1, NOXA1, HCLS1, LASP1, SNX33, PHKA1, ME3, FZD7, NPHP1, STAC3, NRAP, TCP10L2, TNNT1, SRC, TRIP10, PSMA1, SENP8, SORBS2, MPP4, BIN1, RUSC2, ITSN1, MYL1, SNX9, NCK1, MYH7, MYOT, SLN, CASQ1, DUSP13, TNNC1, RPS6KB1, CNN1, GAS7, COTL1, MYOF, MAP1A, IQGAP1, SMTN, MYO9B, PRKCE, GC, MYO6, TMSB4X, PLD1, CTAGE15P, PPP1R9B, CACNA1S, MYLPF, PLCD4, PLN, RYR1, EEF2K, CASQ2, MMP3, BEST3, STRN3, P2RY2, PPP1CB, SRL, CACNG1, HSPB6, JSRP1, HRC, CILP, MYLK3, COMP, EPM2A, PNPLA8, ANO5, ITGB1BP2, INPP5A, CDC27, PLA2G2A, TMEM38A, MAPKAPK5, TRDN, SLC24A3, HOMER2, LAMA1, MYPN, FBLN2, CAV3, THBS4, PGF, TPM4, MYOD1, DUSP3, MYL3, PRKAA2, PTP4A3, PITX2, KCNJ11, PRPH2, GRP, RAPSN, PPP2R3A, CDH15, UCP3 and SNTA1.

A subset of 6 genes is shared between both lists: LAMA1, ME3, MTMR14, GYS1, MYOF and NEBL.

List of gene variants found in Family 3

Gene	Porband Het/Homo	Type	HGVS_NT	HGVS_PROTEIN	EVS 5400 MAF	1000 G MAF	db SNP	PhyloP	SIFT	PolyPhen2	LRT	MutationTaster	GERP++	Chr	Start	End	Ref	Obs	Proband % Obs	Proband Cov	Q_Var	QD
De novo																						
**LAMA1**	Homo	NS	NM_005559:c.2515C>T	NM_005559:p.Pro839Ser	0.0005		rs143031283	C	D	D	D	D	5.12	chr18	7023349	7023349	G	A	83.3	6	136.92	22.82
**ME3**	Het	NS	NM_006680:c.1631T>C	NM_006680:p.Val544Ala				C	D	D	D	D	5.13	chr11	86153885	86153885	A	G	40	5	31.51	6.3
**MTMR14**	Het	NS	NM_001077525:c.1558C>T	NM_001077525:p.His520Tyr				C	D	D	D	D	5.37	chr3	9731772	9731772	C	T	50	4	33.75	8.44
**NEBL**	Het	NS	NM_006393:c.2494G>A	NM_006393:p.Asp832Asn				C	D	D	D	N	5.64	chr10	21101722	21101722	C	T	33.3	6	30.22	5.04
**GYS1**	Het	NS	NM_002103:c.62A>G	NM_002103:p.Glu21Gly				C	T	B	D	D	4.66	chr19	49496308	49496308	T	C	37.5	8	61.18	7.65
**MYOF**	Het	NS	NM_013451:c.1141G>A	NM_013451:p.Val381Ile				C	T	P	D	D	5.34	chr10	95159229	95159229	C	T	33	6	31.13	5.19
**Recessive mode of inheritance**																						
**CDC27**	Het	frameshift	NM_001256:c.714delT				rs112848754							chr17	45234407	45234407	A	-	38%	8	146.93	7.35
**CDC27**	Het	NS	NM_001256:c.1460T>C	NM_001256:p.Ile487Thr			rs75894512	C	T	NA	D	D	5.57	chr17	45219310	45219310	A	G	44%	16	204.29	4.54
**HOMEZ**	Het	NS	NM_020834:c.1423C>G	NM_020834:p.Gln475Glu	0.002									chr14	23745014	23745014	G	C	35%	17	275.7	11.03
**HOMEZ**	Het	deletion	NM_020834:c.1636_1640delATG				rs148005528							chr14	23744801	23744803	CAT	-	29%	7	151.54	7.22
**TCHH**	Het	NS	NM_007113:c.3193G>A	NM_007113:p.Gly1065Arg				N	NA	NA	NA	N	-5.7	chr1	1.52E+08	152082500	C	T	38%	8	109.68	6.09
**TCHH**	Het	NS	NM_007113:c.3329G>A	NM_007113:p.Cys1110Tyr				N	NA	NA	NA	N	-1.5	chr1	1.52E+08	152082364	C	T	33%	6	33.6	3.05

C: conserved. D: damaging, P: possibly damaging, B: benign, T: tolerated, N: neutral, NA: not applicable, Het: heterozygote, Homo: homozygote, NS: non-synonymous

Variants in the *MTMR14* and *MYOF* genes are of particular interest. They have high scores for pathogenicity and conservation. *MTMR14* and *MYOF* may play modifier roles in centronuclear myopathy and the muscular dystrophy phenotype, respectively.

MYOF

Myoferlin (MYOF) belongs to the family of ferlins. It contains a type II transmembrane domain and six C2 domains, which play a role in calcium-dependent membrane fusion events (Britton S et al, 2000). C2 domains are known to bind Ca^{2+}, phospholipids and lipid bilayers (Davletov BA et al, 1993), as well as other proteins in Ca^{2+}-dependent and -independent interactions (Li C et al, 1995).

Myoferlin is expressed in heart and skeletal muscle (Davis et al, 2000). In the myoferlin-null mouse, there is a defective endocytic recycling and decreased body and muscle masses (Doherty KR et al, 2005). In disease and injury models, myoferlin appears to have a role in the muscle regeneration and repair (Demonbreun AR et al, 2010).

Mutations in Dysferlin, another member of the ferlin family, have been linked to LGMD 2B (limb-girdle muscular dystrophy type 2B; MIM253601). It has been suggested that since the phenotype in LGMD 2B is highly variable, genetic modifiers may play a role in mediating phenotypic severity; *MYOF* was named as a possible candidate (Davis DB et al, 2000).

The variant discovered in the proband in this family is a change of a conserved valine at position 381 to an isoleucine (p.Val381Ile). This change is predicted to be damaging by LRT and MutationTaster. The variant is located in the third C2 (C2C) domain of myoferlin and may act by impairing the capacity of myoferlin to bind directly to phospholipids and Ca^{2+} or by disturbing the scaffolding of other proteins, thus impairing vesicle trafficking necessary for normal muscle growth and repair.

MTMR14

The myotubularin-related protein 14 (*MTMR14*: MIM611089), also called *JUMPY* or the muscle-specific inositol phosphatase (*MIP*), shares similar enzymatic activity and substrate specificity with myotubularins (Shen J, 2009). The *MTMR14* gene encodes a muscle-specific inositide phosphatase that acts specifically on phosphatidylinositol 3,5-bisphosphate (*PI(3,5)P2*), and plays a critical role in skeletal muscle calcium homeostasis (Romero-Suarez et al, 2010). *MTMR14* protein is predominantly expressed in skeletal and heart muscle (Shen et al, 2009).

MTMR14 regulates autophagy, which depends on both PtdIns3P and PtdIns(3,5)P2 (Gibbs EM et al, 2010), as well as excitation–contraction coupling (Dowling JJ et al, 2010). Mutations in 3 myotubularins are involved in several neuromuscular disorders: X-linked centronuclear myopathy caused by mutations in *MTM1* gene (MIM 310400); Charcot-Marie-Tooth neuropathies CMT4B1 and CMT4B2 caused by *MTMR2* (MIM601382) and *MTMR13* (MIM604563) mutations, respectively. Inactivating heterozygous variants in *MTMR14* have been reported in two patients with autosomal centronuclear myopathy - p.Arg336Gln and p.Tyr462Cys (Tosch et al, 2006). The patient carrying p.Tyr462Cys also carried a missense mutation in *DNM2* (Glu368Ly) implicated in autosomal dominant centronuclear myopathy. It has been postulated that either *MTMR14* is involved in recessive centronuclear myopathy and the second mutation could not be detected or that it represents a modifier for the centronuclear myopathy phenotype (Tosch et al, 2006). A recent study by Hnia et al, suggested that the loss of *MTMR14* is not the cause for early-onset centronuclear myopathy in the *Mtmr14* knock out mouse and Zebrafish (Hnia K et al, 2011 and 2012).

The mutation found in the proband in the present study is a p.His520Tyr in exon 17 of *MTMR14*. This exon appears to be alternatively spliced and it does not code for the phosphatase domain involved in physiologic function of the protein (Tosch et al, 2006). However the histidine at position 520 is well conserved across species and the change to tyrosine is predicted to be damaging (SIFT, PolyPhen, LRT, and MutationTaster). This variant might have a dominant effect by means of haploinsufficiency that would lead to a dysregulation of autophagy and the accumulation of PtdIns(3,5)P(2) and PtdIns (3,4)P(2) impairing muscle function.

LAMA1

LAMA1 (MIM150320) expression in adults is highly restricted, being observed primarily in the central nervous system, eye, kidney, digestive system, and reproductive organs. It is not expressed in the muscle (Falk M et al, 1999). Although Laminin-2 chain (*LAMA2*; MIM156225) deficiency in humans and mice leads to severe forms of congenital muscular dystrophy (MIM607855), deficiency of Lama1 has been involved in retinal vasculopathy (Edwards MM et al, 2010).

The p.Pro839Ser variant found in the proband in this family is NOT predicted to be the cause of the nemaline myopathy phenotype.

NEBL

Although *NEBL* represents an excellent candidate for Nemaline myopathy because of its high homology to Nebulin (NEB: MIM161650), it is excluded as the cause of the disorder in this family because the protein is exclusively expressed in the heart and mutations in this gene have been linked to cardiomyopathies.

GYS1 and ME3

Mutations in *GYS1* (MIM138570) have been linked to Glycogen Storage Disease (GSD; MIM611556) and the p.Glu21Gly variant found in the proband is not predicted to be involved in Nemaline myopathy phenotype.

ME3 (MIM604626) is a malic enzyme that catalyzes the oxidative decarboxylation of malate to pyruvate. It is highly expressed in heart, skeletal muscle, brain, and other tissues. The variant p.Glu21Gly is found *de novo* in the proband, and although conserved, it is not predicted to be pathogenic by either SIFT or PolyPhen.

CDC27, HOMEZ and TCHH

Based on information available about these genes and their functions, they are not believed to play a role in the physiopathology of Nemaline myopathy. *CDC27* (MIM116946) is a subunit of the anaphase-promoting complex. *TCHH* (Trichohyalin; MIM190370) confers mechanical strength to the hair follicle inner root sheath and is thought to be associated with hair morphology. *HOMEZ (*MIM608119) is a homeodomain leucine zipper containing factor (MIM608119) whose function has not been elucidated.

INTERPRETATION

The genetic variants described above **may or may not** be associated with the clinical phenotype in this patient. **The relationship of these genes to your patient's phenotype is UNKNOWN.**

These results are considered **RESEARCH FINDINGS** because a definite association of these genes with the condition has not been verified and the results have not been validated with Sanger sequencing. There is NO current robust genetic evidence to implicate these genes with disease.

Based on the information currently available, these are **variants of unclear clinical significance**, which may or may NOT be involved with disease in your patient. We do NOT recommend a change of medical management based on this variant at this time. We encourage your patient to have confirmatory testing by Sanger sequence verification of this variant. In addition, your patient may be interested and/or eligible for participation in further research on this disease.

NOTE

Patients cannot receive these results without first speaking to the IIHG Research Genetic Counselor. If upon speaking with the IIHG Research Genetic Counselor the patient indicates they would like to participate in an active line of research at the University of Iowa, the IIHG Research Genetic Counselor will provide the patient with the contact information of an appropriate Principle Investigator. If the patient would like us to transfer the results of their sequencing test to the researcher, the patient must call or write to the IIHG and request the IIHG Clinical Diagnostic Service Laboratory provide a copy of the genetic sequencing results to the researcher.

REFERENCES

Britton S, Freeman T, Vafiadaki E, Keers S, Harrison R, Bushby K, Bashir R. The third human FER-1-like protein is highly similar to dysferlin. Genomics 68:313-321, 2000.

Davis DB. Delmonte AJ, Ly, CT, McNally EM. Myoferlin, a candidate gene and potential modifier of muscular dystrophy. Hum Molec Genet 9:217-226, 2000.

Davletov BA and Sudhof TC. A single C2 domain from synaptotagmin I is sufficient for high affinity Ca2+/phospholipid binding. J Biol Chem 268:26386–26390, 1993.

Demonbreun AR, Lapidos KA, Heretis K, Levin S, Dale R, Pytel P, Svensson EC, McNally EM. Myoferlin regulation by NFAT in muscle injury, regeneration and repair. J Cell Sci123(Pt 14):2413-22, 2010.

Doherty KR, Cave A, Davis DB, Delmonte AJ, Posey A, Earley JU, Hadhazy M, McNally EM. Normal myoblast fusion requires myoferlin. Development132(24):5565-75. 2005.

Dowling JJ, Low SE, Busta AS, Feldman EL. Zebrafish MTMR14 is required for excitation-contraction coupling, developmental motor function and the regulation of autophagy. Hum Mol Genet 19(13):2668-81, 2010.

Gibbs EM, Feldman EL, Dowling JJ. The role of MTMR14 in autophagy and in muscle disease. Autophagy 6(6):819-20, 2010.

Hnia K, Kretz C, Amoasii L, Böhm J, Liu X, Messaddeq N, Qu CK, Laporte J. Primary T-tubule and autophagy defects in the phosphoinositide phosphatase Jumpy/MTMR14 knockout mice muscle. Adv Enzyme Regul 52(1): 98-107, 2011.

Hnia K, Vaccari I, Bolino A. Myotubularin phosphoinositide phosphatases: cellular functions and disease pathophysiology. Trends Mol Med 18(6):317–327, 2012.

Li C, Ullrich B, Zhang JZ, Anderson RG, Brose N and Sudhof TC. Ca(2+)-dependent and -independent activities of neural and non-neural synaptotagmins. Nature 375:594–599,1995.

Romero-Suarez S, Shen J, Brotto L, Hall T, Mo C, Valdivia HH, Andresen J, Wacker M, Nosek T M, Qu CK, Brotto M. Muscle-specific inositide phosphatase (MIP/MTMR14) is reduced with age and its loss accelerates skeletal muscle aging process by altering calcium homeostasis. Aging 2:504-513, 2010.

Shen J, Yu,WM, Brotto M, Scherman JA, Guo C, Stoddard C, Nosek TM, Valdivia HH, Qu CK. Deficiency of MIP/MTMR14 phosphatase induces a muscle disorder by disrupting Ca(2+) homeostasis. Nature Cell Biol 11:769-776, 2009.

Tosch,V, Rohde HM, Tronchere H, Zanoteli E, Monroy N, Kretz C, Dondaine N, Payrastre B, Mandel JL, Laporte J. A novel PtdIns3P and PtdIns(3,5)P2 phosphatase with an inactivating variant in centronuclear myopathy. Hum Molec Genet 15:3098-3106, 2006.

ADDENDUM II. UNRELATED CONSENTED SECONDARY FINDINGS

The following categories of results are consent-based options that are ONLY available if your patient has requested these results.

Patient/Subject Name: Johnny Q. Doh (Family 3 proband) Date of Birth: Not provided Gender: M

Ethnicity/Race: White/non-Hispanic Consent category: √minor/□assent/□adult

Medical Record ID#: ______________Lab ID#: ______________Other DB ID#:________________

Specimen type: _Whole Blood_____ Date Received: _7/01/2012____ Date Reported: **9/01/2012**

Referring physician: ___________________ Referring Genetic Counselor:___________________

Type of test ordered: x WGS x WES □ Targeted Capture/Amplicon Sequencing □ Other:_____________

A. SECONDARY FINDINGS: PEDIATRIC ONSET TREATABLE CONDITIONS

As per the challenge we are not reporting these results.

B. SECONDARY FINDINGS: PHARMACOGENETICS (OTHER)

As per the challenge we are not reporting these results.

C. SECONDARY FINDINGS: CARRIER STATUS

As per the challenge we are not reporting these results.

D. SECONDARY FINDINGS: ADULT ONSET TREATABLE CONDITIONS

As per the challenge we are not reporting these results.

E. SECONDARY FINDINGS: ADULT ONSET NON-TREATABLE CONDITIONS (MUST BE OVER 18)

As per the challenge we are not reporting these results.

ADDENDUM III. LONGEVITY REPORTS

Patient/Subject Name: Johnny Q. Doh (Family 3 proband) Date of Birth: Not provided Gender: M

Ethnicity/Race: White/non-Hispanic Consent category: √minor/□assent/□adult

Medical Record ID#: _____________Lab ID#: _____________Other DB ID#:_______________

Specimen type: Whole Blood Date Received: 7/01/2012 Date Reported: **9/01/2012**

Referring physician: __________________ Referring Genetic Counselor:__________________

Type of test ordered: x WGS x WES □ Targeted Capture/Amplicon Sequencing □ Other:_____________

If your patient has requested Longevity reporting, the Iowa Institute of Human Genetics (IIHG) will re-analyze genomic data for the **PRIMARY CONDITION** on an annual basis for 7 years. After 7 years testing on a new sample should be considered due to modernized sequencing technology. These reports will be provided as in the medical record as addendums to the original report and will be available to you when you see your patient in annual follow-up. For assistance in interpreting the significance of these variants, please consult the referring Genetic Counselor.

The following variants are now known to be disease causing:

	Gene	Variant (cDNA & Protein annotation)	State (heterozygous vs homozygous)	Frequency European Americans	Frequency African Americans	Disease
1						
2						
3						
4						
5						

The following variants were originally reported to be disease causing, but are now known to be benign polymorphisms:

	Gene	Variant (cDNA & Protein annotation)	State (heterozygous vs homozygous)	Frequency European Americans	Frequency African Americans	Disease
1						
2						
3						
4						
5						

ADDENDUM IV. OBTAIN RAW DATA (REQUIRES ADDITIONAL GENETIC COUNSELING)

Patient/Subject Name: Johnny Q. Doh (Family 3 proband) Date of Birth: Not provided Gender: M

Ethnicity/Race: White/non-Hispanic Consent category: √minor/□assent/□adult

Medical Record ID#: _____________Lab ID#: _____________Other DB ID#:_______________

Specimen type: Whole Blood Date Received: 7/01/2012 Date Reported: **9/01/2012**

Referring physician: _________________ Referring Genetic Counselor:__________________

Type of test ordered: x WGS x WES □ Targeted Capture/Amplicon Sequencing □ Other:____________

________________ received the following data files on ___ /_____ / ____.

Patient Name Day / Month / Year

Bioinformatics fill in fill type options

□ .BAM files

□ VCF files

□ Other: ______________________________________
